# Combined Modeling Approaches for Assessing Sodium-Iodide Symporter Inhibition

**DOI:** 10.1021/acs.jcim.5c02855

**Published:** 2026-01-23

**Authors:** Julia Kandler, Ayse Sıla Kantarçeken, Aljoša Smajić, Gerhard F. Ecker

**Affiliations:** Department of Pharmaceutical Sciences, 27258 University of Vienna, Josef-Holaubek-Platz 2, 1090 Vienna, Austria

## Abstract

The sodium-iodide symporter (NIS, SLC5A5) plays a crucial role in thyroid hormone synthesis. Especially during brain development, correct thyroid signaling is of critical importance. Hence, inhibition of this transporter can lead to neurodevelopmental disorders, such as lowered IQ or autism. In order to uncover environmental chemicals with the potential of causing developmental neurotoxicity (DNT), NIS was selected for modeling. To support next-generation risk assessment, in silico-based methods were utilized. Docking-based virtual screening workflows of a library of compounds with experimentally determined inhibitory activity on NIS were applied. In addition, machine learning (ML) models based on random forest (RF), extreme gradient boosting (XGB), and support vector machines (SVM) were trained using extended-connectivity fingerprints 4 (ECFP4) and continuous and data-driven descriptors (CDDDs) with 9-fold cross validation to discriminate between NIS inhibiting and noninhibiting compounds. Ultimately, combining ML and docking predictions improved discrimination, achieving an area under the receiver operating characteristic curve (ROC AUC) of 0.77. Thresholds for optimal discrimination between actives and inactives were determined using kernel density estimate plots, at which a Matthews correlation coefficient (MCC) of 0.32, and a balanced accuracy (BA) of 0.78 were achieved on the internal test set. By combining ML predictions with docking scores and training on a larger, more diverse data set of 1412 compounds, this study provides a novel and robust framework for NIS inhibition prediction, which constitutes a new approach method in toxicological risk assessment.

## Graphical abstract

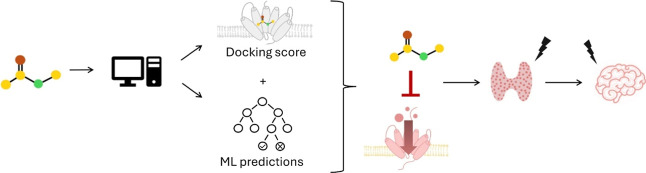

## Introduction

Several processes involved in human brain development have been shown to be vulnerable to external stimuli. Pre- and postnatal exposure to environmental chemicals has been reported as a key contributor to the occurrence of developmental neurotoxicity (DNT). Approximately 10–15% of newborns are affected by DNT, such as autism spectrum disorder, attention deficit disorder, and reduced IQ with increasing prevalence. Hence, an urgent need for reliable test methods exists.^1–3^ Traditionally, animal models have been widely used for chemical safety assessment. However, these models often pose significant limitations, including restricted predictive value for human biology, animal distress, and high time and resource demands. Consequently, animal-free new approach methods, including in vitro, in chemico, and in silico models, have been developed.^4,5^ In the course of this paradigm shift in toxicology, the adverse outcome pathway (AOP) concept has emerged as a systematic approach for linking exposure to stressors to concrete physiological pathways. The AOP-Wiki database (https://aopwiki.org/), which is guided and scientifically reviewed by the Organization for Economic Co-operation and Development (OECD), provides access to curated information on these pathways.^6^ AOPs are triggered by stressors which interact with biological targets, a so-called molecular initiating event (MIE). MIEs are followed by a sequence of key events leading to cellular, molecular, structural, and functional changes in biological systems, ultimately resulting in an adverse outcome. Nevertheless, it is important to note that a single AOP is not able to fully explain the complex process leading to DNT. Hence, a network of AOPs rather than a single AOP enables a better representation of the overall mechanistic understanding of DNT.^3,4,7^ Several reported MIEs leading to DNT are associated with the disruption of thyroid hormone homeostasis. One of the proteins essential for a correct thyroid hormone synthesis is the sodium-iodide symporter (NIS).^8^ NIS transports iodide against its electrochemical gradient by coupling it to the inward transport of two sodium ions, which move down their electrochemical gradient into the thyroid gland. Subsequently, iodide is oxidized and coupled with tyrosyl residues. Finally, the iodotyrosines are coupled leading to the formation of 3,5,3′,5′-tetraiodo-l-thyronine (T4) and 3,5,3′-triiodo-l-thyronine (T3).^9–11^ The inhibition of NIS results in decreased iodide uptake, leading to a decreased synthesis of thyroid hormones which are essential for correct pre- and postnatal brain development. Accordingly, the AOP-Wiki database reports strong evidence linking NIS inhibition to adverse neurodevelopmental effects in offspring.^12^ NIS belongs to the solute carrier family 5 and shows a LeuT-fold consisting of 13 transmembrane helices. Although NIS has a high affinity for iodide and can discriminate between iodide and chloride, it also translocates other anions, such as perchlorate, pertechnetate, and perrhenate. Iodide plays a critical role, especially in embryonic and post embryonic development. Therefore, the transporter occurs not only in the thyroid gland but also in the lactating mammary gland, ensuring the supply of iodide for newborns. Mutations in NIS may lead to congenital hypothyroidism which, when not treated immediately after birth, leads to impaired growth and cognitive deficiency.^13,14^

Several ligand-based models have been already developed to predict the MIE NIS inhibition. These models rely on molecular similarity of ligands, typically based on descriptors of molecular structure, pharmacophore features, or properties.^15^ For instance, Gadaleta et al.^2^ created a screening method for predicting (developmental) neurotoxicity that integrates multiple quantitative structure–activity relationship (QSAR) models for several MIEs, including the inhibition of NIS. Balanced RF models were trained on extended fingerprints (Daylight Chemical Information Systems, Inc., 2019) which were calculated from ChEMBL^16^ data. For model building, 56 active, and 3371 inactive compounds were used. The inactive set mainly consisted of decoys, which were not directly tested against this target but were instead sourced from other end points. This approach was selected because the original ChEMBL data set was heavily skewed toward active compounds. The model achieved a balanced accuracy (BA) of 0.98, a Matthews correlation coefficient (MCC) of 0.96 and an area under the receiver operating characteristics curve (ROC AUC) of 1.0 on the test set. The authors stated that the extremely good performance may be caused by the high structural homogeneity of the actives set, while the inactives were structurally heterogeneous. Strengths of this approach include the high apparent performance and the integration of the model into a broader screening framework, however the reliance on decoy inactives and the structural homogeneity of the actives limit the generalizability of the NIS model. Garcia de Lomana et al.^17^ created a battery of machine learning (ML) models for the prediction of several MIEs leading to the perturbation of thyroid hormone homeostasis. For the development of the NIS inhibition model, 55 active (31 after applying cytotoxicity filtering) and 747 inactive compounds were retrieved from the ToxCast database.^18^ Based on molecular fingerprints and physiochemical descriptors, five ML algorithms-logistic regression, RF, XGB, SVM, and neural networks, along with three data balancing approaches, were employed to predict individual MIEs. Subsequently, multitask neural network models were developed to combine these MIEs. For NIS, the best performance was achieved with logistic regression in combination with oversampling, showing an MCC of 0.41, BA of 0.68, and ROC AUC of 0.86. Advantages of this approach include the use of experimentally derived data, comparison of multiple algorithms and data-balancing strategies, realistic performance metrics for NIS inhibition, and the implementation into a broader thyroid toxicity modeling framework. However, the number of experimentally tested NIS inhibitors for model training remains limited. Dracheva et al.^19^ also combined models of several MIEs to predict thyroid hormone system disruptors. For the development of the NIS inhibition model, 39 active compounds and 164 inactive compounds were obtained from Wang et al.^20^ A conformal prediction framework with underlying RF on RDKit chemical descriptors was explored. While the strength of this approach is the combination of multiple targets for predicting TH disruption, its focus on NIS inhibition is limited and based on a relatively small number of compounds, which may restrict the generalizability of the resulting predictions. Additionally, QSAR models for NIS inhibition were developed by the Technical University of Denmark (DTU) and incorporated into the Danish (Q)­SAR database, which is supported by Leadscope Enterprise software (Research Group for Chemical Risk Assessment and GMO, National Food Institute, Technical University of Denmark, http://qsar.food.dtu.dk). At the time of the investigations, no publicly available documentation on the training set composition or predictive performance for the NIS inhibition models could be found on the website, which limits an objective assessment of their applicability. In summary, existing approaches for NIS inhibition prediction are exclusively ligand-based and rely on a limited number of experimentally tested actives. Their applicability is constrained by the composition of the data sets and by limited interpretability at the protein–ligand interaction level.

To the best of our knowledge, no structure-based approaches have been conducted yet. Among structure-based methods, molecular docking is commonly used for predicting interactions and poses between a small molecule at the binding site of a protein, as well as for estimating their affinity scores. These scores can be utilized for ranking and prioritizing the compounds.^21^ To address limitations in existing approaches, the scope of the subsequent study was to develop computational models of the MIE “Inhibition of NIS” relating to the DNT-associated AOP 134 ^12^ published in the AOP-Wiki, integrating molecular docking with ML methods. Docking experiments complement ligand-based approaches by introducing structural information from the protein binding pocket. Additionally, the resulting docking poses can provide insights into the binding modes of potential inhibitors, offering mechanistic explanations that can enhance interpretability of the predictions.

The created model allows initial screening of chemicals to flag potential neurodevelopmental disruptors and can be integrated into the ASPA workflow, a broad-purpose, transparent and reproducible risk assessment framework developed by the ASPIS cluster of European Horizon 2020 research projects. ASPA is part of next-generation risk assessment efforts and aims to reduce animal experimentation, while guiding scientists and regulators from problem formulation to risk characterization. Within the hazard pillar, screening tools can be used for identifying potential toxicants with high sensitivity, while later stages employ follow-up experiments to increase specificity.^22^

## Materials and Methods

### Data Set and Overall Workflow

The data set used for modeling, consisted of approximately 1800 environmental chemicals screened by Wang et al.^23^ for NIS inhibition using a radioactive iodide uptake (RAIU) assay. The authors reported that the chemical library was designed to be structurally diverse and to cover known endocrine-active chemicals, pharmacologically active compounds, and industrial-use chemicals, such as agrochemicals. Compounds demonstrating >20% RAIU inhibition were further tested at multiple concentrations and classified as HIT1 actives if significant inhibition was observed. A cytotoxicity filter was applied to the 400 resulting HIT1 actives. Out of them, 112 compounds were found to produce noncytotoxic RAIU inhibition and were therefore classified as HIT2 actives.^23^

Molecular docking and ML models were developed independently using the noncytotoxic data set, while cytotoxic actives were set aside and predicted later. The outputs from both approaches were subsequently combined into one consensus scoring framework to improve predictive performance. A schematic illustration of the data set composition and data partitioning is provided in the Supporting Information (Figure S1).

### Molecular Docking

At the time of the investigations, one inward open and two inward occluded structures of NIS (PDB-ID: 7UUY, 7UV0, 7UUZ)^14^ determined by cryo-electron microscopy were available on the Protein Data Bank (PDB).^24^ However, since the inhibitors are expected to bind to the outward open conformation, homology models of rat NIS published by Chakrabarti et al.^25^ were used for the docking experiments. The authors modeled the inward open conformation using the bacterial sodium/galactose symporter vSGLT (PDB ID: 2XQ2, 2.73 Å resolution) and the outward open structure using the sodium-coupled sialic acid symporter (PDB ID: 5NVA, 2.26 Å resolution) as templates, which share 32% and 25% sequence identity with NIS, respectively. To explore the conformational transition between outward and inward states, they performed well-tempered metadynamics simulations of the ion-bound transporter (three replicas, 2.1 μs total) using an SVM-based collective variable approach with two inter-residue distance features (MET 23 CA-SER 474 CA distance and LYS 86 CA-VAL 412 CA distance). No protein backbone restraints were applied during the production simulations. The root mean square deviation between the inward and the outward open structures was 2.66 Å. The resulting trajectories along with details about the hyperplane definition and collective variable selection, were made publicly available and can be accessed via the original publication.^25^ The trajectories exhibited frequent transitions between metastable states without full convergence into one single stable conformation. Hence, 16 random frames sampled along the transition pathway were selected for docking. One of the outward directed protein structures obtained from the MD trajectory was compared to the inward open structure from PDB (PDB-ID: 7UUY) in Figure S2. Additionally, PyMOL 2.4.1 was used for calculating the molecular surface area and the solvent accessible surface area (SASA) of the binding pocket for each frame of the trajectory. The iodide and sodium binding site (see Figure 1) was selected as the binding pocket for inhibitors, since competitive binding of inhibitors has been observed at this site in homologous transporters, such as in the sodium-glucose cotransporter 2 (SGLT-2). Ravera et al.^14^ identified that the iodide binding site is highly conserved and exhibits a partially positive electrostatic surface potential. The site consists of Q72, Q94 and lipophilic residues. The sodium cations are coordinated by Q72, S416, F417, S69, and Y144, establishing the two sodium binding sites. During the transport cycle, the first Na^+^ binds to the Na2 site, causing a conformational change of NIS and therefore increasing the affinity for Na^+^ at the Na1 site and for I^–^.^14^ For this reason, docking was performed into structures without ions, with one Na^+^, with both Na^+^ ions, and with all the ions.

**Figure 1:**
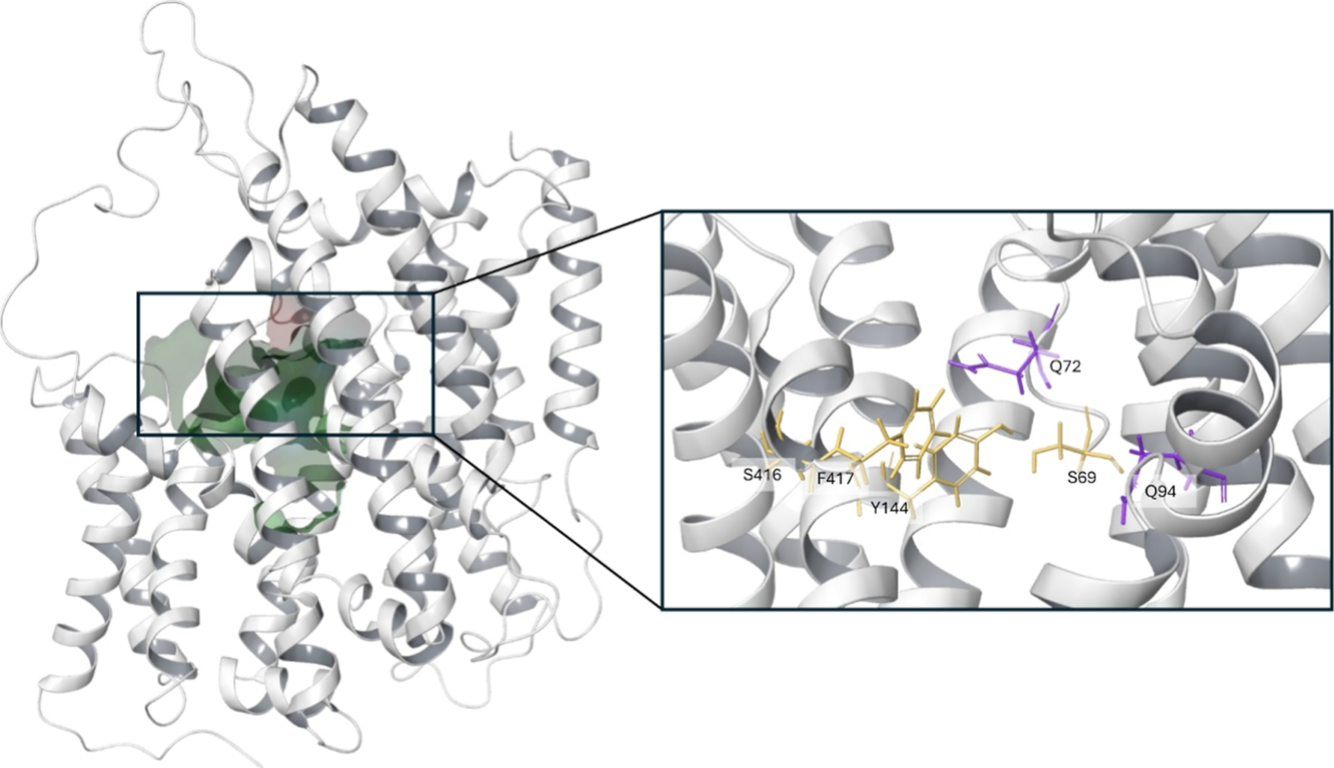
Protein conformation selected for docking studies. On the left, the surface of the binding pocket is color-coded according to residue properties: green for hydrophobic, red for negatively charged and blue for polar uncharged. The pocket is predominantly hydrophobic. On the right, residues important for iodide binding are highlighted in purple and those involved in sodium coordination are shown in yellow.

Since the compounds used in the docking experiments were tested for inhibition of human NIS while docking was performed into rat models, the sequences were compared beforehand utilizing the basic local alignment search tool (BLAST)^26^ with standard settings. The overall sequence identity between human and rat NIS is 83.82%. Within the binding pocket, no differences were identified, except for a substitution of threonine (rThr517) in the rat sequence with serine (hSer522) in the human sequence.

Glide was selected for the docking experiments, since the virtual screening workflow is well-established within the applied research framework, ensuring methodological consistency and reproducibility. Additionally, Glide has been extensively benchmarked and demonstrated high accuracy compared to other docking engines.^27–30^ The proteins were prepared with Protein Preparation Wizard^31^ (Schrödinger Release 2022-4: Protein Preparation Workflow; Epik, Schrödinger, LLC New York, NY, 2022; Impact, Schrödinger, LLC, New York, NY; Prime, Schrödinger, LLC, New York, NY, 2022.) and the ligands with LigPrep (Schrödinger Release 2022-4: LigPrep, Schrödinger, LLC, New York, NY, 2022.), both at pH 7.4 ± 0.4. The remaining settings were left at their default values. Glide^29,30^ (Schrödinger Release 2022-4: Glide, Schrödinger, LLC New York, NY, 2022.) standard precision (SP) docking was performed with the virtual screening workflow in Maestro (Schrödinger Release 2022-4: Maestro, Schrödinger, LLC, New York, NY, USA) while keeping default settings, except the grid size was set to 20 Å, 10 poses per compound state were generated, and after docking, all compounds were kept. The selected docking protocol allows for ligand flexibility, while keeping the protein structure rigid. The OPLS4 ^32^ force field was utilized for all preparation and docking tasks. Glide offers three different docking options-HTVS (high throughput virtual screening), SP (standard precision) and XP (extra precision), which differ primarily in computational speed and accuracy. While HTVS is optimized for speed, SP employs the same scoring function but performs more exhaustive conformational sampling with an approximately 10-fold increase in computational time. In contrast, XP, which uses an alternative scoring function along with more rigorous conformational sampling is computationally most expensive.^33^ To balance accuracy and efficiency when screening a larger ligand set, the SP mode was selected for the present study.

Different conformers of the same compound were ranked according to the Emodel scoring function. Subsequently, the top ranked conformers were ranked separately using either the docking score or the GlideScore implemented in Maestro.^34^ No substantial differences were observed between the two ranking schemes. Hence, the docking score was used for subsequent analyses, since it includes Epik state penalties.^35^ The ability of the virtual screening workflow to assign better scores to active compounds was assessed by generating a receiver operating characteristic (ROC) curve and determining its area under the curve (AUC). For this purpose, the Enrichment Calculator integrated into the Schrödinger software package and Python were utilized. Additionally, a kernel density estimate (KDE) plot, created using the scipy.stats module and plotted with matplotlib in Python, was employed to visualize the estimated distribution of active and inactive compounds at their docking scores and to determine a threshold for classifying compounds as predicted actives or inactives.^36,37^ This allowed calculation of binary classification metrics, including MCC and BA. MCC ranges between −1 and 1 and accounts for both positive and negative instances while remaining robust to class imbalance.^38^ BA is the average of the accuracy obtained on the active and inactive classes, also suitable on imbalanced data sets, and ranges from 0 to 1.^39^

Based on these analyses, the protein model, which achieved the highest ROC AUC, was selected for further studies. No significant Ramachandran outliers or structural issues were observed in the binding pocket of the prepared protein. The overall Ramachandran *Z*-score of −1.9 and χ1/χ2 angle correlation *Z*-score of −0.58 indicate that both backbone conformations and side chain rotamers fall within expected ranges for well-refined structures.^40^

### Machine Learning

The ML studies were conducted in Python 3.10. The models were trained and tested utilizing the same set of molecules employed in the docking experiments, with cytotoxic ones excluded prior to model development (Figure S1), to avoid training on compounds with inconclusive in vitro activity labels.^23^ First, a standardization workflow was applied to ensure data consistency, which involved exclusion of inorganic compounds, neutralization of charges, and salt stripping while retaining the largest fragment of each compound, resulting in a final data set of 1412 compounds. Stereochemistry was removed based on preliminary tests comparing model performance with and without stereochemistry information (see Table S3). Continuous and data-driven molecular descriptors (CDDDs)^41^ and extended-connectivity fingerprints with bond diameter 4 (ECFP4) were generated for each compound. A random, stratified split was applied, dividing the data into an 80% training set and a 20% test set. The models were designed as binary classifiers, predicting activity labels (active = 1, inactive = 0).

### Baseline Models

Initially, training was performed with RF, SVM, and XGB algorithms using nested 9-fold cross validation (CV). Hyperparameter tuning was conducted in five inner folds with following parameters:

- RF: n_estimators: 50, 100, 200, max_depth: None, 10, 20
- SVM: C: 0.1, 1, 10, kernel: linear, rbf
- XGB: n_estimators: 50, 100, 200, max_depth: 3, 5, 7

Subsequently, the nine outer folds were trained using the best performing parameters. The most common parameters across the nine outer folds (listed in the Supporting Information: “Selected parameters in machine learning”) were selected for retraining of the whole training set.

Finally, the resulting model for each algorithm was applied to the test set.

### Nested Cross Validation with Undersampling and Hyperparameter Tuning

In order to address the class imbalance between actives and inactives (∼1:12.5), undersampled subsets of the data were used for model training. The sampling workflow is illustrated in Figure 2. Undersampling was performed within the outer loops. In this process, the inactives in each training fold were divided into nine subsets. Each of them was combined with the full set of actives present in the corresponding training fold. This resulted in nine distinct training sets per outer fold. The corresponding validation folds remained imbalanced, preserving the class distribution of the full data set, which allows a more accurate estimate of how the models would perform on unseen data.

**Figure 2:**
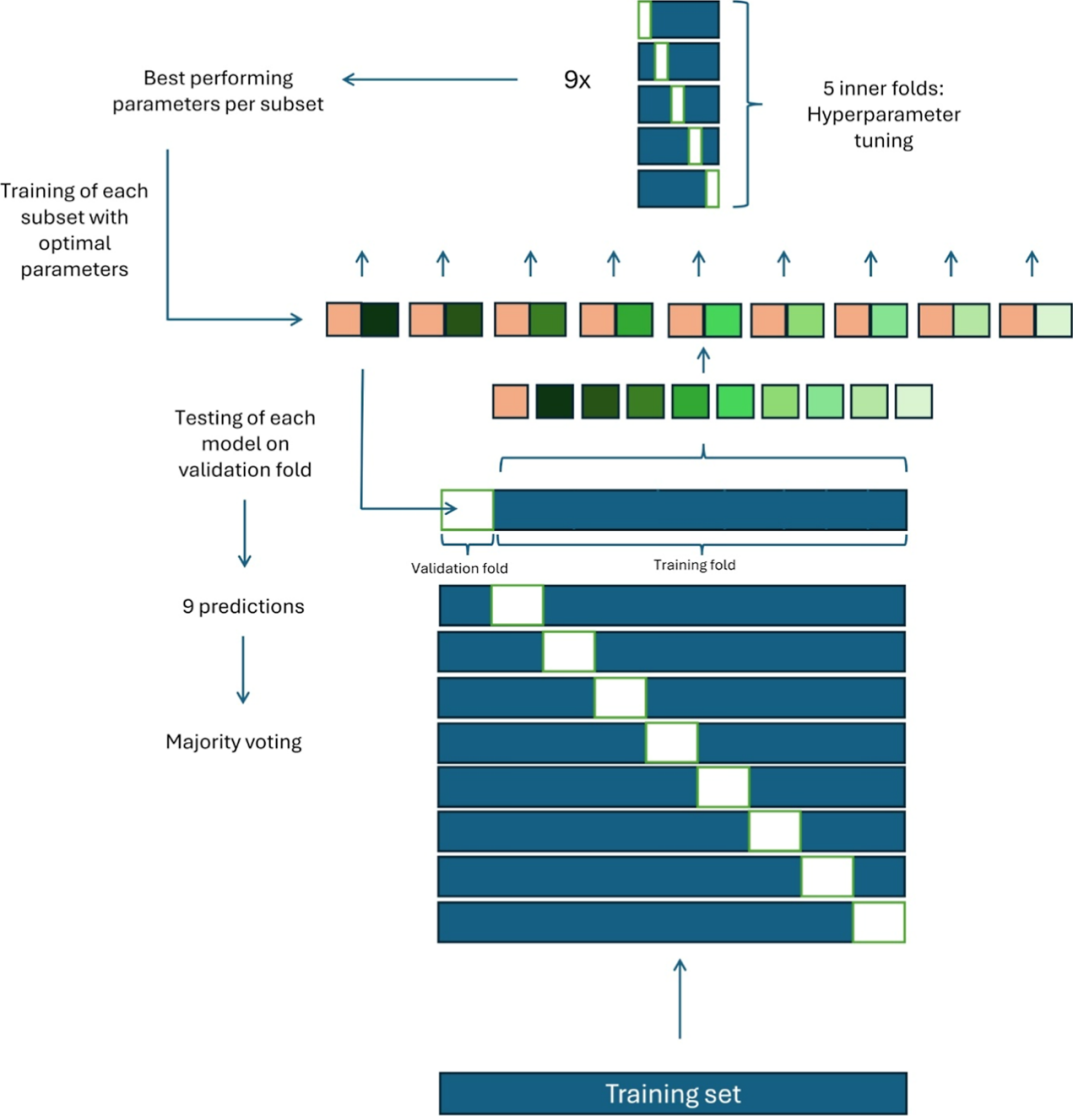
Visual representation of the undersampling workflow within nested cross validation. The blue bar represents the training set which was split into training and validation fold (in white) nine times, with each validation fold comprising a distinct set of compounds. Each training fold was divided into active compounds (in red) and inactive compounds (in green). The inactives were further split into nine subsets (represented by different shades of green). Each subset was combined with the full set of actives, resulting in nine approximately balanced training sets. These sets were each passed into the inner loop for hyperparameter optimization, which was conducted using 5-fold cross validation. The validation fold (in white) shifted across iterations in the same manner as for the outer fold. The nine sets of hyperparameters exhibiting the best performance were then used to train models on the full outer training fold. These models were tested on the original, imbalanced validation fold, producing nine predictions per compound. A majority vote across the predictions determined a final classification label which was used for the performance metric calculation. This procedure was repeated for each outer fold. Finally, the mean of performance metrics across all folds was calculated.

Hyperparameter tuning was conducted in the same manner as for the baseline models. Since each outer fold generated nine binary predictions, majority voting was used to derive a single final prediction per fold. Ultimately, the most common parameters across the nine outer folds were selected (listed in the Supporting Information: “Selected parameters in machine learning”) and used for retraining of the training set, which was again split into nine undersampled subsets. Therefore, retraining resulted in nine distinct models per ML algorithm.

Each of the nine models was applied to the test set, leading to nine predictions per compound. For these, the majority vote was used to calculate performance metrics, such as MCC and BA, while the sum of votes was applied to generate the ROC curve.

### Cross Validation with Undersampling and Default Parameters

In order to evaluate if hyperparameter tuning led to an improvement of the model quality, the training set was trained on default parameters with 9-fold CV. The inactives from each outer fold were again split into nine subsets, each of which was combined with the active set. Each of the nine models was used to predict the activities of the imbalanced validation fold. The majority vote for each fold was taken to calculate the performance metrics. Finally, the undersampling approach was applied to the whole data set. The nine sets were trained on default parameters, and the nine resulting models were applied on the test set. The majority votes and the sum of votes were used for further performance evaluations as described previously.

### Consensus Scoring

To integrate the docking and ML approaches, consensus scores were computed. Docking scores were directly obtained from the docking output, whereas the ML models did not produce explicit scores. Instead, the sum of positive predictions across nine models generated via the undersampling approach was considered as a score. Both the docking scores and the prediction sums from the test set were normalized to a range between zero and one, using min–max normalization. Since more negative docking scores indicate stronger binding affinity, the docking scores were negated before normalization. Normalized scores *x*_norm_ were calculated as

$$x_{norm}=\frac{x-x_{\min}}{x_{mix}-x_{\min}}$$

where *x* is the raw score. Fixed minimum and maximum values were derived from the training set. For ML predictions *x*_min_ = 0 and *x*_max_ = 9. For negated docking scores *x*_min_ = −2.80 and *x*_max_ = 12.073.

Finally, the normalized scores were combined by calculating their sum. The resulting values were then used to generate ROC curves and KDE plots, from which thresholds for the discrimination between actives and inactives were determined. Ultimately, the consensus scoring technique was applied for predicting the cytotoxic actives, that were excluded from model testing and training. Additionally, compounds from ChEMBL tested for NIS inhibition were used as an external test set.

### Data Visualization and Molecular Similarity Analysis

In order to visualize the chemical space, identify structural similarities between compounds and evaluate the applicability domain, MolCompass^42^ was utilized. This tool employs a pretrained parametric t-distributed stochastic neighbor embedding (t-SNE) model. ECFP fingerprints were calculated for the standardized compounds, and dimensionality reduction was performed using a feed-forward artificial neural network, yielding two output values per compound (X- and Y-coordinates), allowing further analysis in a two-dimensional space.

To assess similarities between the compounds used for model training and evaluation and the predicted compounds, Tanimoto coefficients were calculated based on ECFP4, since previous studies have demonstrated their high performance in chemical structure comparison. The Tanimoto coefficient is commonly used for comparing molecular similarities by quantifying the overlap between fingerprints. It provides a numerical value between 0 and 1, where 0 indicates no similarity and 1 indicates identical molecular structures.^43,44^ ECFP4 were generated using RDKit. After calculating the Tanimoto coefficients, the five nearest neighbors from the model-building compounds were identified for each predicted compound. Subsequently, the mean Tanimoto similarity for the nearest neighbor of each query compound was calculated to evaluate the overall similarity between the predicted and the model-building compounds.

## Results and Discussion

### Molecular Docking

Within this study, ligand preparation resulted in 1751 compounds which were docked into 16 frames of an MD trajectory of NIS,^25^ simulating the transition between the outward and the inward open state in the presence of bound ions. Docking was performed for distinct constellations of ions in each frame. For each frame, ROC curves were generated to assess the ability of docking scores to rank actives above inactives. The ROC AUCs for the enrichment of HIT1 actives ranged from 0.61 to 0.70. Molecular surface areas and SASAs of the binding pockets were calculated for each frame. However, no correlation with enrichment performance could be observed. Consequently, frame selection for subsequent analyses was based on the ROC AUC values obtained against experimental data. A table reporting the molecular surface areas, SASAs, and the corresponding ROC values for the frames used for docking is provided in the Supporting Information (Table S1).

The highest ROC AUC was obtained for frame 249 without any ions in the binding pocket. The structure of this frame was therefore selected for subsequent studies. In this docking run, 1745 compounds were successfully docked. All 6 compounds that could not be placed in the binding pocket were classified as inactive in vitro. For the enrichment calculations, the undocked compounds were retained in the data set and assigned a docking score of 2.8, slightly worse than the worst docking score (2.78) in the output. This approach ensures that compounds without output poses are ranked below successfully docked compounds. The enrichment of HIT1 actives according to their docking scores resulted in a ROC AUC of 0.70. When enrichment was performed for HIT2 actives, defined as noncytotoxic inhibitors, as described in the methods ("Data Set and Overall Workflow") and Figure S1, and cytotoxic HIT1 actives were treated as inactive, the ROC AUC decreased to 0.63. This trend was consistently observed across all analyzed frames (see Table S1). The drop in the performance prompted us to hypothesize that a part of the cytotoxic actives may inhibit the transporter through specific binding. Hence, a third enrichment analysis was conducted, excluding the cytotoxic HIT1 actives entirely from the data set. This adjustment partially restored performance (ROC AUC 0.67), consistent with the idea that these compounds contribute to some signal in docking. ROC curves illustrating the docking results for frame 249 with different ion constellations are shown in Figure S3.

When applying the estimated distributions of compounds against their docking scores, a similar change in performance was observed. The actives and the inactives curve showed the largest separation when treating the HIT1 actives as active (Figure S4a). At docking score −6.52 an MCC of 0.29 and a BA of 0.66 were achieved. When only the noncytotoxic actives (HIT2 actives) were treated as actives, the actives curve shifted into the direction of the inactives curve. In this case, the optimal discrimination of inactives and actives dropped to an MCC of 0.12 and a BA of 0.62. When completely excluding the cytotoxic actives from the data set, a slight improvement, with an MCC 0.16 and a BA of 0.65 was observed. For a full comparison including all three KDE plots, see Figure S4.

Taken together, the enrichment and distribution analyses indicated that when cytotoxic actives were included as active compounds, the best apparent separation between actives and inactives was achieved. This suggests that at least some cytotoxic compounds may interact with NIS through specific binding, contributing to the observed activity.

### Machine Learning

The cytotoxic compounds were excluded from the data set used for ML model training and testing. This decision was made for two reasons: first, to avoid including compounds with inconclusive biological behavior in the training data, and second, to validate the hypothesis derived from docking results by later predicting the activity of these compounds using the trained models.

### Baseline Models

Baseline models were trained using nested 9-fold CV with hyperparameter tuning. The MCC (measures the overall quality of binary classifications) and BA (average sensitivity and specificity) values for the training and test sets across all models and descriptor combinations are summarized in Table S2. Due to the strong class imbalance, all models, except for the SVM model trained on CDDDs, exhibited a poor performance. For this SVM model, the MCC was acceptable in both the training and test sets, but the BA remained relatively low. This can be explained by the high specificity of 0.98 alongside a low sensitivity of 0.24 on the test set, indicating poor performance in detecting active compounds. A tendency to overpredict inactive compounds can likely be attributed to the class imbalance in the training data. This is particularly problematic in the context of toxicology, where failing to identify active, potentially toxic compounds should be avoided. To address this issue, an undersampling approach was applied in the subsequent steps.

Interestingly, including stereochemical information in CDDDs did not improve model performance (see Table S3 for baseline model results). Although descriptor differences between stereoisomers were detectable (e.g., via Tanimoto similarity), they were relatively small compared to differences captured by other features. Consequently, the model was likely confused by nearly identical descriptors for stereoisomers with partly contrasting activity labels in the training set, which reduced overall performance. Thus, all herein described ML studies were performed with the data set excluding stereochemistry.

### Nested CV with Undersampling and Hyperparameter Tuning

To address class imbalance, undersampling was performed within each outer training fold. Each compound received nine predictions from the models trained on the undersampled subsets, which were combined via majority voting to generate a single final prediction. Performance metrics were averaged across folds (Table 1). Compared to the baseline models, the performance across different algorithms showed less variability, indicating improved robustness of the models after undersampling. Overall, models trained on CDDDs outperformed those trained on ECFP4 fingerprints. For instance, the RF model trained on CDDD representations achieved a specificity of 0.81 and sensitivity of 0.57 on the test set. Compared to the baseline model, its ability to identify active compounds increased, while still maintaining reasonably high specificity.

**Table 1:** Performance Metrics on the Test and Training Set across Different ML Algorithms and Molecular Representations Resulting from Majority Voting, Based on Training on Undersampled Subsets with Hyperparameter Tuning^a^

		ECFP4			CDDD		
		RF	SVM	XGB	RF	SVM	XGB
training	MCC	0.23 ± 0.08	0.22 ± 0.11	0.21 ± 0.10	0.36 ± 0.11	0.40 ± 0.12	0.35 ± 0.14
	BA	0.66 ± 0.05	0.67 ± 0.90	0.67 ± 0.07	0.78 ± 0.08	0.81 ± 0.08	0.77 ± 0.10
test	MCC	0.23	0.19	0.17	0.24	0.23	0.25
	BA	0.67	0.65	0.64	0.69	0.68	0.69

a The training performance is reported as the mean and standard deviation of MCC and BA across the nine outer cross-validation folds.

The nine different predictions served multiple purposes. They were not only used for majority class voting, but their sum was also treated as a score. This score enabled the creation of ROC curves (Figure 3b) for the test set for direct comparison with docking results. Additionally, scores from CDDD-based models were incorporated into consensus scoring alongside docking scores in later stages.

**Figure 3:**
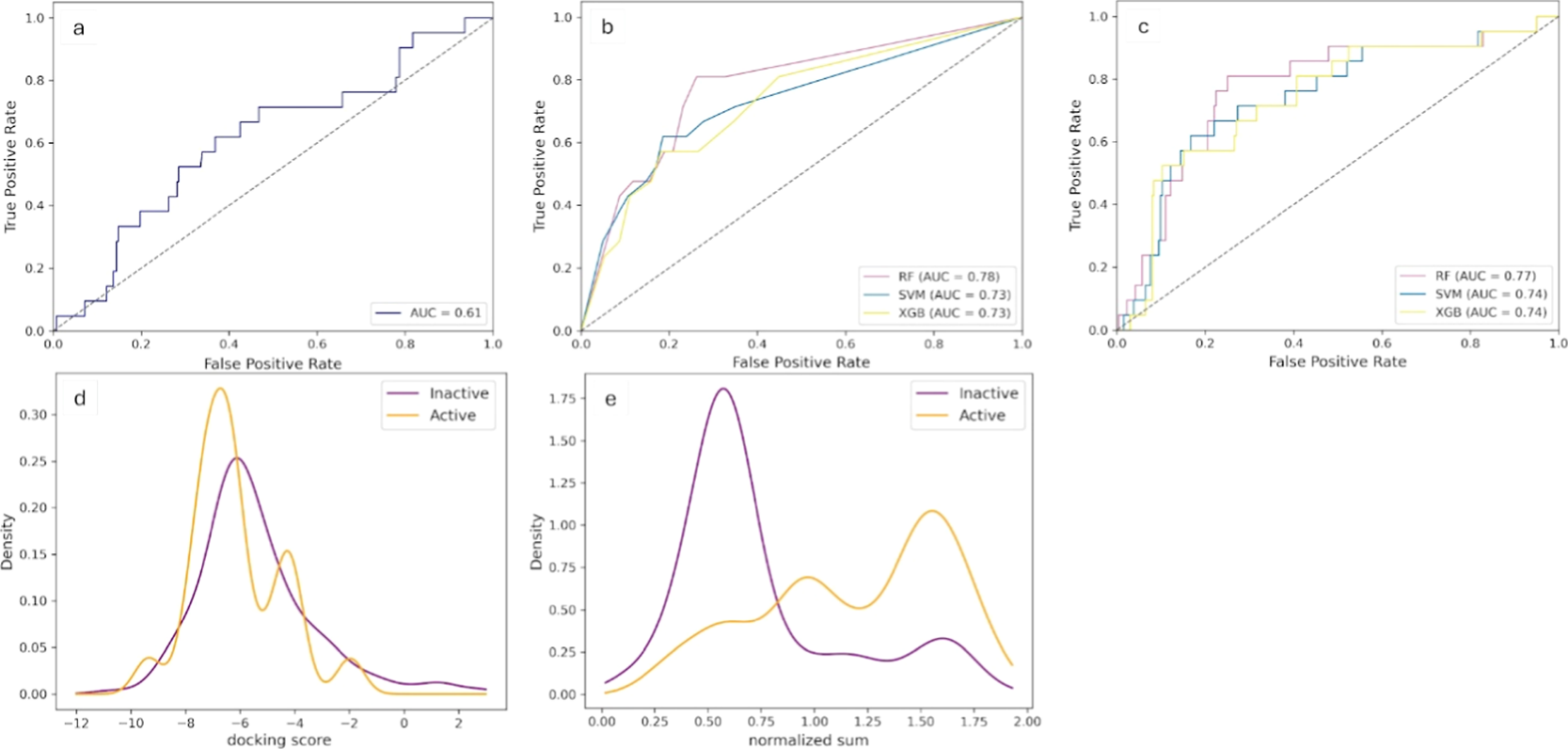
Enrichment analysis plots. (a–c) Receiver operating characteristic (ROC) plots. The true positive rate is shown on the *y*-axis, and the false positive rate on the *x*-axis. Enrichment refers to the proportion of active compounds identified at different thresholds. (a) ROC plot for docking-based virtual screening applied to the test set compounds from the machine learning data set. (b) ROC plot for predictions of 284 test set compounds using different ML algorithms trained on CDDDs, based on the sum of active votes. The pink curve (RF) corresponds to the random forest model, the blue curve (SVM) represents the results from the support vector machine model, and the yellow curve (XGB) corresponds to the extreme gradient boosting model. (c) ROC plot for test set predictions using a combination of docking scores and the sum of votes of different ML algorithms after hyperparameter optimization trained on CDDDs. The pink curve corresponds to the RF combination, the yellow curve to the XGB combination, and the blue curve to the SVM combination. (d) Density distributions of docking scores (Glide) for actives and inactives in the test set used for machine learning. The orange function represents 21 tested actives, whereas the purple function represents the 263 tested inactives. A slight separation of the active and the inactive curves is observed, suggesting that compounds can be distinguished as actives and inactives by docking scores to some extent. (e) Density distributions of actives and inactives at different consensus scores obtained from combining docking scores with the sum of active votes from the random forest model trained on CDDDs after hyperparameter optimization. The separation between the active and inactive curves improved substantially compared to docking alone. KDE plots for the other algorithms and parameters are shown in the Supporting Information (Figure S5 and S6).

### Cross Validation and Default Parameters

In order to evaluate the impact of hyperparameter tuning, the same procedure was repeated with a simple 9-fold CV using the default parameters. The corresponding performance metrices are listed in Table S4. Statistical comparison using the Wilcoxon signed-rank test revealed no significant differences in the performance of models with default and tuned parameters (MCC: *p* = 0.94; BA: *p* = 0.84 on the test set). This finding aligns with the recently published article from Tetko et al.,^45^ in which the overall benefit of hyperparameter optimization was questioned.

### Consensus Scoring

In order to combine the docking-based and the CDDD-based ML models, a consensus score was generated for the compounds in the test set. Subsequently, enrichment analyses and performance metrics were calculated as in the previous experiments. For a more accurate comparison, the docking results were reanalyzed, focusing exclusively on the test set used in the ML experiments (ROC and KDE plots are provided in Figure 3a,d). Compared to the docking results for the data set without cytotoxic compounds, the performance remained nearly unchanged at the same docking score threshold applied for the whole set (−6.52), achieving a ROC AUC of 0.61, an MCC of 0.14, and a BA of 0.62.

The consensus scores demonstrated a substantial improvement in distinguishing between active and inactive compounds. The best performance was achieved by combining docking scores with the sum of active votes from the random forest models, resulting in a ROC AUC of 0.77, an MCC of 0.32, and a BA of 0.78 at a threshold of 0.82. This consensus model was therefore selected for subsequent predictions and analyses. MCCs and BAs for all types of consensus scoring are listed in Table 2. Corresponding plots are presented in Figure 3c,e.

**Table 2:** Performance Metrics for the Test Set across Consensus Scores That Combine Docking Scores with the Sum of Active Votes from Different ML Algorithms Trained on CDDDs

	ML algorithm	hyperparameters	MCC	BA	ROC AUC
docking score +	RF	default	0.32	0.78	0.77
		tuned	0.32	0.78	0.77
	SVM	default	0.31	0.72	0.75
		tuned	0.30	0.73	0.74
	XGB	default	0.31	0.72	0.72
		tuned	0.32	0.71	0.74

Compared to previous approaches, the presented method specifically targeted NIS inhibition, whereas most other published models addressed broader end points such as TH disruption or DNT. Additionally, our study employed a larger and more representative data set. For example, Gadaleta et al.^2^ trained their model on CHEMBL14 data, which included 56 highly homogeneous actives and 3371 inactives, the majority of which were decoys. While their model reported a substantially higher performance (BA = 0.98, MCC = 0.96, ROC AUC = 1.0) than the model developed in the present study, this may be attributed to the high homogeneity of their actives set. Another weakness is the large set of decoys that was not experimentally tested, which can increase the risk of false negatives. In contrast, our training set consists of only experimentally tested compounds, and the actives cover a broader chemical space, enhancing the generalizability. In another instance Garcia de Lomana et al.^17^ reported more realistic performance metrics for NIS inhibition prediction, with BA = 0.68 (±0.06), MCC = 0.41 (±0.16) and ROC AUC = 0.86 (±0.08) for their best-performing model in 10-fold CV. A strength of this study is the use of experimentally derived data and a systematic comparison of multiple ML algorithms. However, the authors noted that the high standard deviation may indicate overfitting, which they attributed to the low number of actives (31) relative to 747 inactives in their training set. Our models showed comparable performance metrics and similarly high CV standard deviations in ML (Table 1), but were trained on a larger data set, consisting of 1048 inactives and 84 actives. Notably, Garcia de Lomana et al.^17^ did not report test set performances for NIS inhibition, preventing a direct comparison of final model performance. In our study, the differences between CV and test set performance provide no indication of strong overfitting. Beyond ML, we further combined the predictions with docking scores, which introduces novelty and may help mitigate overfitting. Dracheva et al.^19^ used 39 active and 164 inactive compounds, of which 80% were allocated for training. A strength of their approach is the application of conformal prediction, which provides an estimation of prediction uncertainty. However, the relatively small data set and their focus on thyroid disruption rather than NIS inhibition specifically represent limitations with respect to target-specific model reliability. In contrast, the considerably larger number of compounds in our data set may provide greater reliability for predicting NIS inhibition.

Taken together, by leveraging a larger, more diverse, and experimentally verified data set and combining ML with docking predictions, our approach provides a novel and potentially more robust framework for predicting NIS inhibition compared to previous studies. The created consensus model can be used for early stage screening to flag potential NIS inhibitors, for instance as a part of the hazard pillar in the ASPA workflow. Although the precision is relatively low (0.20), indicating the presence of false positives, the high sensitivity (0.81) ensures that the majority of the actives are captured. Hence, the model can help to prioritize compounds for subsequent evaluation using more advanced computational methods (e.g., MM-GBSA or MD simulations) or in vitro testing, within the broader ASPA workflow for structured, animal-free risk assessment.

### Classification of Cytotoxic Actives

Of the 400 compounds classified as inhibitors of iodide transport into the cell during in vitro experiments, 288 were identified as cytotoxic. One aim of this study was to explore which of these compounds might also exhibit NIS inhibition through specific binding.

For the final models, the test and training set were combined and retrained using the tuned RF models on CDDD representations, repeated across nine undersampled splits. Predictions were derived through calculating the consensus score from docking scores and the sum of active votes by the ML models (ranging from 0 to 9). Based on prior KDE analyses performed on the internal test set to identify a threshold for optimal discrimination between actives and inactives, as described in the Consensus Scoring sections of the Materials and Methods and Results and Discussion, compounds with consensus scores greater than 0.82 were classified as active.

Compounds lacking either docking score, an ML prediction or both, were inspected in more detail. In one instance, values were missing due to salt stripping and duplicate removal during ligand preparation before docking. The smaller fragments of salts were removed and if the larger fragments were identical, one was discarded to eliminate the duplicate. Consequently, the missing values for the salts were replaced with those from the larger fragment that was retained during ligand preparation. In another instance, stereochemistry was removed during standardization before ML, which resulted in duplicates that were subsequently removed. This led to cases where docking scores were available, but ML predictions were missing. To address this, ML predictions from the corresponding stereoisomers with available values were used to replace the missing data. As a result, the consensus scores indirectly incorporated stereochemistry information through the inclusion of the docking score. Lastly, compounds containing tin or zinc were excluded in ligand preparation for docking. Zinc pyrithione was removed prior to both docking and ML. Consequently, no consensus scores could be predicted for these (marked as NaN).

In total, 217 compounds (∼75%) were predicted as active, 64 (∼22%) as inactive, and no predictions could be made for seven compounds. A table listing the compounds, their corresponding scores, and predictions is provided in the Supporting Information (Table S5). While the predictions are not fully reliable due to the limited predictive performance of the model, most actives within the applicability domain are likely captured. Careful examination of the predictions can increase confidence in selecting compounds for further testing. Flagged compounds can then be evaluated in cell-free systems, where cytotoxicity does not impact the results, such as in membrane vesicle models.^46,47^

### Chemical Space Analysis

To assess whether the cytotoxic compounds occupied a similar chemical space as the training compounds, they were initially plotted with MolCompass.^42^ No notable outliers were observed in this plot (Figure 4), suggesting that the predicted compounds are well-represented within the domain of the training data. The mean Tanimoto similarity between each compound in the predicted set and its nearest neighbor in the training set is 0.47, indicating a moderate level of structural similarity. Certain predicted compounds were located in regions of the plot, where the surrounding training compounds showed opposing activity labels. To contextualize these predictions, the five nearest neighbors (based on Tanimoto similarity) from the training set were calculated for each predicted compound and examined in more detail. A complete list of the five nearest neighbors for each predicted compound is provided in the Supporting Information ("Nearest neighbors of predicted cytotoxic compounds").

**Figure 4:**
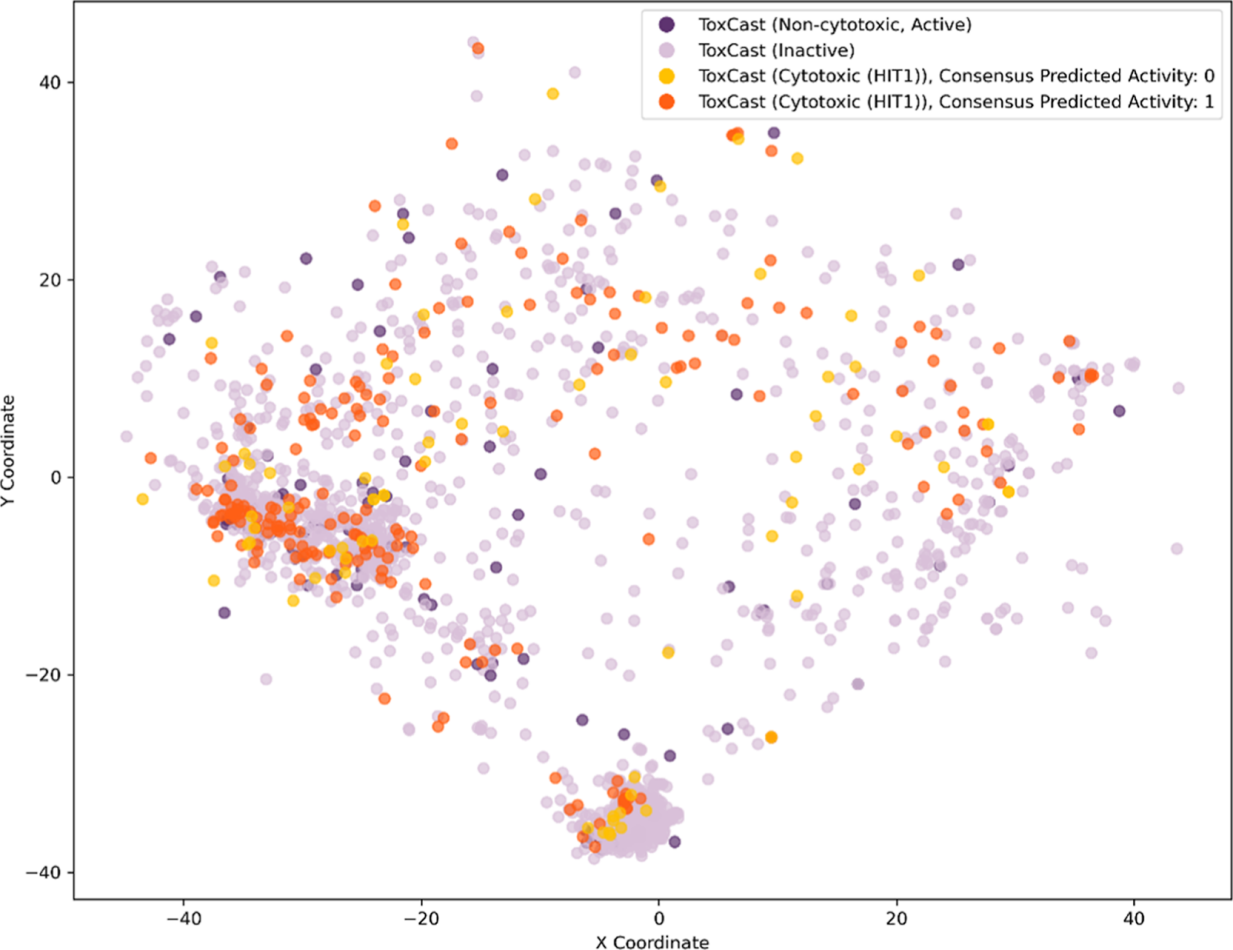
Visualization of predicted cytotoxic compounds (shaded in orange) and training data (shaded in purple), based on chemical space mapping using MolCompass. After calculating ECFP fingerprints, dimensionality reduction was performed using a pretrained parametric t-SNE model implemented via a feed forward artificial neural network, yielding two output values per compound (*X*- and *Y*-coordinates) for visualization in a two-dimensional space. Dark purple indicates measured actives, while light purple indicates measured inactives. Similarly, dark orange represents predicted actives, and yellow represents predicted inactives.

In general, the analysis revealed the following observations: compounds with higher lipophilicity were more likely to receive active classification labels, consistent with the lipophilic nature of the NIS binding pocket. For most ML predictions, plausible explanations could be identified based on chemical features and neighborhood context, consistent with the predicted compounds lying within the applicability domain. In contrast, docking results were less consistently interpretable. Examination of local chemical neighborhoods showed that only two negatively predicted compounds were located near experimentally active training compounds (see Supporting Information: “Additional example cases”), whereas several actively predicted compounds occurred in regions with inactive training compounds. This tendency reflects the higher sensitivity and lower specificity of the model. Such behavior is desirable in the context of initial screening in toxicology, where identifying as many potential actives as possible is prioritized over minimizing false positives.

A small subset of compounds from the predicted data set was selected to investigate and illustrate the model's practical application. Two cases that best demonstrate the interpretability and the limitations of the consensus approach are discussed below, while further examples covering additional prediction scenarios are provided in the Supporting Information (Section “Additional example cases”).

### C.I. Solvent Yellow 14

C.I. Solvent Yellow 14 is an example of a compound that is surrounded by compounds with high Tanimoto similarity with contrasting predictions. Nevertheless, its prediction is explainable both by docking and by the ML model. The compound received nine positive votes from the ML models and was also classified as active by docking. Its nearest neighbor in the training set, C.I. Acid Orange 7, has a Tanimoto similarity of 0.68 and was measured as inactive. The only structural difference between the two compounds is the presence of a para-substituted sulfonic acid group on the phenyl ring in C.I. Orange 7. Moreover, the remaining four nearest neighbors of C.I. Solvent 14 have Tanimoto similarities ranging from 0.44 to 0.48 but were all classified as inactive in vitro (see Table 3).

**Table 3:**
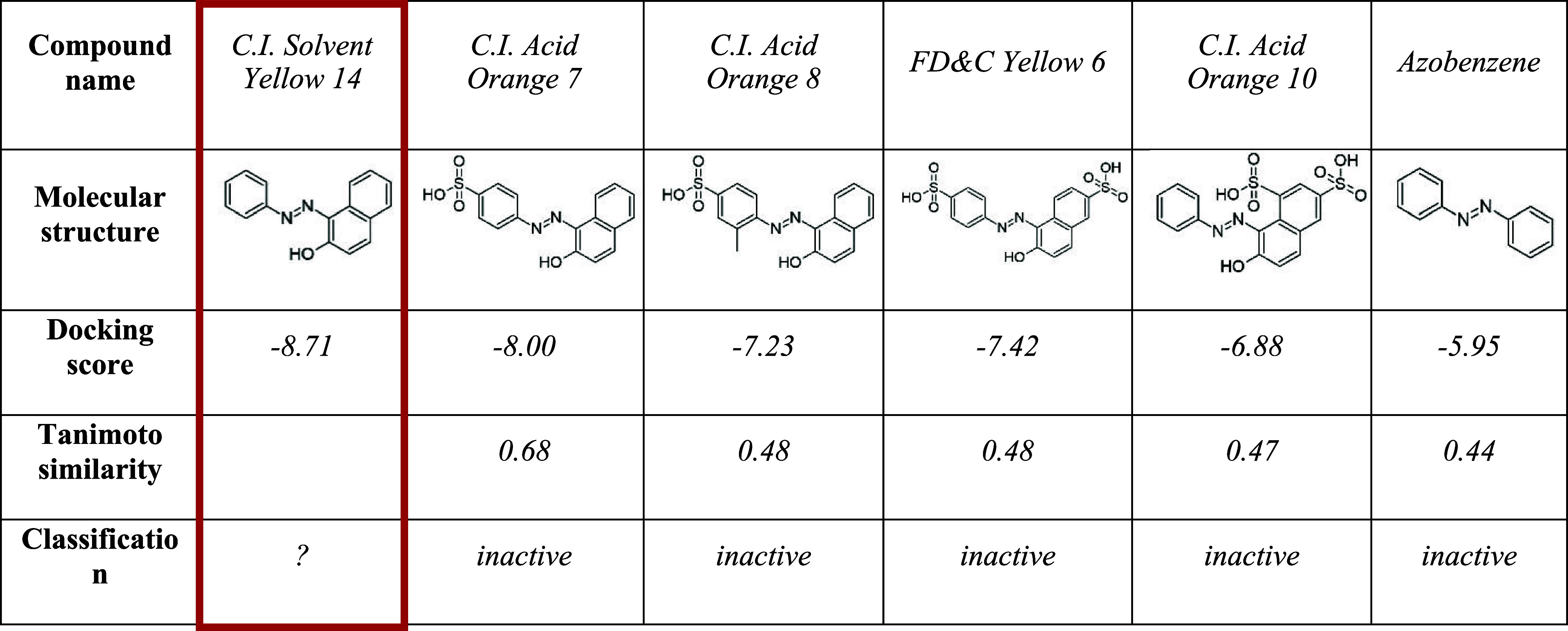
Five Nearest Neighbors of Predicted Compound^a^

a C.I. Solvent Yellow 14 (highlighted in red), along with their molecular structures and corresponding docking scores. C.I. Solvent Yellow 14 was predicted to be active by both the docking and ML models, as well as by the consensus model. The neighbors were classified as inactive in vitro.

To gain further insights, the docked poses from C.I. Solvent Yellow 14 and C.I. Acid Orange 7 were compared (Figure 5). The naphthyl moieties of both molecules adopt similar orientations, each forming π–π interactions with Phe417 and Tyr475, as well as a hydrogen bond to Gly250. The phenyl ring of C.I. Solvent Yellow 14 engages in an additional π–π interaction to Phe470, which does not occur with C.I. Acid Orange 7. A positional shift of the phenyl ring likely disrupts this interaction. This shift may be attributed to electrostatic repulsion from the negatively charged Glu79 shown in the upper right corner of the binding pocket in Figure 5.

**Figure 5:**
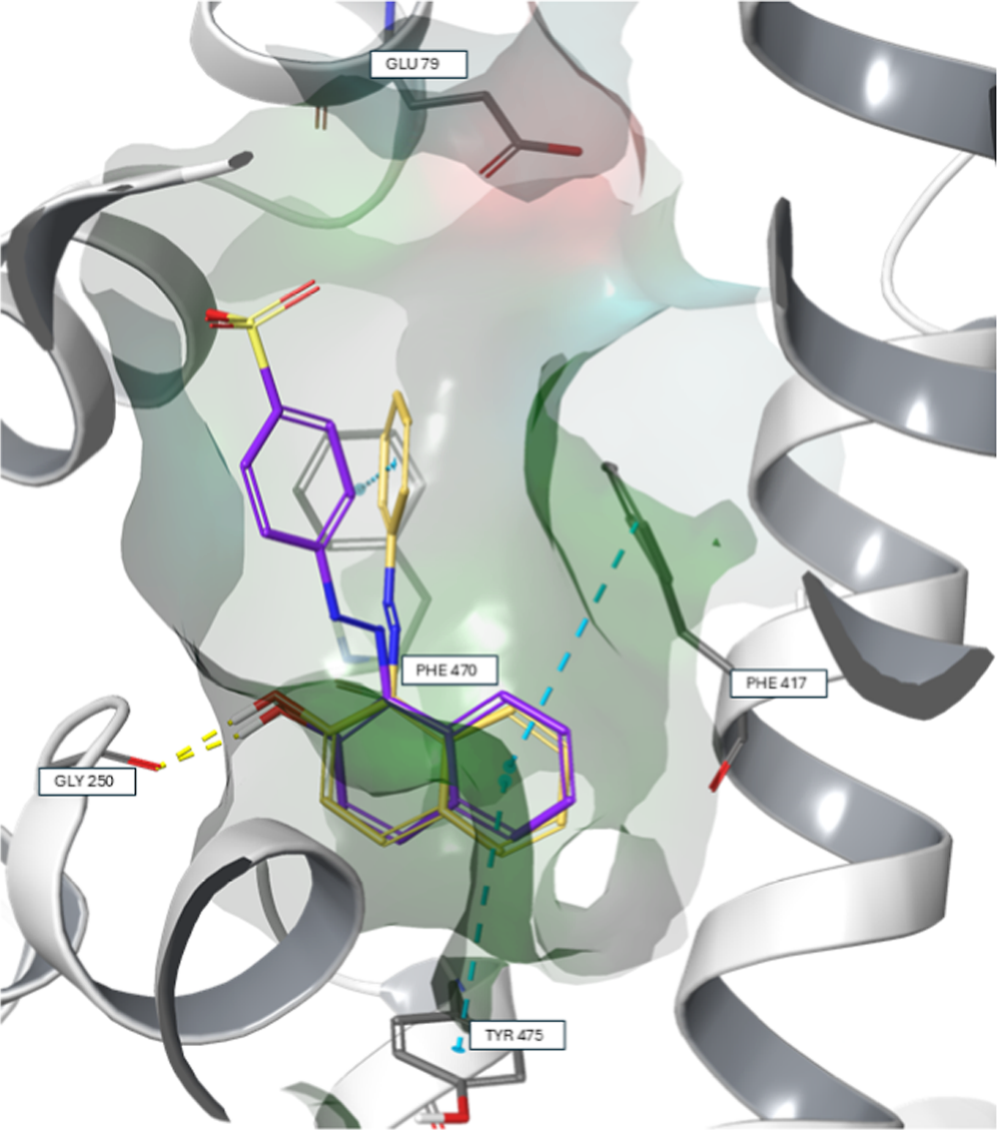
Docking poses of C.I. Solvent Yellow 14 (in yellow) and C.I. Acid Orange 7 (in purple). The protein surface is colored according to residue properties: green for hydrophobic, red for negatively charged, blue for polar uncharged, and gray for glycine residues. Yellow dashed lines indicate hydrogen bonds, and blue dashed lines represent π–π interactions.

When applying the previously determined docking score threshold of −6.52, all of the nearest neighbors, except for Azobenzene, were incorrectly classified as active by docking (see Table 3). Interestingly, the docking scores suggest that sulfonic acid groups negatively influence binding affinity. This effect may not only be explained through electrostatic repulsion from Glu79 but could also appear due to the predominantly lipophilic nature of the binding pocket, which likely disfavors highly polar or charged substituents. Notably, C.I. Solvent Yellow 14, which lacks sulfonic acid groups, received the most favorable docking score, aligning with its positive ML prediction.

### Conazoles

All conazoles from both the predicted and the training set were examined (Table 4). According to the docking score threshold of −6.52 four out of eight inactive training compounds were correctly classified as inactive, whereas all predicted compounds received a positive classification. Notably, the only experimentally confirmed active compound, Econazole nitrate, achieved the best docking score among the training compounds, indicating that the docking score partially reflects the activity trend. Among the predicted compounds nine out of 11 compounds were classified as active by the ML model. In contrast, the training set contains eight inactive and one active conazole. The only positively tested conazole, Econazole nitrate, contains an additional chlorine atom at the ortho position of the chlorophenyl ring, highlighting the potential influence of lipophilic substituents on activity.

**Table 4:**
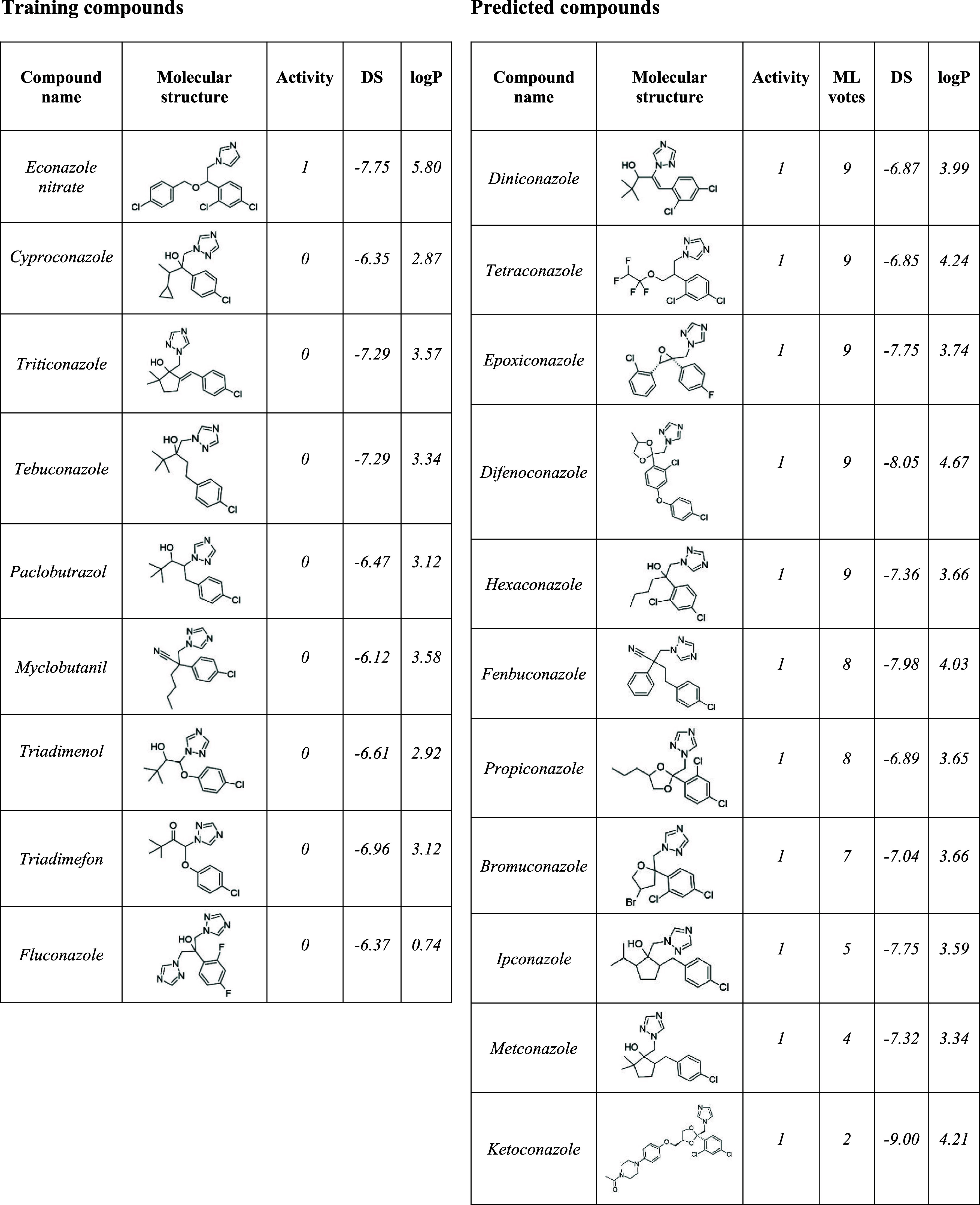
Conazole Compounds from the Training and the Predicted Sets^a^

a For training compounds, activity corresponds to the experimentally measured activity. For predicted compounds, activity refers to the classification by the predicted consensus score. “DS” indicates the docking score, and “ML votes” refers to the number of active predictions out of the nine machine learning models. Additionally, the lipophilicity of the compounds is described by the calculated logP. Counterions were removed during standardization.

Examination of the NIS binding pocket supports positive predictions for compounds with ortho-chlorine substituents, as they orient toward a hydrophobic cavity. For instance, Cypro- and Tetraconazole adopt very similar binding conformations (Figure 6a). Nevertheless, Tetraconazole which contains an ortho-substituted chlorine was classified as active, while Cyproconazole was measured as inactive, likely due to differences in their lipophilicity.

**Figure 6:**
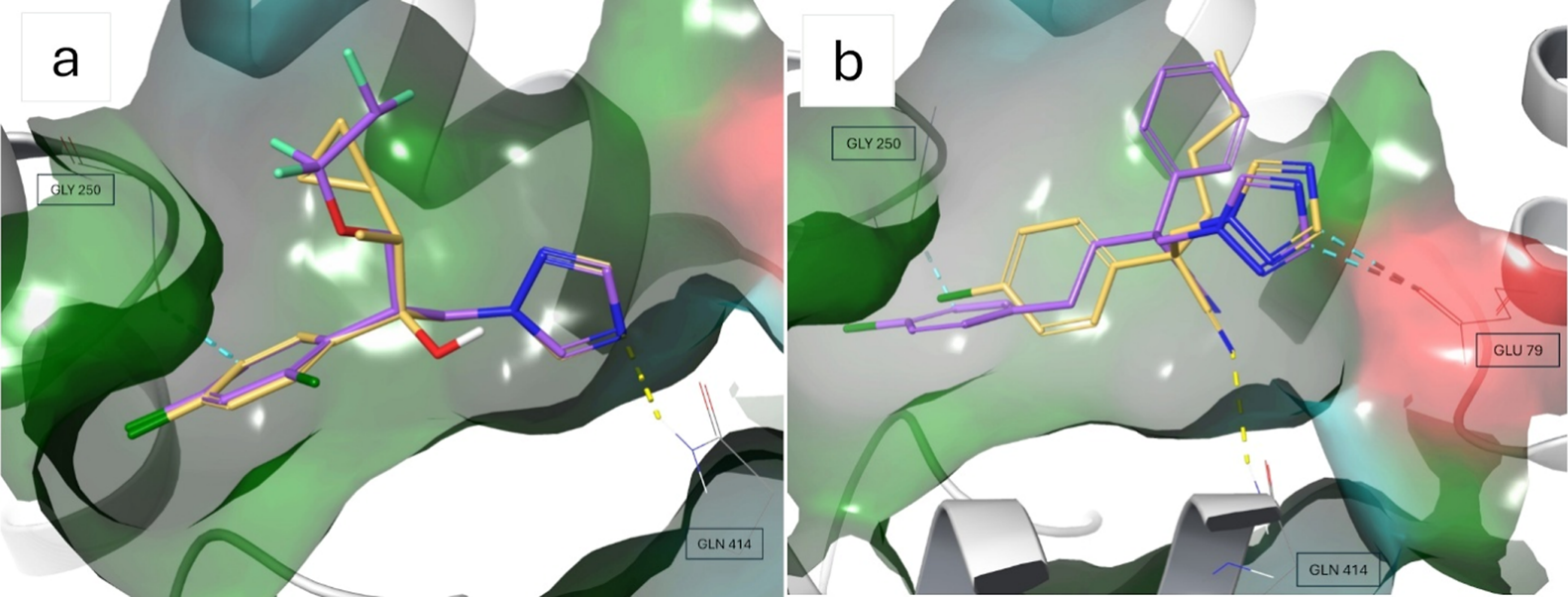
Docking pose examples of conazoles. The surface of the binding pocket was colored according to residue properties. Hydrophobic areas are green, areas with glycine residues are gray, blue stands for polar uncharged and red for negatively charged areas. Blue dashed lines refer to aromatic hydrogen bonds and yellow dashed lines to hydrogen bonds. (a) Positively predicted Tetraconazole (in purple) and negatively measured Cyproconazole (in yellow). Tetraconazole contains groups with higher lipophilicity in lipophilic areas in the pocket, e.g. an additional chlorine at the chlorophenyl ring in the lipophilic cavity (left). (b) Actively predicted Fenbuconazole (in purple) and negatively measured Myclobutanil (in yellow). Fenbuconazole exhibits a deeper fit into the hydrophobic cavity at the left side.

In another example, Fenbuconazole was also classified as active by the consensus score, while its five nearest neighbors, showing a Tanimoto similarity ranging from 0.65 to 0.32, were all measured to be inactive. Fenbuconazole's nearest neighbor Myclobutanil contains a butyl group instead of the phenyl ring and a shorter linker between the nitrile and chlorophenyl moieties. These features reduce lipophilicity and likely alter the binding mode. Docked poses (Figure 6b) show that the triazole rings of both compounds are stabilized by an aromatic hydrogen bond with Glu 79. However, due to the shorter linker in myclobutanil, the chlorophenyl ring adopts a worse fit into the hydrophobic pocket. This aligns with the docking scores, according to which Myclobutanil was classified as inactive and Fenbuconazole as active.

To quantify the effect of lipophilicity, logP values were calculated using the rdkit.Chem.Crippen module. Boxplots were created to compare logP distributions across measured and predicted activity classes for both ML-only and consensus predictions (Figure 7). In the ML-based plot (left), a clear separation between actives and inactives can be observed at a logP of around 3.5, with the exception of Ketoconazole, which is structurally distinct from the other conazoles. In contrast, the consensus-based (right) plot shows a larger overlap in logP values between activity classes, suggesting that the integration of docking scores reduces the lipophilicity-based separation observed in the ML-only model. Metconazole, which lies just below this threshold, was predicted as inactive by the ML model but as active in the consensus approach. Compared to the negatively measured Triticonazole, it contains a chlorophenylmethyl-instead of a chlorophenyliden-group (single bond instead of double bond). This suggests that the ML model may have correctly predicted Metconazole's inactivity, while docking, assigning nearly identical scores to both compounds, misclassified them as active. It is tempting to speculate that the lipophilic nature of the NIS binding pocket accommodating the chlorophenyl moiety present in almost all conazoles might put a bias on the docking-based classification.

**Figure 7:**
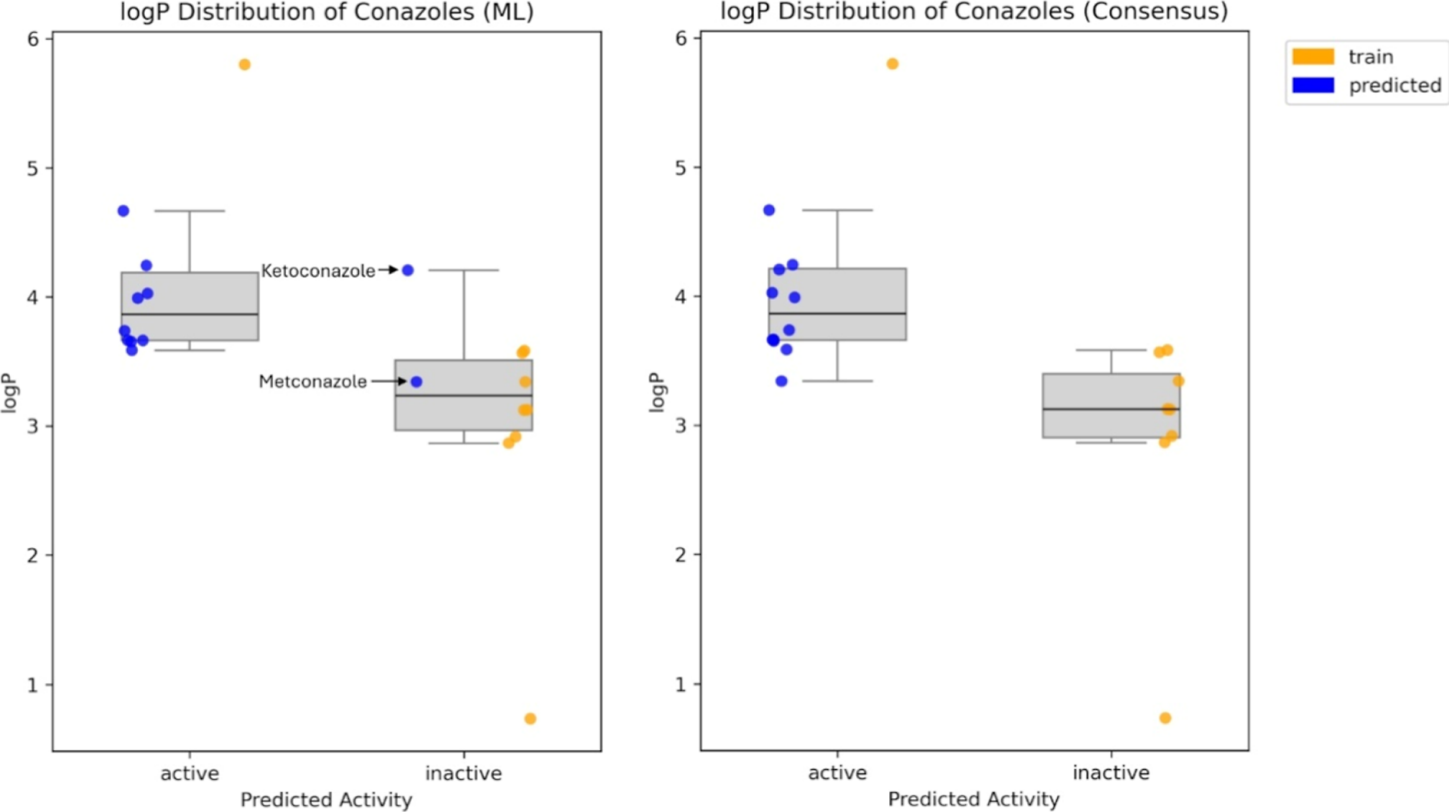
Boxplots showing logP distributions of active and inactive compounds. Compounds from the training set have experimentally confirmed activities and are shown in orange (“train”). Predicted compounds are shown in blue (“predicted”). The right plot is based on consensus predictions, that combine machine learning and docking. In the left plot, only machine learning predictions were considered. Boxes represent the interquartile range with whiskers that extend to the rest of the data, excluding outliers. The central line in the boxes indicates the median.

### Classification of ChEMBL Compounds

An external test set consisting of 70 compounds tested in vitro on rat NIS was retrieved from the ChEMBL database (release 35).^48^ Following the recommendations of the publicly available IDG (Illuminating the Druggable Genome) initiative^49^ a threshold of ≤1 μM (corresponding to a pChEMBL value ≥6) was used to assign binary classification labels. Because this threshold resulted in a highly imbalanced data set with approximately 96% actives, additional thresholds of 6.5 and 7 were explored. The same workflow described in “Classification of Cytotoxic Actives” was applied, and the resulting predictions were compared with the binary classifications derived from the respective pChEMBL thresholds. The generated scores and resulting predictions are listed in the Supporting Information (Table S6).

The anions BF_4_^–^, PF_6_^–^, NO_3_^–^, and ClO_4_^–^ were removed during standardization prior to ML due to a filter that removes inorganic compounds. Hence, no predictions were generated for them by either ML or the consensus scoring approach. Nevertheless, these compounds were still included in the molecular docking workflow. However, the docking scoring function yielded poor scores, suggesting weak binding and resulting in false negative predictions. Possible reasons for this could include the small ligand size, which limits their ability to form interactions, and their charge which often poses challenges for force field based docking scoring functions.^50^

As shown in the performance metrics (Table 5), docking alone generally outperformed the consensus approach across all thresholds. This discrepancy may be due to issues with the applicability domain of the ML model, as discussed in the “Chemical Space Analysis” section.

**Table 5:** Performance Metrics of the Consensus- and Docking-Based Classification Approaches on the External Test Set Retrieved from ChEMBL^a^

pChEMBL threshold	6		6.5		7	
ratio inactives: actives	5:65		21:49		36:34	
	consensus approach	docking	consensus approach	docking	consensus approach	docking
accuracy	0.54	0.71	0.49	0.77	0.31	0.64
BA	0.75	0.73	0.50	0.73	0.31	0.64
MCC	0.25	0.23	0.00	0.45	–0.39	0.30
precision	1.00	0.98	0.71	0.85	0.3	0.6
recall	0.51	0.71	0.48	0.82	0.28	0.82
F1 score	0.67	0.82	0.57	0.83	0.29	0.69
TP	31	46	22	40	9	28
TN	4	3	10	13	11	16
FP	0	1	9	7	22	19
FN	30	19	24	9	23	6

a TP, TN, FP, and FN denote true positives, true negatives, false positives, and false negatives, respectively.

Overall, the consensus model exhibited lower performance on the external test set compared to the internal test set, with a substantial increase of false negatives. In the context of toxicology where the goal is to identify as many active (potentially toxic) compounds as possible, this outcome is particularly problematic. Interestingly, at a pChEMBL threshold of 7, where the data set exhibits an almost balanced distribution between active and inactive compounds, the consensus approach performed worst, despite the ML model having been trained on a nearly balanced data set and producing balanced predicted activity labels. At this threshold, the model even yields a negative MCC, indicating that the predictions are anticorrelated with the experimental classifications. Overall, the consensus approach fails in the classification task due to limitations in the ML component. In contrast, docking performed notably well, particularly at threshold 6.5.

### Chemical Space Analysis

When calculating the mean Tanimoto similarity of the nearest neighbors between the training and the ChEMBL data set, it was 0.31 indicating relatively low chemical similarity. To assess the applicability domain, MolCompass was employed to visualize the training data and the external test set in a two-dimensional space. For comparative purposes, separate plots were generated for the consensus predictions, as well as for the individual predictions made by the ML and the docking model (Figure 8). In the upper right region of the plots (circled in blue), a substantial number of false negative compounds can be observed in both the ML and the consensus prediction plot. In contrast, many of these compounds were correctly identified as active by the docking approach. Notably, within this region, these compounds appear to occupy a different chemical space than those in the training set, indicating limitations in the applicability domain of the ML model. This behavior is consistent with the composition of the ChEMBL data set, where a large fraction of compounds originates from a structure–activity relationship study in which small, systematic modifications were introduced around a lead scaffold to optimize NIS inhibition for the treatment of hyperthyroidism.^51^ As a result, this set represents a narrow congeneric series that occupies a region of chemical space that is only sparsely represented in the ML training data.

**Figure 8:**
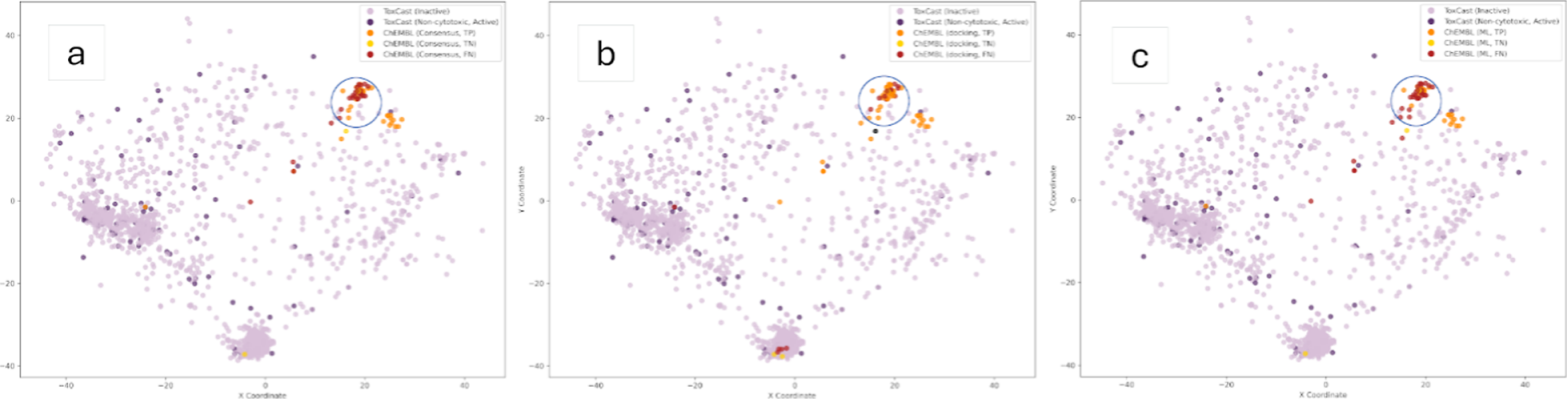
Visualization of external test set from ChEMBL (shaded in orange) and training data (shaded in purple), based on chemical space mapping using MolCompass. Dark purple indicates measured actives, while light purple indicates measured inactives. For the external test set, dark orange represents true positives, yellow represents true negatives and red represents false negatives, based on comparison to the binary classifications at a pChEMBL threshold of 6. In (a) ChEMBL compounds were shaded according to consensus predictions, in (b) according to docking-based predictions, and in c, according to ML-based predictions. The regions circled in blue indicate differences in predictions made by different models for compounds outside the applicability domain.

This finding illustrates the advantage of the consensus approach, as docking is data-naïve and not limited by the applicability domain of the ML model. Unlike data-driven approaches, it evaluates ligand–protein interactions a priori for each compound, without relying on previously learned chemical information. Consequently, it can correctly identify active compounds even when they lie outside the applicability domain. This example further underscores the importance of assessing the applicability domain, comparing results obtained with different predictive methods, and investigating whether contrasting predictions arise from chemical space outliers.

## Conclusion

In the present study, a binary classification model for the prediction of NIS inhibitors was developed, combining molecular docking with ML methods. By integrating the two approaches, additional structural information was incorporated, enhancing the model’s classification capabilities, hence, contributing to a more comprehensive understanding of potential NIS inhibitors. Notably, by screening and classifying a set of compounds with inconclusive activity, the model demonstrated its utility in addressing cases where inhibition through specific binding could not be assessed in the applied in vitro assay due to cytotoxicity. Although the predictive performance of the model was limited, combining its output with visual inspection of docking poses and applicability domain analyses provided a preliminary assessment of which compounds might deserve further investigation.

In the broader context of DNT assessment, this approach could serve as a part of an initial screening tool to prioritize compounds for in vitro testing, reducing the experimental efforts and focusing resources on the most alerting chemicals. It is important to note that the NIS model alone is insufficient for predicting DNT and must be integrated with other models that incorporate additional MIEs for a more comprehensive assessment. In this regard, compounds can also be tested across various models, each simulating different DNT related MIEs, enabling the identification of relevant targets. This approach allows for a focus on the relevant protein targets in subsequent experimental studies.

The presented work introduces an in silico-based new approach method in alignment with next generation risk assessment strategies, supporting animal-free risk assessment. Moving forward, validating this method through in vitro experiments and developing further models of MIEs leading to thyroid hormone homeostasis disruption will provide a more comprehensive picture about the relationship between disrupted thyroid pathways and the onset of DNT. Taken together, these efforts will contribute to the continued advancement of predictive toxicology.

## Supplementary Material



## Data Availability

A jupyter notebook with the standardization workflow, the final ML models, and the consensus scoring workflow are publicly available at: https://github.com/PharminfoVienna/NIS_model_notebook.

## Notes

Schematic overview of data composition and partitioning (Figure S1); structures of NIS in distinct conformational states (Figure S2); ROC plots for docking-based virtual screening results (Figure S3); KDE plots for docking-based virtual screening results (Figure S4); KDE plots for consensus scoring results with optimized parameters in the ML component (Figure S5); KDE plots for consensus scoring results with default parameters in the ML component (Figure S6); ROC AUC values, binding pocket SASAs, and molecular surface areas for different trajectory frames (Table S1); performance metrics for baseline ML models (Table S2); MCCs for baseline ML models trained with retained stereochemistry (Table S3); performance metrics for ML models trained on undersampled subsets with default parameters (Table S4); predictions for compounds that decreased iodide uptake but also exhibited cytotoxicity in vitro (Table S5); predictions for the external test set, derived from ChEMBL (Table S6); list of selected ML parameters (“Selected parameters in machine learning”); list of nearest neighbors for each predicted cytotoxic compound (“Nearest neighbors of predicted cytotoxic compounds”); representative examples of predicted cytotoxic compounds used to assess the interpretability of the consensus approach (“Additional example cases”, including Tables S7–S12 and Figure S7) (PDF)

## Abbreviations

AOP: adverse outcome pathway
BA: balanced accuracy
BLAST: basic local alignment search tool
CDDD: continuous and data driven descriptors
CV: cross validation
DNT: developmental neurotoxicity
DS: docking score
ECFP: extended connectivity fingerprint
HTVS: high throughput virtual screening
IDG: illuminating the druggable genome
KDE: kernel density estimation
MCC: Matthews correlation coefficient
MD: molecular dynamics
MIE: molecular initiating event
ML: machine learning
NIS: sodium iodide symporter
QSAR: quantitative structure activity relationship
RAIU: radioactive iodine uptake
RF: random forest
ROC AUC: receiver operating characteristics area under the curve
SASA: solvent accessible surface area
SGLT-2: sodium glucose transporter 2
SLC: solute carrier
SP: standard precision
SVM: support vector machine
T3: 3,5,3′-triiodo-l-thyronine
T4: 3,5,3′,5′-tetraiodo-l-thyronine
t-SNE: t-distributed stochastic neighbor embedding
XGB: extreme gradient boosting
XP: extra precision
