# Supplementary material for: Combined Modeling Approaches for Assessing Sodium-Iodide Symporter Inhibition
Source: J Chem Inf Model. 2026 Jan 23;66(3):1688–703. doi: 10.1021/acs.jcim.5c02855 (PMC12892324; doi:10.1021/acs.jcim.5c02855)
Supplement: Supplementary file 1 [file ci5c02855_si_002.pdf]

Supporting Information

Combined modeling approaches for assessing sodium-iodide symporter inhibition

*Julia Kandler, Ayse Sıla Kantarçeken, Aljoša Smajić, and Gerhard F. Ecker**

[*]University of Vienna, Department of Pharmaceutical Sciences, Josef-Holaubek-Platz 2, 1090 Vienna, Austria

Table of Contents

Additional figures and tables 2
Selected parameters in machine learning 20
Nearest neighbors of predicted cytotoxic compounds 21
Additional example cases 337

Additional figures and tables

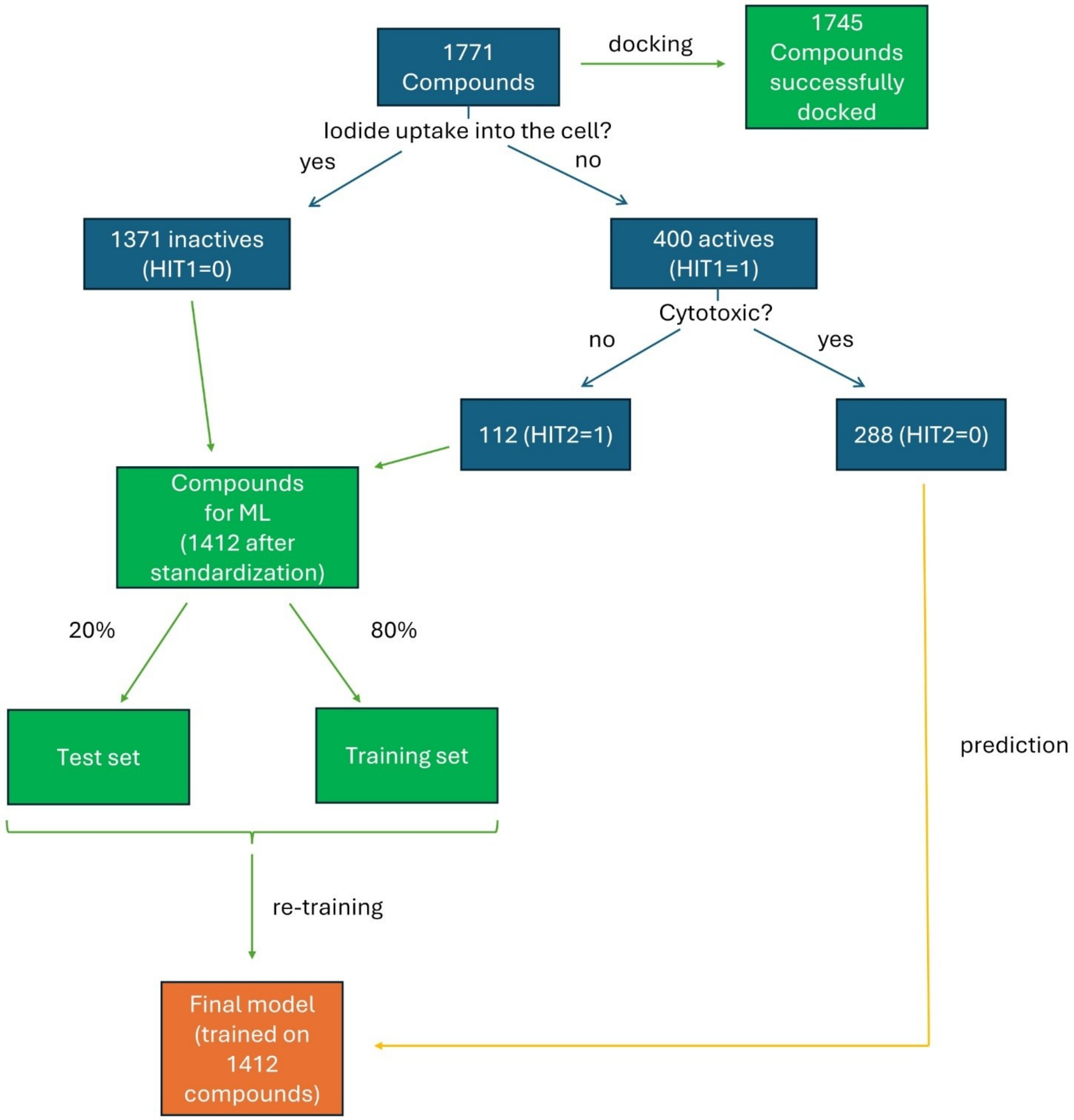

Figure S1. Schematic overview of the dataset composition and data partitioning. The diagram illustrates the classification of screened compounds based on iodide uptake inhibition (HIT1), cytotoxicity filtering leading to the HIT2 subset, and the subsequent selection of compounds for molecular docking and machine learning.

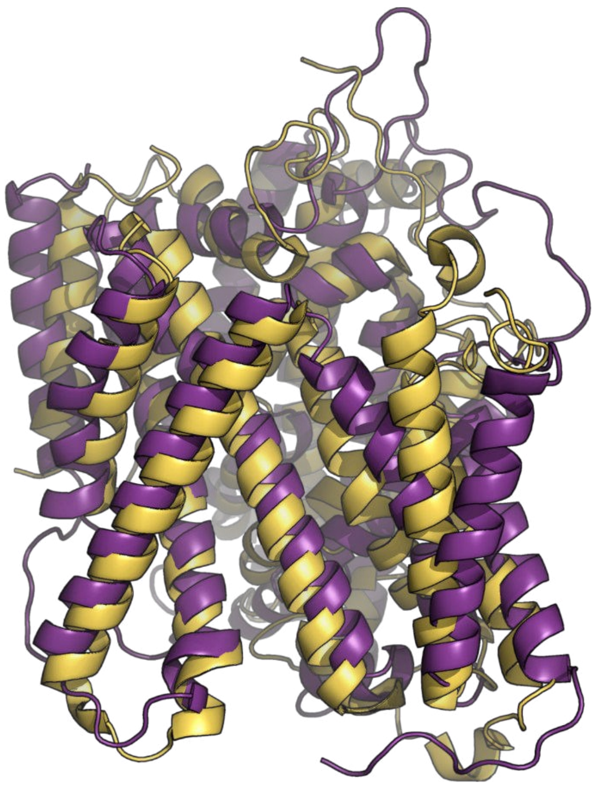

Figure S2. Structures of the sodium-iodide symporter (NIS) in distinct conformational states. The inward open state (PDB ID: 7UUY) is shown in yellow, and an outward-facing intermediate state derived from a molecular dynamics trajectory published by Chakrabarti et al.[25] is shown in purple). Visualization was performed with PyMol 2.5.2.

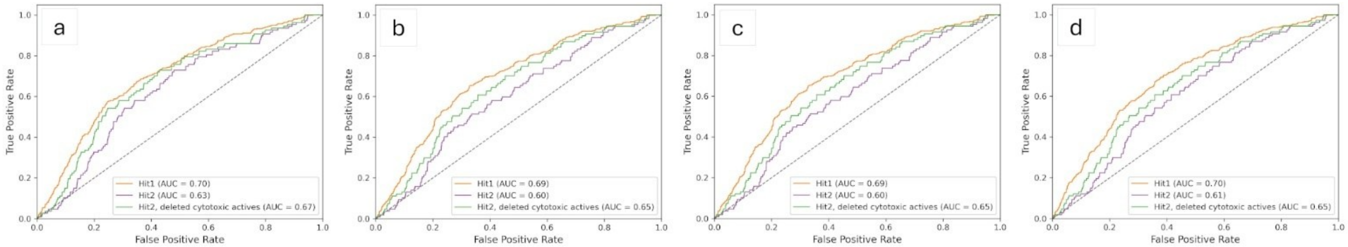

Figure S3. Receiver operating characteristic (ROC) curves of docking-based virtual screening results for the NIS model (frame 249) under different enrichment conditions based on docking scores. HIT1 includes all active compounds (cytotoxic and non-cytotoxic), whereas HIT includes only non-cytotoxic actives. The orange curve (HIT1) represents results from docking the complete set of 1751

compounds, considering the enrichment of the 281 HIT1 actives. The purple curve (HIT2) shows results from docking the same 1751 compounds treating only the 112 HIT2 actives as positive. The green curve (HIT2, deleted cytotoxic actives) reflects results from docking only the 1470 inactive and the 112 non-cytotoxic active compounds, with enrichment of HIT2 actives. Panels (a-d) show the enrichments for the transporter model containing **a**, no ions **b**, one sodium ion, **c**, two sodium ions, and **d**, two sodium ions and one iodide ion.

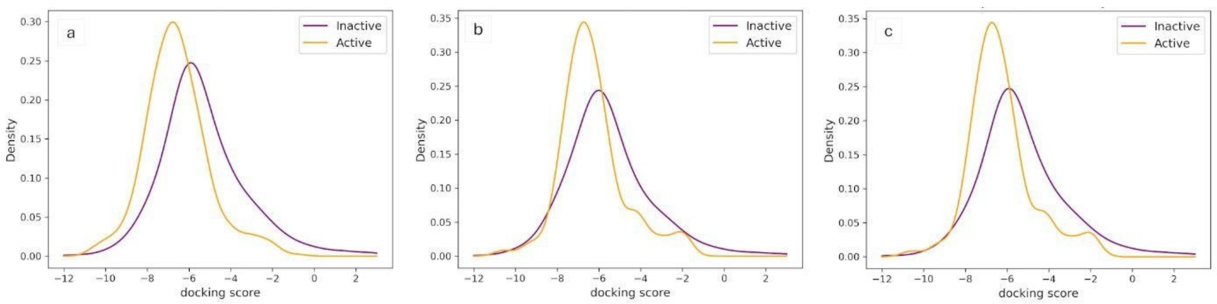

Figure S4. Distribution curves for actives and inactives at their docking scores. The density distributions are illustrated for different compound classifications. The orange function represents the actives, whereas the purple function represents the inactives. A shift of the active curve toward the inactive curve can be observed when cytotoxic actives are reclassified or excluded. **a**, actives contain all 281 tested actives, while inactives contain all 1470 tested inactives. **b**, actives contain the 112 non-cytotoxic actives, and inactives include all 1470 tested inactives and the 169 cytotoxic tested actives. **c**, actives contain only the 112 non-cytotoxic tested actives, and inactives include the 1470 tested inactives.

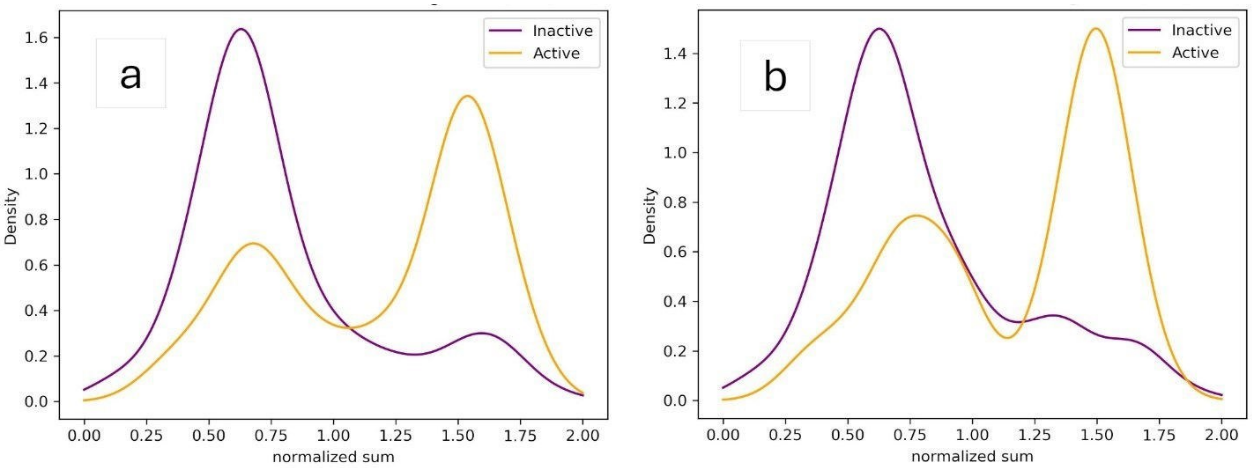

Figure S5. Distribution curves of actives (orange) and inactives (purple) plotted against their consensus scores (normalized sum). The consensus score was calculated by combining normalized docking scores with normalized scores from different ML models: a, support vector machine and b, extreme gradient boosting. The models were trained using optimized parameters.

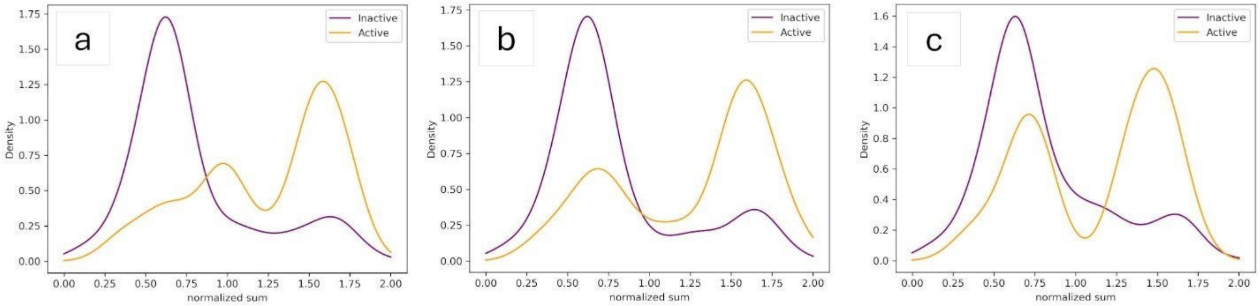

Figure S6. Distribution curves of actives (orange) and inactives (purple) plotted against their consensus scores (normalized sum). The consensus score was calculated by combining normalized docking scores with normalized scores from different ML models: **a**, random forest, **b**, support vector machine, and **c**, extreme gradient boosting. All models were trained using default parameters.

Table S1. Binding pocket solvent accessible surface area (SASA) and molecular surface area for

each frame of the trajectory describing the transition between inward and outward open states of the sodium-iodide symporter. ROC AUC values are reported only for frames in which docking was performed and correspond to docking into protein models without bound ions. The trajectory was retrieved from Chakrabarti et al[25].

Frame Number	ROC AUC Hit1	ROC AUC Hit2	SASA	Surface Area (A^2)
1	0.63	0.55	919.60	4532.25
2	0.68	0.6	1056.72	4530.34
3	0.7	0.6	1104.92	4549.70
4			998.99	4570.96
5			1129.35	4584.69
6			1159.33	4518.90
7			1053.58	4528.95
8	0.69	0.6	1130.09	4534.25
9			1093.98	4577.33
10			1135.38	4576.81
11			1226.78	4524.62
12			1094.53	4558.53
13			1078.96	4533.24
14			1176.86	4486.61
15			1108.73	4509.41
16			1053.85	4551.03
17			1114.69	4521.69
18			1073.05	4540.28
19	0.68	0.57	1161.06	4531.57
20			1171.02	4546.48
21	0.7	0.62	1034.87	4556.81
22			1087.44	4512.34
23			1118.66	4548.79
24	0.66	0.58	1158.77	4548.63
25			1055.72	4517.94
26			1127.95	4552.91
27			874.85	4531.12
28			1108.17	4569.56
29			1172.88	4476.61
30			968.99	4536.07
31			1117.80	4575.12
32			1283.89	4469.46
33			1138.08	4532.07
34			1222.05	4566.20
35			1233.57	4545.59

36			1217.63	4552.01
37			998.13	4549.62
38			1065.36	4555.87
39			1192.70	4523.18
40			1147.93	4475.52
41			1165.34	4563.91
42	0.65	0.55	1117.73	4479.93
43			941.64	4548.59
44			1103.08	4540.38
45			708.15	4524.75
46			1103.53	4600.48
47			1255.26	4557.48
48			965.64	4519.73
49			1158.54	4483.16
50			1106.37	4472.93
51			1232.20	4510.55
52			1129.20	4573.04
53			1034.26	4563.95
54			1066.94	4524.24
55			1133.29	4572.06
56			975.20	4522.20
57			963.12	4522.38
58			1064.98	4562.95
59			1174.38	4544.28
60			1188.30	4526.33
61			1081.37	4540.44
62			850.97	4523.17
63			1217.50	4591.98
64	0.69	0.6	1097.51	4573.07
65			1232.11	4516.18
66			1226.67	4572.22
67			1099.12	4534.42
68			1225.99	4547.97
69			1149.05	4510.33
70			1109.65	4548.08
71			1261.12	4550.54
72			1145.73	4564.11
73			1101.60	4539.06
74			1048.99	4536.66
75			1140.14	4552.56
76			1100.71	4546.03
77			1173.71	4535.91
78			1210.18	4569.45

79			1159.99	4519.17
80			1149.40	4487.88
81			1227.81	4534.93
82			1118.30	4512.84
83			1188.64	4598.13
84			1165.14	4557.16
85			1130.89	4544.87
86			1029.46	4568.57
87			1155.18	4559.90
88			1138.96	4538.90
89			1088.10	4557.97
90			983.03	4510.07
91			1286.93	4531.70
92	0.64	0.56	809.05	4564.86
93			978.12	4571.68
94			1024.80	4531.58
95			1178.06	4524.73
96			1142.48	4536.64
97			1125.72	4534.21
98			1270.52	4528.34
99			984.86	4553.66
100			1194.53	4523.44
101			1170.32	4543.15
102			1136.98	4506.05
103			1069.89	4529.41
104			1069.56	4564.30
105			1256.54	4580.14
106			1184.12	4548.88
107			1248.82	4534.85
108			1061.31	4525.04
109			1067.71	4532.59
110			1104.36	4538.05
111			1109.13	4530.93
112			1095.95	4579.68
113			1119.77	4541.06
114			1137.21	4537.17
115			1182.20	4589.09
116			933.19	4553.88
117			1168.16	4555.62
118	0.69	0.6	811.61	4506.75
119			1155.22	4540.68
120			1264.24	4541.31
121			1131.20	4536.40

122			878.49	4501.93
123			1116.39	4484.96
124			898.87	4512.86
125			845.50	4518.16
126			853.33	4537.34
127			867.40	4527.03
128			1131.38	4547.77
129			1166.72	4534.86
130			1217.04	4561.45
131			1102.82	4560.92
132			856.97	4530.90
133			1028.17	4537.81
134			1185.82	4531.17
135			963.82	4478.42
136			969.13	4550.56
137			996.05	4541.51
138			1162.57	4584.11
139			819.76	4494.94
140	0.69	0.61	1053.75	4522.40
141			785.29	4514.25
142			827.88	4534.64
143			892.74	4501.65
144			862.66	4530.60
145			977.49	4534.07
146			1309.20	4533.63
147			1185.98	4513.41
148			811.98	4527.80
149			975.64	4470.32
150			1221.52	4500.75
151			848.03	4507.43
152			1163.04	4492.87
153			1249.37	4528.44
154			1066.48	4541.28
155			767.55	4489.65
156			853.70	4526.65
157			930.09	4543.47
158			1311.98	4531.19
159			922.30	4482.47
160			878.02	4538.67
161			1075.14	4522.08
162			910.63	4550.70
163			1094.89	4549.13
164			1233.14	4522.67

165			1051.88	4561.44
166			837.72	4518.88
167			851.39	4566.44
168			810.41	4543.57
169			742.83	4538.35
170			809.06	4522.45
171			950.91	4524.66
172			877.29	4504.10
173			1261.02	4552.67
174			901.75	4526.96
175			879.37	4474.52
176			851.70	4536.36
177			893.96	4510.75
178			1130.92	4540.66
179			1155.85	4543.76
180			1255.62	4519.71
181			1098.17	4525.63
182			931.41	4554.21
183			897.58	4511.05
184			928.50	4549.10
185			1127.58	4504.14
186			877.43	4529.59
187			912.98	4557.39
188	0.65	0.57	1258.59	4551.36
189			1026.86	4545.05
190			1342.79	4524.31
191			897.17	4531.85
192			1027.47	4531.54
193			1263.47	4530.19
194			919.76	4545.04
195			1234.81	4529.38
196			877.92	4515.92
197			1152.15	4532.99
198			889.62	4518.66
199			1121.36	4505.01
200			1028.41	4555.01
201			881.77	4483.31
202			1171.74	4559.03
203			1320.93	4529.51
204			729.54	4542.15
205			1239.63	4498.27
206			911.87	4558.93
207			1000.11	4543.50

208			1231.56	4497.36
209			1084.43	4507.42
210			889.26	4500.55
211	0.7	0.61	1219.75	4548.18
212			830.59	4542.37
213			919.48	4530.12
214			1296.05	4499.27
215			980.44	4565.99
216			972.38	4545.89
217			899.84	4507.05
218			1331.21	4537.98
219			1021.35	4535.68
220			851.31	4521.70
221			911.75	4499.90
222			1001.27	4536.29
223			1147.99	4547.50
224			816.73	4506.55
225			1344.94	4542.12
226			1210.85	4533.63
227			992.02	4518.28
228			1045.15	4484.10
229			1086.93	4528.81
230	0.61	0.58	848.20	4541.19
231			1175.17	4509.08
232			1319.04	4535.24
233			1157.90	4495.33
234			1290.28	4512.96
235			866.69	4525.59
236			984.91	4495.06
237			1252.22	4503.75
238			847.09	4520.46
239			1009.02	4490.93
240			1470.91	4564.70
241			1465.00	4535.96
242			846.07	4519.95
243			1328.27	4512.22
244			857.25	4554.68
245			1285.48	4547.86
246			1035.19	4514.75
247			868.93	4535.12
248			1194.18	4532.34
249	0.7	0.64	879.11	4574.65

Table S2. Performance metrics on the test and training set across different ML algorithms and molecular representations, based on training on the imbalanced dataset (baseline models). The training performance is reported as the mean and standard deviation of MCC and BA across the nine outer cross-validation folds.

		ECFP4			CDDD		
		RF	SVM	XGB	RF	SVM	XGB
Training	MCC	0.16 ± 0.16	0.20 ± 0.08	0.23 ± 1.3	0.04 ± 0.11	0.42 ± 0.19	0.22 ± 0.13
	BA	0.54 ± 0.04	0.59 ± 0.04	0.57 ± 0.04	0.51 ± 0.03	0.66 ± 0.07	0.56 ± 0.05
Test	MCC	-0.02	0.21	0.18	- 0.02	0.29	0.24
	BA	0.50	0.60	0.56	0.5	0.61	0.57

Table S3. Matthews correlation coefficients (MCCs) for baseline ML models trained on CDDDs with retained stereochemistry. The training performance is reported as the mean and standard deviation across the nine outer cross-validation folds.

	MCC (train)	MCC (test)
**RF**	0.09 ± 0.14	-0.02
**SVM**	0.32 ± 0.22	0.22
**XGB**	0.18 ± 0.21	0.17

Table S4. Performance metrics of the test and training set across different ML algorithms and molecular representations for predictions resulting from majority voting, based on training on undersampled subsets with default parameters. The training performance is reported as the mean and standard deviation of MCC and BA across the nine outer cross-validation folds.

		ECFP4			CDDD		
		RF	SVM	XGB	RF	SVM	XGB

**Training**	**MCC**	0.27 ± 0.08	0.19 ± 0.10	0.19 ± 0.10	0.35 ± 0.10	0.38 ± 0.12	0.37 ± 0.12
	**BA**	0.69 ± 0.06	0.64 ± 0.07	0.66 ± 0.07	0.77 ± 0.07	0.80 ± 0.09	0.79 ± 0.08
**Test**	**MCC**	0.18	0.23	0.14	0.26	0.27	0.23
	**BA**	0.61	0.67	0.61	0.70	0.72	0.67

Table S5. Compounds tested for NIS inhibition by Wang et al.[23], that showed a decrease in iodide uptake but also exhibited cytotoxicity. The inhibition of NIS by specific binding was predicted by applying a consensus approach that combines docking and random forest models. The table presents the sum of positive random forest predictions, the docking scores, and their respective normalized values. The consensus score, calculated as the sum of the normalized scores, was used to assign binary classification labels in the "Predicted Activity" column based on a threshold of 0.82.

Substance Name	Substance CASRN	Predicted Activity	Random Forest Prediction	Docking Score	Normalized Random Forest Prediction	Normalized Docking Score	Consensus Score
4,4'-Methylenebis(2,6-diethylaniline)	13680-35-8	1	9	-9.62	1.00	0.83	1.83
4,4'-Methylenebis(N,N-dimethylaniline)	101-61-1	1	9	-9.08	1.00	0.8	1.8
Amiodarone hydrochloride	19774-82-4	1	9	-9.02	1.00	0.79	1.79
C.I. Solvent Yellow 14	842-07-9	1	9	-8.71	1.00	0.77	1.77
Trifloxystrobin	141517-21-7	1	9	-8.63	1.00	0.77	1.77
SR125047	NOCAS_47342	1	9	-8.54	1.00	0.76	1.76
4-Hydroxytamoxifen	68392-35-8	1	9	-8.49	1.00	0.76	1.76
Elzasonan	361343-19-3	1	9	-8.40	1.00	0.75	1.75
Clomiphene citrate (1:1)	50-41-9	1	9	-8.34	1.00	0.75	1.75
UK-337312	203942-49-8	1	7	-10.44	0.78	0.89	1.67
2,2'-Methylenebis(4-methyl-6-tert-butylphenol)	119-47-1	1	9	-8.26	1.00	0.74	1.74
Famoxadone	131807-57-3	1	9	-8.18	1.00	0.74	1.74
Chlorophacinone	3691-35-8	1	9	-8.12	1.00	0.73	1.73
2,2'-Methylenebis(ethyl-6-tert-butylphenol)	88-24-4	1	9	-8.06	1.00	0.73	1.73
1-(2-Chlorophenyl)-N-methyl-N-(1-methylpropyl)-3-isoquinolinecarboxamide	85532-75-8	1	9	-8.06	1.00	0.73	1.73
Difenoconazole	119446-68-3	1	9	-8.05	1.00	0.73	1.73
Celecoxib	169590-42-5	1	9	-8.01	1.00	0.73	1.73
Benfluralin	1861-40-1	1	9	-7.91	1.00	0.72	1.72
C.I. Disperse Orange 37	13301-61-6	1	9	-7.85	1.00	0.72	1.72
Pyriproxyfen	95737-68-1	1	9	-7.83	1.00	0.71	1.71
N-Phenyl-1-naphthylamine	90-30-2	1	9	-7.83	1.00	0.71	1.71
Auramine hydrochloride	2465-27-2	1	8	-8.91	0.89	0.79	1.68
Cyhalofop-butyl	122008-85-9	1	9	-7.83	1.00	0.71	1.71
Epoxiconazole	133855-98-8	1	9	-7.75	1.00	0.71	1.71
Diethylstilbestrol	56-53-1	1	9	-7.68	1.00	0.7	1.7
Pyridaben	96489-71-3	1	9	-7.62	1.00	0.7	1.7

Tamoxifen citrate	54965-24-1	1	9	-7.60	1.00	0.7	1.7
Tamoxifen	10540-29-1	1	9	-7.60	1.00	0.7	1.7
Gentian Violet	548-62-9	1	9	-7.58	1.00	0.7	1.7
Fenamidone	161326-34-7	1	9	-7.53	1.00	0.69	1.69
2-tert-Butyl-4-ethylphenol	96-70-8	1	9	-7.52	1.00	0.69	1.69
HMR1426	262376-75-0	1	9	-7.50	1.00	0.69	1.69
Pyraclostrobin	175013-18-0	1	9	-7.43	1.00	0.69	1.69
meso-Hexestrol	84-16-2	1	9	-7.40	1.00	0.69	1.69
(2R,6S)-Fenpropimorph	67564-91-4	1	9	-7.39	1.00	0.69	1.69
Fluazinam	79622-59-6	1	9	-7.39	1.00	0.68	1.68
4-Cumylphenol	599-64-4	1	9	-7.36	1.00	0.68	1.68
Hexaconazole	79983-71-4	1	9	-7.36	1.00	0.68	1.68
Quinoxyfen	124495-18-7	1	9	-7.35	1.00	0.68	1.68
Rhodamine B	81-88-9	1	8	-8.37	0.89	0.75	1.64
Butylated hydroxytoluene	128-37-0	1	9	-7.23	1.00	0.67	1.67
Triphenyl phosphate	115-86-6	1	9	-7.23	1.00	0.67	1.67
Benzyl salicylate	118-58-1	1	9	-7.22	1.00	0.67	1.67
Dicumyl peroxide	80-43-3	1	9	-7.20	1.00	0.67	1.67
Benzyl 4-hydroxybenzoate	94-18-8	1	9	-7.18	1.00	0.67	1.67
2-(2,6-Diisopropylphenyl)-5-hydroxy-1H-isoindole-1,3-dione	105624-86-0	1	9	-7.14	1.00	0.67	1.67
2-(Thiocyanomethylthio)benzothiazole	21564-17-0	1	9	-7.12	1.00	0.67	1.67
1-(2-Chlorophenyl)-1-(4-chlorophenyl)-2,2-dichloroethane	53-19-0	1	9	-7.11	1.00	0.67	1.67
2-Chloro-4-phenylphenol	92-04-6	1	9	-7.08	1.00	0.66	1.66
7,12-Dimethylbenz(a)anthracene	57-97-6	1	8	-8.13	0.89	0.73	1.62
Fluazifop-butyl	69806-50-4	1	9	-7.04	1.00	0.66	1.66
Celestolide	13171-00-1	1	8	-8.10	0.89	0.73	1.62
2,2-Bis(4-hydroxyphenyl)-1,1,1-trichloroethane	2971-36-0	1	9	-7.01	1.00	0.66	1.66
Fenofibrate	49562-28-9	1	9	-7.01	1.00	0.66	1.66
2,6-Di-tert-butylphenol	128-39-2	1	9	-7.00	1.00	0.66	1.66
Bisphenol AF	1478-61-1	1	9	-6.97	1.00	0.66	1.66
p,p'-DDD	72-54-8	1	9	-6.95	1.00	0.66	1.66
Fenbuconazole	114369-43-6	1	8	-7.98	0.89	0.72	1.61
DDT	50-29-3	1	9	-6.89	1.00	0.65	1.65
Diniconazole	83657-24-3	1	9	-6.87	1.00	0.65	1.65
Tetraconazole	112281-77-3	1	9	-6.85	1.00	0.65	1.65
p,p'-DDE	72-55-9	1	9	-6.83	1.00	0.65	1.65
Dichlorophen	97-23-4	1	9	-6.83	1.00	0.65	1.65
Bisphenol B	77-40-7	1	9	-6.79	1.00	0.64	1.64
5-Chloro-N-(2-chloro-4-nitrophenyl)-2-hydroxybenzamide	50-65-7	1	9	-6.79	1.00	0.64	1.64
Triflumizole	68694-11-1	1	9	-6.79	1.00	0.64	1.64
Bis(4-(dimethylamino)phenyl)methanone	90-94-8	1	8	-7.85	0.89	0.72	1.6
Phenolphthalein	77-09-8	1	8	-7.81	0.89	0.71	1.6
Chlorobenzilate	510-15-6	1	9	-6.72	1.00	0.64	1.64
AVE5638	725228-45-5	1	7	-8.87	0.78	0.78	1.56
Phenylparaben	17696-62-7	1	9	-6.68	1.00	0.64	1.64
Prodiamine	29091-21-2	1	9	-6.68	1.00	0.64	1.64
(+-)-Diclofop-methyl	51338-27-3	1	9	-6.68	1.00	0.64	1.64
Fenarimol	60168-88-9	1	9	-6.60	1.00	0.63	1.63
Mepronil	55814-41-0	1	9	-6.60	1.00	0.63	1.63
Chlorfenapyr	122453-73-0	1	9	-6.60	1.00	0.63	1.63
Forchlorfenuron	68157-60-8	1	9	-6.58	1.00	0.63	1.63
2,4-Di-tert-pentylphenol	120-95-6	1	9	-6.55	1.00	0.63	1.63

Fenoxycarb	72490-01-8	1	9	-6.53	1.00	0.63	1.63
Amitraz	33089-61-1	1	9	-6.53	1.00	0.63	1.63
Picoxystrobin	117428-22-5	1	9	-6.49	1.00	0.62	1.62
Flusilazole	85509-19-9	1	8	-7.56	0.89	0.7	1.59
Phenolphthalin	81-90-3	1	8	-7.55	0.89	0.7	1.58
9-Phenanthrol	484-17-3	1	7	-8.63	0.78	0.77	1.55
SB243213A	200940-23-4	1	8	-7.54	0.89	0.7	1.58
Fenpyroximate (Z,E)	111812-58-9	1	9	-6.44	1.00	0.62	1.62
Bifenazate	149877-41-8	1	9	-6.44	1.00	0.62	1.62
4,4'-Dichlorodiphenyl sulfone	80-07-9	1	9	-6.44	1.00	0.62	1.62
4-tert-Butylphenyl salicylate	87-18-3	1	9	-6.39	1.00	0.62	1.62
Quizalofop-ethyl	76578-14-8	1	9	-6.36	1.00	0.62	1.62
2,2',6,6'-Tetrachlorobisphenol A	79-95-8	1	9	-6.30	1.00	0.61	1.61
Methylene blue	61-73-4	1	8	-7.38	0.89	0.68	1.57
Phosalone	2310-17-0	1	9	-6.27	1.00	0.61	1.61
Fenoxaprop-ethyl	66441-23-4	1	9	-6.22	1.00	0.61	1.61
Fenoxaprop-P-ethyl	71283-80-2	1	9	-6.22	1.00	0.61	1.61
Ethalfluralin	55283-68-6	1	9	-6.18	1.00	0.6	1.6
Prochloraz	67747-09-5	1	9	-6.16	1.00	0.6	1.6
Clotrimazole	23593-75-1	1	9	-6.15	1.00	0.6	1.6
Allethrin	584-79-2	1	9	-6.14	1.00	0.6	1.6
4-(1,1,3,3-Tetramethylbutyl)phenol	140-66-9	1	9	-6.12	1.00	0.6	1.6
Carfentrazone-ethyl	128639-02-1	1	8	-7.15	0.89	0.67	1.56
FR150011	149413-74-1	1	7	-8.16	0.78	0.74	1.51
Hexane-1,6-diyl dibenzoate	22915-73-7	1	8	-7.06	0.89	0.66	1.55
Coumaphos	56-72-4	1	9	-5.97	1.00	0.59	1.59
1,3-Diphenyl-1,3-propanedione	120-46-7	1	8	-7.03	0.89	0.66	1.55
Fenthion	55-38-9	1	9	-5.95	1.00	0.59	1.59
Fluazifop-P-butyl	79241-46-6	1	9	-5.94	1.00	0.59	1.59
Fluoxastrobin	361377-29-9	1	8	-7.00	0.89	0.66	1.55
Heptachlor epoxide B	1024-57-3	1	9	-5.89	1.00	0.58	1.58
Captafol	2425-06-1	1	9	-5.89	1.00	0.58	1.58
Dieldrin	60-57-1	1	9	-5.86	1.00	0.58	1.58
Propiconazole	60207-90-1	1	8	-6.89	0.89	0.65	1.54
Perfluorooctanesulfonamide	754-91-6	1	9	-5.80	1.00	0.58	1.58
Aldrin	309-00-2	1	9	-5.79	1.00	0.58	1.58
4,4'-Sulfonylbis[2-(prop-2-en-1-yl)phenol]	41481-66-7	1	8	-6.87	0.89	0.65	1.54
Endosulfan I	959-98-8	1	9	-5.76	1.00	0.58	1.58
o-Aminoazotoluene	97-56-3	1	8	-6.84	0.89	0.65	1.54
Nitrofen	1836-75-5	1	9	-5.76	1.00	0.58	1.58
Flavone	525-82-6	1	8	-6.83	0.89	0.65	1.54
Temephos	3383-96-8	1	9	-5.71	1.00	0.57	1.57
Butralin	33629-47-9	1	9	-5.68	1.00	0.57	1.57
S-Bioallethrin	28434-00-6	1	9	-5.66	1.00	0.57	1.57
Zoxamide	156052-68-5	1	8	-6.73	0.89	0.64	1.53
3,3',5,5'-Tetrabromobisphenol A	79-94-7	1	9	-5.63	1.00	0.57	1.57
Folpet	133-07-3	1	9	-5.61	1.00	0.57	1.57
Tiratricol	51-24-1	1	8	-6.68	0.89	0.64	1.53
CP-105696	158081-99-3	1	7	-7.76	0.78	0.71	1.49
Oxadiazon	19666-30-9	1	9	-5.57	1.00	0.56	1.56
3-Chloro-2-((3R)-5-chloro-1-(2,4-dimethoxybenzyl)-3-methyl-2-oxo-2,3-dihydro-1H-indol-3-yl)-N-ethyl-N-(3-pyridinylmethyl)benzamide hydrochloride (1:1)	1437319-51-1	1	7	-7.73	0.78	0.71	1.49
1,4-Bis(N-	14233-37-5	1	9	-5.56	1.00	0.56	1.56

isopropylamino)anthraquinone								
Tonalide	21145-77-7	1	8	-6.63	0.89	0.63	1.52
Profenofos	41198-08-7	1	9	-5.52	1.00	0.56	1.56
Chrysin	480-40-0	1	7	-7.66	0.78	0.7	1.48
Bisphenol A diglycidyl ether	1675-54-3	1	8	-6.55	0.89	0.63	1.52
Imazalil	35554-44-0	1	9	-5.42	1.00	0.55	1.55
Chlordane	57-74-9	1	9	-5.41	1.00	0.55	1.55
Nordihydroguaiaretic acid	500-38-9	1	6	-8.60	0.67	0.77	1.43
Pendimethalin	40487-42-1	1	9	-5.25	1.00	0.54	1.54
Darbufelone mesylate	139340-56-0	1	9	-5.23	1.00	0.54	1.54
Parathion	56-38-2	1	9	-5.22	1.00	0.54	1.54
Heptachlor	76-44-8	1	9	-5.19	1.00	0.54	1.54
Endosulfan sulfate	1031-07-8	1	9	-5.17	1.00	0.54	1.54
Endosulfan	115-29-7	1	9	-5.17	1.00	0.54	1.54
Tebupirimfos	96182-53-5	1	9	-5.14	1.00	0.53	1.53
Bensulide	741-58-2	1	8	-6.16	0.89	0.6	1.49
Bromuconazole	116255-48-2	1	7	-7.04	0.78	0.66	1.44
p-Bromodiphenyl ether	101-55-3	1	8	-5.92	0.89	0.59	1.47
Chlorpyrifos	2921-88-2	1	9	-4.80	1.00	0.51	1.51
Benz(a)anthracene	56-55-3	1	6	-8.03	0.67	0.73	1.39
Ethion	563-12-2	1	9	-4.74	1.00	0.51	1.51
Mifepristone	84371-65-3	1	5	-9.03	0.56	0.8	1.35
HMR1171 trifluoroacetate (1:1)	NOCAS_48522	1	4	-10.02	0.44	0.86	1.31
Ro 23-7637	107071-66-9	1	4	-9.98	0.44	0.86	1.3
Prallethrin	23031-36-9	1	8	-5.61	0.89	0.57	1.45
Flutamide	13311-84-7	1	7	-6.62	0.78	0.63	1.41
MK-968	NOCAS_47334	1	5	-8.63	0.56	0.77	1.32
Farglitazar	196808-45-4	1	4	-9.65	0.44	0.84	1.28
1H,1H,2H,2H-Perfluorooctyl iodide	2043-57-4	1	8	-5.31	0.89	0.55	1.43
PharmaGSID_47337	1061517-62-1	1	4	-9.52	0.44	0.83	1.27
SAR102779	NOCAS_47387	1	3	-10.57	0.33	0.9	1.23
Propargite	2312-35-8	1	7	-6.17	0.78	0.6	1.38
Thiobencarb	28249-77-6	1	7	-6.12	0.78	0.6	1.38
N-Ethylperfluorooctanesulfonamide	4151-50-2	1	9	-3.85	1.00	0.45	1.45
MK-274	NOCAS_47328	1	6	-7.02	0.67	0.66	1.33
PharmaGSID_48505	NOCAS_48505	1	5	-8.08	0.56	0.73	1.29
PharmaGSID_47330	NOCAS_47330	1	4	-9.15	0.44	0.8	1.25
CJ-013610	249296-43-3	1	3	-10.22	0.33	0.88	1.21
PharmaGSID_48514	NOCAS_48514	1	6	-6.82	0.67	0.65	1.31
Cinmethylin	87818-31-3	1	6	-6.76	0.67	0.64	1.31
Phenanthrene	85-01-8	1	5	-7.80	0.56	0.71	1.27
Ipconazole	125225-28-7	1	5	-7.75	0.56	0.71	1.27
Pentachlorophenol	87-86-5	1	7	-5.38	0.78	0.55	1.33
Pirimiphos-methyl	29232-93-7	1	7	-5.13	0.78	0.53	1.31
Captan	133-06-2	1	6	-6.19	0.67	0.6	1.27
Phorate	298-02-2	1	8	-3.96	0.89	0.45	1.34
Tribufos	78-48-8	1	7	-4.98	0.78	0.52	1.3
PD 0343701	676116-04-4	1	6	-5.99	0.67	0.59	1.26
Acetylcedrene	32388-55-9	1	5	-7.02	0.56	0.66	1.22
Benzyl butyl phthalate	85-68-7	1	5	-6.98	0.56	0.66	1.21
Di(propylene glycol) dibenzoate	27138-31-4	1	5	-6.89	0.56	0.65	1.21
Methyl abietate	127-25-3	1	5	-6.88	0.56	0.65	1.21
Disulfoton	298-04-4	1	9	-2.45	1.00	0.35	1.35
PharmaGSID_48506	588941-45-1	1	4	-7.44	0.44	0.69	1.13
SSR150106	NOCAS_47362	1	5	-6.31	0.56	0.61	1.17

SB236057A	180084-01-9	1	3	-8.42	0.33	0.75	1.09
Metconazole	125116-23-6	1	4	-7.32	0.44	0.68	1.12
PharmaGSID_48519	686756-87-6	1	3	-8.37	0.33	0.75	1.08
Sodium dodecyl sulfate	151-21-3	1	8	-2.73	0.89	0.37	1.26
Dodecyl sulfate triethanolamine salt	139-96-8	1	8	-2.73	0.89	0.37	1.26
Ketoconazole	65277-42-1	1	2	-9.00	0.22	0.79	1.02
SR58611	929601-09-2	1	1	-9.83	0.11	0.85	0.96
AVE6324	NOCAS_47377	1	2	-8.50	0.22	0.76	0.98
4-Hexylresorcinol	136-77-6	1	4	-6.24	0.44	0.61	1.05
C.I. Acid Orange 156	68555-86-2	1	3	-7.28	0.33	0.68	1.01
SSR 241586 HCl	NOCAS_47353	1	2	-8.29	0.22	0.75	0.97
Heptyl p-hydroxybenzoate	1085-12-7	1	6	-3.79	0.67	0.44	1.11
Fluthiacet-methyl	117337-19-6	1	3	-6.72	0.33	0.64	0.97
Farnesol	4602-84-0	1	6	-3.39	0.67	0.42	1.08
C.I. Acid Red 114	6459-94-5	1	0	-9.84	0.00	0.85	0.85
Testosterone propionate	57-85-2	1	3	-6.56	0.33	0.63	0.96
Didecylmethyl(3-(trimethoxysilyl)propyl)ammonium chloride	68959-20-6	1	2	-7.64	0.22	0.7	0.92
Chlorhexidine diacetate	56-95-1	1	3	-6.54	0.33	0.63	0.96
Diclosulam	145701-21-9	1	4	-5.40	0.44	0.55	1
2-Ethylhexyl p-hydroxybenzoate	5153-25-3	1	5	-4.31	0.56	0.48	1.03
Sodium tridecyl sulfate	3026-63-9	1	6	-3.23	0.67	0.41	1.07
2-(Phenylmethylene)octanal	101-86-0	1	3	-6.20	0.33	0.61	0.94
N-(Cyclohexylthio)phthalimide	17796-82-6	1	3	-6.17	0.33	0.6	0.94
17-((1-Oxohexyl)oxy)pregn-4-ene-3,20-dione	630-56-8	1	2	-7.16	0.22	0.67	0.89
Pioglitazone hydrochloride	112529-15-4	1	1	-7.97	0.11	0.72	0.84
Troglitazone	97322-87-7	1	1	-7.91	0.11	0.72	0.83
PharmaGSID_48511	1062243-51-9	0	0	-8.91	0.00	0.79	0.79
PharmaGSID_48172	NOCAS_48172	1	1	-7.80	0.11	0.71	0.82
Thiodicarb	59669-26-0	1	4	-4.54	0.44	0.49	0.94
CP-100829	135080-03-4	1	1	-7.78	0.11	0.71	0.82
Zearalenone	17924-92-4	0	1	-7.61	0.11	0.7	0.81
Fabesetron hydrochloride	129299-90-7	0	1	-7.59	0.11	0.7	0.81
Tributyltetradecylphosphonium chloride	81741-28-8	1	3	-5.43	0.33	0.55	0.89
SAR377142	NOCAS_47385	0	0	-8.59	0.00	0.77	0.77
Dinoseb	88-85-7	1	2	-6.26	0.22	0.61	0.83
Bicalutamide	90357-06-5	0	0	-8.25	0.00	0.74	0.74
C.I. Direct Yellow 12	2870-32-8	0	0	-8.18	0.00	0.74	0.74
Nelivaptan	439687-69-1	0	0	-8.15	0.00	0.74	0.74
Progesterone	57-83-0	0	1	-7.05	0.11	0.66	0.77
Methidathion	950-37-8	1	3	-4.82	0.33	0.51	0.85
Biochanin A	491-80-5	0	0	-8.00	0.00	0.73	0.73
Apigenin	520-36-5	0	0	-7.82	0.00	0.71	0.71
CP-283097	171866-31-2	0	0	-7.81	0.00	0.71	0.71
Kaempferol	520-18-3	0	0	-7.72	0.00	0.71	0.71
Quercetin	117-39-5	0	0	-7.69	0.00	0.71	0.71
Diphenylamine	122-39-4	0	2	-5.48	0.22	0.56	0.78
Sodium myristyl sulfate	1191-50-0	1	4	-3.10	0.44	0.4	0.84
Equilin	474-86-2	0	0	-7.42	0.00	0.69	0.69
Octylbicycloheptenedicarboximide	113-48-4	0	1	-6.33	0.11	0.61	0.73
1,4-Dihydroxy-2-naphthoic acid	31519-22-9	0	0	-7.41	0.00	0.69	0.69
Oryzalin	19044-88-3	0	1	-6.28	0.11	0.61	0.72
Flumioxazin	103361-09-7	0	0	-7.31	0.00	0.68	0.68

Flumiclorac-pentyl	87546-18-7	0	1	-6.22	0.11	0.61	0.72
4,4'-Dithiodimorpholine	103-34-4	0	2	-5.06	0.22	0.53	0.75
Tetramethrin	7696-12-0	0	0	-7.18	0.00	0.67	0.67
Thiram	137-26-8	0	2	-4.78	0.22	0.51	0.73
2-tert-Butylphenol	88-18-6	0	0	-6.85	0.00	0.65	0.65
2,2'-(Tetradecylimino)diethanol	18924-66-8	0	1	-5.71	0.11	0.57	0.68
Dicloran	99-30-9	0	0	-6.77	0.00	0.64	0.64
Piperonyl butoxide	51-03-6	0	0	-6.68	0.00	0.64	0.64
Docusate sodium	577-11-7	0	0	-6.64	0.00	0.63	0.63
Methyltrioctylammonium chloride	5137-55-3	0	1	-5.52	0.11	0.56	0.67
Sulfasalazine	599-79-1	0	0	-6.56	0.00	0.63	0.63
Sodium hexyldecyl sulfate	1120-01-0	0	1	-5.44	0.11	0.55	0.67
2-Tert-Butyl-5-methylphenol	88-60-8	0	0	-6.52	0.00	0.63	0.63
Octyl gallate	1034-01-1	0	2	-4.34	0.22	0.48	0.7
Besonprodil	253450-09-8	0	1	-5.37	0.11	0.55	0.66
1,2-Benzenedicarboxaldehyde	643-79-8	0	0	-6.30	0.00	0.61	0.61
2,3-Diaminotoluene	2687-25-4	0	0	-6.30	0.00	0.61	0.61
Fenaminosulf	140-56-7	0	0	-6.28	0.00	0.61	0.61
Cloprop	101-10-0	0	0	-6.27	0.00	0.61	0.61
3-Trifluoromethyl-4-nitrophenol	88-30-2	0	0	-6.19	0.00	0.6	0.6
1,2-Phenylenediamine	95-54-5	0	0	-6.06	0.00	0.6	0.6
Oleyl sarcosine	110-25-8	0	0	-6.05	0.00	0.59	0.59
Didecyldimethylammonium chloride	7173-51-5	0	1	-4.82	0.11	0.51	0.62
2,4-Dinitrophenol	51-28-5	0	0	-5.87	0.00	0.58	0.58
Thidiazuron	51707-55-2	0	0	-5.82	0.00	0.58	0.58
Ethofumesate	26225-79-6	0	0	-5.67	0.00	0.57	0.57
2-Methyl-4,6-dinitrophenol	534-52-1	0	0	-5.58	0.00	0.56	0.56
1-Phenyl-1H-pyrrole-2,5-dione	941-69-5	0	0	-5.53	0.00	0.56	0.56
2,4,5-Trichlorophenol	95-95-4	0	0	-5.53	0.00	0.56	0.56
Dichlone	117-80-6	0	0	-5.45	0.00	0.55	0.55
1,2-Dibromo-2,4-dicyanobutane	35691-65-7	0	0	-5.12	0.00	0.53	0.53
4,5-Dichloro-3H-1,2-dithiol-3-one	1192-52-5	0	0	-5.12	0.00	0.53	0.53
Octhilinone	26530-20-1	0	0	-5.01	0.00	0.52	0.52
Triethylene glycol bis(2-ethylhexanoate)	94-28-0	0	0	-4.98	0.00	0.52	0.52
Riboflavin	83-88-5	0	0	-4.94	0.00	0.52	0.52
1,2-Benzisothiazolin-3-one	2634-33-5	0	0	-4.89	0.00	0.52	0.52
Diisobutyl phthalate	84-69-5	0	1	-3.46	0.11	0.42	0.53
Laurocapram	59227-89-3	0	0	-4.26	0.00	0.47	0.47
Triallyl trimellitate	2694-54-4	0	0	-4.17	0.00	0.47	0.47
1-Dodecyl-2-pyrrolidinone	2687-96-9	0	0	-3.38	0.00	0.42	0.42
Hexadecyltrimethylammonium bromide	57-09-0	0	0	-2.93	0.00	0.39	0.39
Dibutyl phthalate	84-74-2	0	0	-2.69	0.00	0.37	0.37
Myristyltrimethylammonium chloride	4574-04-3	0	0	-2.20	0.00	0.34	0.34
Dodecyltrimethylammonium chloride	112-00-5	0	0	-2.01	0.00	0.32	0.32
8,10-Dodecadien-1-ol	33956-49-9	0	0	-0.84	0.00	0.24	0.24
Triphenyltin hydroxide	76-87-9	NaN	8	NaN	0.89	NaN	NaN
Ziram	137-30-4	NaN	0	NaN	0.00	NaN	NaN
Tributyltin chloride	1461-22-9	NaN	8	NaN	0.89	NaN	NaN
Tetrabutyltin	1461-25-2	NaN	4	NaN	0.44	NaN	NaN
Zinc pyrithione	13463-41-7	NaN	NaN	NaN	NaN	NaN	NaN
Tributyltin benzoate	4342-36-3	NaN	1	NaN	0.11	NaN	NaN
Tributyltin methacrylate	2155-70-6	NaN	1	NaN	0.11	NaN	NaN

Table S6. Predictions for external test set, derived from ChEMBL tested for NIS Inhibition. The table includes the sum of positive random forest predictions, the docking scores, and their respective normalized values. A consensus score was calculated by summing these two normalized values. Binary classification labels in the "Predicted Activity" column were assigned by applying a threshold of 0.82 to the consensus score. The corresponding pChEMBL values from ChEMBL are also provided for reference.

Molecule ChEMBL ID	Compound Key	pChEMBL Value	Predicted Activity	Random Forest Prediction	Docking Score	Normalized Random Forest Prediction	Normalized Docking Score	Consensus Score
CHEMBL3979088	13	8.96	1	9	-8.91	1.00	0.79	1.79
CHEMBL3957527	12	6.7	1	9	-8.58	1.00	0.77	1.77
CHEMBL3951491	4	8.28	1	9	-8.44	1.00	0.76	1.76
CHEMBL3917239	9	6.85	1	9	-8.39	1.00	0.75	1.75
CHEMBL3954902	11	6.77	1	9	-8.14	1.00	0.74	1.74
CHEMBL3905478	6	6.85	1	9	-8.02	1.00	0.73	1.73
CHEMBL3905889	ITB-2; 2	6.52	1	9	-8.01	1.00	0.73	1.73
CHEMBL3940879	7	6.77	1	9	-7.94	1.00	0.72	1.72
CHEMBL3895780	5	6.7	1	9	-7.82	1.00	0.71	1.71
CHEMBL3907206	ITB-1; 1	6.4	1	9	-7.73	1.00	0.71	1.71
CHEMBL3959729	52	7.02	1	6	-9.60	0.67	0.83	1.50
CHEMBL3920002	10	7.24	1	9	-7.29	1.00	0.68	1.68
CHEMBL3943543	8	9.39	1	7	-7.91	0.78	0.72	1.50
CHEMBL3960716	ITB-11	6.4	1	9	-6.21	1.00	0.61	1.61
CHEMBL3957039	51	7.19	1	5	-8.79	0.56	0.78	1.33
CHEMBL3920461	33	6.46	1	7	-6.62	0.78	0.63	1.41
CHEMBL3976525	44	6.02	1	6	-6.84	0.67	0.65	1.31
CHEMBL3905375	36	6.6	1	5	-7.06	0.56	0.66	1.22
CHEMBL3969249	35	6.7	1	5	-7.03	0.56	0.66	1.22
CHEMBL3966686	34	6.52	1	5	-6.95	0.56	0.66	1.21
CHEMBL3945731	48	6.26	1	5	-6.36	0.56	0.62	1.17
CHEMBL2333501	58 & 14	8.19	1	2	-8.47	0.22	0.76	0.98
CHEMBL3897972	45	6.6	1	5	-6.01	0.56	0.59	1.15
CHEMBL3891304	49	6.82	1	3	-7.50	0.33	0.69	1.03
CHEMBL2333516	57 & 11	10.19	1	2	-8.23	0.22	0.74	0.96
CHEMBL3907771	46	6.3	1	3	-6.97	0.33	0.66	0.99
CHEMBL3974084	43	6.07	1	4	-6.11	0.44	0.6	1.04
CHEMBL2333513	54 & 8	8.8	1	1	-8.25	0.11	0.74	0.85
CHEMBL2333504	61 & 17	9.07	0	0	-8.85	0.00	0.78	0.78
CHEMBL2333510	(R)-17	6.2	0	0	-8.85	0.00	0.78	0.78
CHEMBL3893753	42	6.46	1	4	-5.65	0.44	0.57	1.01
CHEMBL3985413	63	7.11	0	0	-8.39	0.00	0.75	0.75
CHEMBL3890967	41	6.26	1	3	-5.93	0.33	0.59	0.92
CHEMBL3943027	47	5.92	0	1	-7.38	0.11	0.68	0.80
CHEMBL3894096	50	7.12	0	0	-8.12	0.00	0.73	0.73
CHEMBL2333509	(S)-17	9.15	0	0	-8.12	0.00	0.73	0.73
CHEMBL3915394	24	7.12	0	1	-7.19	0.11	0.67	0.78
CHEMBL3976335	22	7	0	1	-7.14	0.11	0.67	0.78

CHEMBL277597	32	6.72	1	3	-5.56	0.33	0.56	0.90
CHEMBL1541261	25	6.82	0	1	-7.05	0.11	0.66	0.77
CHEMBL1588586	21	6.46	0	1	-7.00	0.11	0.66	0.77
CHEMBL1702545	23	7.19	0	1	-6.95	0.11	0.66	0.77
CHEMBL3908161	37	7.3	0	0	-7.27	0.00	0.68	0.68
CHEMBL1365550	38	7.1	0	0	-7.25	0.00	0.68	0.68
CHEMBL2333503	60 & 16	7.14	0	0	-7.06	0.00	0.66	0.66
CHEMBL1489246	ITB9 & 1	7.05	0	0	-6.97	0.00	0.66	0.66
CHEMBL2333505	(R)-1	7.16	0	0	-6.97	0.00	0.66	0.66
CHEMBL3950462	31	7.4	0	0	-6.95	0.00	0.66	0.66
CHEMBL3895533	62	7.43	0	0	-6.88	0.00	0.65	0.65
CHEMBL3959979	30	7.3	0	0	-6.83	0.00	0.65	0.65
CHEMBL2333502	59 & 15	8.4	0	0	-6.82	0.00	0.65	0.65
CHEMBL3898213	29	7.05	0	0	-6.66	0.00	0.64	0.64
CHEMBL3900988	28	7.7	0	0	-6.64	0.00	0.63	0.63
CHEMBL2333512	53 & 7	7.05	0	0	-6.63	0.00	0.63	0.63
CHEMBL2333518	13	6	0	1	-5.82	0.11	0.58	0.69
CHEMBL2333511	27 & 6	8.49	0	0	-6.48	0.00	0.62	0.62
CHEMBL2333507	(S)-6	6.2	0	0	-6.48	0.00	0.62	0.62
CHEMBL2333517	12	6.4	0	0	-6.41	0.00	0.62	0.62
CHEMBL2333515	56 & 10	7.12	0	0	-6.38	0.00	0.62	0.62
CHEMBL3952962	40	7.16	0	1	-5.19	0.11	0.54	0.65
CHEMBL3976099	26	6.12	0	0	-5.61	0.00	0.57	0.57
CHEMBL2333506	(S)-1	5.82	0	0	-4.94	0.00	0.52	0.52
CHEMBL2333508	(R)-6	4.85	0	0	-4.84	0.00	0.51	0.51
CHEMBL2333514	55 & 9	9.24	0	0	-4.63	0.00	0.5	0.50
CHEMBL84336	SCN-	4.85	0	0	-2.86	0.00	0.38	0.38
CHEMBL1789400	BF4-	6.12	NaN	NaN	-4.44	NaN	0.49	NaN
CHEMBL1644700	Sodium perchlorate	7	NaN	NaN	-3.32	NaN	0.41	NaN
CHEMBL3327018	PF6-	8.05	NaN	NaN	-3.80	NaN	0.44	NaN
CHEMBL186200	NO3-	NaN	NaN	NaN	-3.06	NaN	0.39	NaN
CHEMBL1161634	ClO4-	6.85	NaN	NaN	-2.81	NaN	0.38	NaN

Selected parameters in machine learning

Baseline models

For models trained on ECFP4 fingerprints, the selected parameters were:

- *RF:* max_depth = None, n_estimators = 200
- *SVM:* C = 1, kernel = linear
- *XGB:* max_depth = 7, n_estimators = 200

For models trained on CDDD representations, the selected parameters were:

- *RF:* max_depth = None, n_estimators = 100

- *SVM:* C = 10, kernel = rbf
- *XGB:* max_depth = 3, n_estimators = 200

Nested cross validation with undersampling and hyperparameter tuning

The selected parameters for the models trained on ECFP4 fingerprints were:

- *RF:* max_depth = 20, max_depth = 50
- *SVM:* C = 10, kernel = rbf
- *XGB:* max_depth = 3, n_estimators = 100

For the models trained on CDDD representations the following parameters were used:

- *RF:* max_depth = 20, C = 200
- *SVM:* C = 10, kernel = rbf
- *XGB:* max_depth = 3, n_estimators = 100

Nearest neighbors of predicted cytotoxic compounds

Five nearest neighbors for each compound tested for NIS inhibition by Wang et al.[23], that showed decreased iodide uptake but also exhibited cytotoxicity. Nearest neighbors were identified from the training set based on Tanimoto coefficients calculated using ECFP4 fingerprints.

Query Compound: 4,4'-Methylenebis(2,6-diethylaniline)

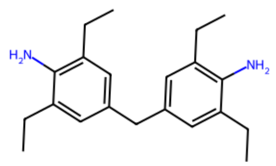

Predicted Activity: 1.0, Votes: 9.0, Docking Score: -9.62

Name	Hit2	Structure	Tanimoto Index
2,6-Diethylaniline	0	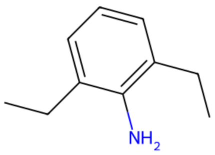	0.500
2-Ethyl-6-methylaniline	0	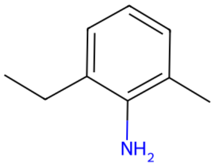	0.379
4,4'-Methylenebis(o-toluidine)	0	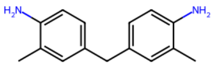	0.345
4,4'-Diaminobiphenyl methane	0	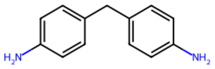	0.320
4,4'-Methylenebis(2,6-di-t-butylphenol)	0	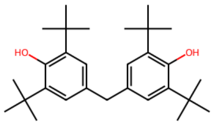	0.300

Query Compound: 4,4'-Methylenebis(N,N-dimethylaniline)

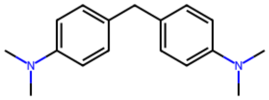

Predicted Activity: 1.0, Votes: 9.0, Docking Score: -9.08

Name	Hit2	Structure	Tanimoto Index
N,N,4-Trimethylaniline	0	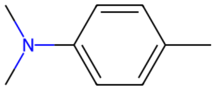	0.524
N,N-Dimethylaniline	0	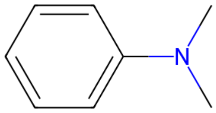	0.476
4,4'-Diaminobiphenyl methane	0	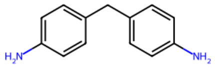	0.409
Diphenylmethane	0	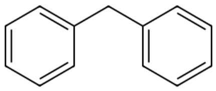	0.381

4,4'-Methylenebis(o-toluidine)	0	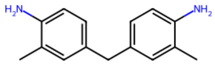	0.321

Query Compound: Amiodarone hydrochloride

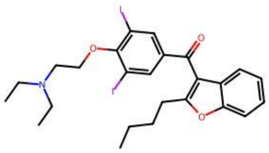

Predicted Activity: 1.0, Votes: 9.0, Docking Score: -9.02

Name	Hit2	Structure	Tanimoto Index
Procaine hydrochloride	0	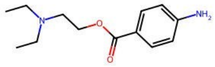	0.292
Octabenzone	0	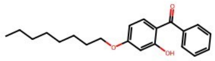	0.282
Butyl benzoate	0	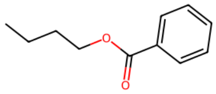	0.274

Hexyl benzoate	0	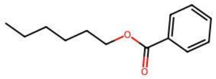	0.266
Dipentyl phthalate	0	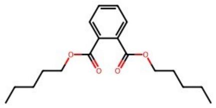	0.254

Query Compound: C.I. Solvent Yellow 14

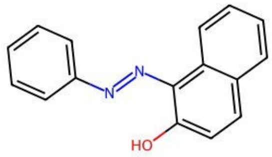

Predicted Activity: 1.0, Votes: 9.0, Docking Score: -8.71

Name	Hit2	Structure	Tanimoto Index
C.I. Acid Orange 7	0	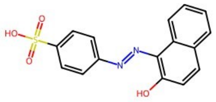	0.676
C.I. Acid Orange 8, monosodium salt	0	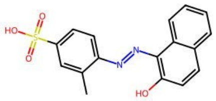	0.478

FD&C; Yellow 6	0	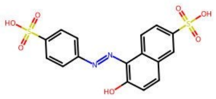	0.476
C.I. Acid Orange 10	0	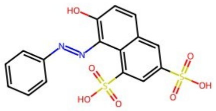	0.467
Azobenzene	0	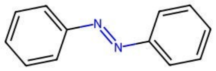	0.444

Query Compound: Trifloxystrobin

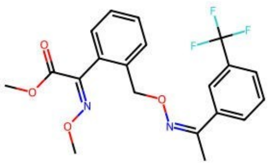

Predicted Activity: 1.0, Votes: 9.0, Docking Score: -8.63

Name	Hit2	Structure	Tanimoto Index
Kresoxim-methyl	1	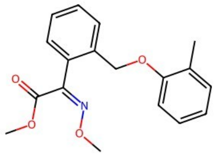	0.492

Fluometuron	0	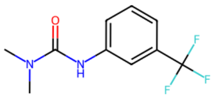	0.302
Flutolanil	1	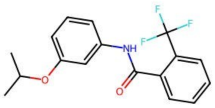	0.286
Dimethyl isophthalate	0	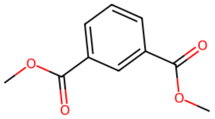	0.268
Dimethyl phthalate	0	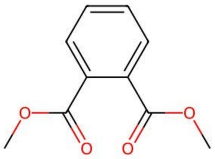	0.255

Query Compound: SR125047

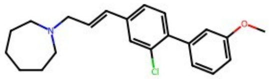

Predicted Activity: 1.0, Votes: 9.0, Docking Score: -8.54

Name	Hit2	Structure	Tanimoto Index

(E)-Anethole	0	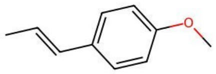	0.245
4-Methoxybenzaldehyde	0	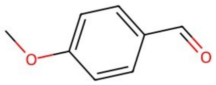	0.226
Isoeugenol	0	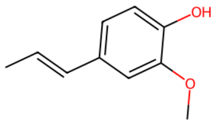	0.220
Raloxifene hydrochloride	0	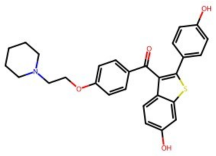	0.213
Spirodiclofen	0	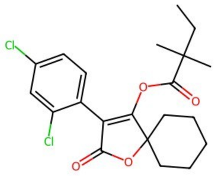	0.213

Query Compound: 4-Hydroxytamoxifen

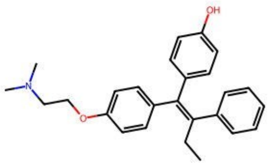

Predicted Activity: 1.0, Votes: 9.0, Docking Score: -8.49

Name	Hit2	Structure	Tanimoto Index
4-(Hexyloxy)phenol	0	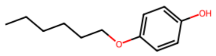	0.400
Octabenzone	0	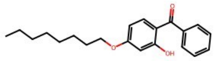	0.351
Diphenhydramine hydrochloride	0	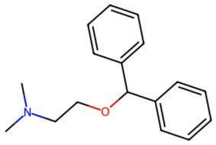	0.333
Butylparaben	0	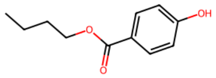	0.320
2-Phenoxyethanol	0	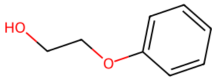	0.318

Query Compound: Elzasonan

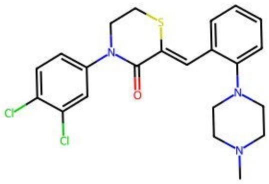

Predicted Activity: 1.0, Votes: 9.0, Docking Score: -8.4

Name	Hit2	Structure	Tanimoto Index
3,4-Dichloroaniline	0	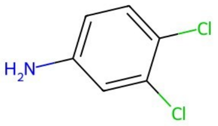	0.222
Benoxacor	0	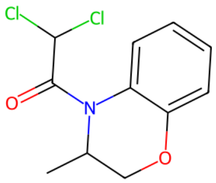	0.214
C.I. Acid Yellow 34, monosodium salt	0	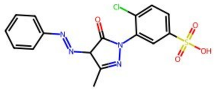	0.210
Chlorpromazine hydrochloride	1	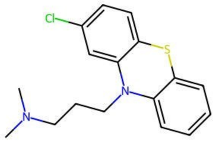	0.205
Clomazone	0	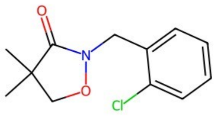	0.200

Query Compound: Clomiphene citrate (1:1)

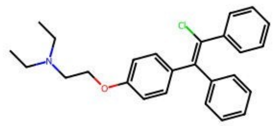

Predicted Activity: 1.0, Votes: 9.0, Docking Score: -8.34

Name	Hit2	Structure	Tanimoto Index
Procaine hydrochloride	0	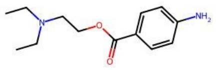	0.367
1,2-Diphenoxyethane	0	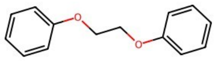	0.342
2-Phenoxyethanol	0	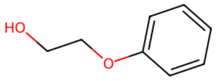	0.341
Octabenzone	0	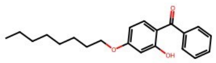	0.321
Butyl benzoate	0	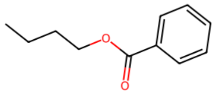	0.319

Query Compound: UK-337312

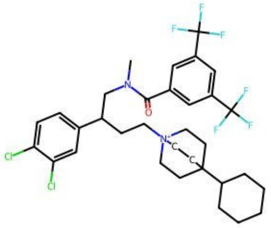

Predicted Activity: 1.0, Votes: 7.0, Docking Score: -10.44

Name	Hit2	Structure	Tanimoto Index
1,2-Dichloro-4-(trifluoromethyl)benzene	0	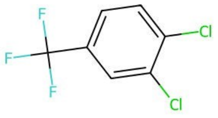	0.239
SSR146977	0	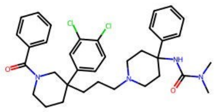	0.221
CP-532623	0	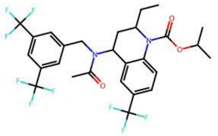	0.218
PharmaGSID_48513	0	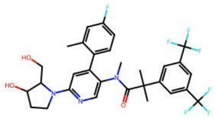	0.215

Diuron	0	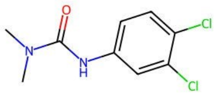	0.213

Query Compound: 2,2'-Methylenebis(4-methyl-6-tert-butylphenol)

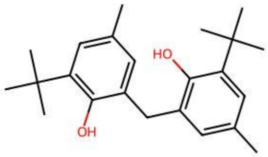

Predicted Activity: 1.0, Votes: 9.0, Docking Score: -8.26

Name	Hit2	Structure	Tanimoto Index
4,4'-Methylenebis(2,6-di-t-butylphenol)	0	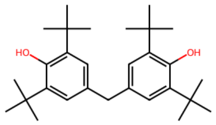	0.517
2,6-Di-tert-butyl-4-ethylphenol	1	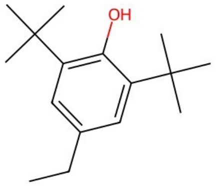	0.452
4-Methyl-2-tert-butylphenol	0	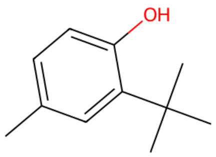	0.452

2,4,6-Tris(tert-butyl)phenol	0	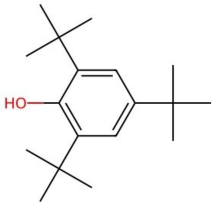	0.448
2,6-Di-tert-butyl-4-methoxyphenol	1	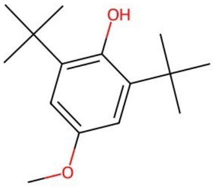	0.406

Query Compound: Famoxadone

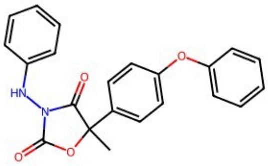

Predicted Activity: 1.0, Votes: 9.0, Docking Score: -8.18

Name	Hit2	Structure	Tanimoto Index
Diphenyl oxide	0	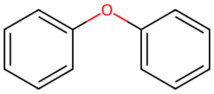	0.308
3-Phenoxybenzoic acid	0	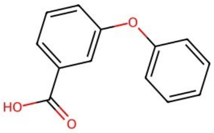	0.260

Fenpropathrin	0	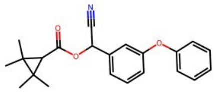	0.246
Ethyl methylphenylglycidate	0	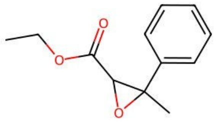	0.246
Fenuron	0	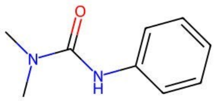	0.245

Query Compound: Chlorophacinone

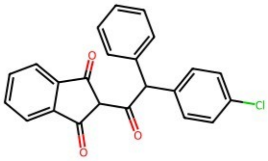

Predicted Activity: 1.0, Votes: 9.0, Docking Score: -8.12

Name	Hit2	Structure	Tanimoto Index
Diphenamid	0	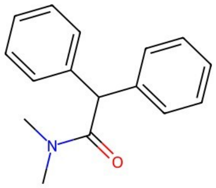	0.385

Esfenvalerate	0	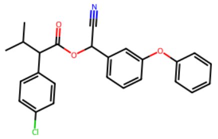	0.295
Halofenozide	0	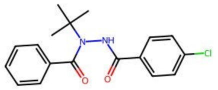	0.294
Benzoin	0	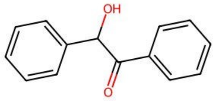	0.293
5-Chlorosalicylanilide	1	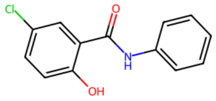	0.292

Query Compound: 2,2'-Methylenebis(ethyl-6-tert-butylphenol)

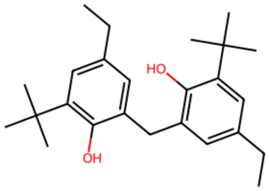

Predicted Activity: 1.0, Votes: 9.0, Docking Score: -8.06

Name	Hit2	Structure	Tanimoto Index

2,6-Di-tert-butyl-4-ethylphenol	1	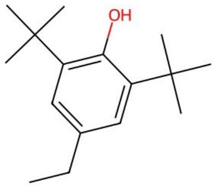	0.741
4,4'-Methylenebis(2,6-di-t-butylphenol)	0	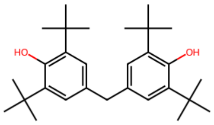	0.586
2,4,6-Tris(tert-butyl)phenol	0	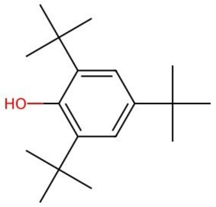	0.419
2,6-Di-tert-butyl-4-methoxyphenol	1	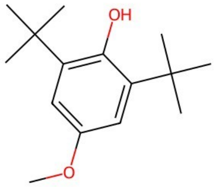	0.382
Irganox 1010	0	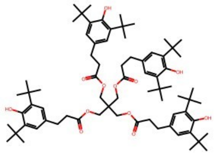	0.381

Query Compound:

1-(2-Chlorophenyl)-N-methyl-N-(1-methylpropyl)-3-isoquinolinecarboxamide

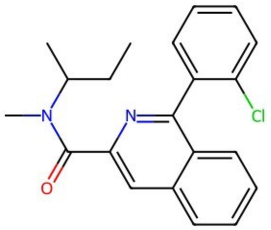

Predicted Activity: 1.0, Votes: 9.0, Docking Score: -8.06

Name	Hit2	Structure	Tanimoto Index
Napropamide	0	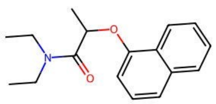	0.290
Methadone hydrochloride	0	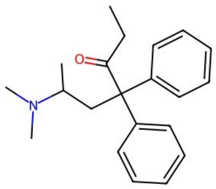	0.262
Clofentezine	0	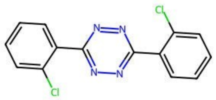	0.260
Ethyl 1-naphthaleneacetate	1	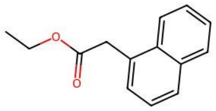	0.250
Imazaquin	0	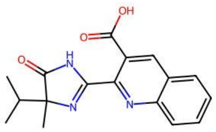	0.243

Query Compound: Difenoconazole

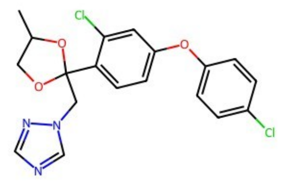

Predicted Activity: 1.0, Votes: 9.0, Docking Score: -8.05

Name	Hit2	Structure	Tanimoto Index
Triticonazole	0	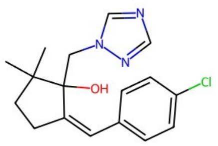	0.347
Cyproconazole	0	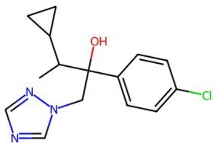	0.343
Tebuconazole	0	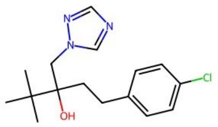	0.304
Myclobutanil	0	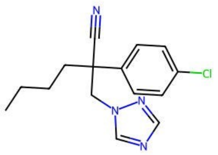	0.288
Triadimenol	0	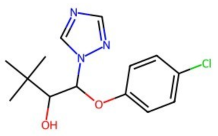	0.286

Query Compound: Celecoxib

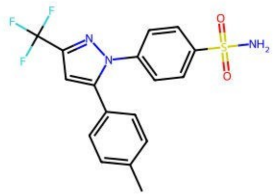

Predicted Activity: 1.0, Votes: 9.0, Docking Score: -8.01

Name	Hit2	Structure	Tanimoto Index
4-Toluenesulfonamide	0	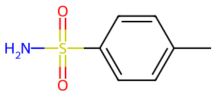	0.450
Difpas-pyrazole	0	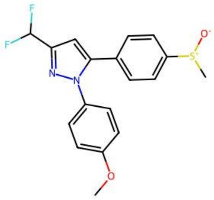	0.317
Sodium 4-methylbenzenesulfonate	0	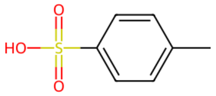	0.289
Chloramine-T trihydrate	0	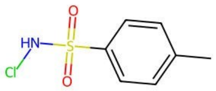	0.271

N-Butyl-p-toluenesulfonamide	0	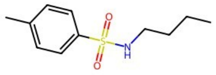	0.241

Query Compound: Benfluralin

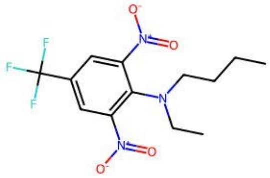

Predicted Activity: 1.0, Votes: 9.0, Docking Score: -7.91

Name	Hit2	Structure	Tanimoto Index
Trifluralin	1	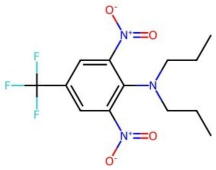	0.744
Flumetralin	1	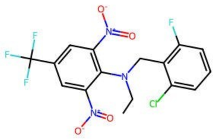	0.547
Isopropalin	0	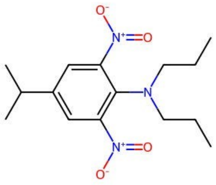	0.457

Oxyfluorfen	1	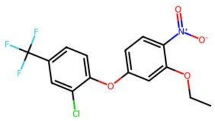	0.328
5,5-Dimethyl-3-(alpha,alpha,alpha-trifluoro-4-nitro-m-tolyl)hydantoin	0	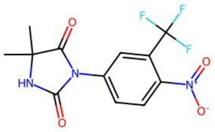	0.288

Query Compound: C.I. Disperse Orange 37

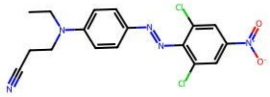

Predicted Activity: 1.0, Votes: 9.0, Docking Score: -7.85

Name	Hit2	Structure	Tanimoto Index
3,4-Dichloronitrobenzene	0	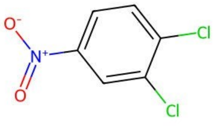	0.308
Flumetralin	1	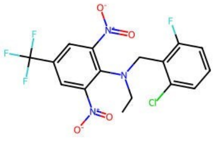	0.274

1-Chloro-2,4-dinitrobenzene	0	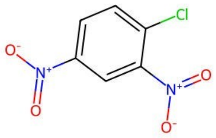	0.273
1-Chloro-4-nitrobenzene	0	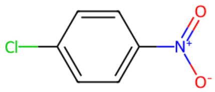	0.250
Isopropalin	0	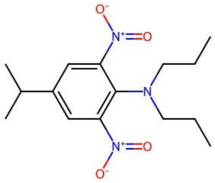	0.238

Query Compound: Pyriproxyfen

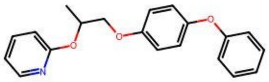

Predicted Activity: 1.0, Votes: 9.0, Docking Score: -7.83

Name	Hit2	Structure	Tanimoto Index
1-Phenoxy-2-propanol	0	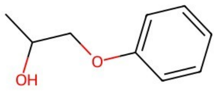	0.436

Diphenyl oxide	0	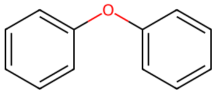	0.353
1,2-Diphenoxyethane	0	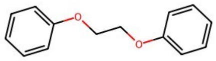	0.324
Etofenprox	0	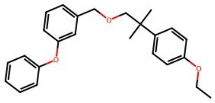	0.298
2-Phenoxyethanol	0	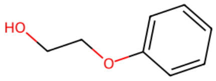	0.293

Query Compound: N-Phenyl-1-naphthylamine

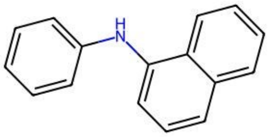

Predicted Activity: 1.0, Votes: 9.0, Docking Score: -7.83

Name	Hit2	Structure	Tanimoto Index

1-Naphthol	0	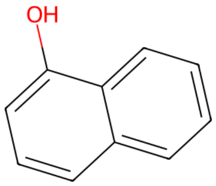	0.393
1-Methylnaphthalene	0	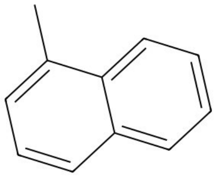	0.393
N-Phenyl-1,4-benzenediamine	0	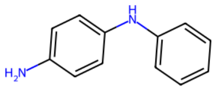	0.379
Pyrimethanil	0	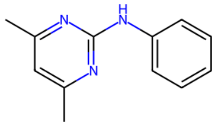	0.364
1,2-Diphenylhydrazine	0	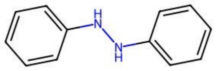	0.360

Query Compound: Auramine hydrochloride

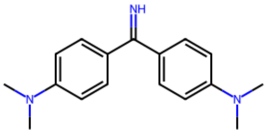

Predicted Activity: 1.0, Votes: 8.0, Docking Score: -8.91

Name	Hit2	Structure	Tanimoto Index
N,N,4-Trimethylaniline	0	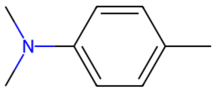	0.478
N,N-Dimethylaniline	0	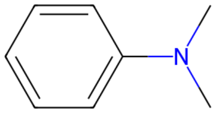	0.435
3-Dimethylaminophenol	0	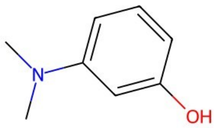	0.345
2-Ethylhexyl 4-(dimethylamino)benzoate	0	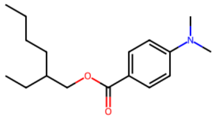	0.333
Methyl red	0	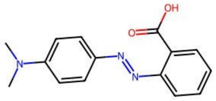	0.308

Query Compound: Cyhalofop-butyl

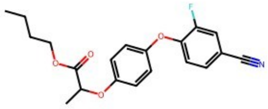

Predicted Activity: 1.0, Votes: 9.0, Docking Score: -7.83

Name	Hit2	Structure	Tanimoto Index
Clodinafop-propargyl	1	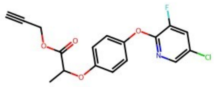	0.403
Butyl lactate	0	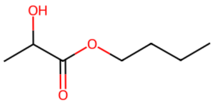	0.333
2,4-D Butyl ester	0	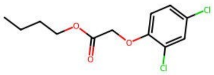	0.317
Haloxyfop-methyl	0	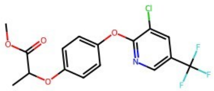	0.314
Butyl benzoate	0	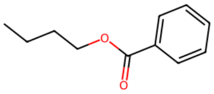	0.304

Query Compound: Epoxiconazole

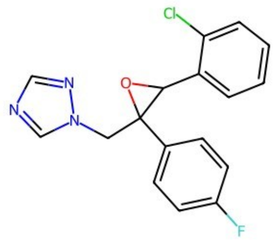

Predicted Activity: 1.0, Votes: 9.0, Docking Score: -7.75

Name	Hit2	Structure	Tanimoto Index
Fluconazole	0	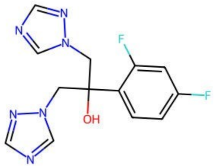	0.317
Triticonazole	0	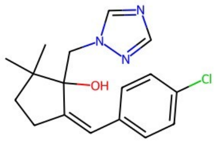	0.309
Cyproconazole	0	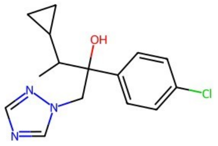	0.303
Tebuconazole	0	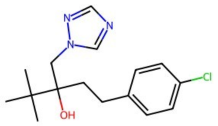	0.281
Myclobutanil	0	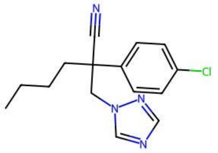	0.265

Query Compound: Diethylstilbestrol

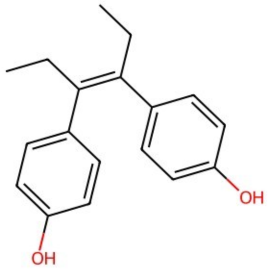

Predicted Activity: 1.0, Votes: 9.0, Docking Score: -7.68

Name	Hit2	Structure	Tanimoto Index
4-Ethylphenol	0	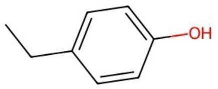	0.478
Ethylparaben	0	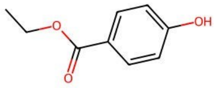	0.464
4-Hydroxyacetophenone	0	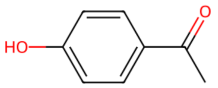	0.458
Hydroquinone	0	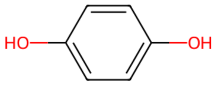	0.444

4-Propylphenol	0	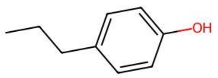	0.440

Query Compound: Pyridaben

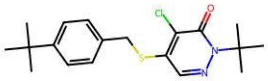

Predicted Activity: 1.0, Votes: 9.0, Docking Score: -7.62

Name	Hit2	Structure	Tanimoto Index
Tebufenpyrad	1	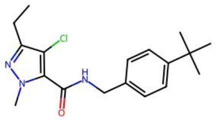	0.317
2-(4-Tert-Butylbenzyl)propionaldehyde	0	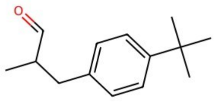	0.294
4,4'-Di-tert-butylbiphenyl	0	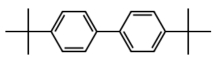	0.256

4-tert-Butyltoluene	0	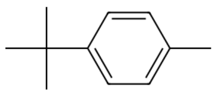	0.250
Norflurazon	0	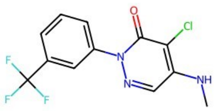	0.246

Query Compound: Tamoxifen citrate

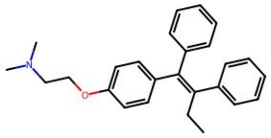

Predicted Activity: 1.0, Votes: 9.0, Docking Score: -7.6

Name	Hit2	Structure	Tanimoto Index
Diphenhydramine hydrochloride	0	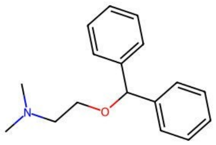	0.364
1,2-Diphenoxyethane	0	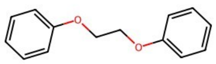	0.351

2-Phenoxyethanol	0	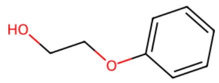	0.350
Octabenzone	0	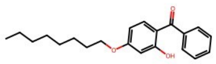	0.327
Butyl benzoate	0	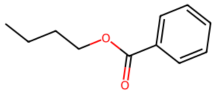	0.326

Query Compound: Gentian Violet

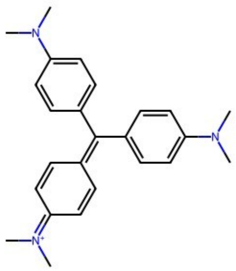

Predicted Activity: 1.0, Votes: 9.0, Docking Score: -7.58

Name	Hit2	Structure	Tanimoto Index
N,N,4-Trimethylaniline	0	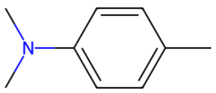	0.355

Basic Blue 7	1	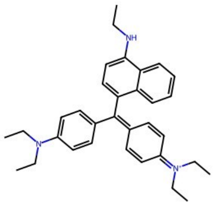	0.339
N,N-Dimethylaniline	0	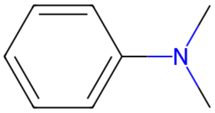	0.323
Sulfan blue	0	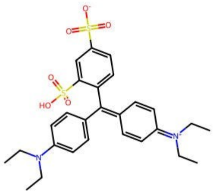	0.322
2-Ethylhexyl 4-(dimethylamino)benzoate	0	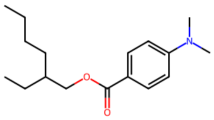	0.280

Query Compound: Fenamidone

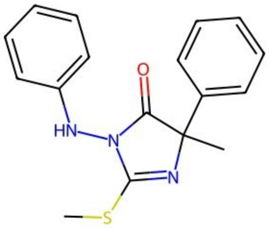

Predicted Activity: 1.0, Votes: 9.0, Docking Score: -7.53

Name	Hit2	Structure	Tanimoto Index

Phenobarbital sodium	0	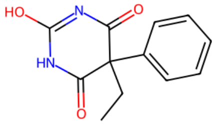	0.236
Fenuron	0	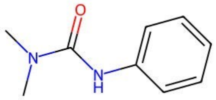	0.234
5,5-Diphenylhydantoin	0	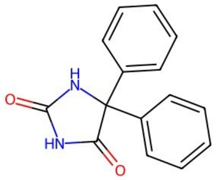	0.234
N-Ethylaniline	0	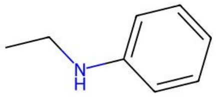	0.233
1,2-Diphenylhydrazine	0	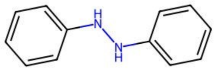	0.231

Query Compound: 2-tert-Butyl-4-ethylphenol

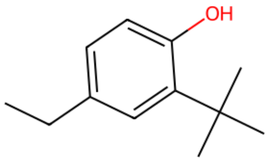

Predicted Activity: 1.0, Votes: 9.0, Docking Score: -7.52

Name	Hit2	Structure	Tanimoto Index
2,6-Di-tert-butyl-4-ethylphenol	1	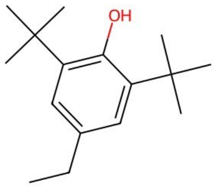	0.607
tert-Butylhydroquinone	0	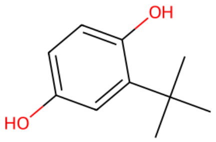	0.571
2,4-Di-tert-butylphenol	1	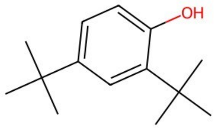	0.552
4-Methyl-2-tert-butylphenol	0	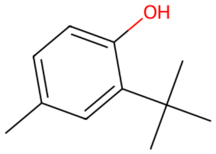	0.552
2-tert-Butyl-4-methoxyphenol	1	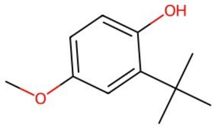	0.500

Query Compound: HMR1426

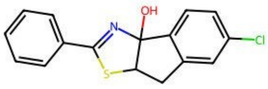

Predicted Activity: 1.0, Votes: 9.0, Docking Score: -7.5

Name	Hit2	Structure	Tanimoto Index
Oxazepam	0	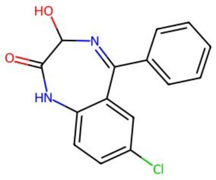	0.322
Clorophene	1	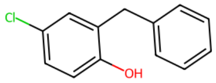	0.245
5-Chlorosalicylanilide	1	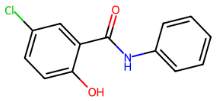	0.228
Cyclanilide	0	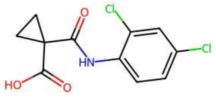	0.220
2,5-Dichlorophenol	0	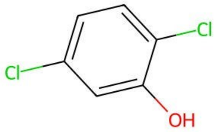	0.213

Query Compound: Pyraclostrobin

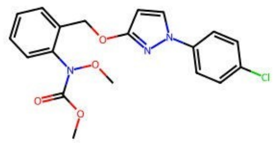

Predicted Activity: 1.0, Votes: 9.0, Docking Score: -7.43

Name	Hit2	Structure	Tanimoto Index
Kresoxim-methyl	1	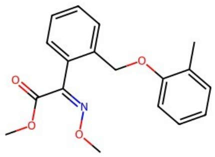	0.250
4-Chlorophenoxyacetic acid	0	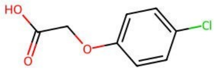	0.237
Halofenozide	0	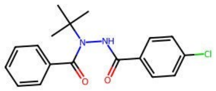	0.235
Monuron	0	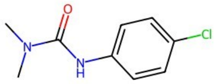	0.233
Ethyl 4-chlorophenyl ketone	0	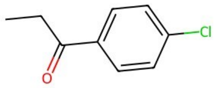	0.224

Query Compound: meso-Hexestrol

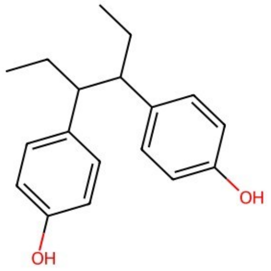

Predicted Activity: 1.0, Votes: 9.0, Docking Score: -7.4

Name	Hit2	Structure	Tanimoto Index
4-(Butan-2-yl)phenol	1	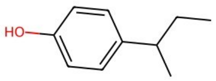	0.625
4-Isopropylphenol	0	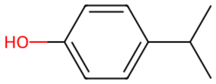	0.522
4-Ethylphenol	0	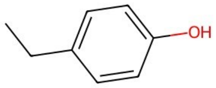	0.458
4-Propylphenol	0	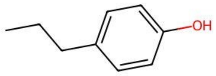	0.423

Hydroquinone	0	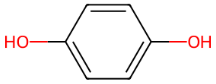	0.421

Query Compound: (2R,6S)-Fenpropimorph

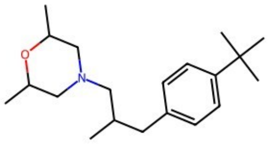

Predicted Activity: 1.0, Votes: 9.0, Docking Score: -7.39

Name	Hit2	Structure	Tanimoto Index
2-(4-Tert-Butylbenzyl)propionaldehyde	0	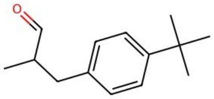	0.409
Tridemorph	0	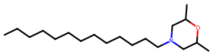	0.304
4,4'-Di-tert-butylbiphenyl	0	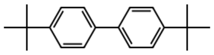	0.282

4-tert-Butyltoluene	0	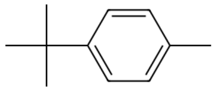	0.275
4-tert-Butylbenzenethiol	0	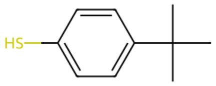	0.268

Query Compound: Fluazinam

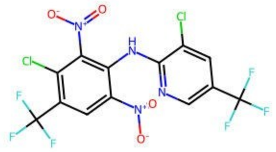

Predicted Activity: 1.0, Votes: 9.0, Docking Score: -7.39

Name	Hit2	Structure	Tanimoto Index
Oxyfluorfen	1	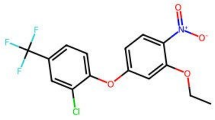	0.324
Acifluorfen	0	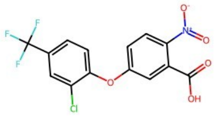	0.309

Fomesafen	0	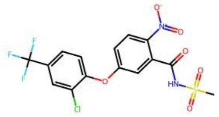	0.293
Haloxyfop-methyl	0	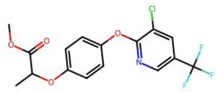	0.286
Trifluralin	1	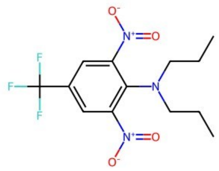	0.283

Query Compound: 4-Cumylphenol

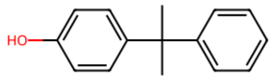

Predicted Activity: 1.0, Votes: 9.0, Docking Score: -7.36

Name	Hit2	Structure	Tanimoto Index
Bisphenol A	1	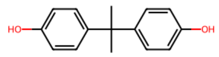	0.800

2,4-Bis(1-methyl-1-phenylethyl)pheno l	1	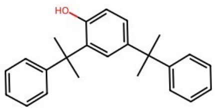	0.586
4-(2-Phenylpropan-2-yl)-N-[4-(2-phen ylpropan-2-yl)phenyl]aniline	0	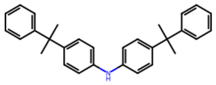	0.577
4-tert-Butylphenol	0	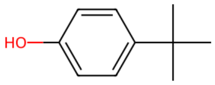	0.565
4,4',4''-Ethane-1,1,1-triyltriphenol	0	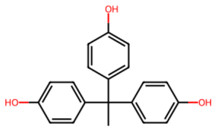	0.565

Query Compound: Hexaconazole

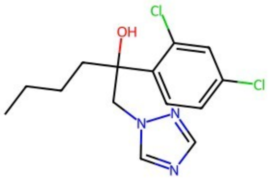

Predicted Activity: 1.0, Votes: 9.0, Docking Score: -7.36

Name	Hit2	Structure	Tanimoto Index

Myclobutanil	0	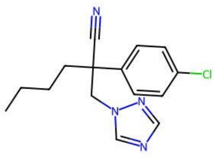	0.518
Tebuconazole	0	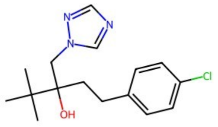	0.500
Fluconazole	0	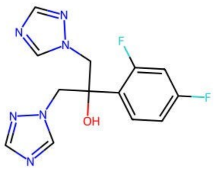	0.472
Cyproconazole	0	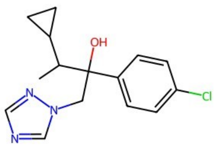	0.417
Triticonazole	0	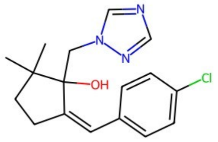	0.313

Query Compound: Quinoxyfen

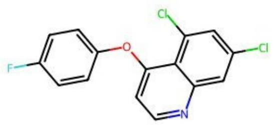

Predicted Activity: 1.0, Votes: 9.0, Docking Score: -7.35

Name	Hit2	Structure	Tanimoto Index
CP-607366	0	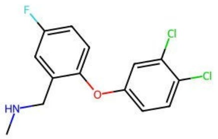	0.315
Triclosan	1	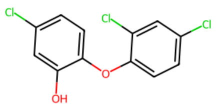	0.255
CP-457677	0	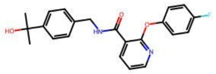	0.254
SB281832	0	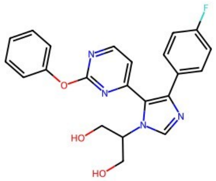	0.250
Clodinafop-propargyl	1	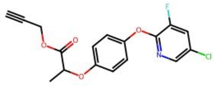	0.242

Query Compound: Rhodamine B

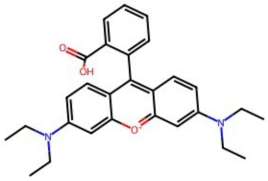

Predicted Activity: 1.0, Votes: 8.0, Docking Score: -8.37

Name	Hit2	Structure	Tanimoto Index
Rhodamine 6G	1	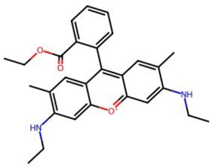	0.417
N,N-Diethylaniline	0	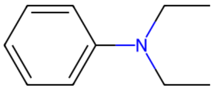	0.357
Monopotassium phthalate	0	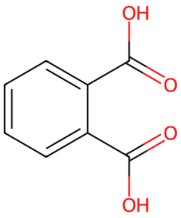	0.317
Methyl red	0	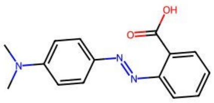	0.309
Monobutyl phthalate	0	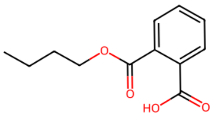	0.302

Query Compound: Butylated hydroxytoluene

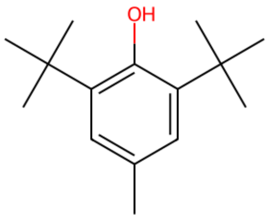

Predicted Activity: 1.0, Votes: 9.0, Docking Score: -7.23

Name	Hit2	Structure	Tanimoto Index
2,4,6-Tris(tert-butyl)phenol	0	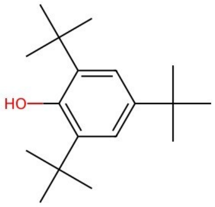	0.636
4,4'-Methylenebis(2,6-di-t-butylphenol)	0	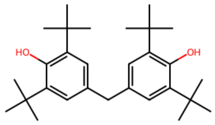	0.583
4-Methyl-2-tert-butylphenol	0	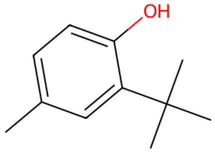	0.560
2,6-Di-tert-butyl-4-methoxyphenol	1	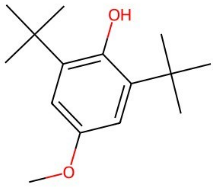	0.560
2,6-Di-tert-butyl-4-ethylphenol	1	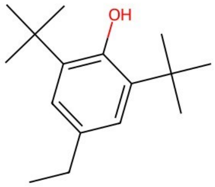	0.560

Query Compound: Triphenyl phosphate

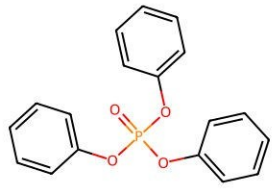

Predicted Activity: 1.0, Votes: 9.0, Docking Score: -7.23

Name	Hit2	Structure	Tanimoto Index
Diphenyl phosphate	0	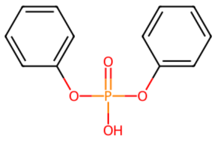	0.636
Diphenyl oxide	0	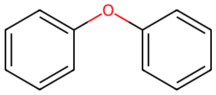	0.450
2-Ethylhexyl diphenyl phosphate	0	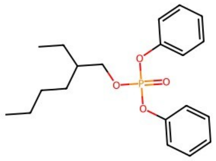	0.444
Diphenyl phosphite	0	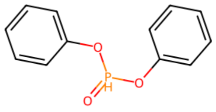	0.417

Triphenyl phosphite	0	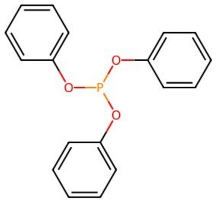	0.391

Query Compound: Benzyl salicylate

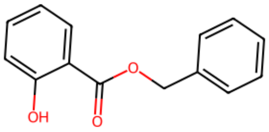

Predicted Activity: 1.0, Votes: 9.0, Docking Score: -7.22

Name	Hit2	Structure	Tanimoto Index
Monobenzyl phthalate	0	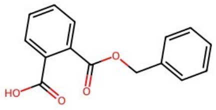	0.710
Benzyl benzoate	1	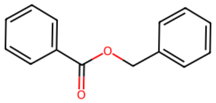	0.613
Phenyl salicylate	1	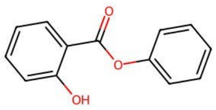	0.545

Benzyl acetate	0	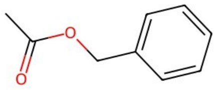	0.531
Methyl salicylate	0	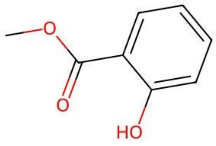	0.531

Query Compound: Dicumyl peroxide

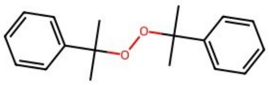

Predicted Activity: 1.0, Votes: 9.0, Docking Score: -7.2

Name	Hit2	Structure	Tanimoto Index
Cumene hydroperoxide	0	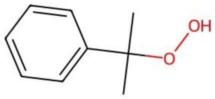	0.636
tert-Butylbenzene	0	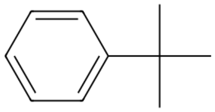	0.550

Benzotrichloride	0	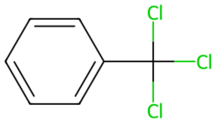	0.409
4-(2-Phenylpropan-2-yl)-N-[4-(2-phenylpropan-2-yl)phenyl]aniline	0	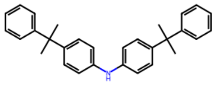	0.407
tert-Butyl perbenzoate	0	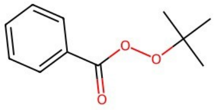	0.367

Query Compound: Benzyl 4-hydroxybenzoate

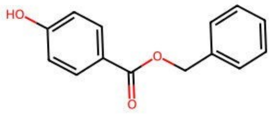

Predicted Activity: 1.0, Votes: 9.0, Docking Score: -7.18

Name	Hit2	Structure	Tanimoto Index
Benzyl benzoate	1	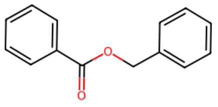	0.815

Ethylparaben	0	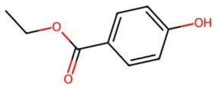	0.581
Monobenzyl phthalate	0	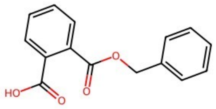	0.576
Benzyl acetate	0	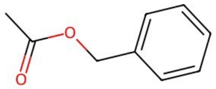	0.548
Propylparaben	0	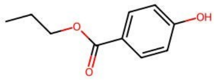	0.545

Query Compound: 2-(2,6-Diisopropylphenyl)-5-hydroxy-1H-isoindole-1,3-dione

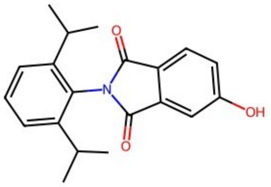

Predicted Activity: 1.0, Votes: 9.0, Docking Score: -7.14

Name	Hit2	Structure	Tanimoto Index

Propofol	1	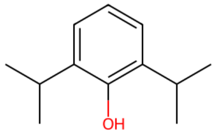	0.389
3-Isopropylphenol	0	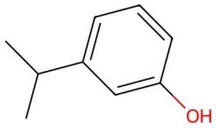	0.359
2-Isopropylphenol	0	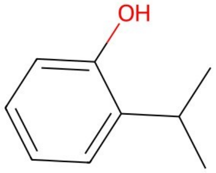	0.342
3-Dimethylaminophenol	0	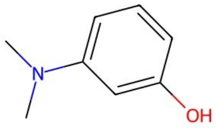	0.293
4-Isopropylphenol	0	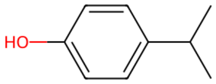	0.289

Query Compound: 2-(Thiocyanomethylthio)benzothiazole

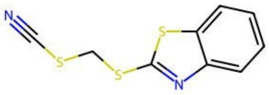

Predicted Activity: 1.0, Votes: 9.0, Docking Score: -7.12

Name	Hit2	Structure	Tanimoto Index
2-Mercaptobenzothiazole	0	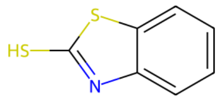	0.395
Methylene bis(thiocyanate)	1	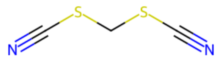	0.303
Benzothiazole	0	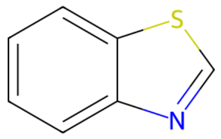	0.275
Dibenzothiophene	1	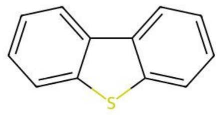	0.243
Quinoline	0	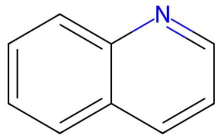	0.225

Query Compound: 1-(2-Chlorophenyl)-1-(4-chlorophenyl)-2,2-dichloroethane

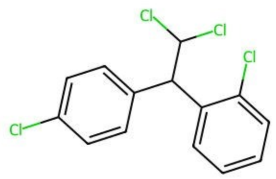

Predicted Activity: 1.0, Votes: 9.0, Docking Score: -7.11

Name	Hit2	Structure	Tanimoto Index
o,p'-DDT	0	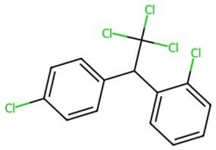	0.545
1,2-Dichlorobenzene	0	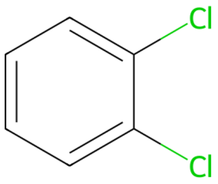	0.346
1,4-Dichlorobenzene	0	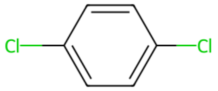	0.320
Benzal chloride	0	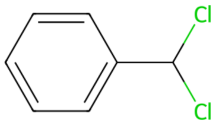	0.300
4-Chlorobenzotrichloride	0	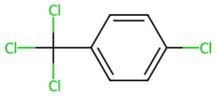	0.290

Query Compound: 2-Chloro-4-phenylphenol

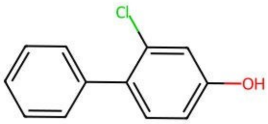

Predicted Activity: 1.0, Votes: 9.0, Docking Score: -7.08

Name	Hit2	Structure	Tanimoto Index
4-Phenylphenol	0	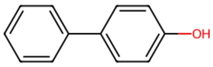	0.462
2-Phenylphenol	0	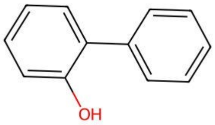	0.429
4-Chloro-3-methylphenol	0	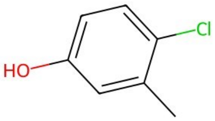	0.400
2-Naphthalenol	0	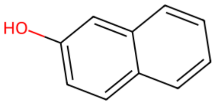	0.379
Phenol	0	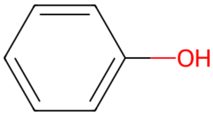	0.360

Query Compound: 7,12-Dimethylbenz(a)anthracene

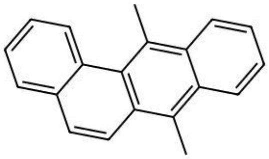

Predicted Activity: 1.0, Votes: 8.0, Docking Score: -8.13

Name	Hit2	Structure	Tanimoto Index
1-Methylnaphthalene	0	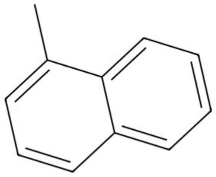	0.444
Dibenz(a,h)anthracene	0	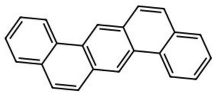	0.407
1,4-Dimethylnaphthalene	0	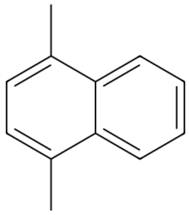	0.346
1-Naphthol	0	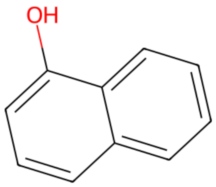	0.345

Pyrene	0	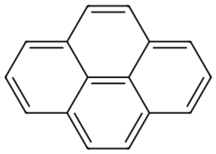	0.320

Query Compound: Fluazifop-butyl

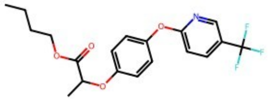

Predicted Activity: 1.0, Votes: 9.0, Docking Score: -7.04

Name	Hit2	Structure	Tanimoto Index
Haloxyfop-methyl	0	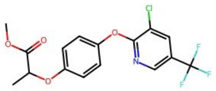	0.492
Clodinafop-propargyl	1	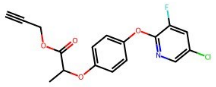	0.388
Butyl lactate	0	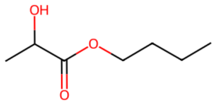	0.340

Lactofen	1	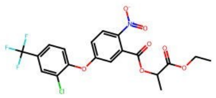	0.321
Butyl benzoate	0	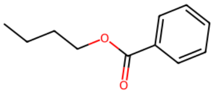	0.309

Query Compound: Celestolide

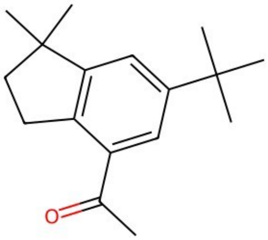

Predicted Activity: 1.0, Votes: 8.0, Docking Score: -8.1

Name	Hit2	Structure	Tanimoto Index
2,4,6-Tris(tert-butyl)phenol	0	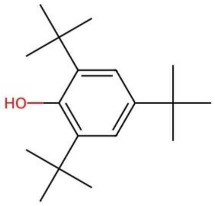	0.282
4-(2,6,6-Trimethyl-cyclohex-1-enyl)-but-3-en-2-one	0	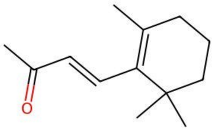	0.245

2-Hydroxyacetophenone	0	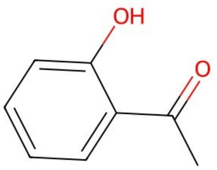	0.244
3-tert-Butylphenol	0	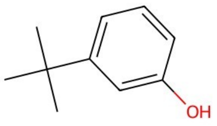	0.238
alpha-Isomethylionone	0	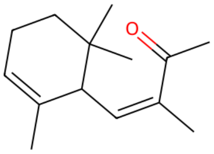	0.235

Query Compound: 2,2-Bis(4-hydroxyphenyl)-1,1,1-trichloroethane

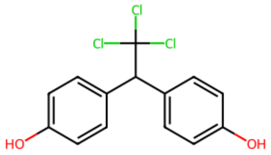

Predicted Activity: 1.0, Votes: 9.0, Docking Score: -7.01

Name	Hit2	Structure	Tanimoto Index
Methoxychlor	1	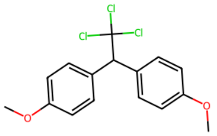	0.519

4-Isopropylphenol	0	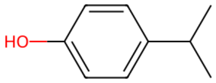	0.458
Hydroquinone	0	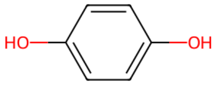	0.421
o,p'-DDT	0	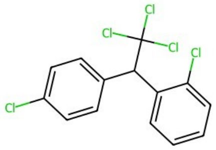	0.406
4-(Butan-2-yl)phenol	1	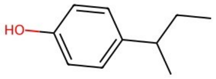	0.393

Query Compound: Fenofibrate

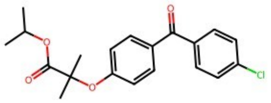

Predicted Activity: 1.0, Votes: 9.0, Docking Score: -7.01

Name	Hit2	Structure	Tanimoto Index

Clofibrate	0	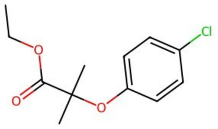	0.548
Triadimefon	0	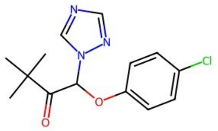	0.345
Ethyl 4-chlorophenyl ketone	0	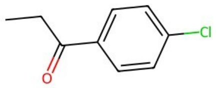	0.333
Chlorpropham	1	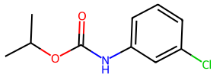	0.320
4-Chlorophenoxyacetic acid	0	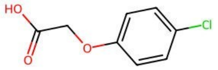	0.318

Query Compound: 2,6-Di-tert-butylphenol

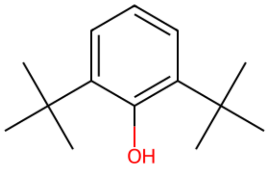

Predicted Activity: 1.0, Votes: 9.0, Docking Score: -7.0

Name	Hit2	Structure	Tanimoto Index
2,4,6-Tris(tert-butyl)phenol	0	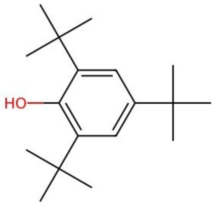	0.522
4,4'-Methylenebis(2,6-di-t-butylphenol)	0	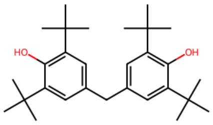	0.480
2,6-Di-tert-butyl-4-ethylphenol	1	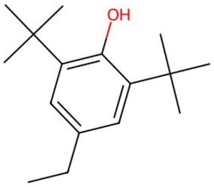	0.462
2,6-Di-tert-butyl-4-methoxyphenol	1	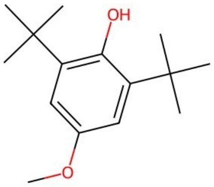	0.462
2,5-Di-tert-butylbenzene-1,4-diol	1	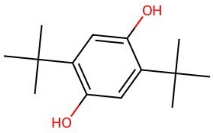	0.455

Query Compound: Bisphenol AF

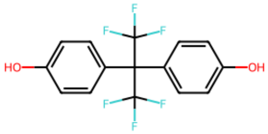

Predicted Activity: 1.0, Votes: 9.0, Docking Score: -6.97

Name	Hit2	Structure	Tanimoto Index
4-tert-Butylphenol	0	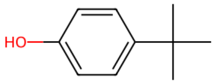	0.478
Bisphenol A	1	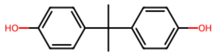	0.478
4,4',4''-Ethane-1,1,1-triyltriphenol	0	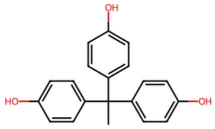	0.478
Hydroquinone	0	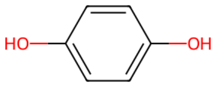	0.444
4-(2-Methylbutan-2-yl)phenol	0	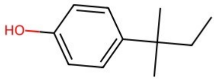	0.407

Query Compound: p,p'-DDD

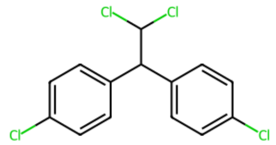

Predicted Activity: 1.0, Votes: 9.0, Docking Score: -6.95

Name	Hit2	Structure	Tanimoto Index
1,4-Dichlorobenzene	0	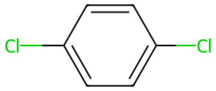	0.471
4-Chlorophenol	0	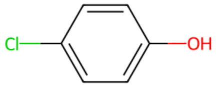	0.364
4-Chloroaniline	0	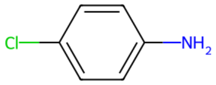	0.364
4-Chlorotoluene	0	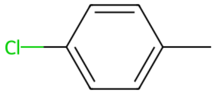	0.364
o,p'-DDT	0	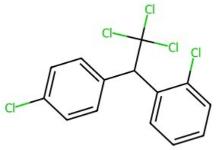	0.344

Query Compound: Fenbuconazole

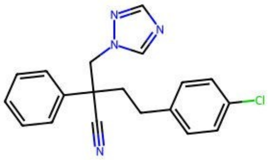

Predicted Activity: 1.0, Votes: 8.0, Docking Score: -7.98

Name	Hit2	Structure	Tanimoto Index
Myclobutanil	0	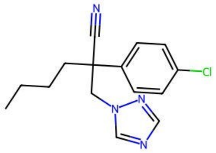	0.654
Tebuconazole	0	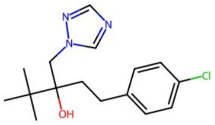	0.547
Cyproconazole	0	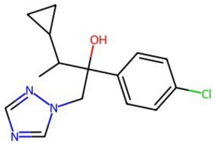	0.387
Anastrozole	0	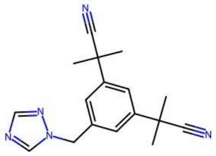	0.368

Paclobutrazol	0	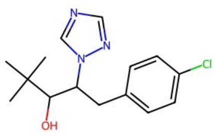	0.323

Query Compound: DDT

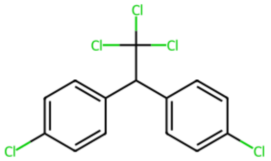

Predicted Activity: 1.0, Votes: 9.0, Docking Score: -6.89

Name	Hit2	Structure	Tanimoto Index
o,p'-DDT	0	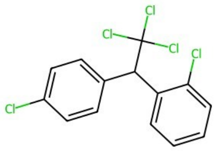	0.630
Methoxychlor	1	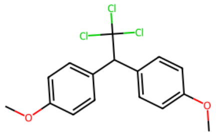	0.538
1,4-Dichlorobenzene	0	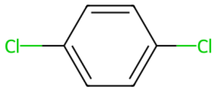	0.444

4-Chlorobenzotrichloride	0	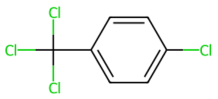	0.435
Dicofol	1	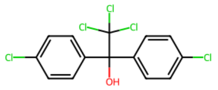	0.385

Query Compound: Diniconazole

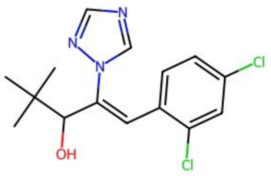

Predicted Activity: 1.0, Votes: 9.0, Docking Score: -6.87

Name	Hit2	Structure	Tanimoto Index
Triadimenol	0	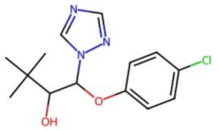	0.379
Paclobutrazol	0	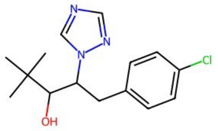	0.379

Triadimefon	0	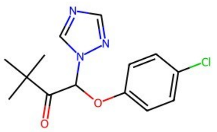	0.328
Tebuconazole	0	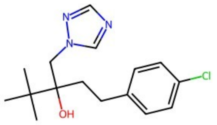	0.311
Cyproconazole	0	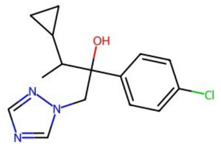	0.292

Query Compound: Tetraconazole

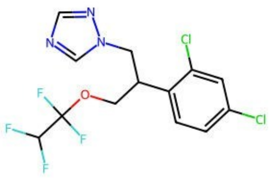

Predicted Activity: 1.0, Votes: 9.0, Docking Score: -6.85

Name	Hit2	Structure	Tanimoto Index
Econazole nitrate	1	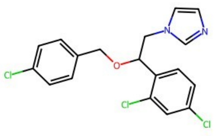	0.333

Cyproconazole	0	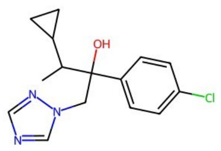	0.313
Tebuconazole	0	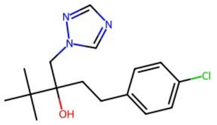	0.312
Myclobutanil	0	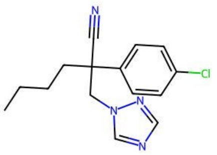	0.294
Fluconazole	0	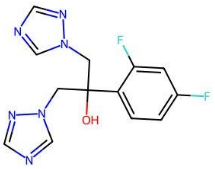	0.286

Query Compound: p,p'-DDE

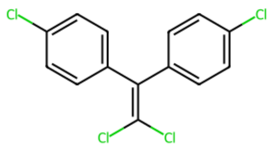

Predicted Activity: 1.0, Votes: 9.0, Docking Score: -6.83

Name	Hit2	Structure	Tanimoto Index

1,4-Dichlorobenzene	0	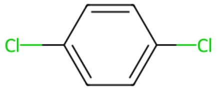	0.471
4-Chlorophenol	0	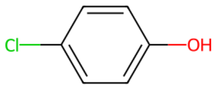	0.429
Ethyl 4-chlorophenyl ketone	0	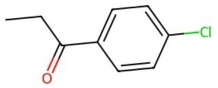	0.407
4-Chlorotoluene	0	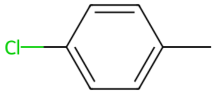	0.364
4-Chloroaniline	0	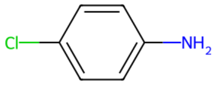	0.364

Query Compound: Dichlorophen

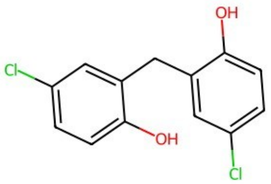

Predicted Activity: 1.0, Votes: 9.0, Docking Score: -6.83

Name	Hit2	Structure	Tanimoto Index
Clorophene	1	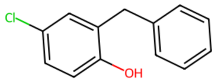	0.704
2,4-Dichlorophenol	0	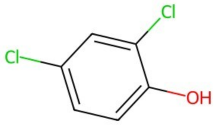	0.542
4-Chloro-2-methylphenol	0	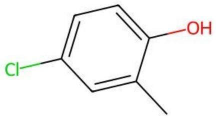	0.500
2,5-Dichlorophenol	0	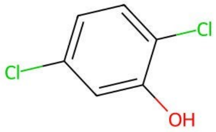	0.480
2,2'-Bisphenol F	0	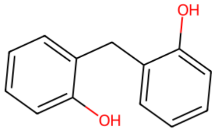	0.440

Query Compound: Bisphenol B

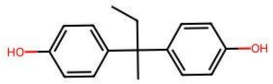

Predicted Activity: 1.0, Votes: 9.0, Docking Score: -6.79

Name	Hit2	Structure	Tanimoto Index
4-(2-Methylbutan-2-yl)phenol	0	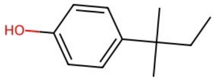	0.667
Bisphenol A	1	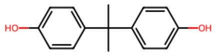	0.565
4-tert-Butylphenol	0	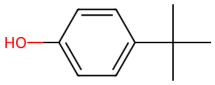	0.565
4,4',4''-Ethane-1,1,1-triyltriphenol	0	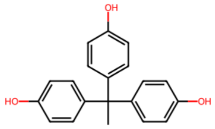	0.565
4,4-Bis(4-hydroxyphenyl)valeric acid	0	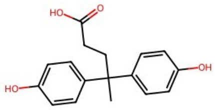	0.533

Query Compound: 5-Chloro-N-(2-chloro-4-nitrophenyl)-2-hydroxybenzamide

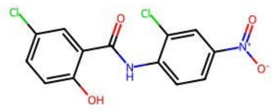

Predicted Activity: 1.0, Votes: 9.0, Docking Score: -6.79

Name	Hit2	Structure	Tanimoto Index
5-Chlorosalicylanilide	1	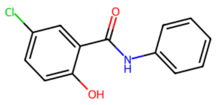	0.500
3,4-Dichloronitrobenzene	0	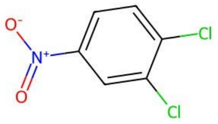	0.429
1-Chloro-4-nitrobenzene	0	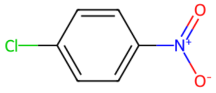	0.390
1-Chloro-2,4-dinitrobenzene	0	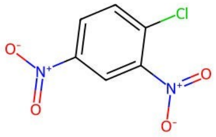	0.378
Cyclanilide	0	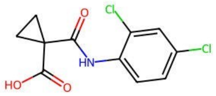	0.365

Query Compound: Triflumizole

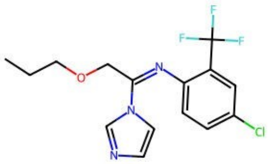

Predicted Activity: 1.0, Votes: 9.0, Docking Score: -6.79

Name	Hit2	Structure	Tanimoto Index
2,4-D-Butotyl	0	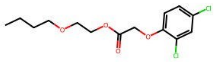	0.268
2,4-D Butyl ester	0	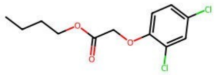	0.265
Econazole nitrate	1	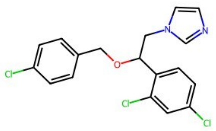	0.260
2,4-D-ethyl ester	0	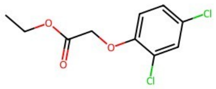	0.242

Flufenpyr-ethyl	0	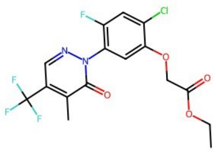	0.241

Query Compound: Bis(4-(dimethylamino)phenyl)methanone

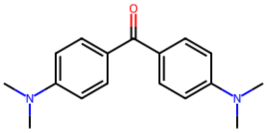

Predicted Activity: 1.0, Votes: 8.0, Docking Score: -7.85

Name	Hit2	Structure	Tanimoto Index
N,N,4-Trimethylaniline	0	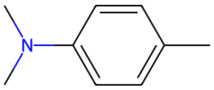	0.478
N,N-Dimethylaniline	0	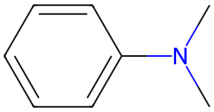	0.435
Benzophenone	0	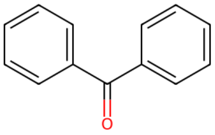	0.435

2-Ethylhexyl 4-(dimethylamino)benzoate	0	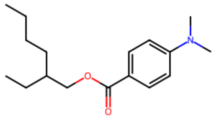	0.400
Methyl red	0	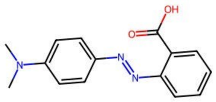	0.378

Query Compound: Phenolphthalein

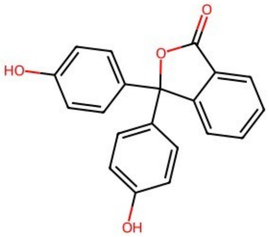

Predicted Activity: 1.0, Votes: 8.0, Docking Score: -7.81

Name	Hit2	Structure	Tanimoto Index
Fluorescein	0	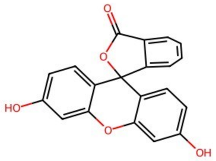	0.605
Phenol red	0	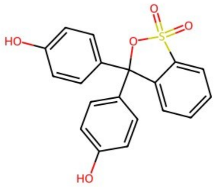	0.487

FD&C; Red 3	1	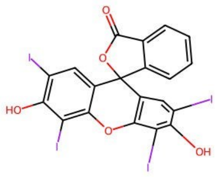	0.477
Eosin	1	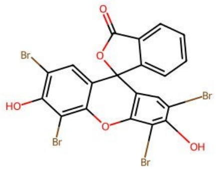	0.477
3-Hydroxyfluorene	0	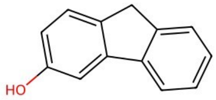	0.308

Query Compound: Chlorobenzilate

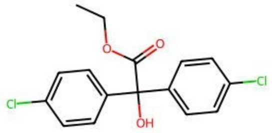

Predicted Activity: 1.0, Votes: 9.0, Docking Score: -6.72

Name	Hit2	Structure	Tanimoto Index
Clofibrate	0	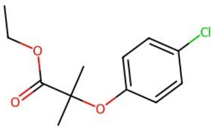	0.568

Triethyl citrate	0	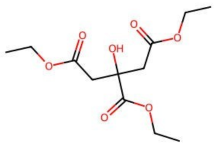	0.429
Ethyl 4-chlorophenyl ketone	0	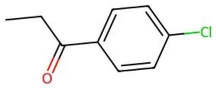	0.400
2,4-D-ethyl ester	0	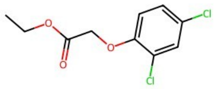	0.395
Dicofol	1	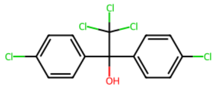	0.394

Query Compound: AVE5638

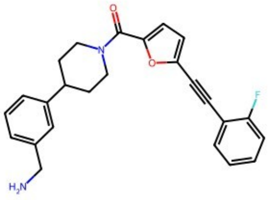

Predicted Activity: 1.0, Votes: 7.0, Docking Score: -8.87

Name	Hit2	Structure	Tanimoto Index

AVE8923	0	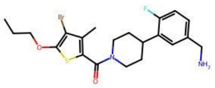	0.341
1,3-Benzenedimethanamine	0	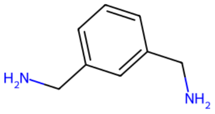	0.250
MK-578	0	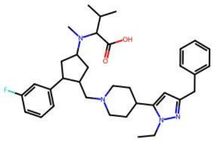	0.248
SB202235	0	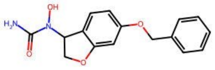	0.205
Enterolactone	0	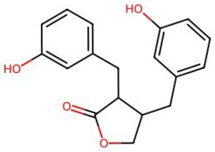	0.189

Query Compound: Phenylparaben

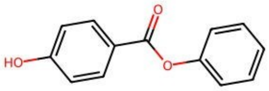

Predicted Activity: 1.0, Votes: 9.0, Docking Score: -6.68

Name	Hit2	Structure	Tanimoto Index
Phenyl benzoate	0	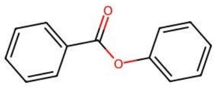	0.760
Diphenyl isophthalate	1	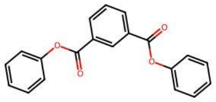	0.621
Phenyl salicylate	1	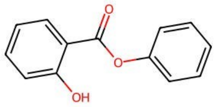	0.548
Methylparaben	0	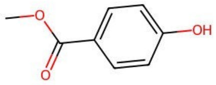	0.517
4-Hydroxybenzoic acid	0	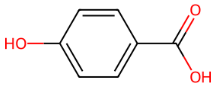	0.481

Query Compound: Prodiamine

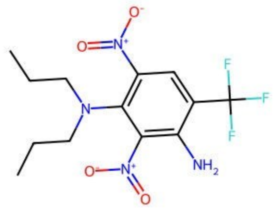

Predicted Activity: 1.0, Votes: 9.0, Docking Score: -6.68

Name	Hit2	Structure	Tanimoto Index
Trifluralin	1	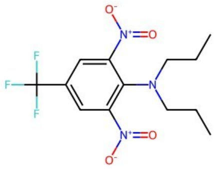	0.614
Isopropalin	0	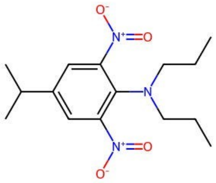	0.489
Flumetralin	1	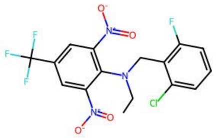	0.349
Hydroxyflutamide	0	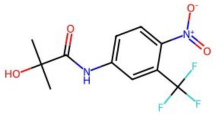	0.310
5,5-Dimethyl-3-(alpha,alpha,alpha-trifluoro-4-nitro-m-tolyl)hydantoin	0	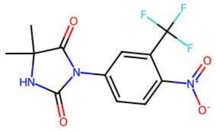	0.295

Query Compound: (+-)-Diclofop-methyl

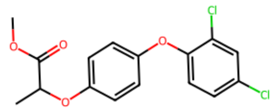

Predicted Activity: 1.0, Votes: 9.0, Docking Score: -6.68

Name	Hit2	Structure	Tanimoto Index
Dichlorprop	0	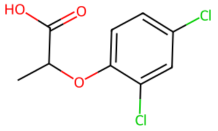	0.535
Haloxyfop-methyl	0	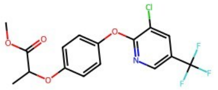	0.519
Mecoprop	0	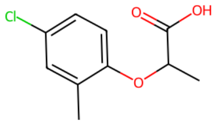	0.426
Triclosan	1	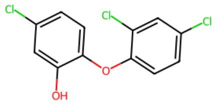	0.419
2,4-D-ethyl ester	0	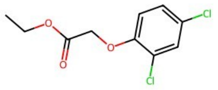	0.408

Query Compound: Fenarimol

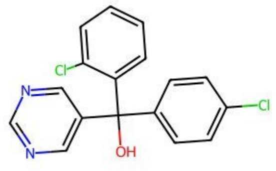

Predicted Activity: 1.0, Votes: 9.0, Docking Score: -6.6

Name	Hit2	Structure	Tanimoto Index
Dicofol	1	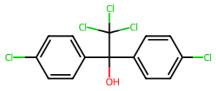	0.351
4-Chlorobenzotrichloride	0	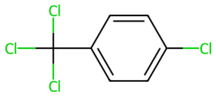	0.343
o,p'-DDT	0	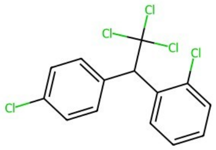	0.318
1-Chloro-4-(trifluoromethyl)benzene	0	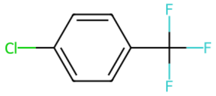	0.297

Cyproconazole	0	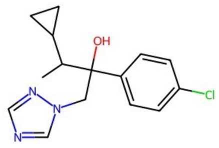	0.276

Query Compound: Mepronil

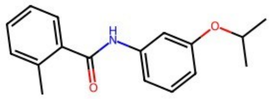

Predicted Activity: 1.0, Votes: 9.0, Docking Score: -6.6

Name	Hit2	Structure	Tanimoto Index
Flutolanil	1	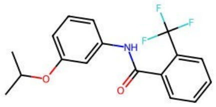	0.682
Desmedipham	0	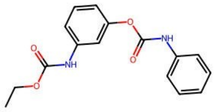	0.458
Phenmedipham	0	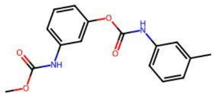	0.447

Methyl 2-methylbenzoate	0	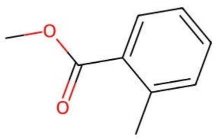	0.425
Chlorpropham	1	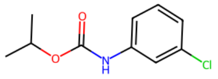	0.404

Query Compound: Chlorfenapyr

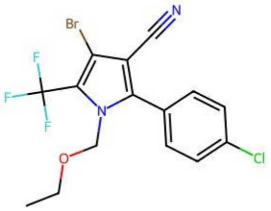

Predicted Activity: 1.0, Votes: 9.0, Docking Score: -6.6

Name	Hit2	Structure	Tanimoto Index
1-Chloro-4-(trifluoromethyl)benzene	0	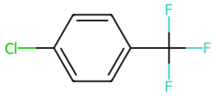	0.250
Clofibrate	0	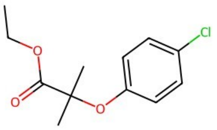	0.233

Myclobutanil	0	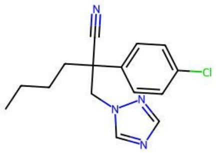	0.229
Cyazofamid	1	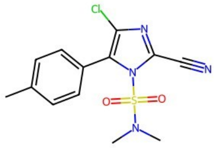	0.221
Dichlobenil	0	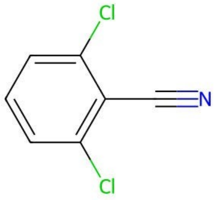	0.220

Query Compound: Forchlorfenuron

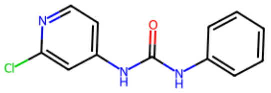

Predicted Activity: 1.0, Votes: 9.0, Docking Score: -6.58

Name	Hit2	Structure	Tanimoto Index
Triclocarban	1	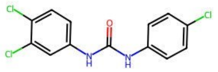	0.436

Fenuron	0	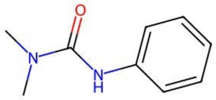	0.405
2-Chloro-N-phenylacetamide	0	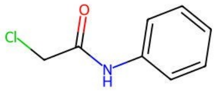	0.405
1-Phenylurea	0	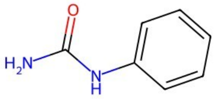	0.400
5-Chlorosalicylanilide	1	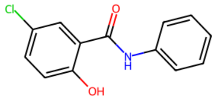	0.395

Query Compound: 2,4-Di-tert-pentylphenol

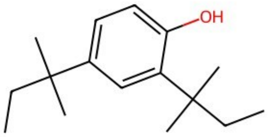

Predicted Activity: 1.0, Votes: 9.0, Docking Score: -6.55

Name	Hit2	Structure	Tanimoto Index

4-(2-Methylbutan-2-yl)phenol	0	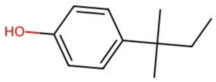	0.552
2,4-Di-tert-butylphenol	1	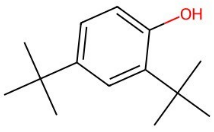	0.533
2,4-Bis(1-methyl-1-phenylethyl)phenol	1	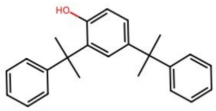	0.457
tert-Butylhydroquinone	0	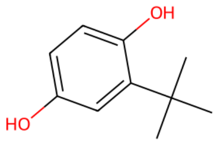	0.406
4-Methyl-2-tert-butylphenol	0	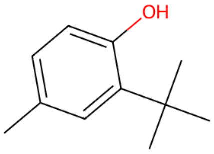	0.394

Query Compound: Fenoxycarb

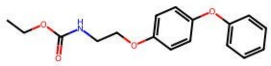

Predicted Activity: 1.0, Votes: 9.0, Docking Score: -6.53

Name	Hit2	Structure	Tanimoto Index
Desmedipham	0	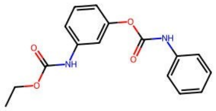	0.429
Ethyl benzoate	0	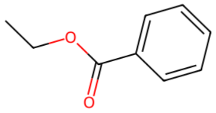	0.390
1,2-Diphenoxyethane	0	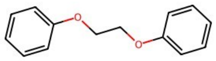	0.389
2-Phenoxyethanol	0	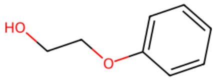	0.385
Thiophanate	0	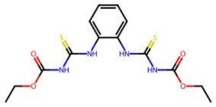	0.383

Query Compound: Amitraz

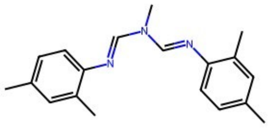

Predicted Activity: 1.0, Votes: 9.0, Docking Score: -6.53

Name	Hit2	Structure	Tanimoto Index
1,2,4-Trimethylbenzene	0	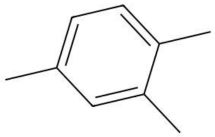	0.393
2,4-Dimethylphenol	0	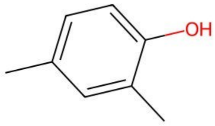	0.355
2,5-Dimethylphenol	0	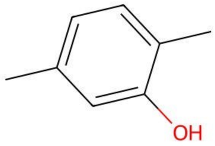	0.312
1,3-Dimethyl-4-nitrobenzene	0	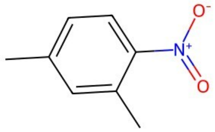	0.306
Sodium m-xylene-4-sulfonate	0	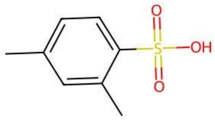	0.306

Query Compound: Picoxystrobin

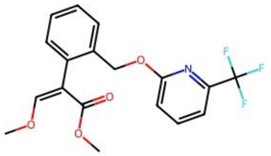

Predicted Activity: 1.0, Votes: 9.0, Docking Score: -6.49

Name	Hit2	Structure	Tanimoto Index
Azoxystrobin	1	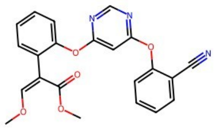	0.412
Kresoxim-methyl	1	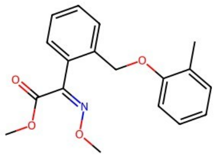	0.354
Dimethyl phthalate	0	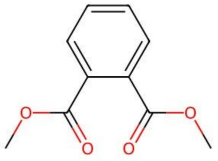	0.269
Flutolanil	1	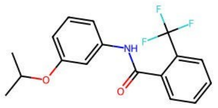	0.261
Trifloxysulfuron-sodium	0	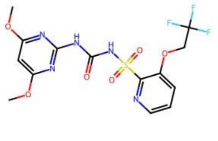	0.253

Query Compound: Flusilazole

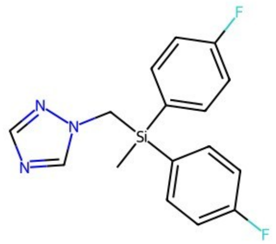

Predicted Activity: 1.0, Votes: 8.0, Docking Score: -7.56

Name	Hit2	Structure	Tanimoto Index
Fluconazole	0	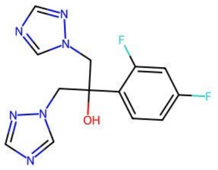	0.413
Tebuconazole	0	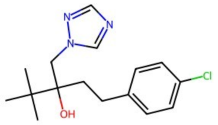	0.308
Anastrozole	0	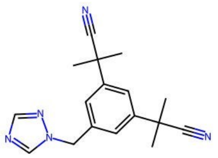	0.306
Myclobutanil	0	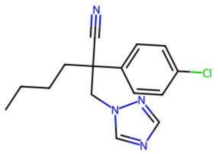	0.286

Cyproconazole	0	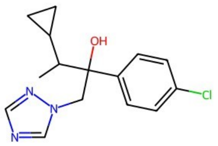	0.286

Query Compound: Phenolphthalin

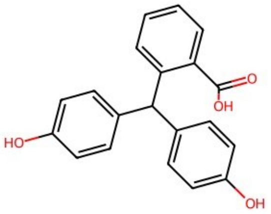

Predicted Activity: 1.0, Votes: 8.0, Docking Score: -7.55

Name	Hit2	Structure	Tanimoto Index
Monopotassium phthalate	0	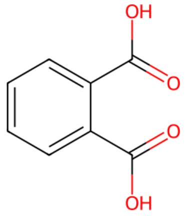	0.464
Salicylic acid	0	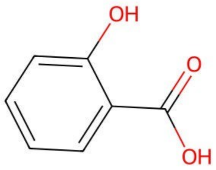	0.452
4-Hydroxybenzoic acid	0	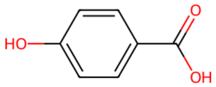	0.387

Monomethyl phthalate	0	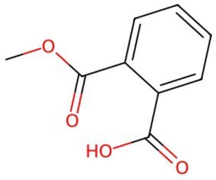	0.361
Monobenzyl phthalate	0	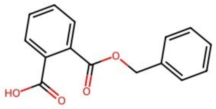	0.359

Query Compound: 9-Phenanthrol

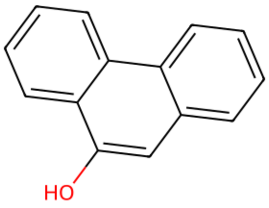

Predicted Activity: 1.0, Votes: 7.0, Docking Score: -8.63

Name	Hit2	Structure	Tanimoto Index
Dibenz(a,h)anthracene	0	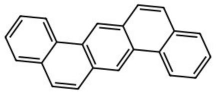	0.522
1-Naphthol	0	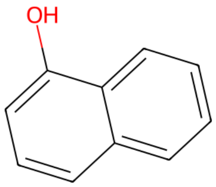	0.500

Benzo(b)fluoranthene	0	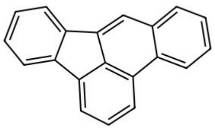	0.414
2-Naphthalenol	0	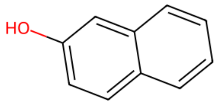	0.385
1,?2-?Benzenediol	0	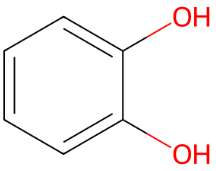	0.381

Query Compound: SB243213A

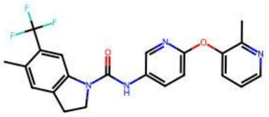

Predicted Activity: 1.0, Votes: 8.0, Docking Score: -7.54

Name	Hit2	Structure	Tanimoto Index
Trifloxysulfuron-sodium	0	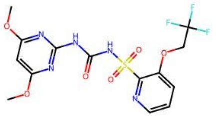	0.291

Flutolanil	1	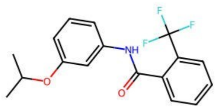	0.269
Flufenpyr-ethyl	0	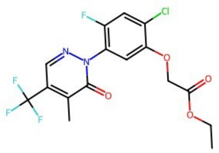	0.242
Haloxyfop-methyl	0	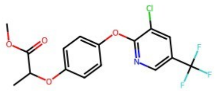	0.235
Hydroxyflutamide	0	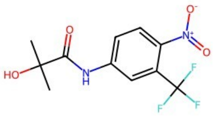	0.228

Query Compound: Fenpyroximate (Z,E)

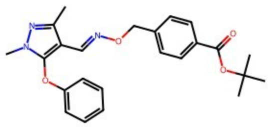

Predicted Activity: 1.0, Votes: 9.0, Docking Score: -6.44

Name	Hit2	Structure	Tanimoto Index

Benzyl benzoate	1	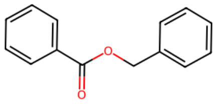	0.300
tert-Butyl perbenzoate	0	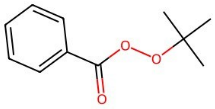	0.274
Phenothrin	0	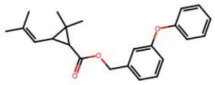	0.266
Permethrin	0	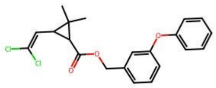	0.266
Dimethylbenzylcarbinyl acetate	0	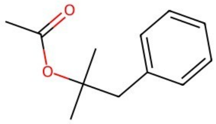	0.266

Query Compound: Bifenazate

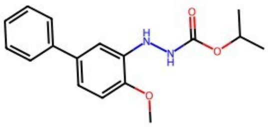

Predicted Activity: 1.0, Votes: 9.0, Docking Score: -6.44

Name	Hit2	Structure	Tanimoto Index
Propham	0	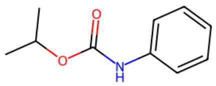	0.422
Chlorpropham	1	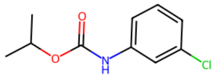	0.346
Diisopropyl phthalate	0	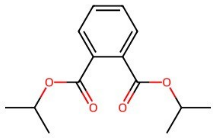	0.333
Propoxur	0	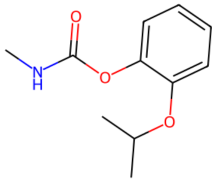	0.314
Sulisobenzone	0	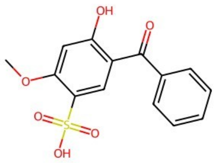	0.293

Query Compound: 4,4'-Dichlorodiphenyl sulfone

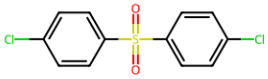

Predicted Activity: 1.0, Votes: 9.0, Docking Score: -6.44

Name	Hit2	Structure	Tanimoto Index
4,4'-Sulfonyldiphenol	0	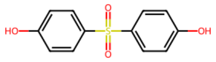	0.524
Dapsone	0	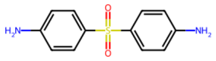	0.524
1,4-Dichlorobenzene	0	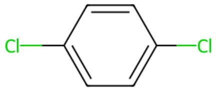	0.500
1-Chloro-4-nitrobenzene	0	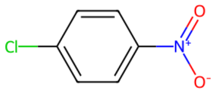	0.417
4-Chlorotoluene	0	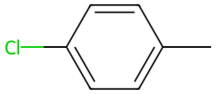	0.381

Query Compound: 4-tert-Butylphenyl salicylate

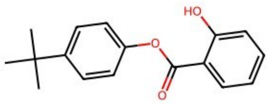

Predicted Activity: 1.0, Votes: 9.0, Docking Score: -6.39

Name	Hit2	Structure	Tanimoto Index
Phenyl salicylate	1	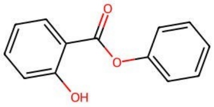	0.667
Methyl salicylate	0	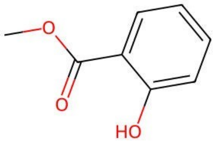	0.514
2-Hydroxyacetophenone	0	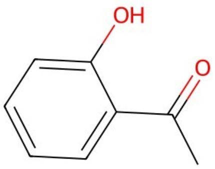	0.429
Hexyl salicylate	1	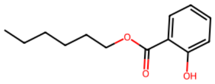	0.409
Salicylic acid	0	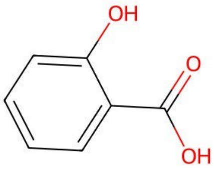	0.400

Query Compound: Quizalofop-ethyl

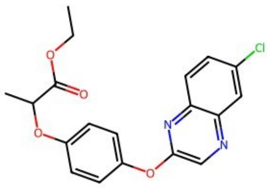

Predicted Activity: 1.0, Votes: 9.0, Docking Score: -6.36

Name	Hit2	Structure	Tanimoto Index
Clodinafop-propargyl	1	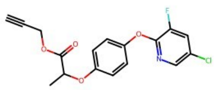	0.446
Haloxyfop-methyl	0	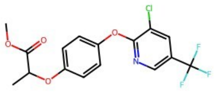	0.394
2,4-D-ethyl ester	0	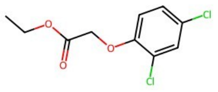	0.339
Mecoprop	0	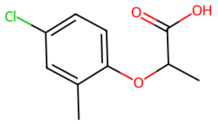	0.328

Clofibrate	0	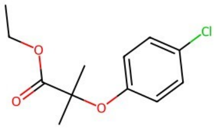	0.328

Query Compound: 2,2',6,6'-Tetrachlorobisphenol A

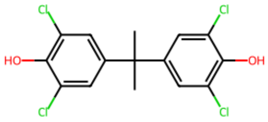

Predicted Activity: 1.0, Votes: 9.0, Docking Score: -6.3

Name	Hit2	Structure	Tanimoto Index
2,4,6-Trichlorophenol	0	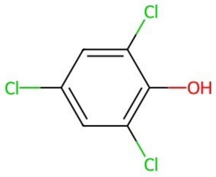	0.500
Bisphenol A	1	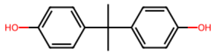	0.400
2,4,6-Tris(tert-butyl)phenol	0	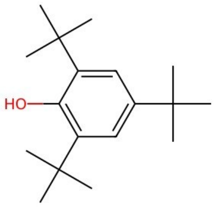	0.370

2,4-Bis(1-methyl-1-phenylethyl)phenol	1	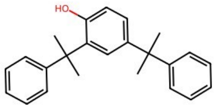	0.324
1,2-Dichloro-4-(trifluoromethyl)benzene	0	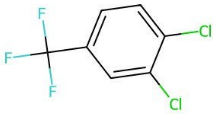	0.310

Query Compound: Methylene blue

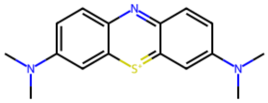

Predicted Activity: 1.0, Votes: 8.0, Docking Score: -7.38

Name	Hit2	Structure	Tanimoto Index
N,N,4-Trimethylaniline	0	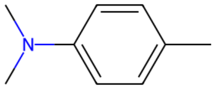	0.345
3-Dimethylaminophenol	0	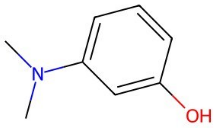	0.333

N,N-Dimethylaniline	0	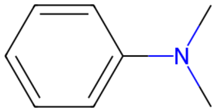	0.310
7-(Dimethylamino)-4-methylcoumarin	0	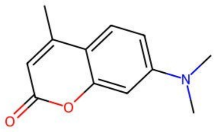	0.293
6-Methylquinoline	0	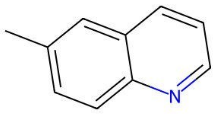	0.270

Query Compound: Phosalone

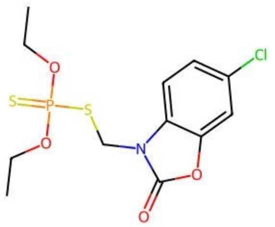

Predicted Activity: 1.0, Votes: 9.0, Docking Score: -6.27

Name	Hit2	Structure	Tanimoto Index
Azamethiphos	0	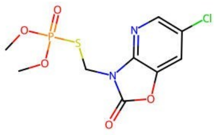	0.417

Phosmet	0	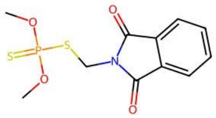	0.298
Terbufos	1	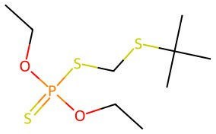	0.288
Sulprofos	1	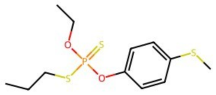	0.283
2,4-D-ethyl ester	0	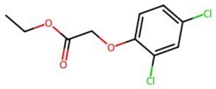	0.271

Query Compound: Fenoxaprop-ethyl

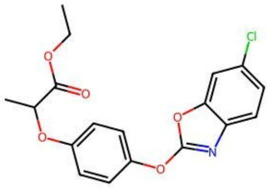

Predicted Activity: 1.0, Votes: 9.0, Docking Score: -6.22

Name	Hit2	Structure	Tanimoto Index

Clodinafop-propargyl	1	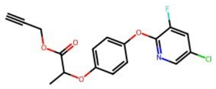	0.403
Haloxyfop-methyl	0	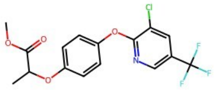	0.353
Clofibrate	0	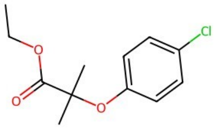	0.328
Mecoprop	0	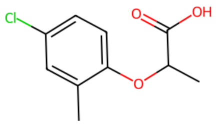	0.328
2,4-D-ethyl ester	0	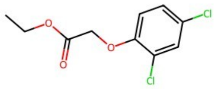	0.317

Query Compound: Ethalfluralin

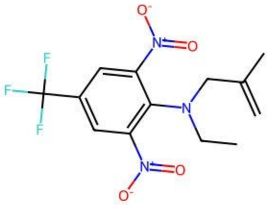

Predicted Activity: 1.0, Votes: 9.0, Docking Score: -6.18

Name	Hit2	Structure	Tanimoto Index
Trifluralin	1	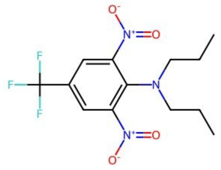	0.628
Flumetralin	1	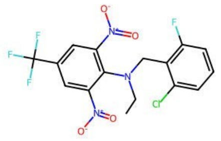	0.527
Isopropalin	0	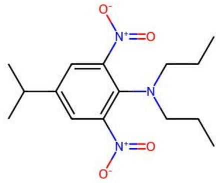	0.380
Oxyfluorfen	1	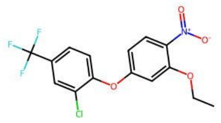	0.317
Hydroxyflutamide	0	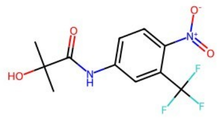	0.293

Query Compound: Prochloraz

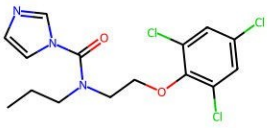

Predicted Activity: 1.0, Votes: 9.0, Docking Score: -6.16

Name	Hit2	Structure	Tanimoto Index
2,4-D Butyl ester	0	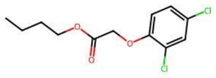	0.286
Econazole nitrate	1	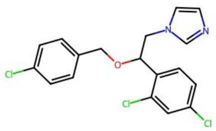	0.279
2,4-D-Butotyl	0	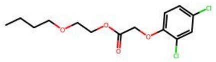	0.269
2,4-D-ethyl ester	0	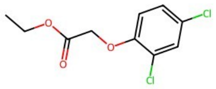	0.262
2,4-Dichlorophenoxybutyric acid	0	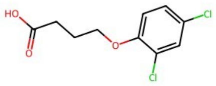	0.254

Query Compound: Clotrimazole

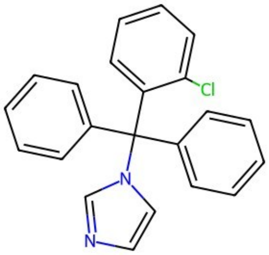

Predicted Activity: 1.0, Votes: 9.0, Docking Score: -6.15

Name	Hit2	Structure	Tanimoto Index
Benzotrichloride	0	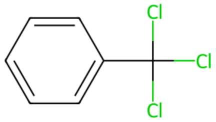	0.314
1,2-Dichlorobenzene	0	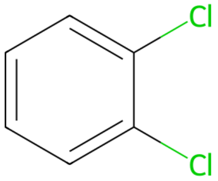	0.273
Clofentezine	0	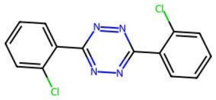	0.250
tert-Butylbenzene	0	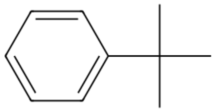	0.243
2-Chlorophenol	0	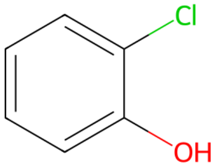	0.237

Query Compound: Allethrin

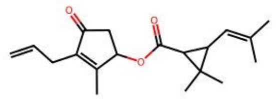

Predicted Activity: 1.0, Votes: 9.0, Docking Score: -6.14

Name	Hit2	Structure	Tanimoto Index
Phenothrin	0	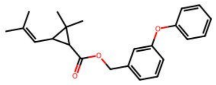	0.343
Resmethrin	0	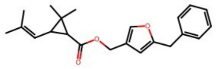	0.329
Tefluthrin	0	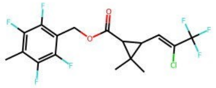	0.294
Permethrin	0	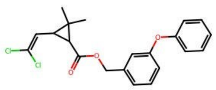	0.268

Bifenthrin	0	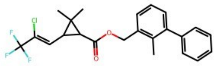	0.263

Query Compound: 4-(1,1,3,3-Tetramethylbutyl)phenol

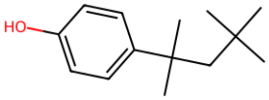

Predicted Activity: 1.0, Votes: 9.0, Docking Score: -6.12

Name	Hit2	Structure	Tanimoto Index
4-(2-Methylbutan-2-yl)phenol	0	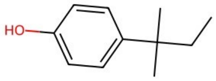	0.640
Bisphenol A	1	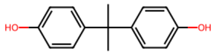	0.542
4,4',4''-Ethane-1,1,1-triyltriphenol	0	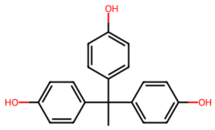	0.542

4-tert-Butylphenol	0	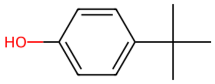	0.542
Octrizole	0	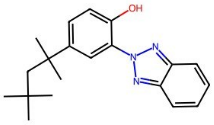	0.425

Query Compound: Carfentrazone-ethyl

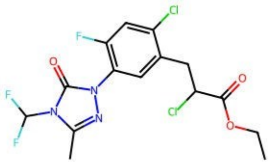

Predicted Activity: 1.0, Votes: 8.0, Docking Score: -7.15

Name	Hit2	Structure	Tanimoto Index
Sulfentrazone	0	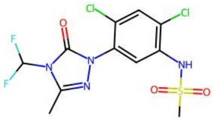	0.431
Flufenpyr-ethyl	0	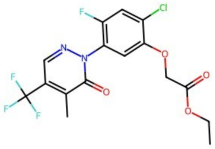	0.355

Pyraflufen-ethyl	0	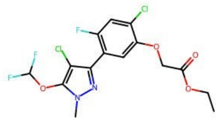	0.321
2,4-D-ethyl ester	0	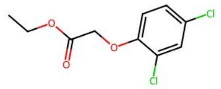	0.242
Chlorpyrifos oxon	0	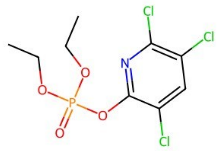	0.227

Query Compound: FR150011

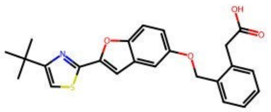

Predicted Activity: 1.0, Votes: 7.0, Docking Score: -8.16

Name	Hit2	Structure	Tanimoto Index
1-Naphthaleneacetic acid	0	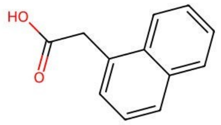	0.274

Octabenzone	0	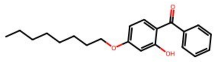	0.240
SB202235	0	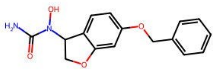	0.235
Diclofenac sodium	0	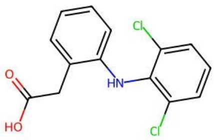	0.229
CP-114271	0	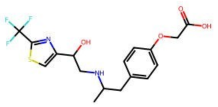	0.227

Query Compound: Hexane-1,6-diyl dibenzoate

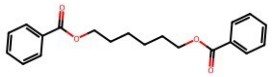

Predicted Activity: 1.0, Votes: 8.0, Docking Score: -7.06

Name	Hit2	Structure	Tanimoto Index

Pentane-1,5-diyl dibenzoate	0	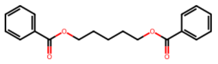	1.000
Hexyl benzoate	0	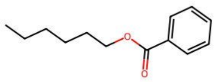	0.821
Butyl benzoate	0	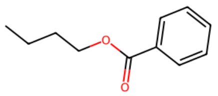	0.750
Diethylene glycol dibenzoate	0	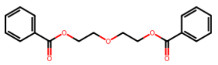	0.704
Ethylenebis(oxyethylene) dibenzoate	0	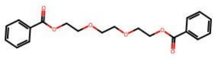	0.704

Query Compound: Coumaphos

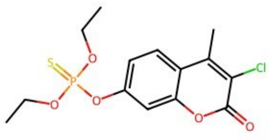

Predicted Activity: 1.0, Votes: 9.0, Docking Score: -5.97

Name	Hit2	Structure	Tanimoto Index
Fenitrothion	1	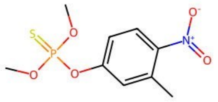	0.316
Diazinon	0	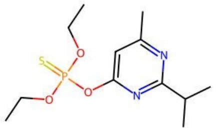	0.310
5,7-Dimethoxy-2H-chromen-2-one	0	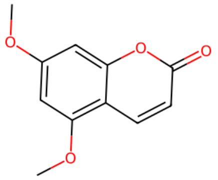	0.291
Isazofos	1	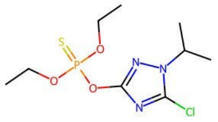	0.288
Fenamiphos	0	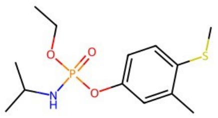	0.286

Query Compound: 1,3-Diphenyl-1,3-propanedione

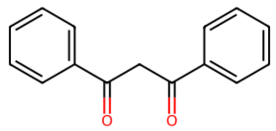

Predicted Activity: 1.0, Votes: 8.0, Docking Score: -7.03

Name	Hit2	Structure	Tanimoto Index
2-Chloroacetophenone	0	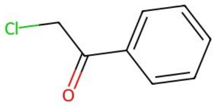	0.636
1,2-Diphenylethanone	0	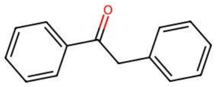	0.609
Sodium benzoate	0	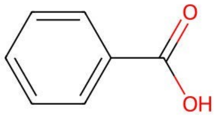	0.550
Benzophenone	0	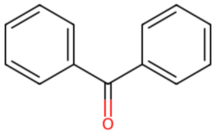	0.550
Heptanophenone	0	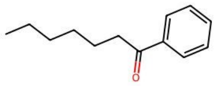	0.500

Query Compound: Fenthion

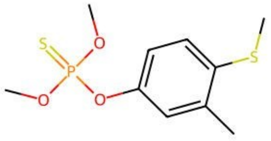

Predicted Activity: 1.0, Votes: 9.0, Docking Score: -5.95

Name	Hit2	Structure	Tanimoto Index
Fenitrothion	1	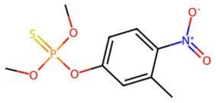	0.537
Fenamiphos	0	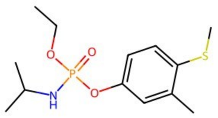	0.468
Methyl parathion	0	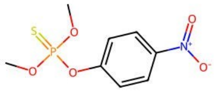	0.415
UK-416244	0	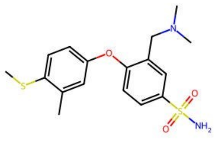	0.352
Chlorpyrifos-methyl	1	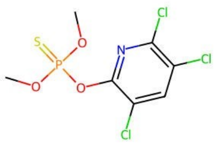	0.318

Query Compound: Fluoxastrobin

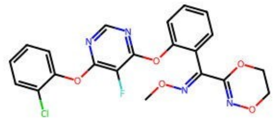

Predicted Activity: 1.0, Votes: 8.0, Docking Score: -7.0

Name	Hit2	Structure	Tanimoto Index
Kresoxim-methyl	1	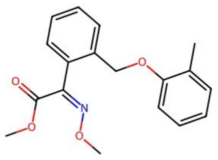	0.240
Azoxystrobin	1	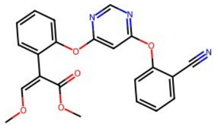	0.232
CP-457677	0	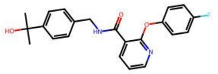	0.200
Haloxyfop-methyl	0	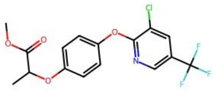	0.195

Clodinafop-propargyl	1	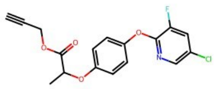	0.190

Query Compound: Heptachlor epoxide B

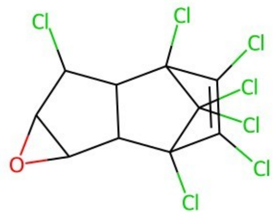

Predicted Activity: 1.0, Votes: 9.0, Docking Score: -5.89

Name	Hit2	Structure	Tanimoto Index
Chlorendic acid	0	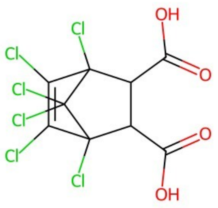	0.333
Hexachlorocyclopentadiene	1	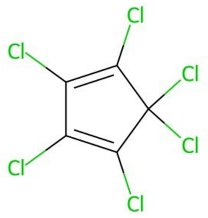	0.207
Lindane	1	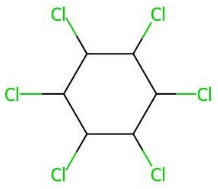	0.160

Kepone	0	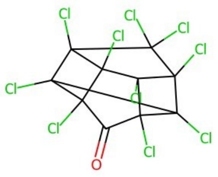	0.147
Mirex	0	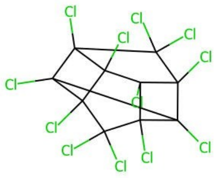	0.143

Query Compound: Captafol

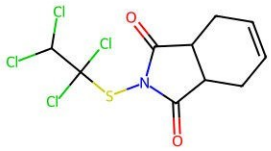

Predicted Activity: 1.0, Votes: 9.0, Docking Score: -5.89

Name	Hit2	Structure	Tanimoto Index
1,2,3,6-Tetrahydrophthalimide	0	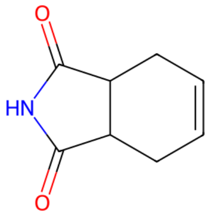	0.314
Benoxacor	0	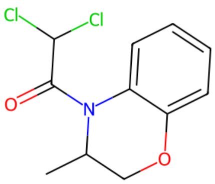	0.185

Triforine	1	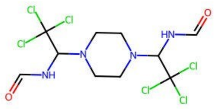	0.159
Buprofezin	1	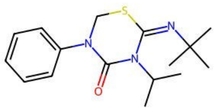	0.158
Procymidone	0	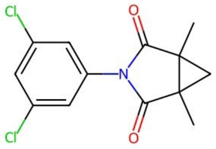	0.149

Query Compound: Dieldrin

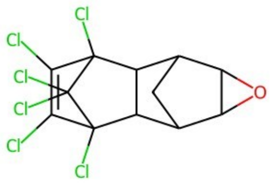

Predicted Activity: 1.0, Votes: 9.0, Docking Score: -5.86

Name	Hit2	Structure	Tanimoto Index
Chlorendic acid	0	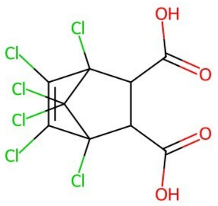	0.333

Hexachlorocyclopentadiene	1	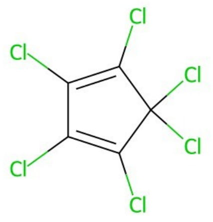	0.207
(-)-beta-Pinene	0	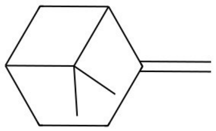	0.150
Kepone	0	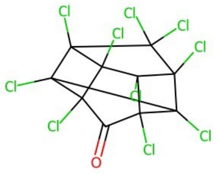	0.147
Mirex	0	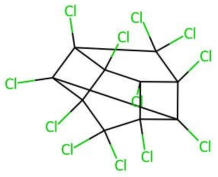	0.143

Query Compound: Propiconazole

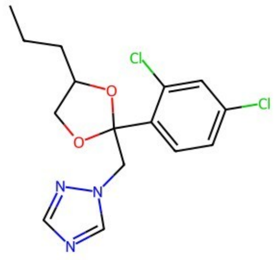

Predicted Activity: 1.0, Votes: 8.0, Docking Score: -6.89

Name	Hit2	Structure	Tanimoto Index

Triticonazole	0	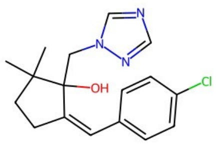	0.357
Cyproconazole	0	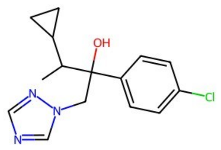	0.353
Myclobutanil	0	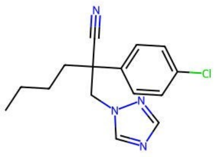	0.333
Tebuconazole	0	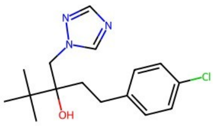	0.313
Paclobutrazol	0	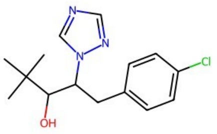	0.257

Query Compound: Perfluorooctanesulfonamide

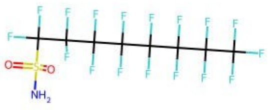

Predicted Activity: 1.0, Votes: 9.0, Docking Score: -5.8

Name	Hit2	Structure	Tanimoto Index
Potassium perfluorohexanesulfonate	1	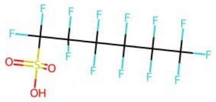	0.565
Perfluorooctanesulfonic acid	1	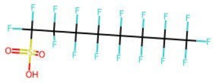	0.565
Potassium perfluorobutanesulfonate	0	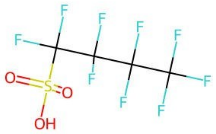	0.522
Perfluoroundecanoic acid	0	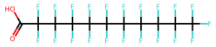	0.346
Perfluorooctanoic acid	0	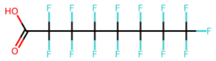	0.346

Query Compound: Aldrin

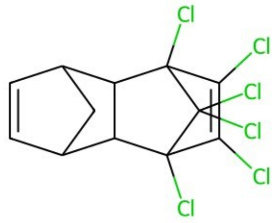

Predicted Activity: 1.0, Votes: 9.0, Docking Score: -5.79

Name	Hit2	Structure	Tanimoto Index
Chlorendic acid	0	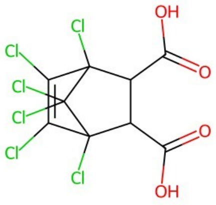	0.355
2-Norbornene-5,6-dicarboxylic anhydride	0	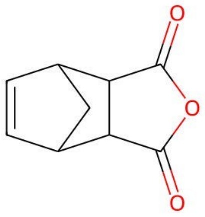	0.281
Dicyclopentadiene	0	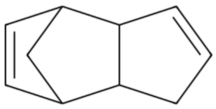	0.242
Hexachlorocyclopentadiene	1	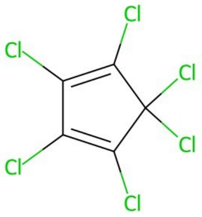	0.222
3,3,5-Trimethylcyclohexanol	0	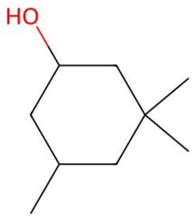	0.171

Query Compound: 4,4'-Sulfonylbis[2-(prop-2-en-1-yl)phenol]

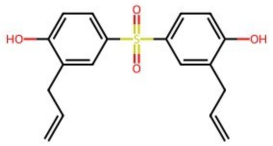

Predicted Activity: 1.0, Votes: 8.0, Docking Score: -6.87

Name	Hit2	Structure	Tanimoto Index
4,4'-Sulfonyldiphenol	0	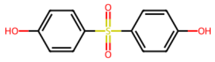	0.375
Eugenol	0	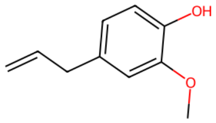	0.366
Safrole	0	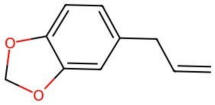	0.317
Dapsone	0	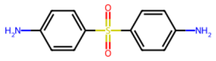	0.294
2-Ethylphenol	0	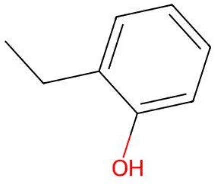	0.278

Query Compound: o-Aminoazotoluene

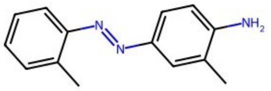

Predicted Activity: 1.0, Votes: 8.0, Docking Score: -6.84

Name	Hit2	Structure	Tanimoto Index
2-Methylaniline	0	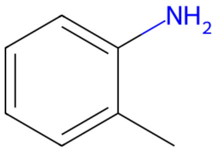	0.433
3,3'-Dimethylbenzidine	0	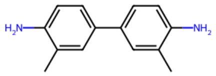	0.406
4,4'-Methylenebis(o-toluidine)	0	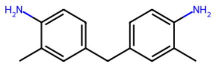	0.371
C.I. Solvent Yellow 1	0	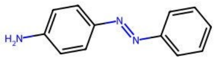	0.353

Toluene 2,4-diisocyanate	0	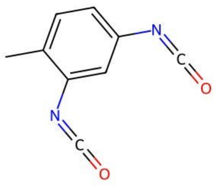	0.333

Query Compound: Nitrofen

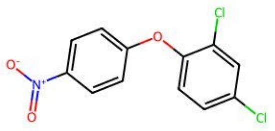

Predicted Activity: 1.0, Votes: 9.0, Docking Score: -5.76

Name	Hit2	Structure	Tanimoto Index
1-Chloro-4-nitrobenzene	0	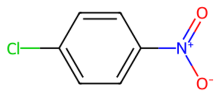	0.531
Triclosan	1	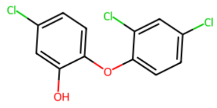	0.447
3,4-Dichloronitrobenzene	0	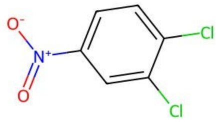	0.444

1-Chloro-2,4-dinitrobenzene	0	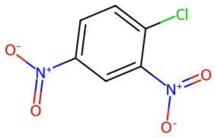	0.421
Acifluorfen	0	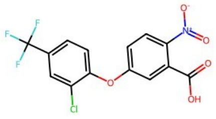	0.415

Query Compound: Flavone

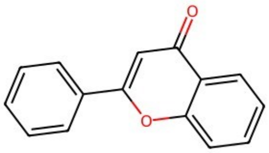

Predicted Activity: 1.0, Votes: 8.0, Docking Score: -6.83

Name	Hit2	Structure	Tanimoto Index
Dibenzofuran	0	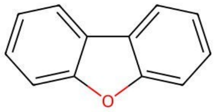	0.429
Coumarin	0	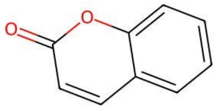	0.314

Warfarin	0	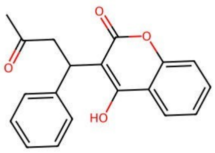	0.312
Biphenyl	0	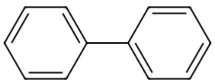	0.296
2,3-Benzofuran	0	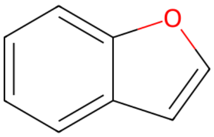	0.265

Query Compound: Temephos

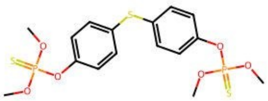

Predicted Activity: 1.0, Votes: 9.0, Docking Score: -5.71

Name	Hit2	Structure	Tanimoto Index
Methyl parathion	0	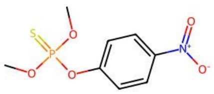	0.529

Fenitrothion	1	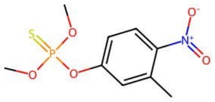	0.425
Sulprofos	1	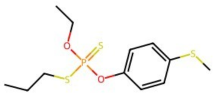	0.349
Chlorpyrifos-methyl	1	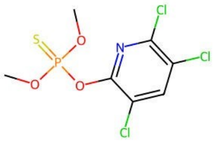	0.333
Hydroquinone dimethyl ether	0	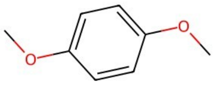	0.296

Query Compound: Butralin

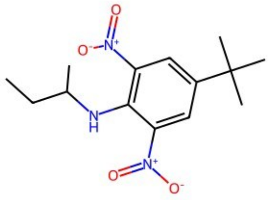

Predicted Activity: 1.0, Votes: 9.0, Docking Score: -5.68

Name	Hit2	Structure	Tanimoto Index

Trifluralin	1	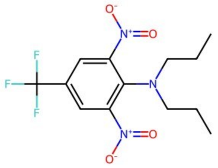	0.354
Isopropalin	0	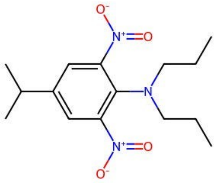	0.333
1,3-Dimethyl-4-nitrobenzene	0	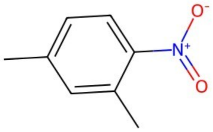	0.279
Flumetralin	1	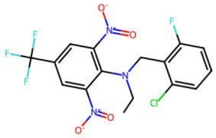	0.274
2,4-Dinitrotoluene	0	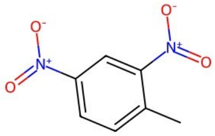	0.273

Query Compound: Zoxamide

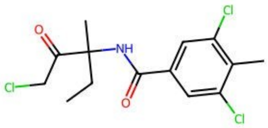

Predicted Activity: 1.0, Votes: 8.0, Docking Score: -6.73

Name	Hit2	Structure	Tanimoto Index
Propyzamide	0	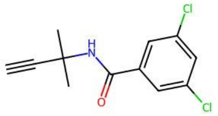	0.362
Propanil	0	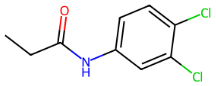	0.265
Tebufenozide	1	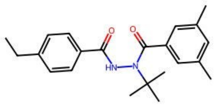	0.259
2-Chloroacetophenone	0	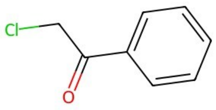	0.256
Halofenozide	0	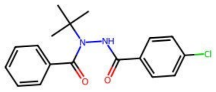	0.255

Query Compound: 3,3',5,5'-Tetrabromobisphenol A

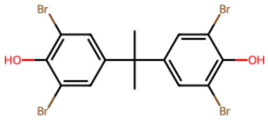

Predicted Activity: 1.0, Votes: 9.0, Docking Score: -5.63

Name	Hit2	Structure	Tanimoto Index
2,4,6-Tribromophenol	0	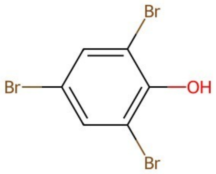	0.500
Bromoxynil	0	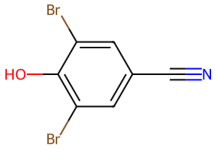	0.407
Bisphenol A	1	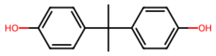	0.400
2,4,6-Tris(tert-butyl)phenol	0	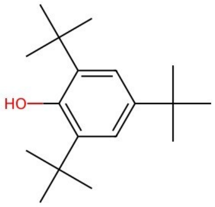	0.370
2,4-Bis(1-methyl-1-phenylethyl)phenol	1	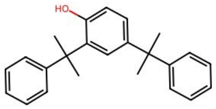	0.324

Query Compound: Folpet

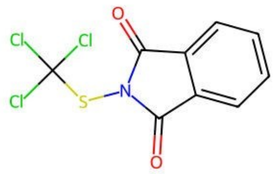

Predicted Activity: 1.0, Votes: 9.0, Docking Score: -5.61

Name	Hit2	Structure	Tanimoto Index
N-Methylphthalimide	0	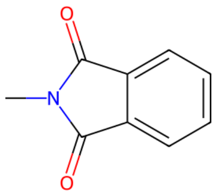	0.429
Phosmet	0	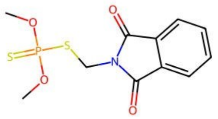	0.317
Phthalimide	0	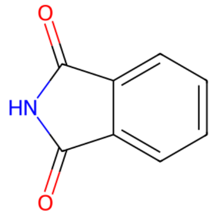	0.310
Thalidomide	0	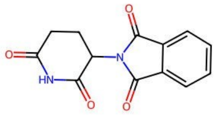	0.300
Benzotrichloride	0	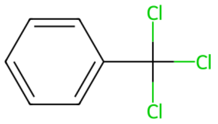	0.276

Query Compound: Tiratricol

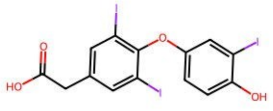

Predicted Activity: 1.0, Votes: 8.0, Docking Score: -6.68

Name	Hit2	Structure	Tanimoto Index
Tetrac	0	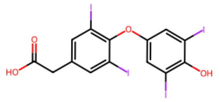	0.711
3,5,3'-Triiodothyronine	1	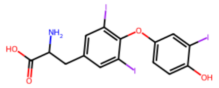	0.659
4-Chlorophenoxyacetic acid	0	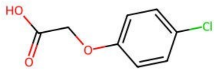	0.295
3-Phenoxybenzenemethanol	0	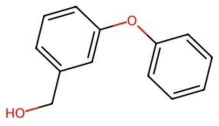	0.261

3-Phenoxybenzoic acid	0	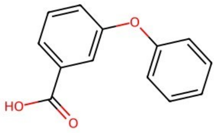	0.261

Query Compound: CP-105696

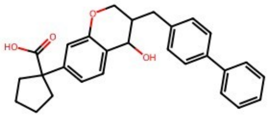

Predicted Activity: 1.0, Votes: 7.0, Docking Score: -7.76

Name	Hit2	Structure	Tanimoto Index
CP-085958	0	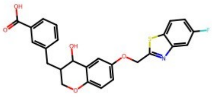	0.315
SB202235	0	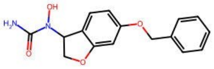	0.301
Enterolactone	0	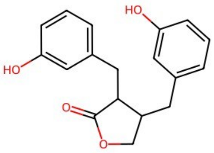	0.297

Benoxacor	0	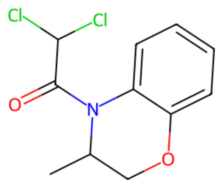	0.214
4,5-dihydro-5,5-diphenyl-1,2-oxazole-3-carboxylic acid, ethyl ester	0	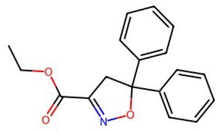	0.211

Query Compound: Oxadiazon

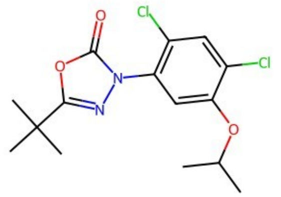

Predicted Activity: 1.0, Votes: 9.0, Docking Score: -5.57

Name	Hit2	Structure	Tanimoto Index
Sulfentrazone	0	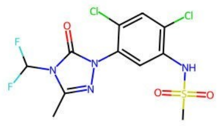	0.273
Flufenpyr-ethyl	0	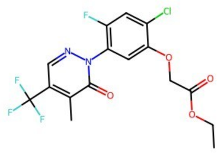	0.253

Dichlorprop	0	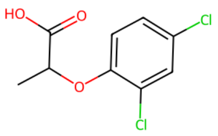	0.250
Chloroneb	0	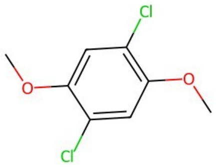	0.217
Propoxur	0	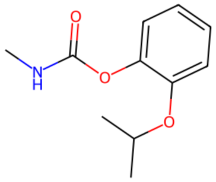	0.211

Query Compound: 3-Chloro-2-((3R)-5-chloro-1-(2,4-dimethoxybenzyl)-3-methyl- 2-oxo-2,3-dihydro-1H-indol-3-yl)-N-ethyl-N-(3-pyridinylmethyl)benzamide hydrochloride (1:1)

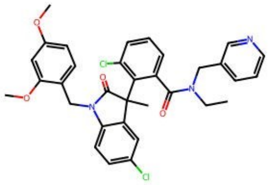

Predicted Activity: 1.0, Votes: 7.0, Docking Score: -7.73

Name	Hit2	Structure	Tanimoto Index
PharmaGSID_48510	0	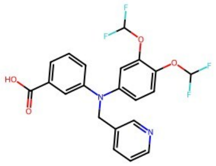	0.260

2,4-D Butyl ester	0	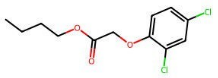	0.217
2,4-D-ethyl ester	0	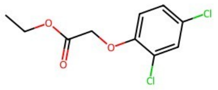	0.213
1-(p-Chlorobenzoyl)-5-methoxy-2-methyl-Indole-3-acetic acid	0	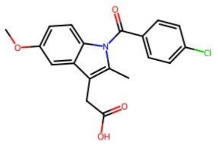	0.210
2,4-D-Butotyl	0	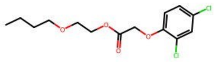	0.208

Query Compound: 1,4-Bis(N-isopropylamino)anthraquinone

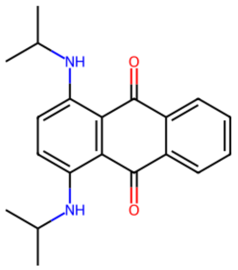

Predicted Activity: 1.0, Votes: 9.0, Docking Score: -5.56

Name	Hit2	Structure	Tanimoto Index

1,4-Diaminoanthraquinone	0	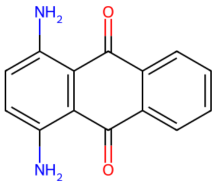 	0.433
2-Aminoanthraquinone	0	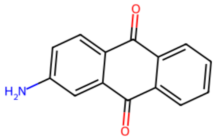 	0.324
N-Methylphthalimide	0	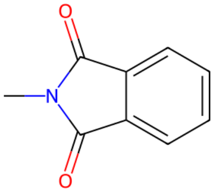 	0.323
Phthalimide	0	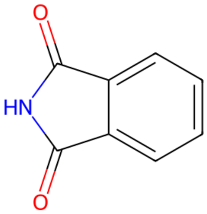 	0.300
Bentazone	0	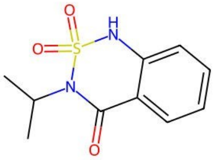 	0.286

Query Compound: Tonalide

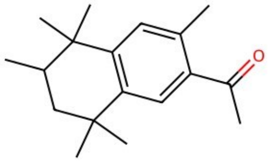

Predicted Activity: 1.0, Votes: 8.0, Docking Score: -6.63

Name	Hit2	Structure	Tanimoto Index
2-Hydroxyacetophenone	0	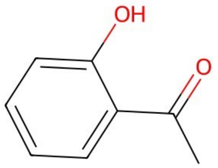	0.238
3,3,5-Trimethylcyclohexyl salicylate	0	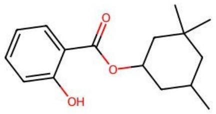	0.232
alpha-Isomethylionone	0	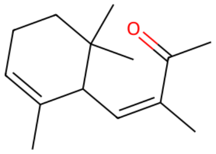	0.231
alpha-Ionone	0	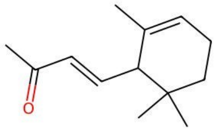	0.231
2'-Acetonaphthone	0	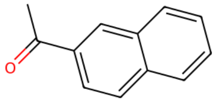	0.222

Query Compound: Profenofos

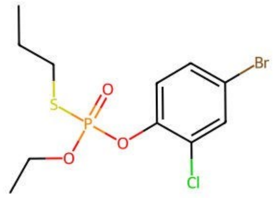

Predicted Activity: 1.0, Votes: 9.0, Docking Score: -5.52

Name	Hit2	Structure	Tanimoto Index
Ethoprop	0	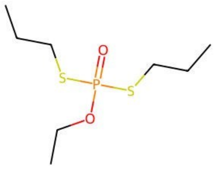	0.372
Sulprofos	1	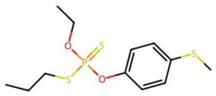	0.327
Chlorpyrifos oxon	0	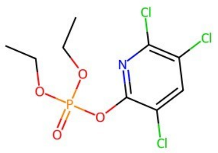	0.321
2,4-D-ethyl ester	0	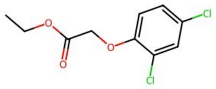	0.291
2,4-D Butyl ester	0	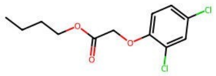	0.271

Query Compound: Chrysin

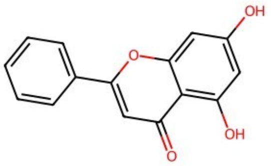

Predicted Activity: 1.0, Votes: 7.0, Docking Score: -7.66

Name	Hit2	Structure	Tanimoto Index
Morin hydrate	0	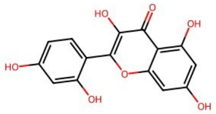	0.432
Genistein	0	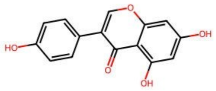	0.391
4-Phenylphenol	0	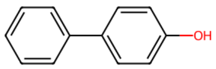	0.306
2,4-Dihydroxybenzophenone	0	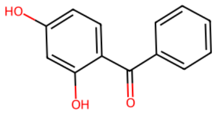	0.295
2-Phenylphenol	0	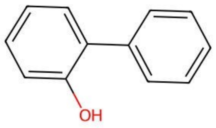	0.289

Query Compound: Bisphenol A diglycidyl ether

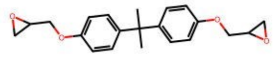

Predicted Activity: 1.0, Votes: 8.0, Docking Score: -6.55

Name	Hit2	Structure	Tanimoto Index
Butyl glycidyl ether	0	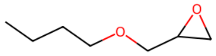	0.351
Bisphenol A	1	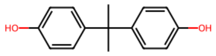	0.333
4-(2-Phenylpropan-2-yl)-N-[4-(2-phenylpropan-2-yl)phenyl]aniline	0	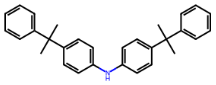	0.289
4-Ethoxyaniline	0	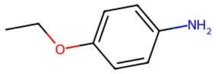	0.270

Etofenprox	0	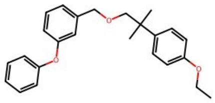	0.259

Query Compound: Imazalil

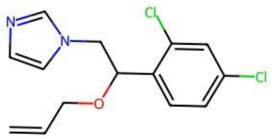

Predicted Activity: 1.0, Votes: 9.0, Docking Score: -5.42

Name	Hit2	Structure	Tanimoto Index
Econazole nitrate	1	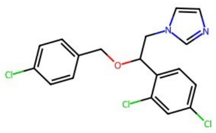	0.714
Dichlorprop	0	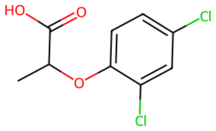	0.246
1,2,4-Trichlorobenzene	0	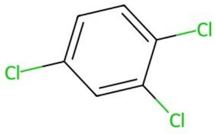	0.244

2,4-Dichlorophenoxyacetic acid	0	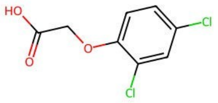	0.232
2,4-Dichlorophenol	0	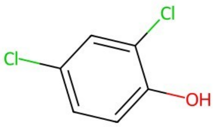	0.229

Query Compound: Chlordane

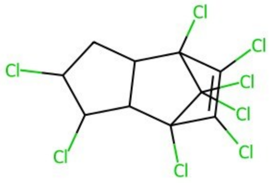

Predicted Activity: 1.0, Votes: 9.0, Docking Score: -5.41

Name	Hit2	Structure	Tanimoto Index
Chlorendic acid	0	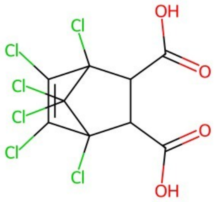	0.333
Hexachlorocyclopentadiene	1	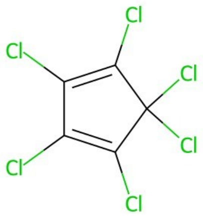	0.207

(-)-beta-Pinene	0	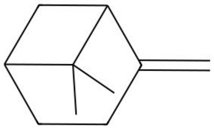	0.179
Lindane	1	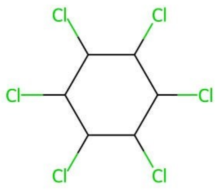	0.160
alpha-Pinene	0	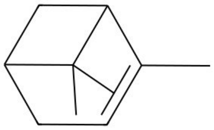	0.150

Query Compound: Nordihydroguaiaretic acid

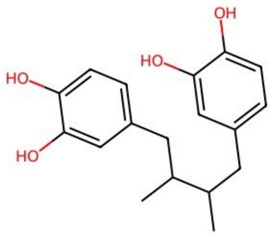

Predicted Activity: 1.0, Votes: 6.0, Docking Score: -8.6

Name	Hit2	Structure	Tanimoto Index
Methyldopa sesquihydrate	0	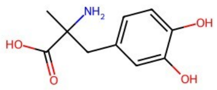	0.417

Isoproterenol hydrochloride	0	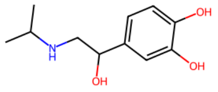	0.389
2,4-Diisopropylphenol	1	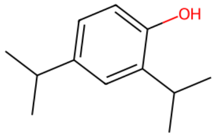	0.344
Eugenol	0	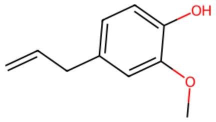	0.316
4-Ethylphenol	0	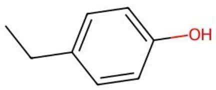	0.310

Query Compound: Pendimethalin

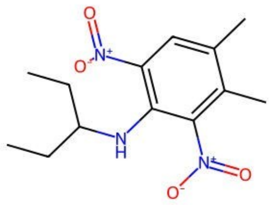

Predicted Activity: 1.0, Votes: 9.0, Docking Score: -5.25

Name	Hit2	Structure	Tanimoto Index

2,3-Dinitrotoluene	0	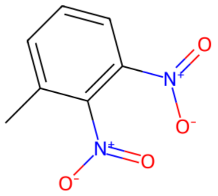	0.357
1,2-Dimethyl-3-nitrobenzene	0	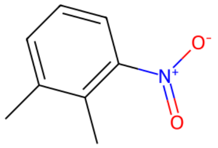	0.333
1,3-Dimethyl-4-nitrobenzene	0	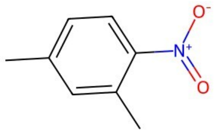	0.333
2,4-Dinitrotoluene	0	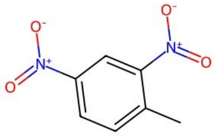	0.326
2,6-Dinitrotoluene	0	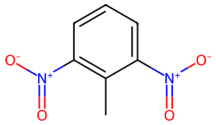	0.325

Query Compound: Darbufelone mesylate

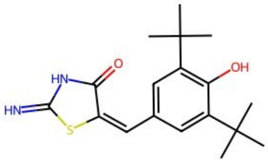

Predicted Activity: 1.0, Votes: 9.0, Docking Score: -5.23

Name	Hit2	Structure	Tanimoto Index
2,4,6-Tris(tert-butyl)phenol	0	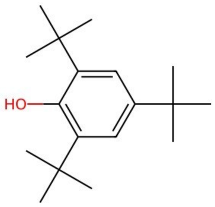	0.350
4,4'-Methylenebis(2,6-di-t-butylphenol)	0	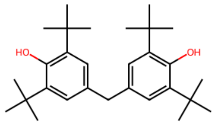	0.333
2,6-Di-tert-butyl-4-ethylphenol	1	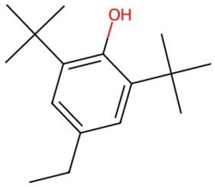	0.326
2,6-Di-tert-butyl-4-methoxyphenol	1	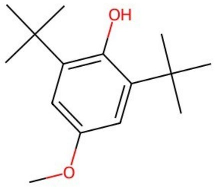	0.326
Irganox 1010	0	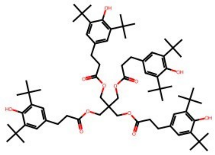	0.283

Query Compound: Parathion

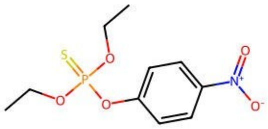

Predicted Activity: 1.0, Votes: 9.0, Docking Score: -5.22

Name	Hit2	Structure	Tanimoto Index
Paraoxon	0	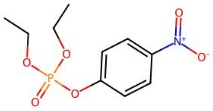	0.694
Methyl parathion	0	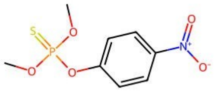	0.686
EPN	1	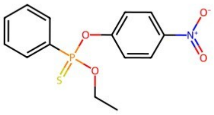	0.659
Fenitrothion	1	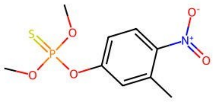	0.422
4-Nitrotoluene	0	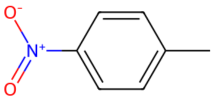	0.400

Query Compound: Heptachlor

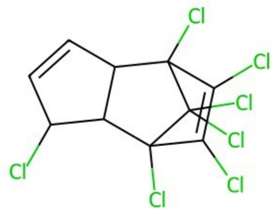

Predicted Activity: 1.0, Votes: 9.0, Docking Score: -5.19

Name	Hit2	Structure	Tanimoto Index
Chlorendic acid	0	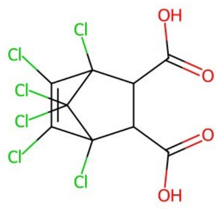	0.344
Hexachlorocyclopentadiene	1	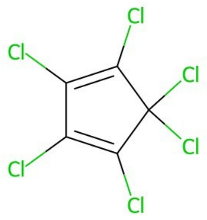	0.214
4-Chlorotoluene	0	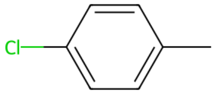	0.161
Kepone	0	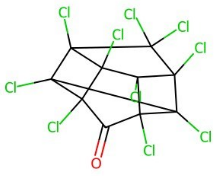	0.152
Mirex	0	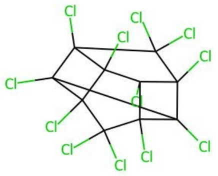	0.148

Query Compound: Endosulfan sulfate

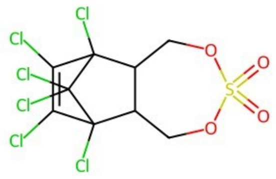

Predicted Activity: 1.0, Votes: 9.0, Docking Score: -5.17

Name	Hit2	Structure	Tanimoto Index
Chlorendic acid	0	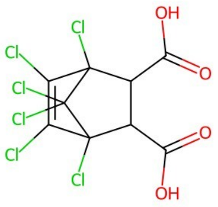	0.364
Hexachlorocyclopentadiene	1	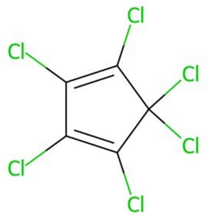	0.200
1,3-Propane sultone	0	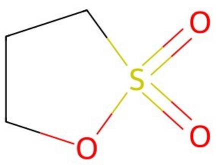	0.176
Kepone	0	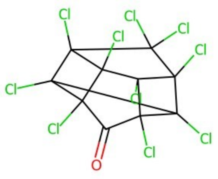	0.176

Propylene carbonate	0	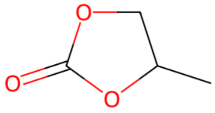	0.162

Query Compound: Endosulfan

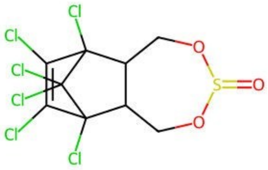

Predicted Activity: 1.0, Votes: 9.0, Docking Score: -5.17

Name	Hit2	Structure	Tanimoto Index
Chlorendic acid	0	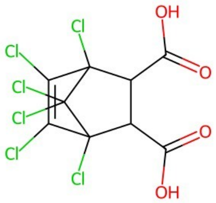	0.364
Hexachlorocyclopentadiene	1	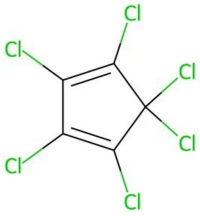	0.200
Propylene carbonate	0	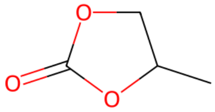	0.194

Butylene carbonate	0	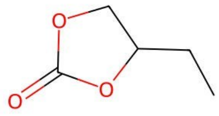	0.179
Kepone	0	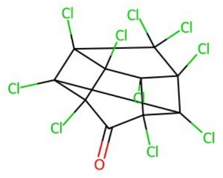	0.176

Query Compound: Tebupirimfos

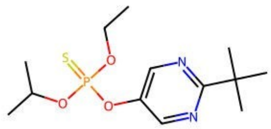

Predicted Activity: 1.0, Votes: 9.0, Docking Score: -5.14

Name	Hit2	Structure	Tanimoto Index
Diazinon	0	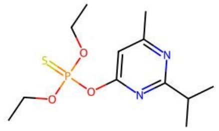	0.346
Chlorethoxyfos	1	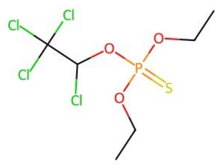	0.333

Isazofos	1	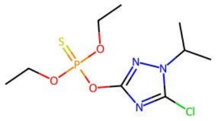	0.321
Terbufos	1	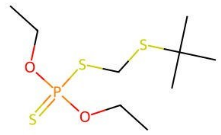	0.250
Diazoxon	0	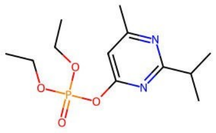	0.250

Query Compound: Bensulide

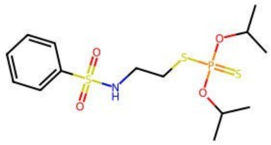

Predicted Activity: 1.0, Votes: 8.0, Docking Score: -6.16

Name	Hit2	Structure	Tanimoto Index
N-Butylbenzenesulfonamide	0	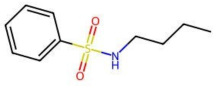	0.455

N-Butyl-p-toluenesulfonamide	0	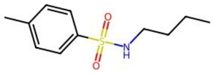	0.327
Phosmet	0	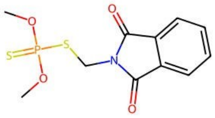	0.278
Propham	0	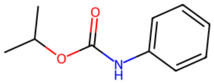	0.260
Propachlor	0	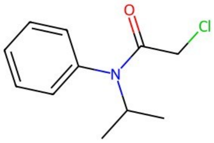	0.250

Query Compound: Bromuconazole

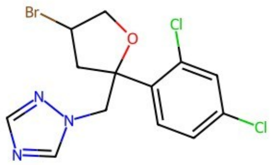

Predicted Activity: 1.0, Votes: 7.0, Docking Score: -7.04

Name	Hit2	Structure	Tanimoto Index

Triticonazole	0	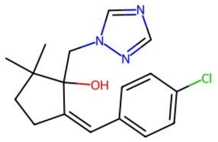	0.338
Cyproconazole	0	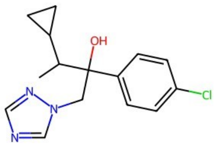	0.313
Tebuconazole	0	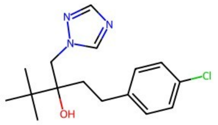	0.292
Myclobutanil	0	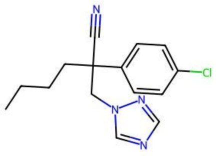	0.275
Fluconazole	0	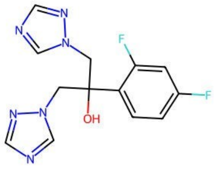	0.246

Query Compound: p-Bromodiphenyl ether

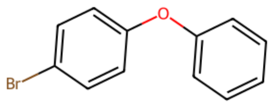

Predicted Activity: 1.0, Votes: 8.0, Docking Score: -5.92

Name	Hit2	Structure	Tanimoto Index
Diphenyl oxide	0	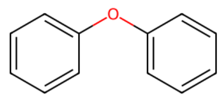	0.667
1-(Bromomethyl)-3-phenoxybenzene	1	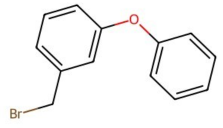	0.448
3-Phenoxybenzoic acid	0	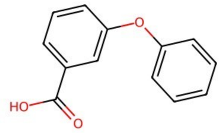	0.400
3-Phenoxybenzenemethanol	0	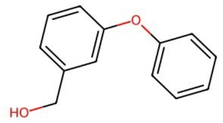	0.400
4,4'-Oxydianiline	0	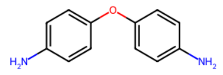	0.391

Query Compound: Chlorpyrifos

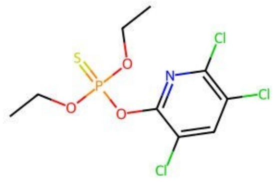

Predicted Activity: 1.0, Votes: 9.0, Docking Score: -4.8

Name	Hit2	Structure	Tanimoto Index
Chlorpyrifos-methyl	1	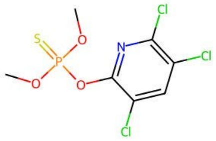	0.686
Chlorpyrifos oxon	0	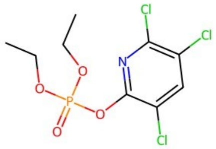	0.676
Diazinon	0	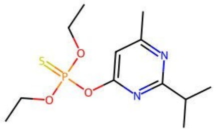	0.413
Isazofos	1	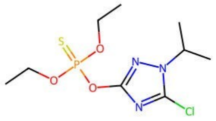	0.413
Triclopyr	0	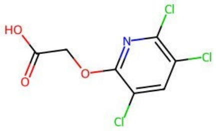	0.405

Query Compound: Benz(a)anthracene

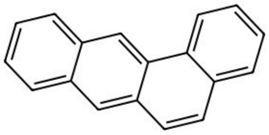

Predicted Activity: 1.0, Votes: 6.0, Docking Score: -8.03

Name	Hit2	Structure	Tanimoto Index
Dibenz(a,h)anthracene	0	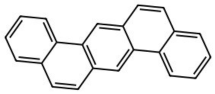	0.889
Anthracene	0	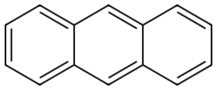	0.556
Benzo(b)fluoranthene	0	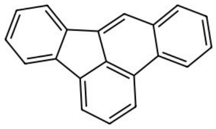	0.429
2-Naphthylamine	0	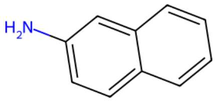	0.400
1-Naphthol	0	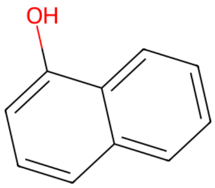	0.400

Query Compound: Ethion

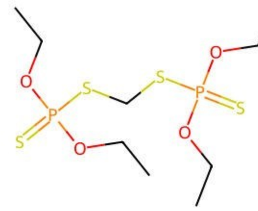

Predicted Activity: 1.0, Votes: 9.0, Docking Score: -4.74

Name	Hit2	Structure	Tanimoto Index
Terbufos	1	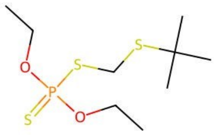	0.680
Sulprofos	1	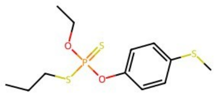	0.368
Ethoprop	0	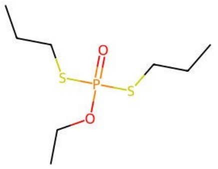	0.357
Triethyl phosphate	0	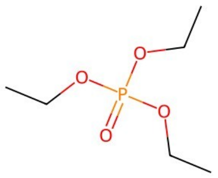	0.348

Chlorethoxyfos	1	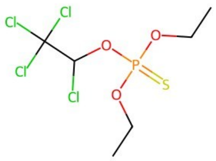	0.312

Query Compound: Mifepristone

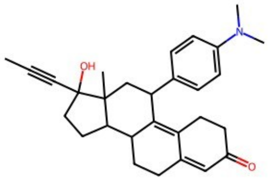

Predicted Activity: 1.0, Votes: 5.0, Docking Score: -9.03

Name	Hit2	Structure	Tanimoto Index
17beta-Trenbolone	0	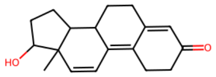	0.333
Norethindrone	0	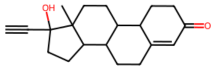	0.321
Levonorgestrel	0	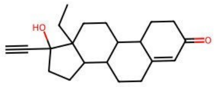	0.277

17-Methyltestosterone	0	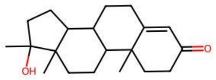	0.260
Mestranol	0	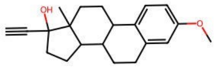	0.247

Query Compound: HMR1171 trifluoroacetate (1:1)

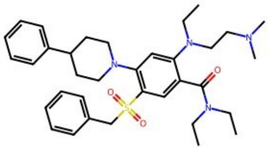

Predicted Activity: 1.0, Votes: 4.0, Docking Score: -10.02

Name	Hit2	Structure	Tanimoto Index
MK-578	0	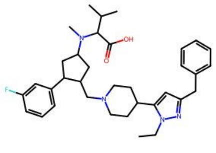	0.260
DEET	0	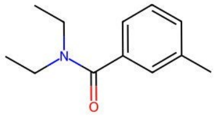	0.243

Butam	0	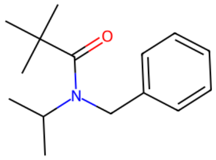	0.236
FD&C; Blue No. 1	0	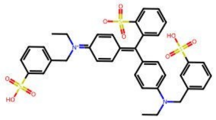	0.228
alpha,alpha-Dimethylphenethyl butyrate	0	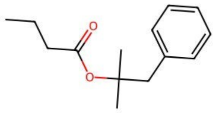	0.216

Query Compound: Ro 23-7637

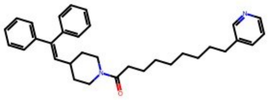

Predicted Activity: 1.0, Votes: 4.0, Docking Score: -9.98

Name	Hit2	Structure	Tanimoto Index
Nicotinic acid	0	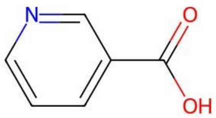	0.315

UK-156819	0	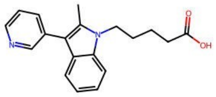	0.288
Niacinamide	0	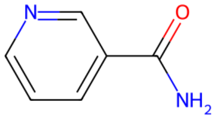	0.286
PharmaGSID_48510	0	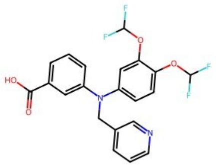	0.282
Heptanophenone	0	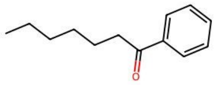	0.271

Query Compound: Prallethrin

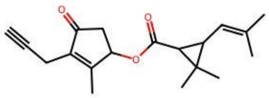

Predicted Activity: 1.0, Votes: 8.0, Docking Score: -5.61

Name	Hit2	Structure	Tanimoto Index

Phenothrin	0	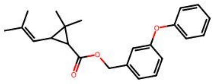	0.343
Resmethrin	0	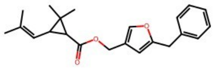	0.329
Tefluthrin	0	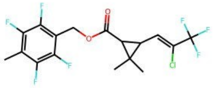	0.294
Permethrin	0	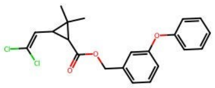	0.268
Bifenthrin	0	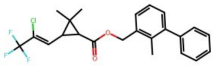	0.263

Query Compound: Flutamide

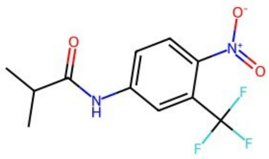

Predicted Activity: 1.0, Votes: 7.0, Docking Score: -6.62

Name	Hit2	Structure	Tanimoto Index
Hydroxyflutamide	0	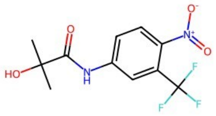	0.682
5,5-Dimethyl-3-(alpha,alpha,alpha-trifluoro-4-nitro-m-tolyl)hydantoin	0	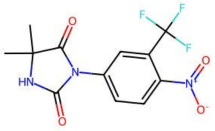	0.400
Flutolanil	1	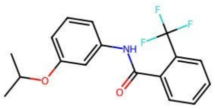	0.357
1,3-Dimethyl-4-nitrobenzene	0	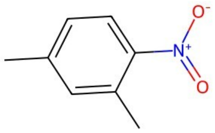	0.311
Fomesafen	0	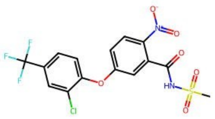	0.309

Query Compound: MK-968

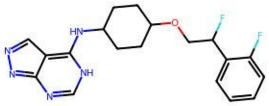

Predicted Activity: 1.0, Votes: 5.0, Docking Score: -8.63

Name	Hit2	Structure	Tanimoto Index
N-Benzyladenine	0	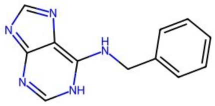	0.239
Kinetin	0	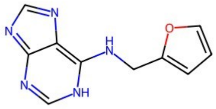	0.211
Etoxazole	1	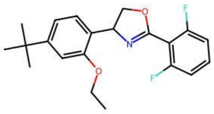	0.188
CP-457920	0	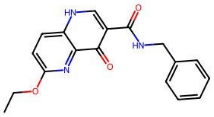	0.184
SAR 150640	0	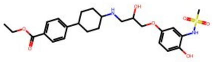	0.160

Query Compound: Farglitazar

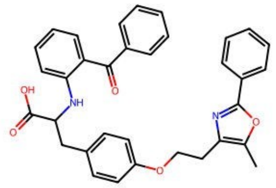

Predicted Activity: 1.0, Votes: 4.0, Docking Score: -9.65

Name	Hit2	Structure	Tanimoto Index
PD-0333941	0	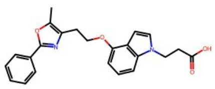	0.412
Octabenzone	0	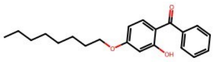	0.311
2-Hydroxy-4-methoxybenzophenone	1	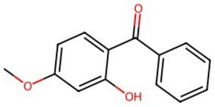	0.271
CP-114271	0	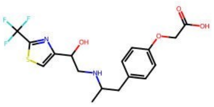	0.258
2-Hydroxybenzophenone	0	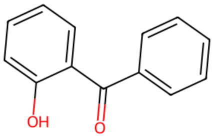	0.250

Query Compound: 1H,1H,2H,2H-Perfluorooctyl iodide

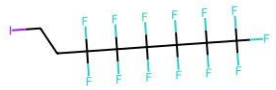

Predicted Activity: 1.0, Votes: 8.0, Docking Score: -5.31

Name	Hit2	Structure	Tanimoto Index
3,3,4,4,5,5,6,6,7,7,8,8,8-Tridecafluorooctanol	0	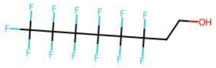	0.565
3,3,4,4,5,5,6,6,7,7,8,8,9,9,10,10,10-Heptadecafluoro-1-decanol	0	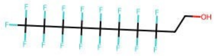	0.565
3,3,4,4,5,5,6,6,7,7,8,8,8-Tridecafluorooctyl methacrylate	0	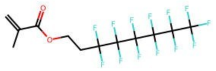	0.371
Perfluorodecanoic acid	0	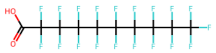	0.296

Perfluorononanoic acid	0	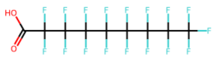	0.296

Query Compound: PharmaGSID_47337

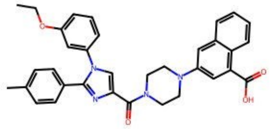

Predicted Activity: 1.0, Votes: 4.0, Docking Score: -9.52

Name	Hit2	Structure	Tanimoto Index
CP-422935	0	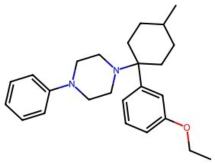	0.321
Ethyl 1-naphthaleneacetate	1	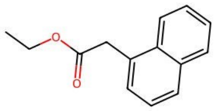	0.250
Desmedipham	0	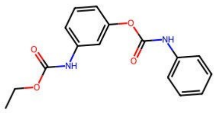	0.235

CP-457920	0	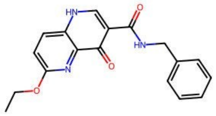	0.215
PD-0333941	0	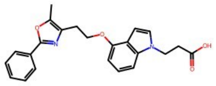	0.214

Query Compound: SAR102779

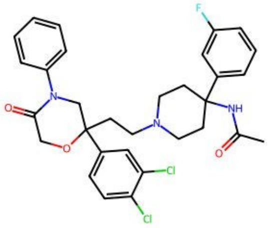

Predicted Activity: 1.0, Votes: 3.0, Docking Score: -10.57

Name	Hit2	Structure	Tanimoto Index
SR144190	0	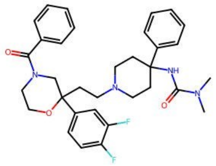	0.430
SSR146977	0	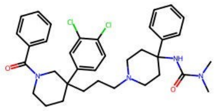	0.409

Haloperidol	0	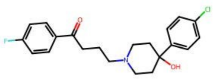	0.261
MK-578	0	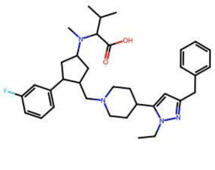	0.219
CP-607366	0	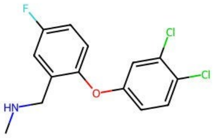	0.209

Query Compound: Propargite

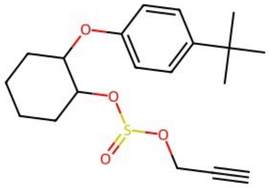

Predicted Activity: 1.0, Votes: 7.0, Docking Score: -6.17

Name	Hit2	Structure	Tanimoto Index
4,4'-Di-tert-butylbiphenyl	0	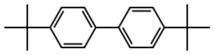	0.239

2-tert-Butylcyclohexyl acetate	0	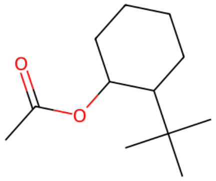	0.236
4-tert-Butyltoluene	0	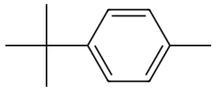	0.234
2-(4-Tert-Butylbenzyl)propionaldehyde	0	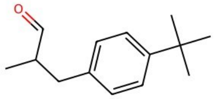	0.232
4-tert-Butylbenzenethiol	0	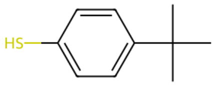	0.229

Query Compound: Thiobencarb

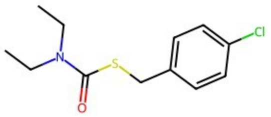

Predicted Activity: 1.0, Votes: 7.0, Docking Score: -6.12

Name	Hit2	Structure	Tanimoto Index

4-Butylchlorobenzene	0	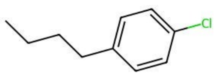	0.400
Ethyl 4-chlorophenyl ketone	0	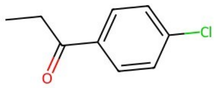	0.389
4-Chloropentylbenzene	0	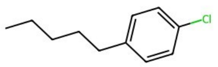	0.389
Pebulate	0	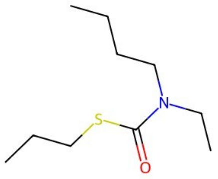	0.350
Monuron	0	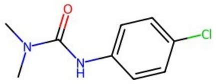	0.325

Query Compound: N-Ethylperfluorooctanesulfonamide

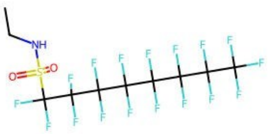

Predicted Activity: 1.0, Votes: 9.0, Docking Score: -3.85

Name	Hit2	Structure	Tanimoto Index
Perfluorooctanesulfonic acid	1	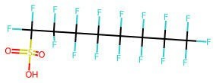	0.448
Potassium perfluorohexanesulfonate	1	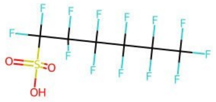	0.448
Potassium perfluorobutanesulfonate	0	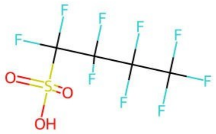	0.414
Perfluoroheptanoic acid	0	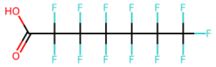	0.281
Perfluorononanoic acid	0	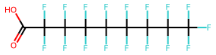	0.281

Query Compound: MK-274

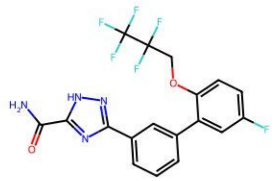

Predicted Activity: 1.0, Votes: 6.0, Docking Score: -7.02

Name	Hit2	Structure	Tanimoto Index
Trifloxysulfuron-sodium	0	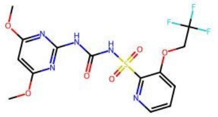	0.238
PharmaGSID_48507	0	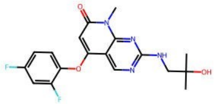	0.221
AVE3295	0	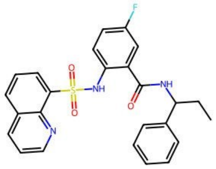	0.220
CP-085958	0	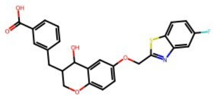	0.212
Flufenacet	0	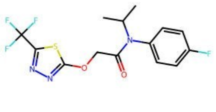	0.210

Query Compound: PharmaGSID_48505

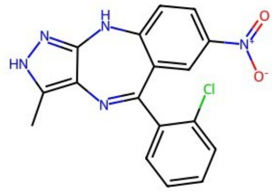

Predicted Activity: 1.0, Votes: 5.0, Docking Score: -8.08

Name	Hit2	Structure	Tanimoto Index
3,4-Dichloronitrobenzene	0	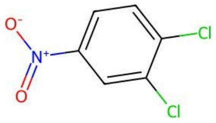	0.327
1-Chloro-2,4-dinitrobenzene	0	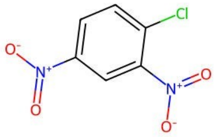	0.315
1,2-Dimethyl-4-nitrobenzene	0	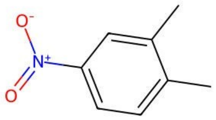	0.302
3-Nitrotoluene	0	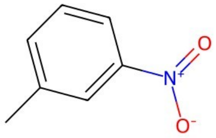	0.296
2,4-Dinitrotoluene	0	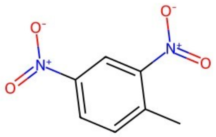	0.291

Query Compound: PharmaGSID_47330

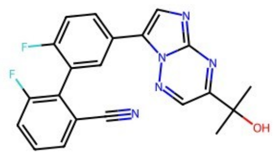

Predicted Activity: 1.0, Votes: 4.0, Docking Score: -9.15

Name	Hit2	Structure	Tanimoto Index
Etoxazole	1	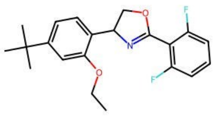	0.202
2-Hydroxybenzonitrile	0	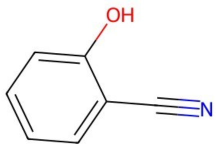	0.197
PharmaGSID_48507	0	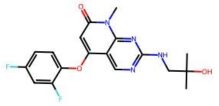	0.189
CP-863187	0	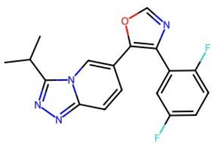	0.186

Azoxystrobin	1	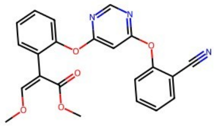	0.184

Query Compound: CJ-013610

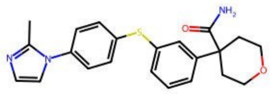

Predicted Activity: 1.0, Votes: 3.0, Docking Score: -10.22

Name	Hit2	Structure	Tanimoto Index
4,5-dihydro-5,5-diphenyl-1,2-oxazole-3-carboxylic acid, ethyl ester	0	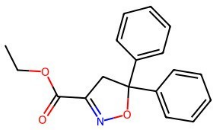	0.203
CP-422935	0	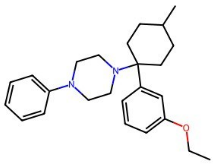	0.193
Dimethomorph	0	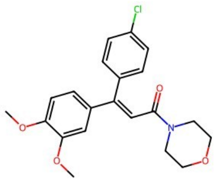	0.185

CP-544439	0	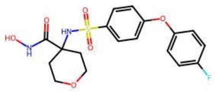	0.185
Ethyl methylphenylglycidate	0	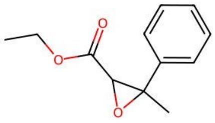	0.181

Query Compound: PharmaGSID_48514

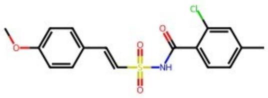

Predicted Activity: 1.0, Votes: 6.0, Docking Score: -6.82

Name	Hit2	Structure	Tanimoto Index
PharmaGSID_48516	0	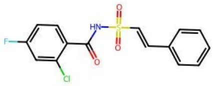	0.556
PharmaGSID_48518	0	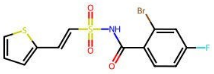	0.333

(E)-Anethole	0	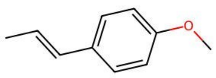	0.333
4-Methoxybenzaldehyde	0	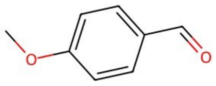	0.312
2-Ethylhexyl trans-4-methoxycinnama te	0	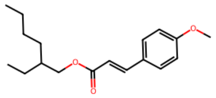	0.288

Query Compound: Cinmethylin

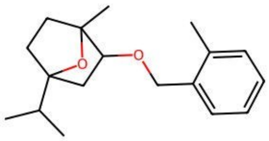

Predicted Activity: 1.0, Votes: 6.0, Docking Score: -6.76

Name	Hit2	Structure	Tanimoto Index
Ethyl methylphenylglycidate	0	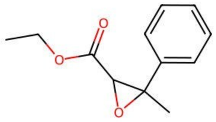	0.220

2-Ethyl-6-methylaniline	0	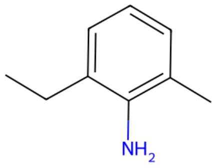	0.220
Clomazone	0	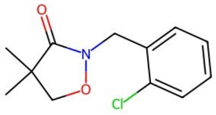	0.213
Kresoxim-methyl	1	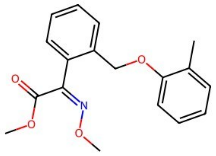	0.212
Metolachlor	0	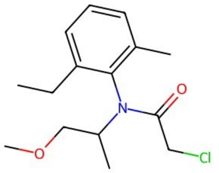	0.212

Query Compound: Phenanthrene

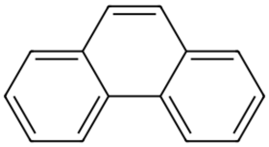

Predicted Activity: 1.0, Votes: 5.0, Docking Score: -7.8

Name	Hit2	Structure	Tanimoto Index

Dibenz(a,h)anthracene	0	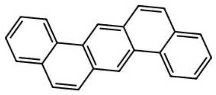	0.750
Naphthalene	0	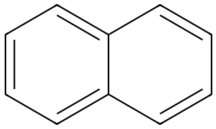	0.538
1-Naphthol	0	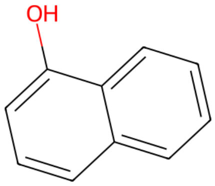	0.526
1-Methylnaphthalene	0	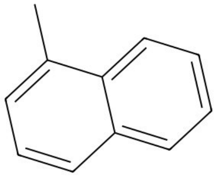	0.526
Anthracene	0	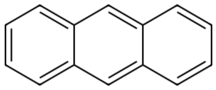	0.467

Query Compound: Ipconazole

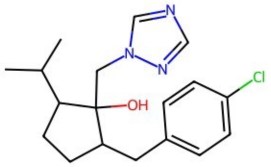

Predicted Activity: 1.0, Votes: 5.0, Docking Score: -7.75

Name	Hit2	Structure	Tanimoto Index
Cyproconazole	0	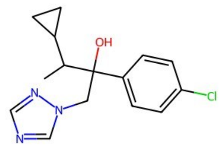	0.435
Triticonazole	0	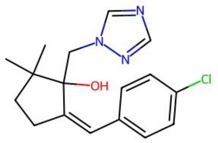	0.415
Tebuconazole	0	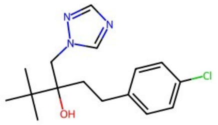	0.393
Paclobutrazol	0	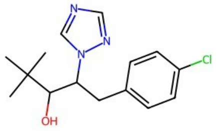	0.349
Myclobutanil	0	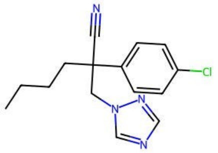	0.309

Query Compound: Pentachlorophenol

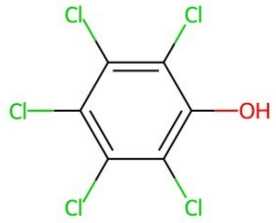

Predicted Activity: 1.0, Votes: 7.0, Docking Score: -5.38

Name	Hit2	Structure	Tanimoto Index
2,4,6-Trichlorophenol	0	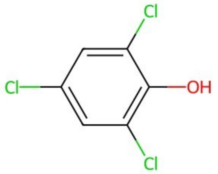	0.333
Pentachloropyridine	0	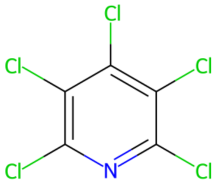	0.312
4-Chloro-3,5-dimethylphenol	0	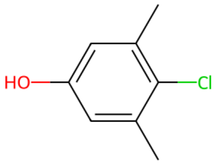	0.300
4-Chlorophenol	0	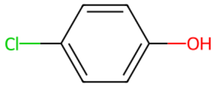	0.278
Pentachloroanisole	0	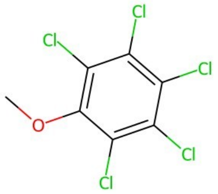	0.278

Query Compound: Pirimiphos-methyl

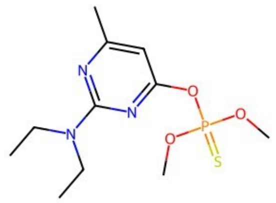

Predicted Activity: 1.0, Votes: 7.0, Docking Score: -5.13

Name	Hit2	Structure	Tanimoto Index
Diazinon	0	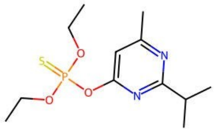	0.447
Chlorpyrifos-methyl	1	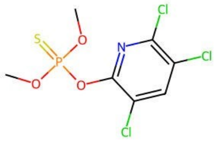	0.348
Diazoxon	0	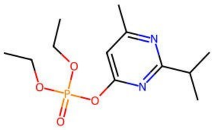	0.333
Fenitrothion	1	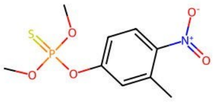	0.288
Methyl parathion	0	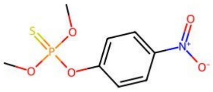	0.265

Query Compound: Captan

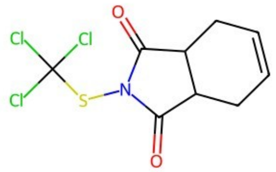

Predicted Activity: 1.0, Votes: 6.0, Docking Score: -6.19

Name	Hit2	Structure	Tanimoto Index
1,2,3,6-Tetrahydrophthalimide	0	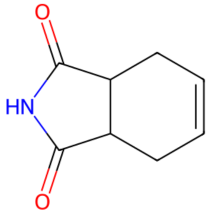	0.355
Symclosene	0	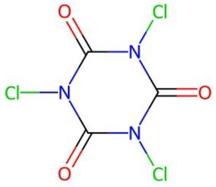	0.167
Procymidone	0	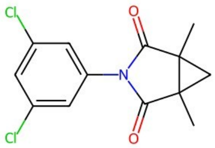	0.163
Propylene carbonate	0	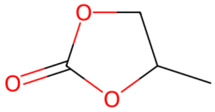	0.162

Tris(2,3-epoxypropyl)isocyanurate	0	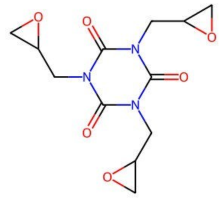	0.162

Query Compound: Phorate

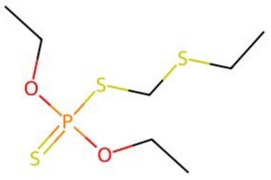

Predicted Activity: 1.0, Votes: 8.0, Docking Score: -3.96

Name	Hit2	Structure	Tanimoto Index
Terbufos	1	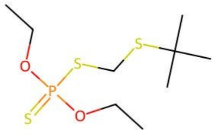	0.607
Sulprofos	1	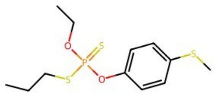	0.341
Ethoprop	0	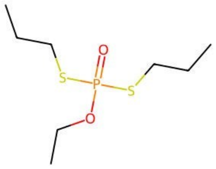	0.323

Triethyl phosphate	0	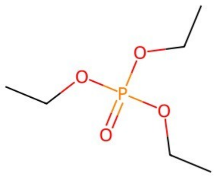	0.308
Chlorethoxyfos	1	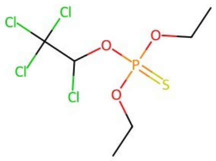	0.286

Query Compound: Tribufos

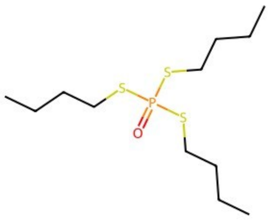

Predicted Activity: 1.0, Votes: 7.0, Docking Score: -4.98

Name	Hit2	Structure	Tanimoto Index
Ethoprop	0	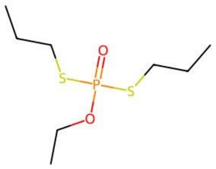	0.423
Tributyl phosphate	0	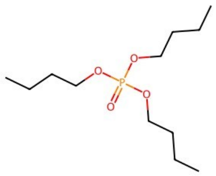	0.360

Pentanal	0	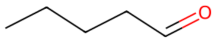	0.348
Decanal	0	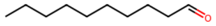	0.320
Octanal	0	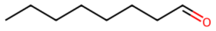	0.320

Query Compound: PD 0343701

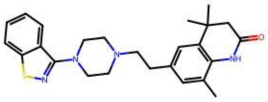

Predicted Activity: 1.0, Votes: 6.0, Docking Score: -5.99

Name	Hit2	Structure	Tanimoto Index
Trelanserin	0	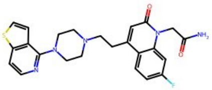	0.260

Clomazone	0	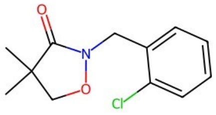	0.195
(2S,3S)-3-Methyl-2-(3-oxo-1,2-benzothiazol-2(3H)-yl)pentanoic acid	0	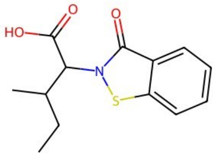	0.188
Volinanserin	0	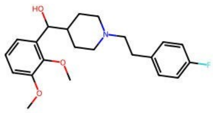	0.180
Zamifenacin	1	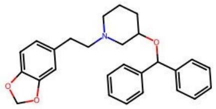	0.176

Query Compound: Acetylcedrene

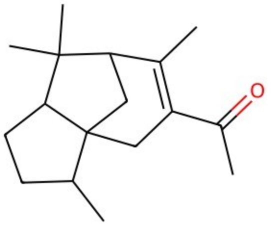

Predicted Activity: 1.0, Votes: 5.0, Docking Score: -7.02

Name	Hit2	Structure	Tanimoto Index

Dehydroacetic acid	0	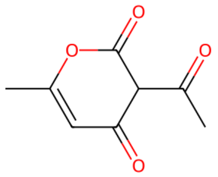	0.204
alpha-Isomethylionone	0	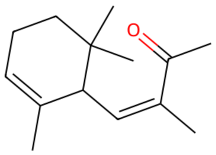	0.204
alpha-Ionone	0	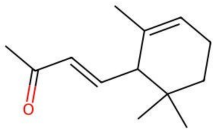	0.204
Sodium abietate	0	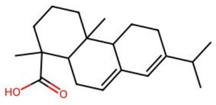	0.203
Fenchol	0	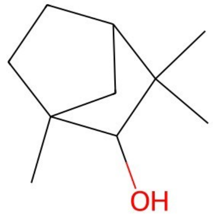	0.196

Query Compound: Benzyl butyl phthalate

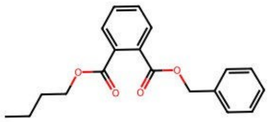

Predicted Activity: 1.0, Votes: 5.0, Docking Score: -6.98

Name	Hit2	Structure	Tanimoto Index
Monobutyl phthalate	0	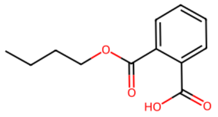	0.743
Dipentyl phthalate	0	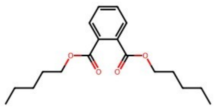	0.706
Di-n-octyl phthalate	0	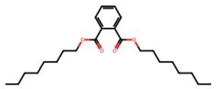	0.686
Dihexyl phthalate	0	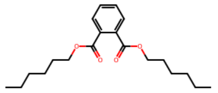	0.686
Octyl decyl phthalate	0	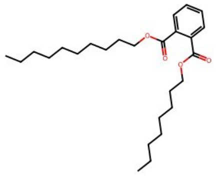	0.686

Query Compound: Di(propylene glycol) dibenzoate

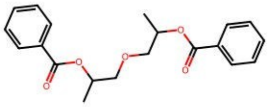

Predicted Activity: 1.0, Votes: 5.0, Docking Score: -6.89

Name	Hit2	Structure	Tanimoto Index
Isopentyl benzoate	0	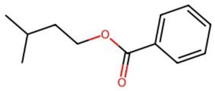	0.500
Ethyl benzoate	0	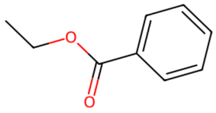	0.485
Diethylene glycol dibenzoate	0	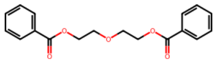	0.471
Ethylenebis(oxyethylene) dibenzoate	0	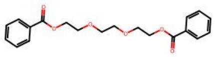	0.471
Isopropyl phenyl ketone	0	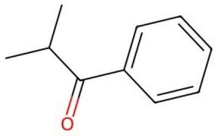	0.438

Query Compound: Methyl abietate

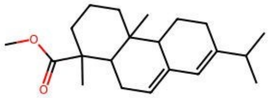

Predicted Activity: 1.0, Votes: 5.0, Docking Score: -6.88

Name	Hit2	Structure	Tanimoto Index
Sodium abietate	0	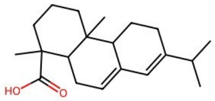	0.780
17beta-Trenbolone	0	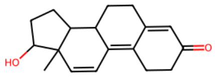	0.225
alpha-Terpinyl acetate	0	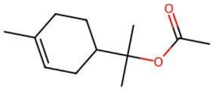	0.222
Terpinyl propionate	0	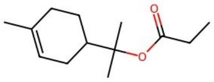	0.212
Ergocalciferol	0	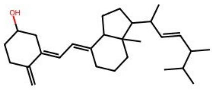	0.210

Query Compound: Disulfoton

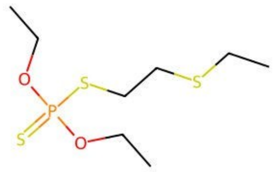

Predicted Activity: 1.0, Votes: 9.0, Docking Score: -2.45

Name	Hit2	Structure	Tanimoto Index
Terbufos	1	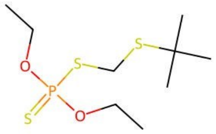	0.516
Sulprofos	1	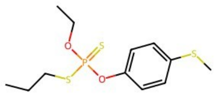	0.390
Ethoprop	0	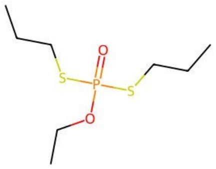	0.344
Triethyl phosphate	0	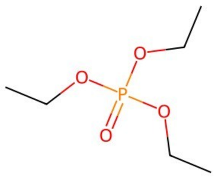	0.286

Chlorethoxyfos	1	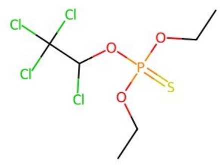	0.270

Query Compound: PharmaGSID_48506

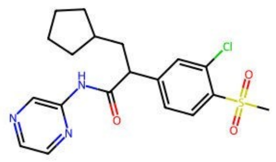

Predicted Activity: 1.0, Votes: 4.0, Docking Score: -7.44

Name	Hit2	Structure	Tanimoto Index
Piragliatin	0	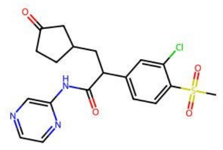	0.797
Isoxaflutole	0	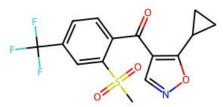	0.238
SR146131 trifluoroacetate (1:1)	0	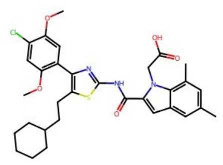	0.208

Propanil	0	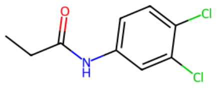	0.206
Haloxyfop-methyl	0	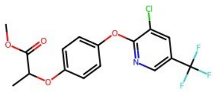	0.205

Query Compound: SSR150106

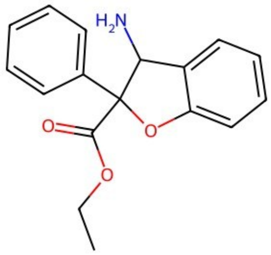

Predicted Activity: 1.0, Votes: 5.0, Docking Score: -6.31

Name	Hit2	Structure	Tanimoto Index
Ethyl methylphenylglycidate	0	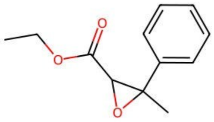	0.420
4,5-dihydro-5,5-diphenyl-1,2-oxazole-3-carboxylic acid, ethyl ester	0	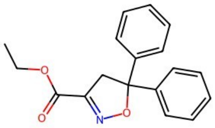	0.364

Ethyl 2,3-epoxy-3-phenylpropionate	0	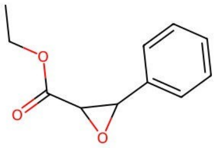	0.360
Ethyl benzoate	0	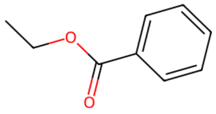	0.356
Diethyl phthalate	0	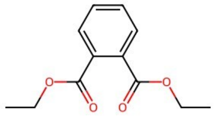	0.333

Query Compound: SB236057A

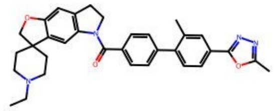

Predicted Activity: 1.0, Votes: 3.0, Docking Score: -8.42

Name	Hit2	Structure	Tanimoto Index
SB413217A	0	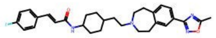	0.216

SR144190	0	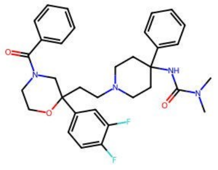	0.200
YM 218	0	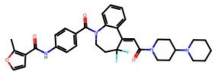	0.200
CI-1018	0	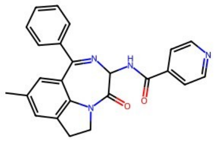	0.183
Surinabant	0	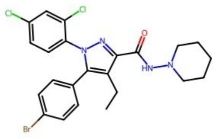	0.183

Query Compound: Metconazole

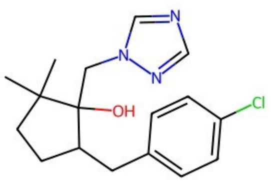

Predicted Activity: 1.0, Votes: 4.0, Docking Score: -7.32

Name	Hit2	Structure	Tanimoto Index

Triticonazole	0	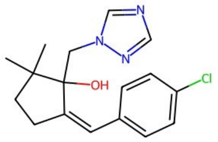	0.500
Tebuconazole	0	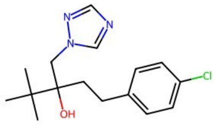	0.407
Cyproconazole	0	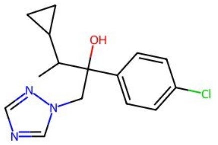	0.403
Paclobutrazol	0	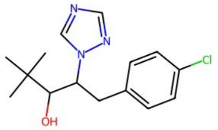	0.361
Myclobutanil	0	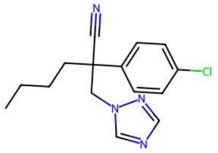	0.318

Query Compound: PharmaGSID_48519

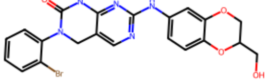

Predicted Activity: 1.0, Votes: 3.0, Docking Score: -8.37

Name	Hit2	Structure	Tanimoto Index
Benoxacor	0	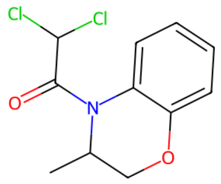	0.217
Cyprodinil	1	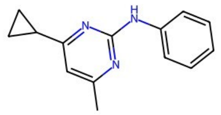	0.210
PHA-00568487	0	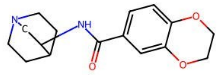	0.205
2',3'-Dideoxyinosine	0	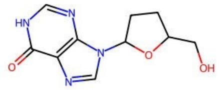	0.202
Pyrimethanil	0	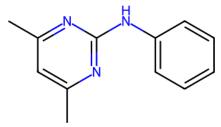	0.187

Query Compound: Sodium dodecyl sulfate

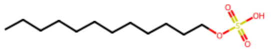

Predicted Activity: 1.0, Votes: 8.0, Docking Score: -2.73

Name	Hit2	Structure	Tanimoto Index
Sodium nonyl sulfate	1	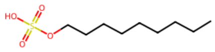	1.000
Octadecyl sulfate sodium salt	0	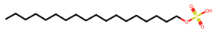	1.000
Sodium decyl sulfate	1	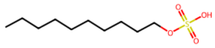	1.000
Sodium octyl sulfate	1	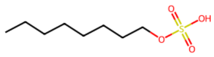	1.000
Sodium ethasulfate	0	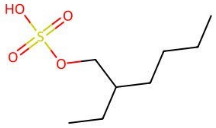	0.469

Query Compound: Ketoconazole

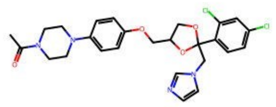

Predicted Activity: 1.0, Votes: 2.0, Docking Score: -9.0

Name	Hit2	Structure	Tanimoto Index
Econazole nitrate	1	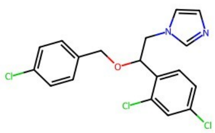	0.294
4-Chlorophenoxyacetic acid	0	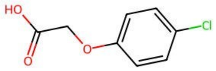	0.230
2,4-D Butyl ester	0	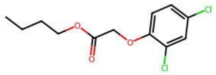	0.224
Dimethomorph	0	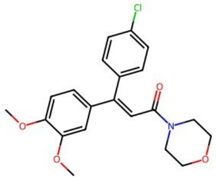	0.220
2,4-D-ethyl ester	0	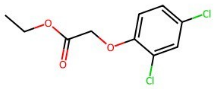	0.220

Query Compound: SR58611

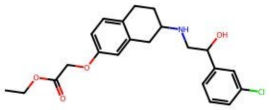

Predicted Activity: 1.0, Votes: 1.0, Docking Score: -9.83

Name	Hit2	Structure	Tanimoto Index
2,4-D-ethyl ester	0	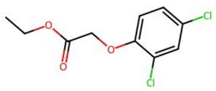	0.358
SAR 150640	0	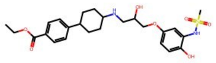	0.319
2,4-D-Butotyl	0	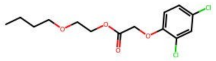	0.303
2,4-D Butyl ester	0	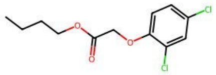	0.301

Triclopyr butotyl	0	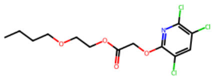	0.282

Query Compound: AVE6324

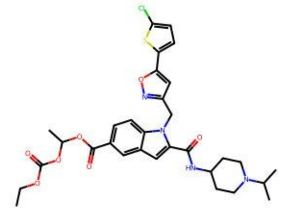

Predicted Activity: 1.0, Votes: 2.0, Docking Score: -8.5

Name	Hit2	Structure	Tanimoto Index
AVE3247	0	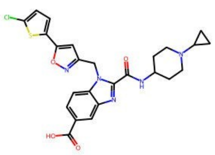	0.490
SAR 150640	0	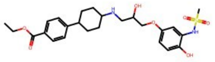	0.217
SR146131 trifluoroacetate (1:1)	0	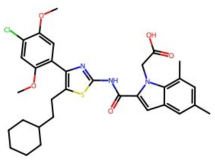	0.206

SB413217A	0	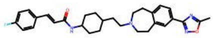	0.205
SAR115740	1	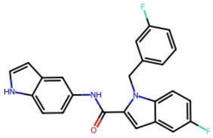	0.200

Query Compound: 4-Hexylresorcinol

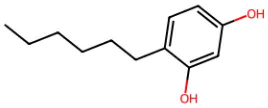

Predicted Activity: 1.0, Votes: 4.0, Docking Score: -6.24

Name	Hit2	Structure	Tanimoto Index
4-Heptylphenol	0	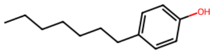	0.548
4-Dodecylphenol	0	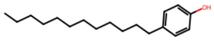	0.548

4-Octylphenol	0	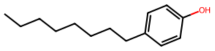	0.548
4-Nonylphenol	0	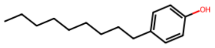	0.548
4-Butylphenol	0	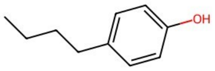	0.438

Query Compound: C.I. Acid Orange 156

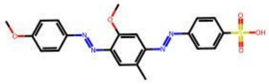

Predicted Activity: 1.0, Votes: 3.0, Docking Score: -7.28

Name	Hit2	Structure	Tanimoto Index
Allura Red C.I.16035	0	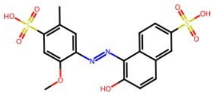	0.475

FD&C; Yellow 6	0	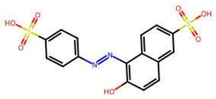	0.364
FD&C; Red 4	0	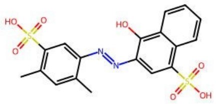	0.357
C.I. Acid Orange 8, monosodium salt	0	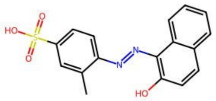	0.350
Sodium 4-methylbenzenesulfonate	0	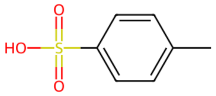	0.349

Query Compound: SSR 241586 HCl

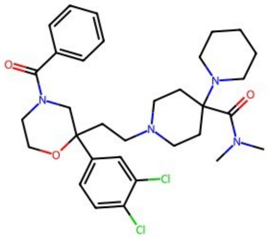

Predicted Activity: 1.0, Votes: 2.0, Docking Score: -8.29

Name	Hit2	Structure	Tanimoto Index

SR144190	0	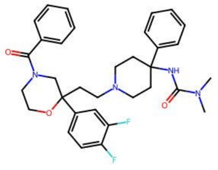	0.560
SSR146977	0	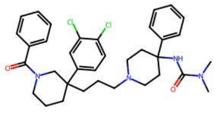	0.500
Haloperidol	0	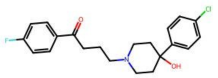	0.239
Diuron	0	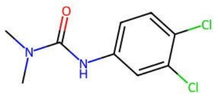	0.224
Spirodiclofen	0	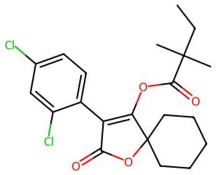	0.206

Query Compound: Heptyl p-hydroxybenzoate

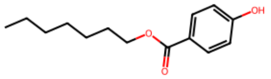

Predicted Activity: 1.0, Votes: 6.0, Docking Score: -3.79

Name	Hit2	Structure	Tanimoto Index
Octylparaben	0	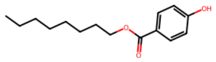	1.000
Butylparaben	0	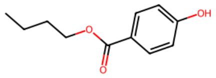	0.867
Propylparaben	0	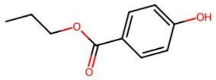	0.742
Hexyl benzoate	0	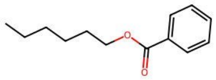	0.727
Tri-n-octyl trimellitate	0	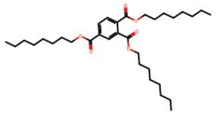	0.649

Query Compound: Fluthiacet-methyl

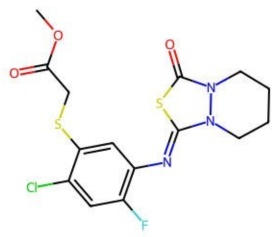

Predicted Activity: 1.0, Votes: 3.0, Docking Score: -6.72

Name	Hit2	Structure	Tanimoto Index
Flufenpyr-ethyl	0	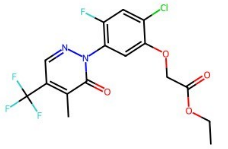	0.218
Pyraflufen-ethyl	0	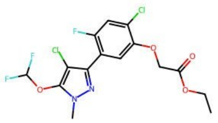	0.205
Surinabant	0	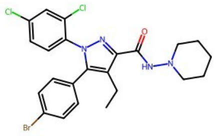	0.198
AVE2865	0	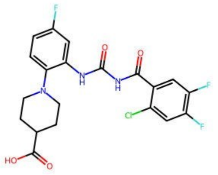	0.195
Propanil	0	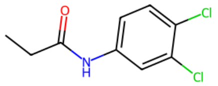	0.194

Query Compound: Farnesol

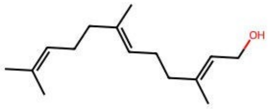

Predicted Activity: 1.0, Votes: 6.0, Docking Score: -3.39

Name	Hit2	Structure	Tanimoto Index
Geraniol	0	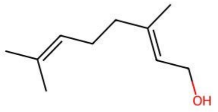	0.950
3,7-Dimethyl-2,6-octadienal	0	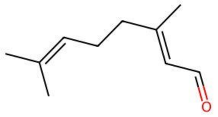	0.519
Geranyl acetate	0	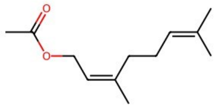	0.500
Nerolidol	0	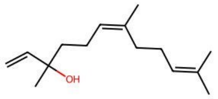	0.500
Citralva	0	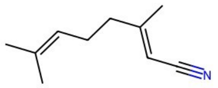	0.500

Query Compound: C.I. Acid Red 114

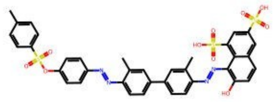

Predicted Activity: 1.0, Votes: 0.0, Docking Score: -9.84

Name	Hit2	Structure	Tanimoto Index
C.I. Acid Orange 10	0	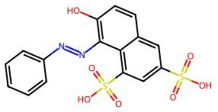	0.538
C.I. Acid Orange 8, monosodium salt	0	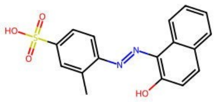	0.437
FD&C; Yellow 6	0	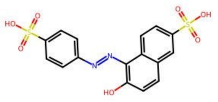	0.433
Allura Red C.I.16035	0	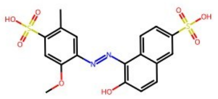	0.421

C.I. Acid Orange 7	0	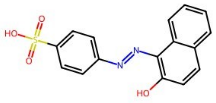	0.371

Query Compound: Testosterone propionate

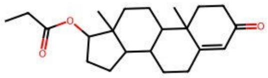

Predicted Activity: 1.0, Votes: 3.0, Docking Score: -6.56

Name	Hit2	Structure	Tanimoto Index
4-Androstene-3,17-dione	0	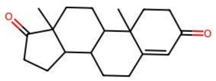	0.547
17-Methyltestosterone	0	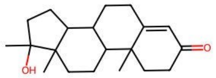	0.536
17alpha-Hydroxyprogesterone	0	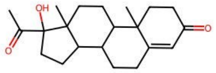	0.525

Corticosterone	0	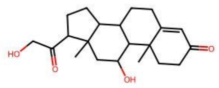	0.470
Isobornyl propanoate	0	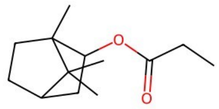	0.339

Query Compound: Didecylmethyl(3-(trimethoxysilyl)propyl)ammonium chloride

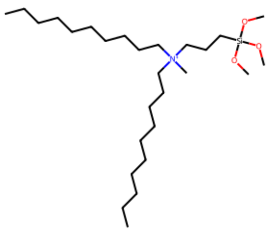

Predicted Activity: 1.0, Votes: 2.0, Docking Score: -7.64

Name	Hit2	Structure	Tanimoto Index
N,N-Dibutyl-N-methylbutan-1-aminium chloride	0	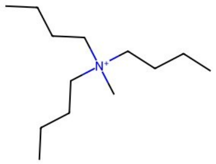	0.462
Trimethoxypropylsilane	0	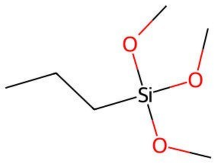	0.444

Triethoxyoctylsilane	0	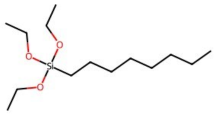	0.419
N,N-Dimethyldecylamine oxide	0	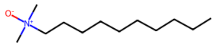	0.400
Triethoxypentylsilane	0	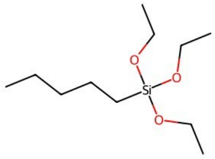	0.387

Query Compound: Chlorhexidine diacetate

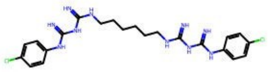

Predicted Activity: 1.0, Votes: 3.0, Docking Score: -6.54

Name	Hit2	Structure	Tanimoto Index
4-Chlorophenylurea	0	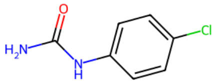	0.378

Monuron	0	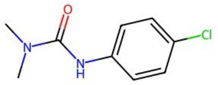	0.350
1,3-Diphenylguanidine	0	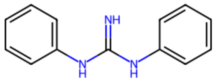	0.343
Triclocarban	1	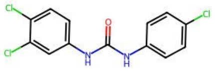	0.333
Diflubenzuron	0	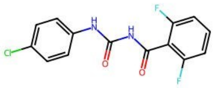	0.319

Query Compound: Diclosulam

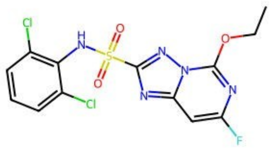

Predicted Activity: 1.0, Votes: 4.0, Docking Score: -5.4

Name	Hit2	Structure	Tanimoto Index

Flumetsulam	0	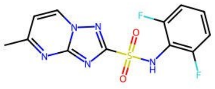	0.338
Penoxsulam	0	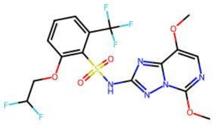	0.265
AVE3295	0	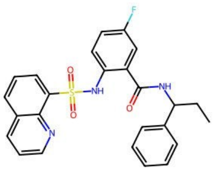	0.247
Propoxycarbazone-sodium	0	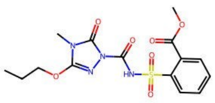	0.222
Flumetralin	1	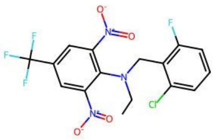	0.221

Query Compound: 2-Ethylhexyl p-hydroxybenzoate

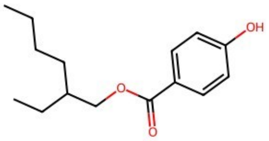

Predicted Activity: 1.0, Votes: 5.0, Docking Score: -4.31

Name	Hit2	Structure	Tanimoto Index
Bis(2-ethylhexyl) terephthalate	0	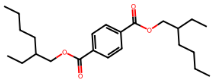	0.882
2-Ethylhexyl 4-(dimethylamino)benzoate	0	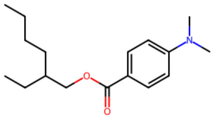	0.732
Tris(2-ethylhexyl) trimellitate	0	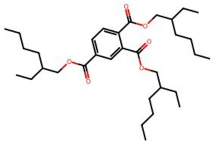	0.690
Di(2-ethylhexyl) phthalate	0	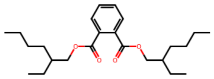	0.650
Butylparaben	0	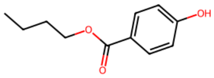	0.605

Query Compound: Sodium tridecyl sulfate

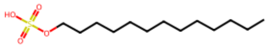

Predicted Activity: 1.0, Votes: 6.0, Docking Score: -3.23

Name	Hit2	Structure	Tanimoto Index
Sodium nonyl sulfate	1	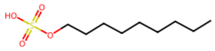	1.000
Octadecyl sulfate sodium salt	0	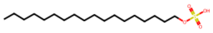	1.000
Sodium decyl sulfate	1	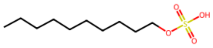	1.000
Sodium octyl sulfate	1	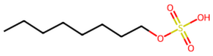	1.000
Sodium ethasulfate	0	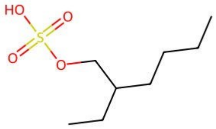	0.469

Query Compound: 2-(Phenylmethylene)octanal

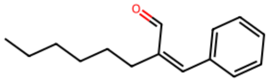

Predicted Activity: 1.0, Votes: 3.0, Docking Score: -6.2

Name	Hit2	Structure	Tanimoto Index
alpha-Amyl cinnamaldehyde	0	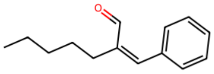	0.967
Heptanophenone	0	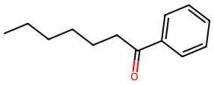	0.486
Dodecylbenzene	0	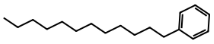	0.389
Octadecanoic acid	0	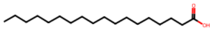	0.382
Tetradecanoic acid	0	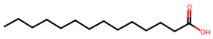	0.382

Query Compound: N-(Cyclohexylthio)phthalimide

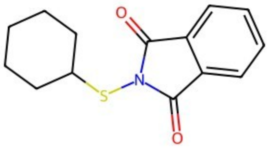

Predicted Activity: 1.0, Votes: 3.0, Docking Score: -6.17

Name	Hit2	Structure	Tanimoto Index
N-Methylphthalimide	0	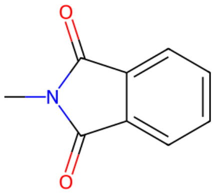	0.375
Thalidomide	0	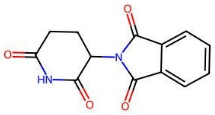	0.366
Cyclohexylphenylketone	0	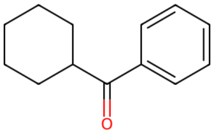	0.316
Dicyclohexyl disulfide	0	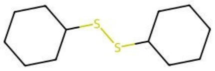	0.300

Dicyclohexyl phthalate	0	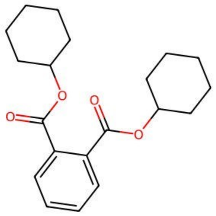	0.300

Query Compound: 17-((1-Oxohexyl)oxy)pregn-4-ene-3,20-dione

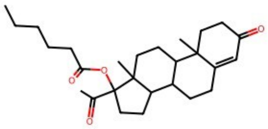

Predicted Activity: 1.0, Votes: 2.0, Docking Score: -7.16

Name	Hit2	Structure	Tanimoto Index
17alpha-Hydroxyprogesterone	0	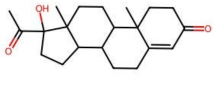	0.590
17-Methyltestosterone	0	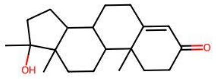	0.550
4-Androstene-3,17-dione	0	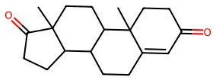	0.483

Corticosterone	0	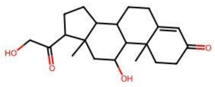	0.368
Melengestrol acetate	1	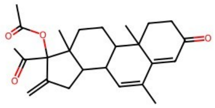	0.354

Query Compound: Pioglitazone hydrochloride

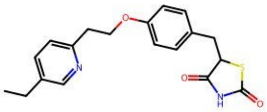

Predicted Activity: 0.0, Votes: 1.0, Docking Score: -7.97

Name	Hit2	Structure	Tanimoto Index
4-(Hexyloxy)phenol	0	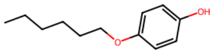	0.255
4-Butyloxyaniline	0	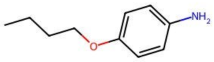	0.245

Imazethapyr	0	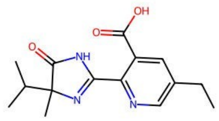	0.243
4-Hexyloxyaniline	0	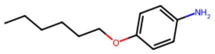	0.232
Dipropyl 2,5-pyridinedicarboxylate	0	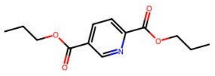	0.230

Query Compound: Troglitazone

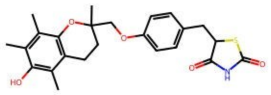

Predicted Activity: 0.0, Votes: 1.0, Docking Score: -7.91

Name	Hit2	Structure	Tanimoto Index
dl-alpha-Tocopheryl acetate	0	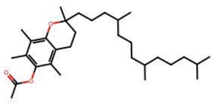	0.300

Dihydrojasmone lactone	0	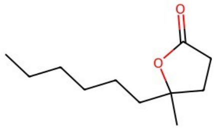	0.200
4-(Hexyloxy)phenol	0	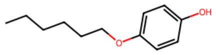	0.194
Eosin	1	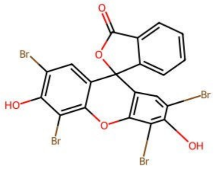	0.177
Atenolol	0	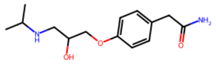	0.177

Query Compound: PharmaGSID_48511

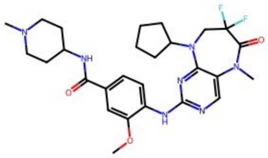

Predicted Activity: 0.0, Votes: 0.0, Docking Score: -8.91

Name	Hit2	Structure	Tanimoto Index

AVE3247	0	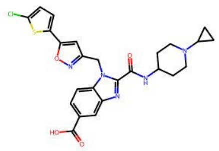	0.221
PHA-00568487	0	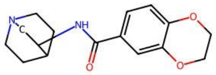	0.220
SB413217A	0	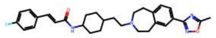	0.219
Hexazinone	0	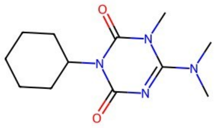	0.212
(R)-N-(Quinuclidin-3-yl)furo(2,3-C)pyridine-5-carboxamide	0	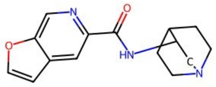	0.208

Query Compound: PharmaGSID_48172

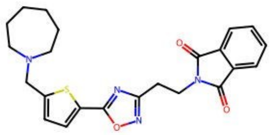

Predicted Activity: 0.0, Votes: 1.0, Docking Score: -7.8

Name	Hit2	Structure	Tanimoto Index
N-Methylphthalimide	0	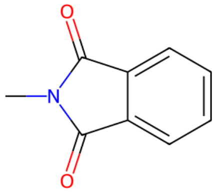	0.241
Phosmet	0	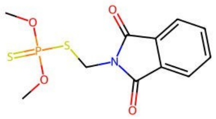	0.227
Thalidomide	0	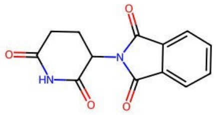	0.197
Raloxifene hydrochloride	0	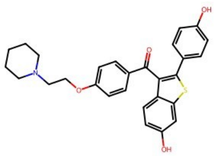	0.188
SSR69071	0	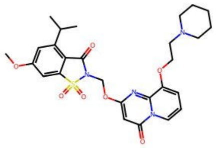	0.188

Query Compound: Thiodicarb

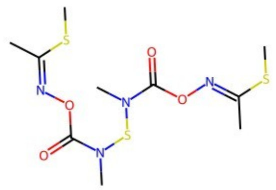

Predicted Activity: 0.0, Votes: 4.0, Docking Score: -4.54

Name	Hit2	Structure	Tanimoto Index
Methomyl	0	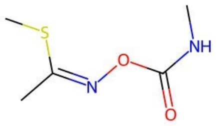	0.516
Oxamyl	0	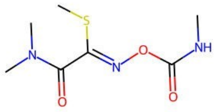	0.333
N,N-Dimethylacetamide	0	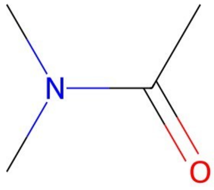	0.259
N-Nitroso-N-methylurea	0	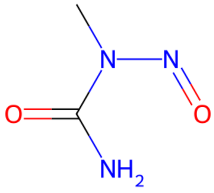	0.250
Methacrylamide	0	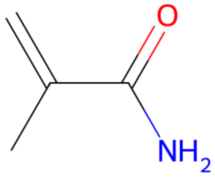	0.241

Query Compound: CP-100829

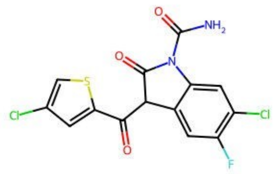

Predicted Activity: 0.0, Votes: 1.0, Docking Score: -7.78

Name	Hit2	Structure	Tanimoto Index
Carbamazepine	0	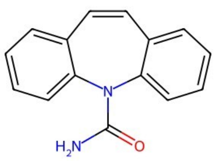	0.236
Flufenpyr-ethyl	0	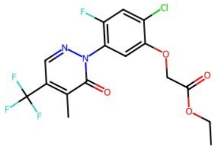	0.210
C.I. Acid Yellow 17, disodium salt	0	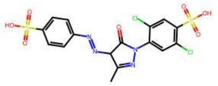	0.190
AVE2865	0	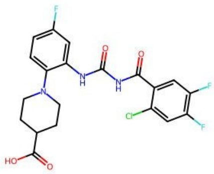	0.185
5-Chloro-2-methyl-3(2H)-isothiazolone	0	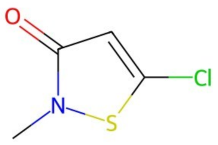	0.182

Query Compound: Zearalenone

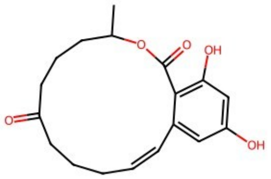

Predicted Activity: 0.0, Votes: 1.0, Docking Score: -7.61

Name	Hit2	Structure	Tanimoto Index
Cyclopentanone	0	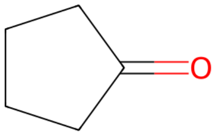	0.205
4-Cyclohexylcyclohexanone	0	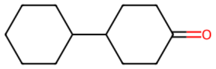	0.200
gamma-Heptalactone	0	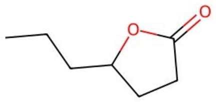	0.200
Cyclohexanone	0	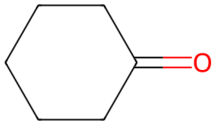	0.200

4-tert-Butylcyclohexanone	0	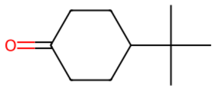	0.196

Query Compound: Fabesetron hydrochloride

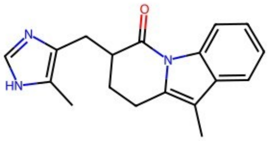

Predicted Activity: 0.0, Votes: 1.0, Docking Score: -7.59

Name	Hit2	Structure	Tanimoto Index
UK-156819	0	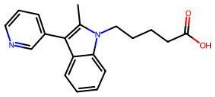	0.213
Thalidomide	0	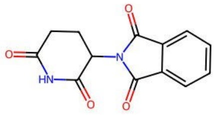	0.206
2',3'-Dideoxyinosine	0	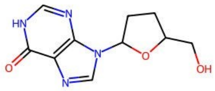	0.205

Cotinine	0	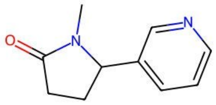	0.197
Theophylline	0	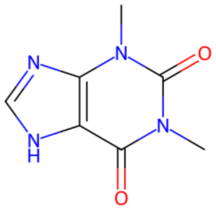	0.197

Query Compound: Tributyltetradecylphosphonium chloride

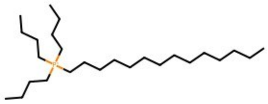

Predicted Activity: 0.0, Votes: 3.0, Docking Score: -5.43

Name	Hit2	Structure	Tanimoto Index
Pentadecane	0		0.533
Decane	0	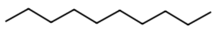	0.533

Hexadecane	0		0.533
Nonane	0	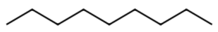	0.533
Undecane	0	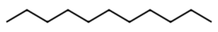	0.533

Query Compound: SAR377142

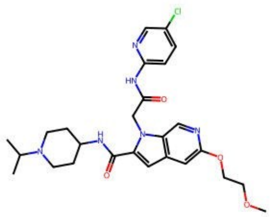

Predicted Activity: 0.0, Votes: 0.0, Docking Score: -8.59

Name	Hit2	Structure	Tanimoto Index
AVE3247	0	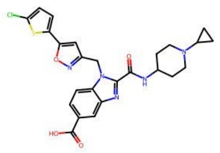	0.239

SR146131 trifluoroacetate (1:1)	0	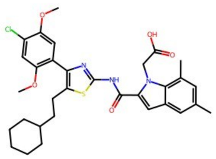	0.226
Candoxatril	0	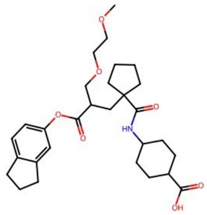	0.222
Hexythiazox	1	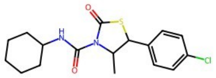	0.220
(R)-N-(Quinuclidin-3-yl)furo(2,3-C)pyridine-5-carboxamide	0	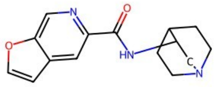	0.215

Query Compound: Dinoseb

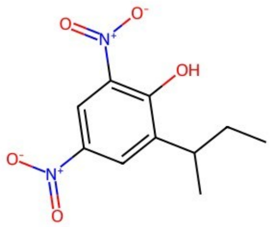

Predicted Activity: 0.0, Votes: 2.0, Docking Score: -6.26

Name	Hit2	Structure	Tanimoto Index

2,4-Dinitrotoluene	0	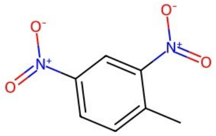	0.375
2-(Butan-2-yl)phenol	0	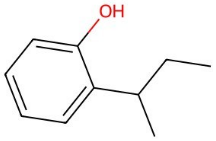	0.359
1-Chloro-2,4-dinitrobenzene	0	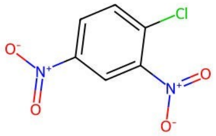	0.341
Isopropalin	0	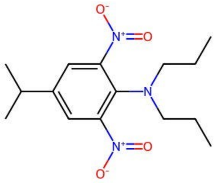	0.340
4-Nitrophenol	0	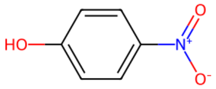	0.316

Query Compound: Bicalutamide

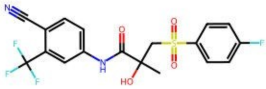

Predicted Activity: 0.0, Votes: 0.0, Docking Score: -8.25

Name	Hit2	Structure	Tanimoto Index
Hydroxyflutamide	0	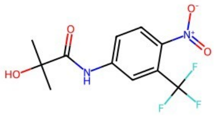	0.403
GSK232420A	0	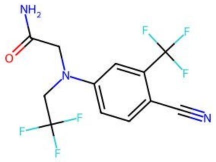	0.348
Flutolanil	1	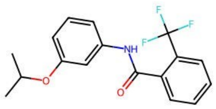	0.290
Fluometuron	0	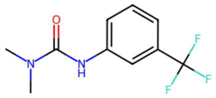	0.246
Flufenacet	0	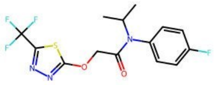	0.234

Query Compound: C.I. Direct Yellow 12

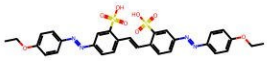

Predicted Activity: 0.0, Votes: 0.0, Docking Score: -8.18

Name	Hit2	Structure	Tanimoto Index
4-Ethoxyaniline	0	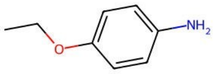	0.326
Disodium 4,4'-bis(2-sulfostyryl)biphenyl	0	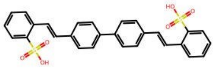	0.320
FD&C; Yellow 6	0	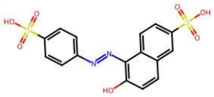	0.304
C.I. Acid Red 1	0	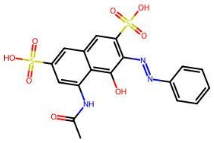	0.284
C.I. Acid Orange 10	0	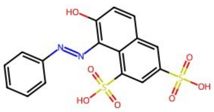	0.283

Query Compound: Nelivaptan

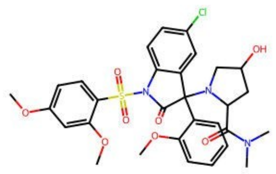

Predicted Activity: 0.0, Votes: 0.0, Docking Score: -8.15

Name	Hit2	Structure	Tanimoto Index
1-(p-Chlorobenzoyl)-5-methoxy-2-methyl-Indole-3-acetic acid	0	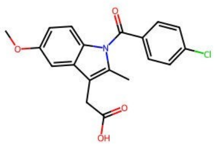	0.223
Sulisobenzone	0	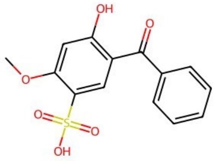	0.218
Indoxacarb	0	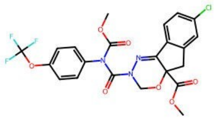	0.205
2-Hydroxy-4-methoxybenzophenone	1	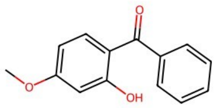	0.205
Dimethomorph	0	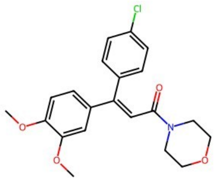	0.202

Query Compound: Progesterone

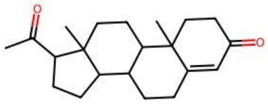

Predicted Activity: 0.0, Votes: 1.0, Docking Score: -7.05

Name	Hit2	Structure	Tanimoto Index
4-Androstene-3,17-dione	0	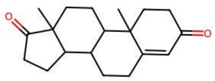	0.617
17alpha-Hydroxyprogesterone	0	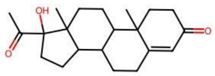	0.615
17-Methyltestosterone	0	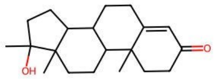	0.600
Corticosterone	0	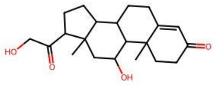	0.542

Spironolactone	0	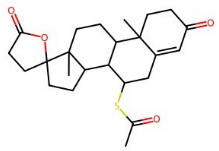	0.382

Query Compound: Methidathion

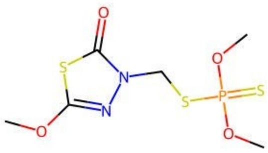

Predicted Activity: 0.0, Votes: 3.0, Docking Score: -4.82

Name	Hit2	Structure	Tanimoto Index
Azinphos-methyl	0	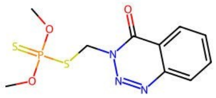	0.440
Phosmet	0	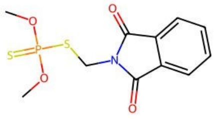	0.422
Dimethoate	0	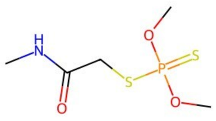	0.349

Malathion	0	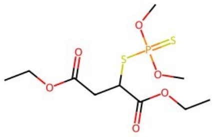	0.255
Azamethiphos	0	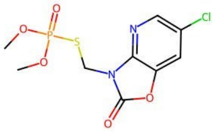	0.230

Query Compound: Biochanin A

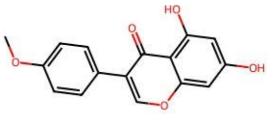

Predicted Activity: 0.0, Votes: 0.0, Docking Score: -8.0

Name	Hit2	Structure	Tanimoto Index
Genistein	0	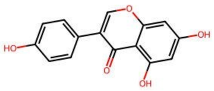	0.750
7,4'-Dihydroxyisoflavone	0	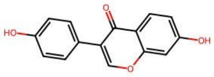	0.478

Morin hydrate	0	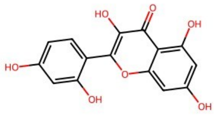	0.408
4-Methoxyphenol	0	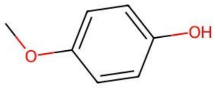	0.350
2-Hydroxy-4-methoxybenzophenone	1	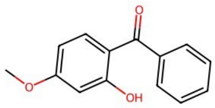	0.308

Query Compound: Apigenin

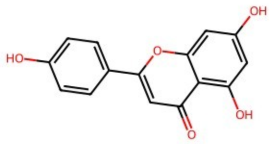

Predicted Activity: 0.0, Votes: 0.0, Docking Score: -7.82

Name	Hit2	Structure	Tanimoto Index
Genistein	0	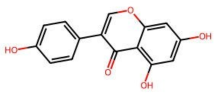	0.500

Morin hydrate	0	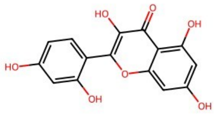	0.476
7,4'-Dihydroxyisoflavone	0	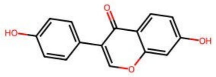	0.356
2,2',4,4'-Tetrahydroxybenzophenone	0	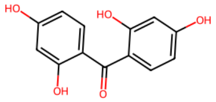	0.282
4-Phenylphenol	0	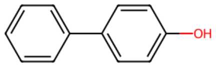	0.278

Query Compound: CP-283097

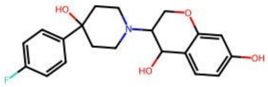

Predicted Activity: 0.0, Votes: 0.0, Docking Score: -7.81

Name	Hit2	Structure	Tanimoto Index

Haloperidol	0	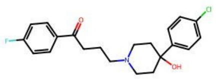	0.309
17alpha-Ethinylestradiol	0	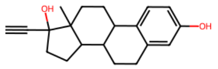	0.271
17beta-Estradiol	0	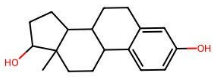	0.250
Estriol	0	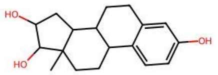	0.246
Estrone	0	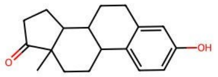	0.232

Query Compound: Kaempferol

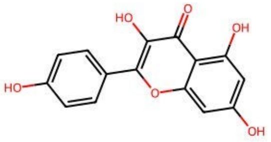

Predicted Activity: 0.0, Votes: 0.0, Docking Score: -7.72

Name	Hit2	Structure	Tanimoto Index
Morin hydrate	0	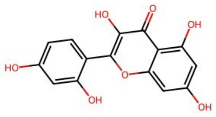	0.750
Genistein	0	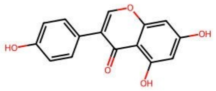	0.561
7,4'-Dihydroxyisoflavone	0	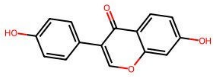	0.378
2,2',4,4'-Tetrahydroxybenzophenone	0	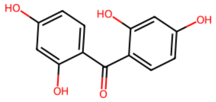	0.275
4-Phenylphenol	0	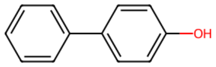	0.270

Query Compound: Quercetin

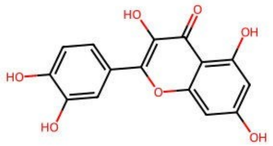

Predicted Activity: 0.0, Votes: 0.0, Docking Score: -7.69

Name	Hit2	Structure	Tanimoto Index
Morin hydrate	0	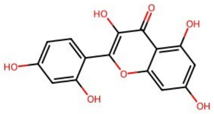	0.667
Genistein	0	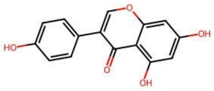	0.467
7,4'-Dihydroxyisoflavone	0	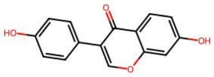	0.306
2,2',4,4'-Tetrahydroxybenzophenone	0	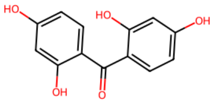	0.233
Methyldopa sesquihydrate	0	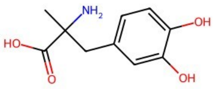	0.212

Query Compound: Diphenylamine

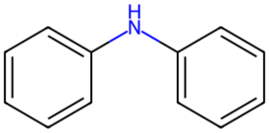

Predicted Activity: 0.0, Votes: 2.0, Docking Score: -5.48

Name	Hit2	Structure	Tanimoto Index
N-Phenyl-1,4-benzenediamine	0	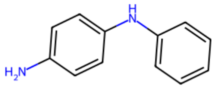	0.667
1,2-Diphenylhydrazine	0	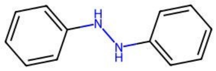	0.600
4-Nitrosodiphenylamine	0	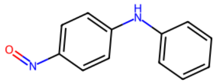	0.571
4-(2-Phenylpropan-2-yl)-N-[4-(2-phenylpropan-2-yl)phenyl]aniline	0	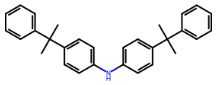	0.500
Pyrimethanil	0	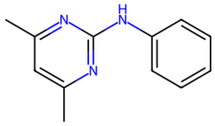	0.458

Query Compound: Sodium myristyl sulfate

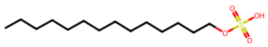

Predicted Activity: 0.0, Votes: 4.0, Docking Score: -3.1

Name	Hit2	Structure	Tanimoto Index
Sodium nonyl sulfate	1	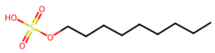	1.000
Octadecyl sulfate sodium salt	0	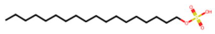	1.000
Sodium decyl sulfate	1	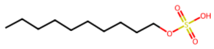	1.000
Sodium octyl sulfate	1	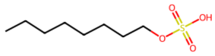	1.000

Sodium ethasulfate	0	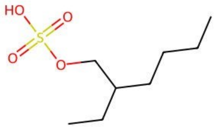	0.469

Query Compound: Equilin

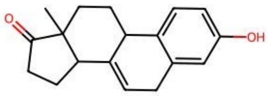

Predicted Activity: 0.0, Votes: 0.0, Docking Score: -7.42

Name	Hit2	Structure	Tanimoto Index
Estrone	0	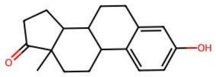	0.500
17beta-Estradiol	0	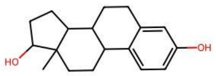	0.350
Estriol	0	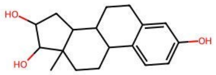	0.344

17alpha-Ethinylestradiol	0	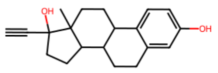	0.328
Dehydroepiandrosterone	0	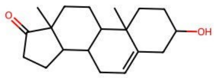	0.295

Query Compound: Octylbicycloheptenedicarboximide

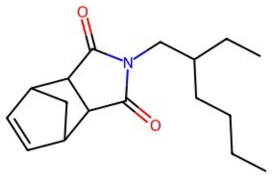

Predicted Activity: 0.0, Votes: 1.0, Docking Score: -6.33

Name	Hit2	Structure	Tanimoto Index
Tris(2-ethylhexyl) phosphate	0	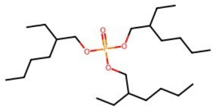	0.341
Bis(2-ethylhexyl) terephthalate	0	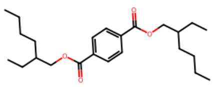	0.327

Bis(2-ethylhexyl) phosphate	0	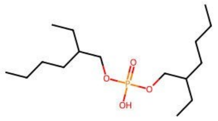	0.326
2-Ethyl-1-hexanol	0	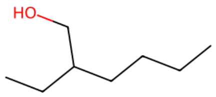	0.325
2-Ethylhexyl diphenyl phosphate	0	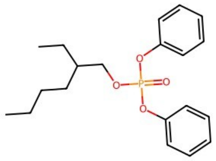	0.321

Query Compound: 1,4-Dihydroxy-2-naphthoic acid

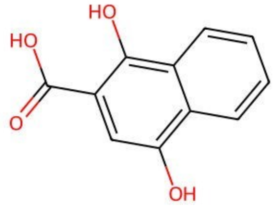

Predicted Activity: 0.0, Votes: 0.0, Docking Score: -7.41

Name	Hit2	Structure	Tanimoto Index
Salicylic acid	0	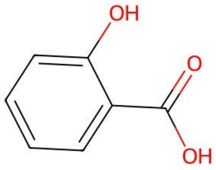	0.500

Monopotassium phthalate	0	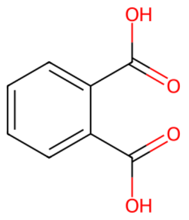	0.462
2-Hydroxybenzophenone	0	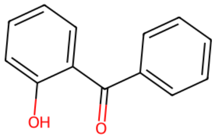	0.364
2,4-Dihydroxybenzophenone	0	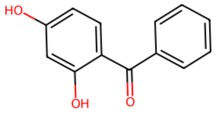	0.361
Sodium benzoate	0	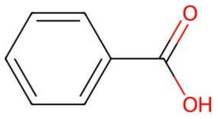	0.357

Query Compound: Oryzalin

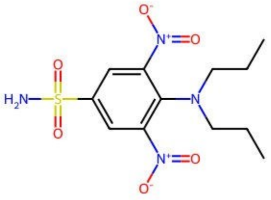

Predicted Activity: 0.0, Votes: 1.0, Docking Score: -6.28

Name	Hit2	Structure	Tanimoto Index

Isopropalin	0	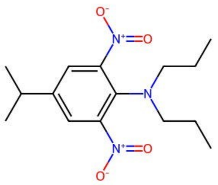	0.600
Trifluralin	1	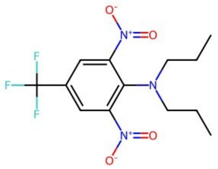	0.585
Flumetralin	1	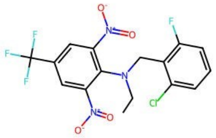	0.317
4-Toluenesulfonamide	0	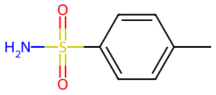	0.308
Mesotrione	0	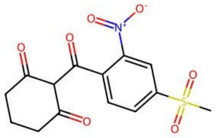	0.286

Query Compound: Flumioxazin

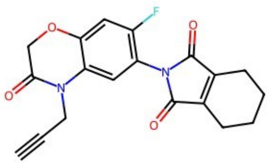

Predicted Activity: 0.0, Votes: 0.0, Docking Score: -7.31

Name	Hit2	Structure	Tanimoto Index
N-Methyl-2-pyrrolidone	0	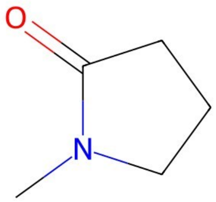	0.173
1-Octyl-2-pyrrolidone	0	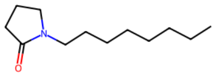	0.167
N-Vinyl-2-pyrrolidone	0	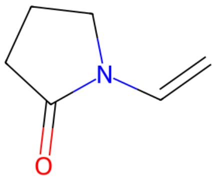	0.164
Fandosentan potassium salt	0	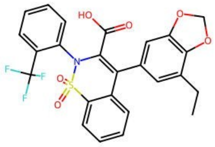	0.157
AVE8923	0	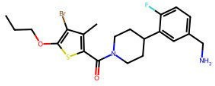	0.157

Query Compound: Flumiclorac-pentyl

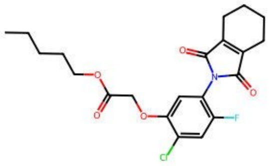

Predicted Activity: 0.0, Votes: 1.0, Docking Score: -6.22

Name	Hit2	Structure	Tanimoto Index
2,4-D Butyl ester	0	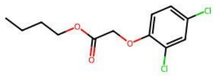	0.475
Flufenpyr-ethyl	0	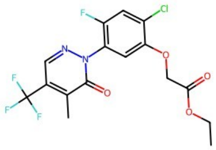	0.444
2,4-D-Butotyl	0	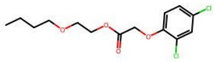	0.422
Triclopyr butotyl	0	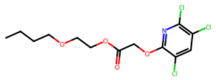	0.373
Pyraflufen-ethyl	0	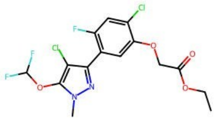	0.368

Query Compound: 4,4'-Dithiodimorpholine

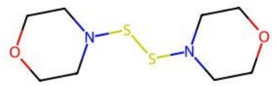

Predicted Activity: 0.0, Votes: 2.0, Docking Score: -5.06

Name	Hit2	Structure	Tanimoto Index
4-Methylmorpholine	0	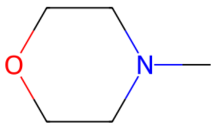	0.421
4-Morpholinepropanamine	0	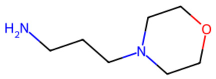	0.308
4-Dodecylmorpholine	0	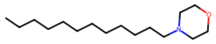	0.286
Morpholine	0	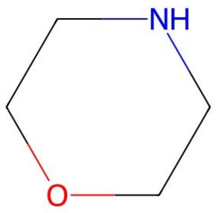	0.250
7a-Ethyldihydro-1H,3H,5H-oxazolo(3, 4-c)oxazole	0	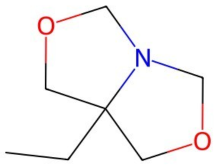	0.179

Query Compound: Tetramethrin

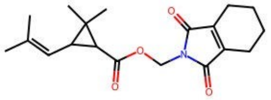

Predicted Activity: 0.0, Votes: 0.0, Docking Score: -7.18

Name	Hit2	Structure	Tanimoto Index
Phenothrin	0	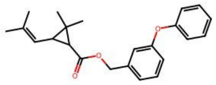	0.410
Resmethrin	0	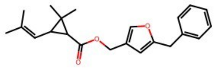	0.391
Tefluthrin	0	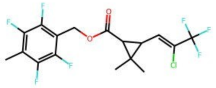	0.333
Permethrin	0	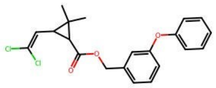	0.323

Bifenthrin	0	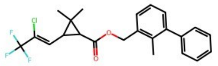	0.296

Query Compound: Thiram

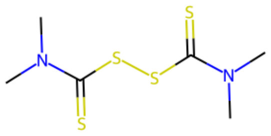

Predicted Activity: 0.0, Votes: 2.0, Docking Score: -4.78

Name	Hit2	Structure	Tanimoto Index
Tetraethylthiuram disulfide	0	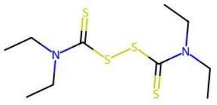	0.450
N,N-Dimethylacetamide	0	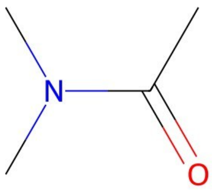	0.278
Dimethylcarbamoyl chloride	0	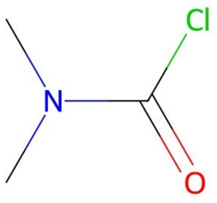	0.263

Trimethylamine	0	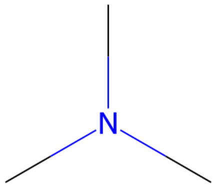	0.214
N,N'-Dimethylthiourea	0	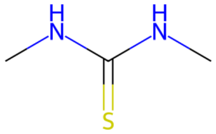	0.211

Query Compound: 2-tert-Butylphenol

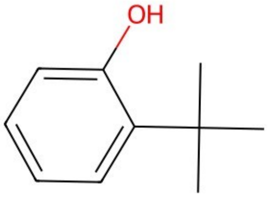

Predicted Activity: 0.0, Votes: 0.0, Docking Score: -6.85

Name	Hit2	Structure	Tanimoto Index
4-Methyl-2-tert-butylphenol	0	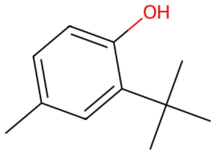	0.560
tert-Butylhydroquinone	0	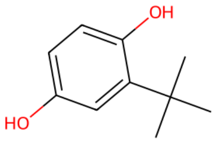	0.520

2,5-Di-tert-butylbenzene-1,4-diol	1	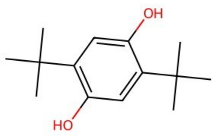	0.500
2,4-Di-tert-butylphenol	1	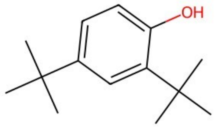	0.500
1,?2-?Benzenediol	0	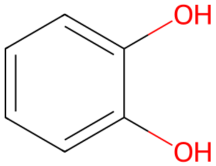	0.474

Query Compound: 2,2'-(Tetradecylimino)diethanol

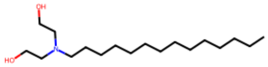

Predicted Activity: 0.0, Votes: 1.0, Docking Score: -5.71

Name	Hit2	Structure	Tanimoto Index
N,N,N',N'-Tetrabutyl-1,6-hexanediamine	0	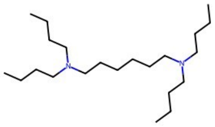	0.650

Tributylamine	0	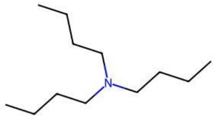	0.550
Triethanolamine	0	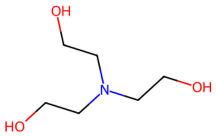	0.526
1-Tridecanol	0		0.524
1-Pentadecanol	0		0.524

Query Compound: Dicloran

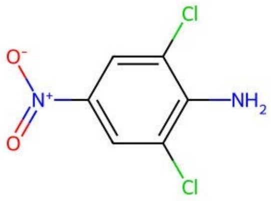

Predicted Activity: 0.0, Votes: 0.0, Docking Score: -6.77

Name	Hit2	Structure	Tanimoto Index

3,4-Dichloronitrobenzene	0	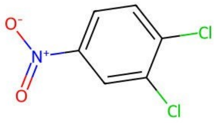	0.556
1-Chloro-2,4-dinitrobenzene	0	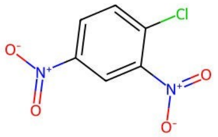	0.467
1-Chloro-4-nitrobenzene	0	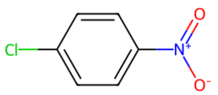	0.444
4-Nitroaniline	0	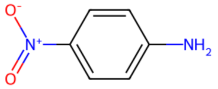	0.444
1,3-Dinitrobenzene	0	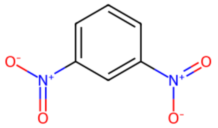	0.407

Query Compound: Piperonyl butoxide

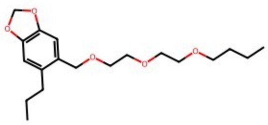

Predicted Activity: 0.0, Votes: 0.0, Docking Score: -6.68

Name	Hit2	Structure	Tanimoto Index
3,6,9,12-Tetraoxahexadecan-1-ol	0		0.342
2-(2-Butoxyethoxy)ethanol	0	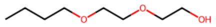	0.342
2-[2-(2-Butoxyethoxy)ethoxy]ethanol	0	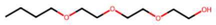	0.342
2-Butoxyethyl acetate	0	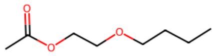	0.341
Triclopyr butotyl	0	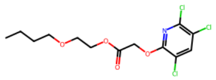	0.321

Query Compound: Docusate sodium

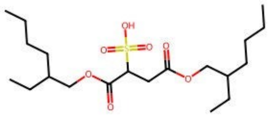

Predicted Activity: 0.0, Votes: 0.0, Docking Score: -6.64

Name	Hit2	Structure	Tanimoto Index
Bis(2-ethylhexyl)hexanedioate	0	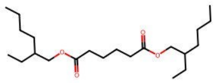	0.605
Bis(2-ethylhexyl) decanedioate	0	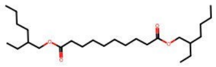	0.590
Bis(2-ethylhexyl) nonanedioate	0	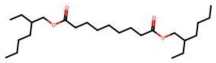	0.590
2-Ethylhexyl thioglycolate	0	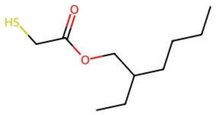	0.590
Sodium 1,4-diisobutyl sulfosuccinate	0	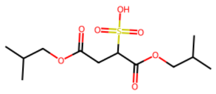	0.579

Query Compound: Methyltrioctylammonium chloride

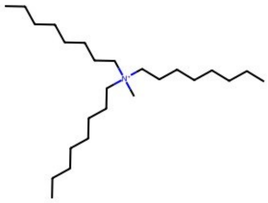

Predicted Activity: 0.0, Votes: 1.0, Docking Score: -5.52

Name	Hit2	Structure	Tanimoto Index
N,N-Dibutyl-N-methylbutan-1-aminium chloride	0	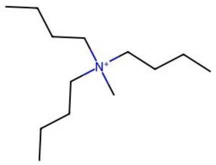	0.750
N,N-Dimethyldecylamine oxide	0	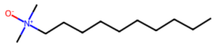	0.600
Hexadecane	0		0.533
Decane	0	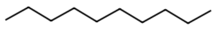	0.533
Undecane	0	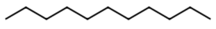	0.533

Query Compound: Sulfasalazine

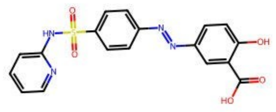

Predicted Activity: 0.0, Votes: 0.0, Docking Score: -6.56

Name	Hit2	Structure	Tanimoto Index
C.I. Acid Orange 7	0	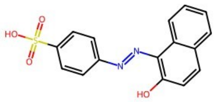	0.333
Sulfaquinoxaline	0	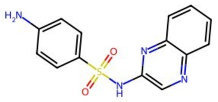	0.317
C.I. Acid Orange 10	0	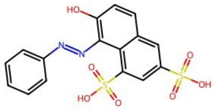	0.312
Salicylic acid	0	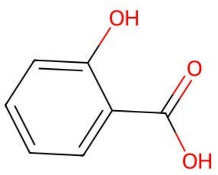	0.312

FD&C; Yellow 6	0	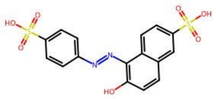	0.311

Query Compound: Sodium hexyldecyl sulfate

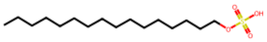

Predicted Activity: 0.0, Votes: 1.0, Docking Score: -5.44

Name	Hit2	Structure	Tanimoto Index
Sodium nonyl sulfate	1	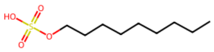	1.000
Octadecyl sulfate sodium salt	0	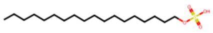	1.000
Sodium decyl sulfate	1	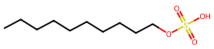	1.000

Sodium octyl sulfate	1	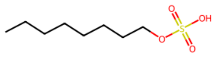	1.000
Sodium ethasulfate	0	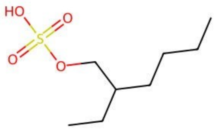	0.469

Query Compound: 2-Tert-Butyl-5-methylphenol

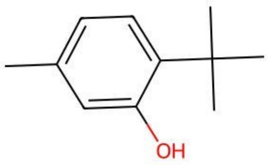

Predicted Activity: 0.0, Votes: 0.0, Docking Score: -6.52

Name	Hit2	Structure	Tanimoto Index
4-Methyl-2-tert-butylphenol	0	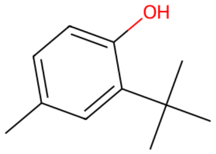	0.708
2,5-Di-tert-butylbenzene-1,4-diol	1	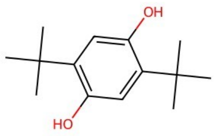	0.591

2,5-Dimethylphenol	0	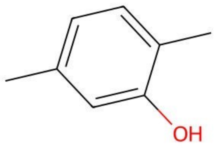	0.542
tert-Butylhydroquinone	0	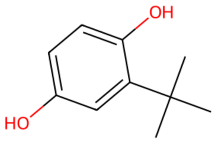	0.481
2,4-Dimethylphenol	0	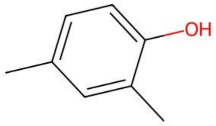	0.480

Query Compound: Octyl gallate

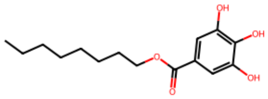

Predicted Activity: 0.0, Votes: 2.0, Docking Score: -4.34

Name	Hit2	Structure	Tanimoto Index
Lauryl gallate	0	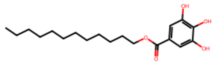	1.000

Propyl gallate	0	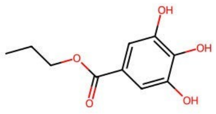	0.750
Octylparaben	0	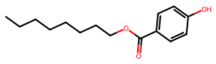	0.639
Hexyl benzoate	0	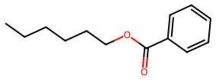	0.611
Tri-n-octyl trimellitate	0	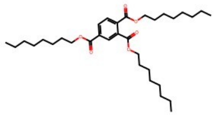	0.590

Query Compound: Besonprodil

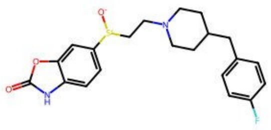

Predicted Activity: 0.0, Votes: 1.0, Docking Score: -5.37

Name	Hit2	Structure	Tanimoto Index

Volinanserin	0	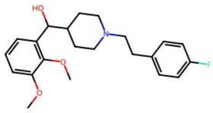	0.247
Haloperidol	0	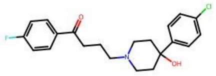	0.213
SB413217A	0	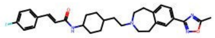	0.208
AVE2865	0	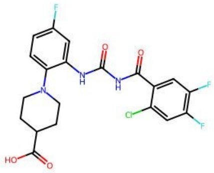	0.193
Enterolactone	0	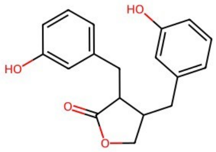	0.192

Query Compound: 1,2-Benzenedicarboxaldehyde

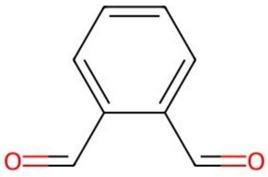

Predicted Activity: 0.0, Votes: 0.0, Docking Score: -6.3

Name	Hit2	Structure	Tanimoto Index
Salicylaldehyde	0	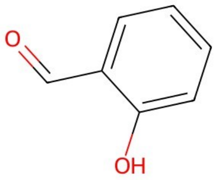	0.632
Sodium 2-formylbenzenesulfonate	0	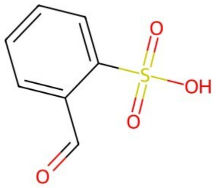	0.522
Phenylacetaldehyde	1	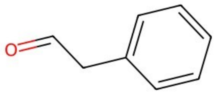	0.381
(2E)-3-Phenylprop-2-enal	0	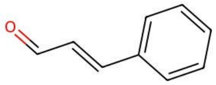	0.348
Furfural	0	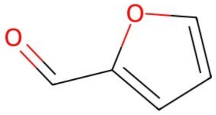	0.348

Query Compound: 2,3-Diaminotoluene

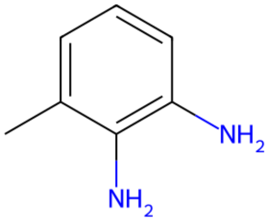

Predicted Activity: 0.0, Votes: 0.0, Docking Score: -6.3

Name	Hit2	Structure	Tanimoto Index
2,6-Dimethylaniline	0	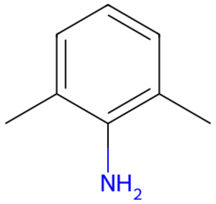	0.722
2-Methylaniline	0	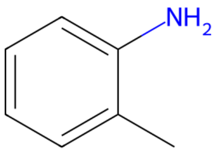	0.600
2-Ethyl-6-methylaniline	0	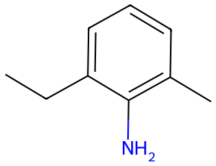	0.520
1,5-Naphthalenediamine	0	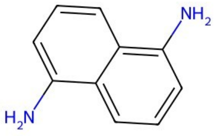	0.429
2,6-Dimethylphenol	0	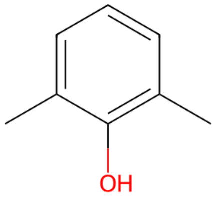	0.409

Query Compound: Fenaminosulf

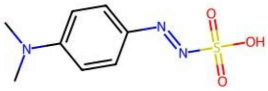

Predicted Activity: 0.0, Votes: 0.0, Docking Score: -6.28

Name	Hit2	Structure	Tanimoto Index
Methyl red	0	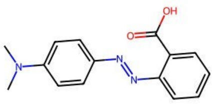	0.450
N,N,4-Trimethylaniline	0	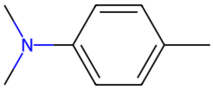	0.367
N,N-Dimethylaniline	0	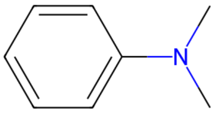	0.333
3-Dimethylaminophenol	0	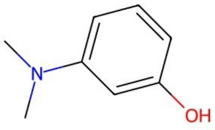	0.278
FD&C; Yellow 6	0	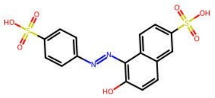	0.271

Query Compound: Cloprop

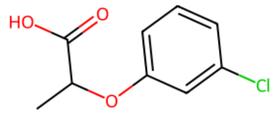

Predicted Activity: 0.0, Votes: 0.0, Docking Score: -6.27

Name	Hit2	Structure	Tanimoto Index
Dichlorprop	0	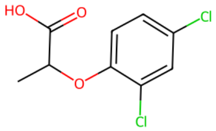	0.583
Mecoprop	0	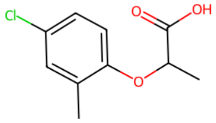	0.568
Chlorpropham	1	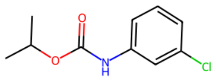	0.439
1,3-Dichlorobenzene	0	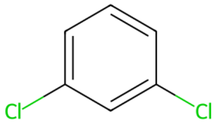	0.379

Diphenyl isophthalate	1	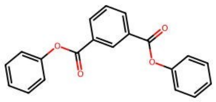	0.342

Query Compound: 3-Trifluoromethyl-4-nitrophenol

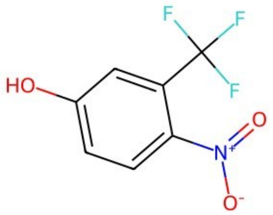

Predicted Activity: 0.0, Votes: 0.0, Docking Score: -6.19

Name	Hit2	Structure	Tanimoto Index
Hydroxyflutamide	0	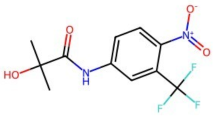	0.524
5,5-Dimethyl-3-(alpha,alpha,alpha-trifluoro-4-nitro-m-tolyl)hydantoin	0	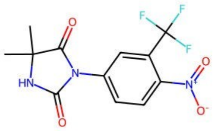	0.489
4-Nitrophenol	0	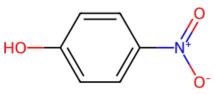	0.406

Sodium 2-nitrophenolate	0	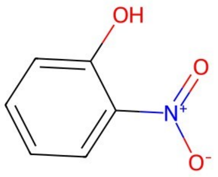	0.382
1,3-Dimethyl-4-nitrobenzene	0	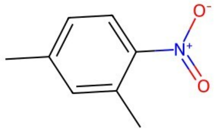	0.361

Query Compound: 1,2-Phenylenediamine

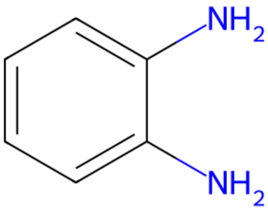

Predicted Activity: 0.0, Votes: 0.0, Docking Score: -6.06

Name	Hit2	Structure	Tanimoto Index
2-Methylaniline	0	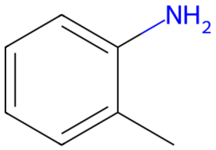	0.562
1,5-Naphthalenediamine	0	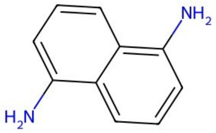	0.533

Aniline hydrochloride	0	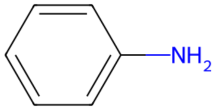	0.500
2-Anisidine	0	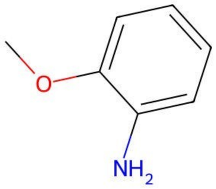	0.474
2-Nitroaniline	0	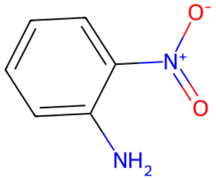	0.429

Query Compound: Oleyl sarcosine

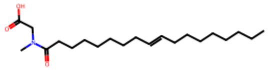

Predicted Activity: 0.0, Votes: 0.0, Docking Score: -6.05

Name	Hit2	Structure	Tanimoto Index
N-Dodecanoyl-N-methylglycine	0	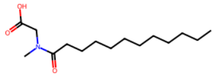	0.806

Oleic acid	0	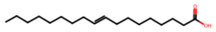	0.636
Linoleic acid	0	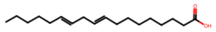	0.583
Ethyl oleate	0	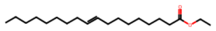	0.526
(9Z,12R)-12-Hydroxyoctadec-9-enoic acid	0	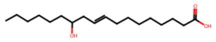	0.524

Query Compound: Didecyldimethylammonium chloride

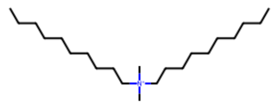

Predicted Activity: 0.0, Votes: 1.0, Docking Score: -4.82

Name	Hit2	Structure	Tanimoto Index

N,N-Dimethyldecylamine oxide	0	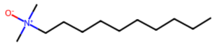	0.600
Nonane	0	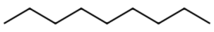	0.533
Hexadecane	0		0.533
Pentadecane	0		0.533
Undecane	0	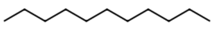	0.533

Query Compound: 2,4-Dinitrophenol

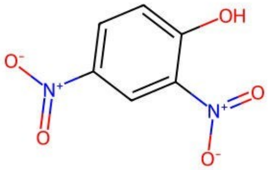

Predicted Activity: 0.0, Votes: 0.0, Docking Score: -5.87

Name	Hit2	Structure	Tanimoto Index
1-Chloro-2,4-dinitrobenzene	0	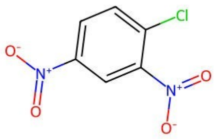	0.586
2,4-Dinitrotoluene	0	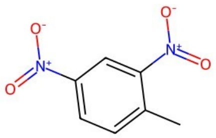	0.586
Sodium 2-nitrophenolate	0	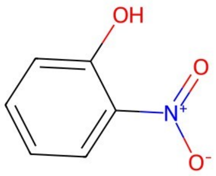	0.536
4-Nitrophenol	0	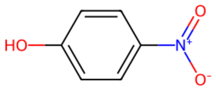	0.519
1,3-Dinitrobenzene	0	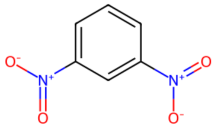	0.481

Query Compound: Thidiazuron

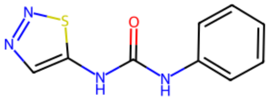

Predicted Activity: 0.0, Votes: 0.0, Docking Score: -5.82

Name	Hit2	Structure	Tanimoto Index
1-Phenylurea	0	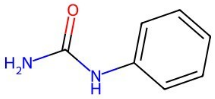	0.400
2-Chloro-N-phenylacetamide	0	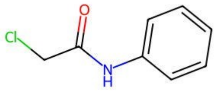	0.368
Fenuron	0	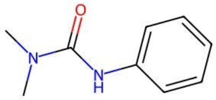	0.368
1,3-Diphenylguanidine	0	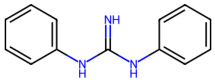	0.343
Propham	0	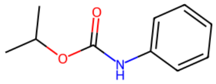	0.341

Query Compound: Ethofumesate

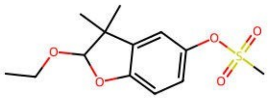

Predicted Activity: 0.0, Votes: 0.0, Docking Score: -5.67

Name	Hit2	Structure	Tanimoto Index
Ethyl methylphenylglycidate	0	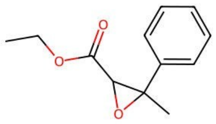	0.224
Ethoxyquin	0	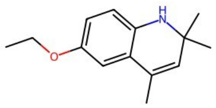	0.220
Paraoxon	0	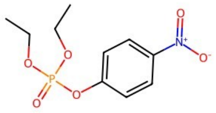	0.211
Diethyl sulfate	0	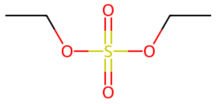	0.209
4-Ethoxyaniline	0	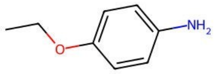	0.208

Query Compound: 2-Methyl-4,6-dinitrophenol

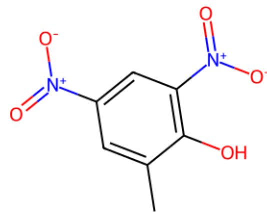

Predicted Activity: 0.0, Votes: 0.0, Docking Score: -5.58

Name	Hit2	Structure	Tanimoto Index
2,4-Dinitrotoluene	0	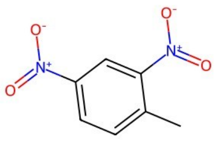	0.548
1,2-Dimethyl-4-nitrobenzene	0	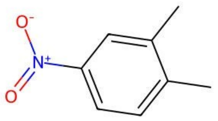	0.484
1,3-Dimethyl-4-nitrobenzene	0	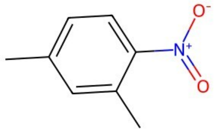	0.424
1-Chloro-2,4-dinitrobenzene	0	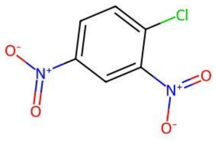	0.412

2-Nitrotoluene	0	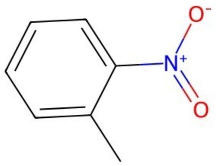	0.406

Query Compound: 1-Phenyl-1H-pyrrole-2,5-dione

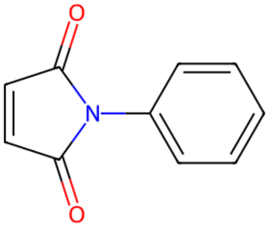

Predicted Activity: 0.0, Votes: 0.0, Docking Score: -5.53

Name	Hit2	Structure	Tanimoto Index
Phthalimide	0	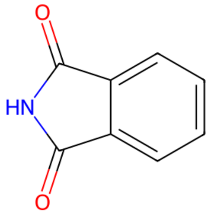	0.320
Chloridazon	0	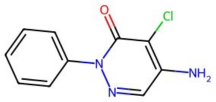	0.306
N-Methylphthalimide	0	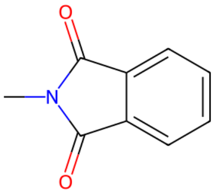	0.296

Diphenyl phosphite	0	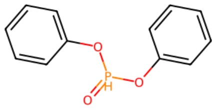	0.296
Biphenyl	0	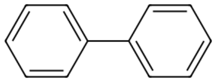	0.286

Query Compound: 2,4,5-Trichlorophenol

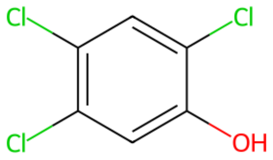

Predicted Activity: 0.0, Votes: 0.0, Docking Score: -5.53

Name	Hit2	Structure	Tanimoto Index
1,2,4,5-Tetrachlorobenzene	0	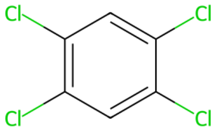	0.571
2,4-Dichlorophenol	0	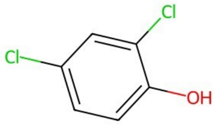	0.550

2,5-Dichlorophenol	0	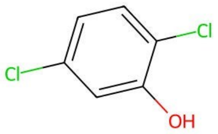	0.550
2,4,6-Trichlorophenol	0	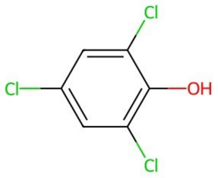	0.400
1,2,4-Trichlorobenzene	0	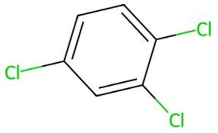	0.400

Query Compound: Dichlone

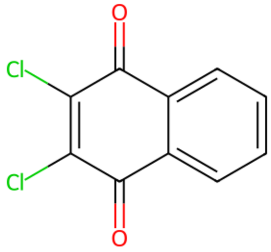

Predicted Activity: 0.0, Votes: 0.0, Docking Score: -5.45

Name	Hit2	Structure	Tanimoto Index
1,4-Diaminoanthraquinone	0	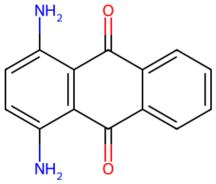	0.458

2-Aminoanthraquinone	0	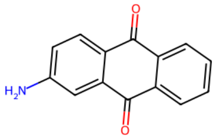	0.423
Phthalimide	0	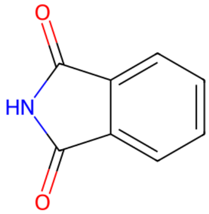	0.409
N-Methylphthalimide	0	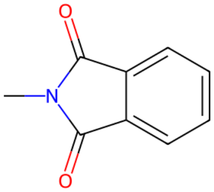	0.375
1,2-Dichlorobenzene	0	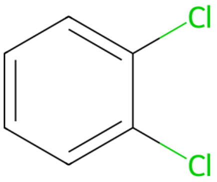	0.368

Query Compound: 1,2-Dibromo-2,4-dicyanobutane

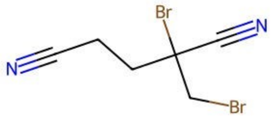

Predicted Activity: 0.0, Votes: 0.0, Docking Score: -5.12

Name	Hit2	Structure	Tanimoto Index

Hexanedinitrile	0	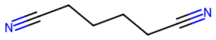	0.304
2-Methylpentanedinitrile	0	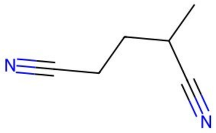	0.233
2,2-Dibromo-3-nitrilopropionamide	0	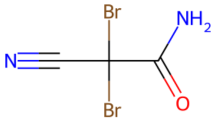	0.233
Propanedinitrile	0	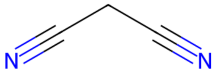	0.217
2-Hydroxy-2-methylpropanenitrile	0	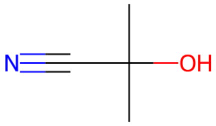	0.192

Query Compound: 4,5-Dichloro-3H-1,2-dithiol-3-one

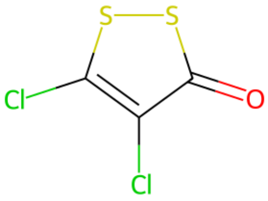

Predicted Activity: 0.0, Votes: 0.0, Docking Score: -5.12

Name	Hit2	Structure	Tanimoto Index
Tetrachlorophthalic anhydride	0	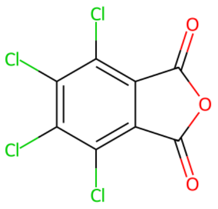	0.250
4,5-Dichloro-2-octyl-3(2H)-isothiazolone	1	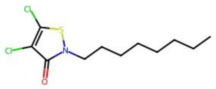	0.229
1,2,3-Trichlorobenzene	0	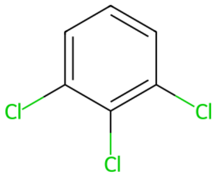	0.227
Clofentezine	0	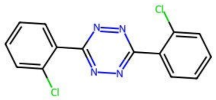	0.222
5-Chloro-2-methyl-3(2H)-isothiazolone	0	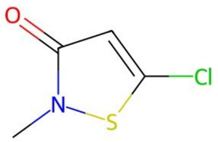	0.207

Query Compound: Octhilinone

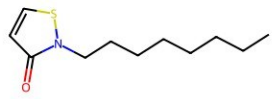

Predicted Activity: 0.0, Votes: 0.0, Docking Score: -5.01

Name	Hit2	Structure	Tanimoto Index
4,5-Dichloro-2-octyl-3(2H)-isothiazolone	1	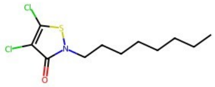	0.447
1-Octyl-2-pyrrolidone	0	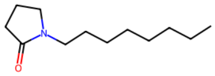	0.359
Dodecylbenzene	0	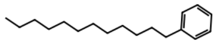	0.343
4-Octylphenol	0	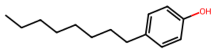	0.324
4-Dodecylphenol	0	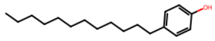	0.324

Query Compound: Triethylene glycol bis(2-ethylhexanoate)

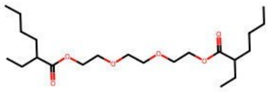

Predicted Activity: 0.0, Votes: 0.0, Docking Score: -4.98

Name	Hit2	Structure	Tanimoto Index
2-Ethylhexanoic acid	0	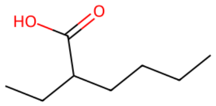	0.533
2-Butoxyethyl acetate	0	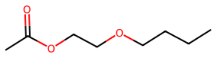	0.500
Butyl lactate	0	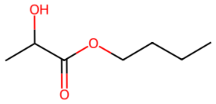	0.424
2-Ethylhexyl acetate	0	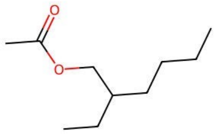	0.417
Bis(2-ethylhexyl) maleate	0	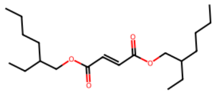	0.405

Query Compound: Riboflavin

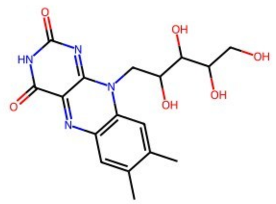

Predicted Activity: 0.0, Votes: 0.0, Docking Score: -4.94

Name	Hit2	Structure	Tanimoto Index
D-Glucitol	0	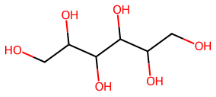	0.213
Stavudine	0	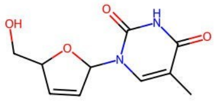	0.209
Arabinose	0	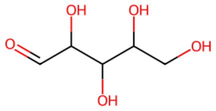	0.208
Imazaquin	0	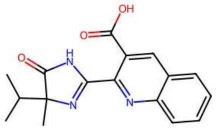	0.189

3'-Azido-3'-deoxythymidine	0	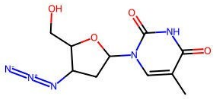	0.179

Query Compound: 1,2-Benzisothiazolin-3-one

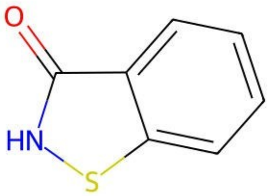

Predicted Activity: 0.0, Votes: 0.0, Docking Score: -4.89

Name	Hit2	Structure	Tanimoto Index
Dibenzothiophene	1	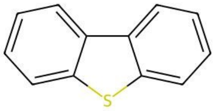	0.400
Phthalimide	0	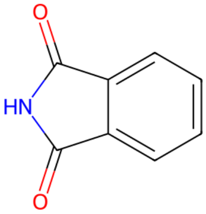	0.286
N-Methylphthalimide	0	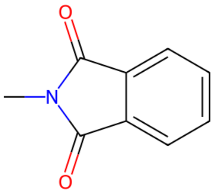	0.267

Diphenyl phosphite	0	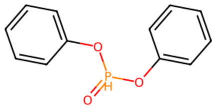	0.267
Sodium saccharin hydrate	0	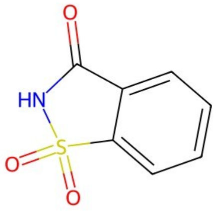	0.265

Query Compound: Diisobutyl phthalate

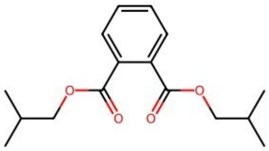

Predicted Activity: 0.0, Votes: 1.0, Docking Score: -3.46

Name	Hit2	Structure	Tanimoto Index
Diethyl phthalate	0	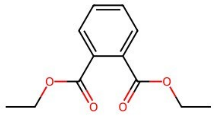	0.607
Di(2-ethylhexyl) phthalate	0	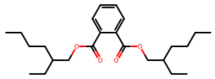	0.556

Diisopropyl phthalate	0	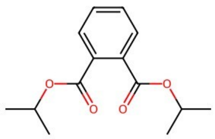	0.552
Dipentyl phthalate	0	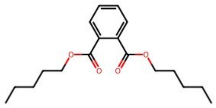	0.515
MEHP	0	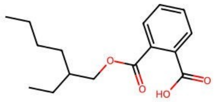	0.500

Query Compound: Laurocapram

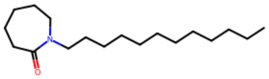

Predicted Activity: 0.0, Votes: 0.0, Docking Score: -4.26

Name	Hit2	Structure	Tanimoto Index
1-Octyl-2-pyrrolidone	0	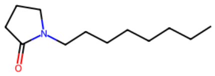	0.862

N-Methyl-2-pyrrolidone	0	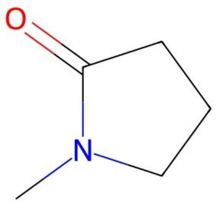	0.364
4-Dodecylmorpholine	0	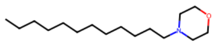	0.351
4,5-Dichloro-2-octyl-3(2H)-isothiazolone	1	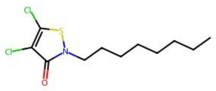	0.333
gamma-Decanolactone	0	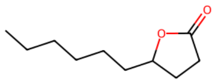	0.310

Query Compound: Triallyl trimellitate

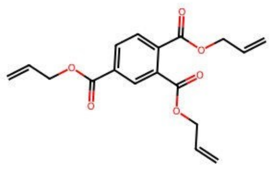

Predicted Activity: 0.0, Votes: 0.0, Docking Score: -4.17

Name	Hit2	Structure	Tanimoto Index

Diallyl phthalate	0	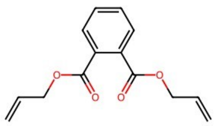	0.656
Tri-n-octyl trimellitate	0	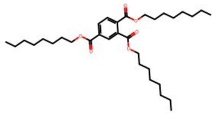	0.525
Tris(2-ethylhexyl) trimellitate	0	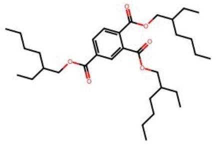	0.467
Diethyl phthalate	0	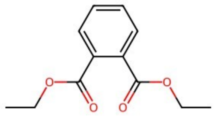	0.351
Ethyl 4-methylbenzoate	0	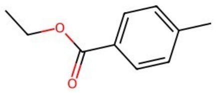	0.342

Query Compound: 1-Dodecyl-2-pyrrolidinone

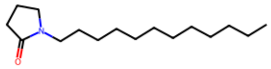

Predicted Activity: 0.0, Votes: 0.0, Docking Score: -3.38

Name	Hit2	Structure	Tanimoto Index
1-Octyl-2-pyrrolidone	0	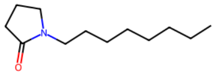	1.000
N-Methyl-2-pyrrolidone	0	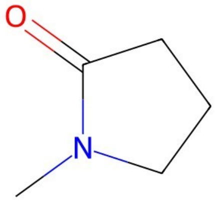	0.433
4-Dodecylmorpholine	0	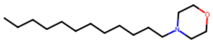	0.371
4,5-Dichloro-2-octyl-3(2H)-isothiazolone	1	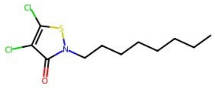	0.350
5-Heptyldihydro-2(3H)-furanone	0	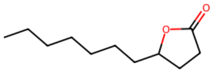	0.325

Query Compound: Hexadecyltrimethylammonium bromide

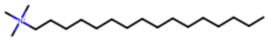

Predicted Activity: 0.0, Votes: 0.0, Docking Score: -2.93

Name	Hit2	Structure	Tanimoto Index
N,N-Dimethyldecylamine oxide	0	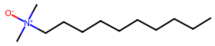	0.600
Hexadecane	0		0.533
Decane	0	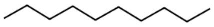	0.533
Pentadecane	0		0.533
Nonane	0	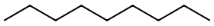	0.533

Query Compound: Dibutyl phthalate

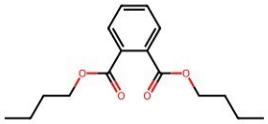

Predicted Activity: 0.0, Votes: 0.0, Docking Score: -2.69

Name	Hit2	Structure	Tanimoto Index
Dipentyl phthalate	0	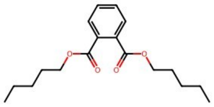	0.889
Monobutyl phthalate	0	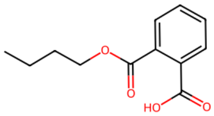	0.862
Octyl decyl phthalate	0	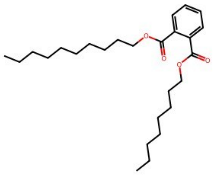	0.857
Dihexyl phthalate	0	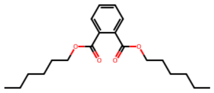	0.857
Di-n-octyl phthalate	0	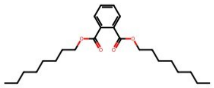	0.857

Query Compound: Myristyltrimethylammonium chloride

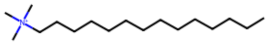

Predicted Activity: 0.0, Votes: 0.0, Docking Score: -2.2

Name	Hit2	Structure	Tanimoto Index
N,N-Dimethyldecylamine oxide	0	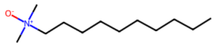	0.600
Hexadecane	0		0.533
Decane	0	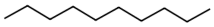	0.533
Pentadecane	0		0.533

Nonane	0	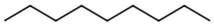	0.533

Query Compound: Dodecyltrimethylammonium chloride

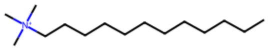

Predicted Activity: 0.0, Votes: 0.0, Docking Score: -2.01

Name	Hit2	Structure	Tanimoto Index
N,N-Dimethyldecylamine oxide	0	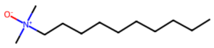	0.600
Hexadecane	0		0.533
Decane	0	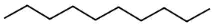	0.533

Pentadecane	0		0.533
Nonane	0	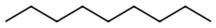	0.533

Query Compound: 8,10-Dodecadien-1-ol

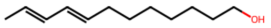

Predicted Activity: 0.0, Votes: 0.0, Docking Score: -0.84

Name	Hit2	Structure	Tanimoto Index
1-Heptanol	0	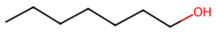	0.360
1-Pentadecanol	0		0.360

1-Dodecanol	0		0.360
1-Hexadecanol	0		0.360
1-Tridecanol	0		0.360

Query Compound: Triphenyltin hydroxide

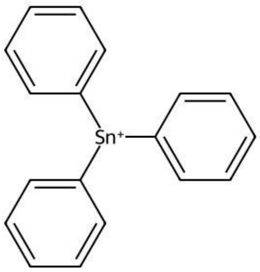

Predicted Activity: nan, Votes: 8.0, Docking Score: nan

Name	Hit2	Structure	Tanimoto Index
Biphenyl	0	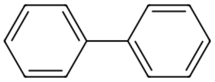	0.400

Aniline hydrochloride	0	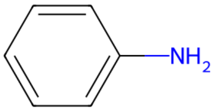	0.353
Phenylmercuric acetate	1	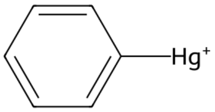	0.353
Benzenethiol	0	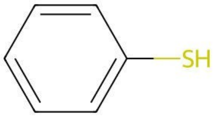	0.353
Phenol	0	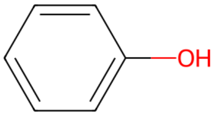	0.353

Query Compound: Ziram

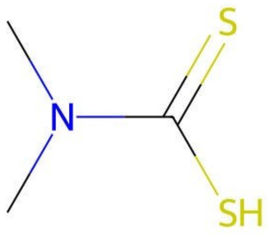

Predicted Activity: nan, Votes: 0.0, Docking Score: nan

Name	Hit2	Structure	Tanimoto Index

Metam-sodium	0	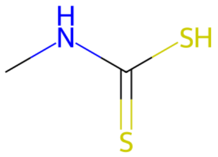	0.375
N,N-Dimethylacetamide	0	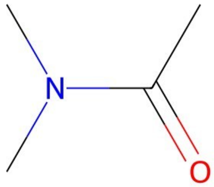	0.312
Dimethylcarbamoyl chloride	0	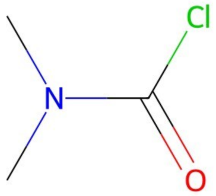	0.294
Maneb	1	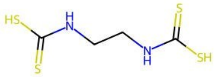	0.263
Trimethylamine	0	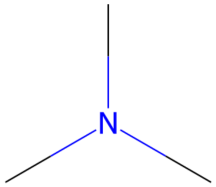	0.250

Query Compound: Tributyltin chloride

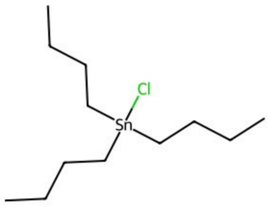

Predicted Activity: nan, Votes: 8.0, Docking Score: nan

Name	Hit2	Structure	Tanimoto Index
Dibutyltin dichloride	1	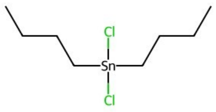	0.647
Butyltin trichloride	0	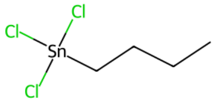	0.647
Decane	0	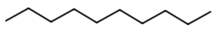	0.375
Pentadecane	0		0.375
Hexadecane	0		0.375

Query Compound: Tetrabutyltin

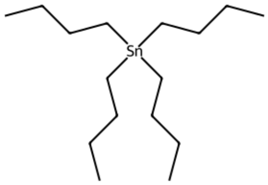

Predicted Activity: nan, Votes: 4.0, Docking Score: nan

Name	Hit2	Structure	Tanimoto Index
Dibutyltin dichloride	1	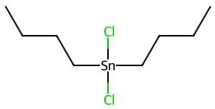	0.529
Butyltin trichloride	0	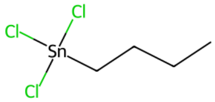	0.529
Nonane	0	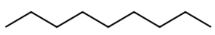	0.429
Pentadecane	0		0.429
Decane	0	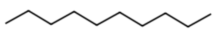	0.429

Query Compound: Tributyltin methacrylate

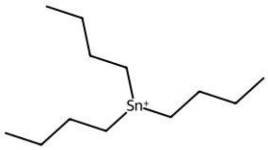

Predicted Activity: nan, Votes: 1.0, Docking Score: nan

Name	Hit2	Structure	Tanimoto Index
Undecane	0	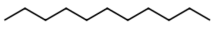	0.429
Nonane	0	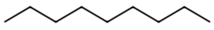	0.429
Hexadecane	0		0.429
Pentadecane	0		0.429
Decane	0	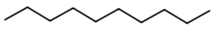	0.429

Additional example cases

Benfluralin

Benfluralin serves as an example of a positive prediction that is explainable by both docking and ML results. It was predicted to be active by all nine ML models and was also classified as active based on molecular docking. Its nearest neighbor, Trifluralin, exhibits a Tanimoto similarity of 0.74 and differs only by a minor N-substituent modification (N-dipropyl instead of N-ethyl-butyl). The second nearest neighbor, Flumetralin (Tanimoto similarity of 0.55), exhibits a substitution of the N-propyl group with a chloro-fluorobenzyl group, which maintains hydrophobic character (see Table S7). Trifluralin and Flumetralin were both experimentally measured active. The close similarity between these three compounds provides strong support for Benfluralin´s predicted bioactivity. Interestingly, Isopropalin (Tanimoto similarity 0.46), which was experimentally measured as inactive, is structurally identical to Trifluralin except for the substitution of a trifluoromethyl group with an isopropyl group on the aromatic ring. Although that difference is relatively small, the trifluoromethyl group appears to be critical for biological activity, likely due to its higher lipophilicity. Although all of the discussed compounds were predicted to be active based on their docking scores, Isopropalin received the least favorable score among them. This trend correlates with experimentally measured activities. Benfluralin exhibits the most favorable score, slightly outperforming Trifluralin, which is consistent with their predicted poses (Fig. S7). The hydrophobic N-propyl of Trifluralin points into the direction of a polar surface, while the N-ethyl group of Benfluralin is shorter and may reduce the unfavorable interaction. Additionally, Benfluralin possesses an additional carbon atom in its second alkyl chain, which extends further into a hydrophobic region of the pocket, likely contributing to a more favorable fit. Summarizing these observations, the positive prediction for Benfluralin should be correct with high certainty.

Table S7. Three nearest neighbors of predicted compound- Benfluralin (highlighted in red), along with their molecular structures and corresponding docking scores. Benfluralin was predicted to be active by both the docking and ML models, as well as by the consensus model. Its two nearest neighbors are measured actives.

**Compound name**	Benfluralin	Trifluralin	Flumetralin	Isopropalin
**Molecular structure**	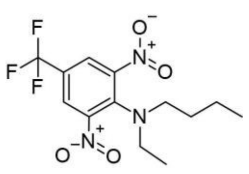	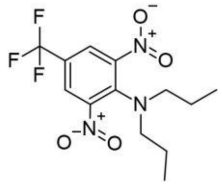	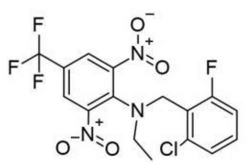	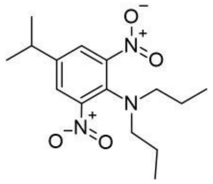
**Docking score**	-7.91	-7.86	-7.50	-6.89
**Tanimoto similarity**		0.74	0.55	0.46
**Classification**	?	active	active	inactive

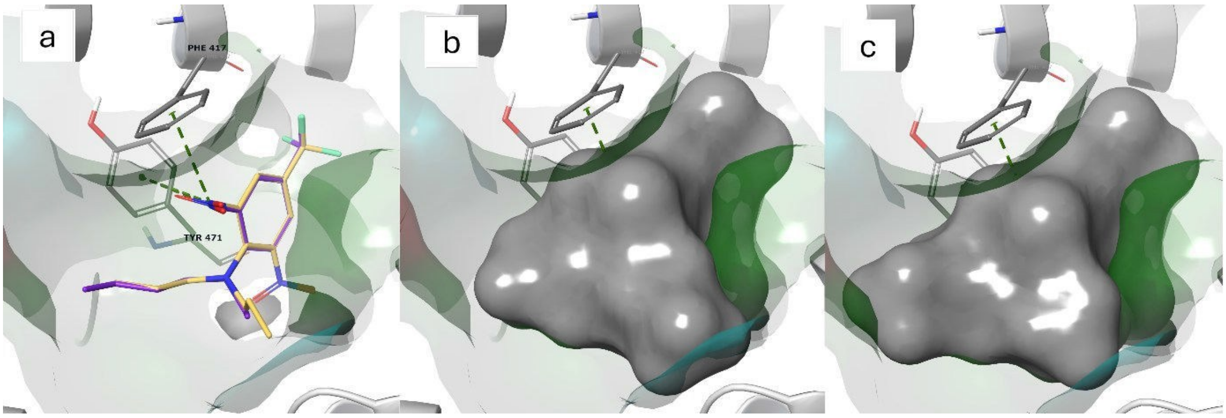

Figure S7. Docking poses of Benfluralin (docking score: -7.91, nine active ML votes) and Trifluralin (docking score: -7.86). The protein surface is colored according to residue properties: green for hydrophobic, red for negatively charged, blue for polar uncharged, and grey for glycine residues. **a**, Binding poses of molecular structures of Benfluralin (in purple)

and Trifluralin (in yellow). Both compounds adopt similar binding poses. Trifluralin contains two N-propyl chains, whereas Benfluralin has one N-butyl and one N-ethyl chain. Green dashed lines represent π–cation interactions. **b**, Molecular surface of Trifluralin. One N-propyl chain extends into the polar, uncharged region of the binding pocket (right front, blue). **c**, Molecular surface of Benfluralin. Compared to Trifluralin, Benfluralin exhibits an improved fit within the binding pocket: its N-butyl chain occupies the hydrophobic region (left front, green) more effectively, while the shorter N-ethyl chain points into the polar uncharged regions (right front, blue), reducing unfavorable interactions.

<u>Octhilinone</u>

Octhilinone represents a case where both docking, and ML results support a negative prediction. It was consistently predicted to be inactive by molecular docking and all nine ML models, while its nearest neighbor, 4,5-Dichloro-2-octyl-3(2H)-isothiazolone (Tanimoto similarity 0.45) was experimentally determined to be active. Structurally, this compound possesses two additional chloro-atoms, which are likely to increase its lipophilicity. The second nearest neighbor, 1-Octyl-2-pyrrolidone (Tanimoto similarity: 0.36), contains a carbon atom in place of the sulfur in the isothiazolone ring, which may result in a slight increase in lipophilicity (see Table S8). Nevertheless, it was measured as inactive. Taken together these observations support the inactive prediction of Octhilinone. According to docking scores, all three compounds were classified as inactive, however the active compound 4,5-Dichloro-2-octyl-3(2H)-isothiazolone received the most favorable score among them, indicating that the docking scores partially reflect the experimental trend in activity.

Table S8. Two nearest neighbors of predicted compound- Octhilinone (highlighted in red), along with their molecular structures and corresponding docking scores. Octhilinone was predicted to be inactive by both the docking and ML models, as well as by the consensus

prediction. Its nearest neighbor 4,5-Dichloro-2-octyl-3(2H)-isothiazolone was experimentally measured active.

Compound name	Octhilinone	4,5-Dichloro-2-octyl-3(2H)-isothiazolone	1-Octyl-2-pyrrolidone
Molecular structure	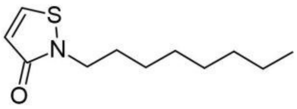	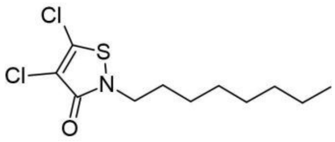	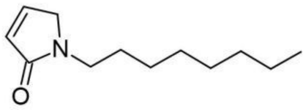
Docking score	-5.01	-5.98	-4.77
Tanimoto similarity		0.45	0.36
Classification	?	active	inactive

Gentian Violet

One instance that is explainable by ML but not by docking results is Gentian Violet. It was predicted to be active by all nine ML models as well as by docking. One of its structural neighbors, Basic Blue 7 shares a Tanimoto similarity of 0.35 and was confirmed to be active in vitro. It appears more lipophilic due to the presence of an additional phenyl ring and extended N-alkyl chains. In contrast, another neighbor Sulfan blue with a Tanimoto similarity of 0.32 was measured inactive (see Table S9). This may be attributed to the exchange of an N-dialkyl group to two sulfonic acid groups, which introduce hydrophilicity and therefore may reduce the binding affinity within the hydrophobic binding pocket. Based on the structural features, the prediction appears plausible. However, the docking scores do not reflect this trend, since Gentian violet received the least favorable score.

Table S9. Neighbors of predicted compound- Gentian Violet (highlighted in red), along with their molecular structures and corresponding docking scores. Gentian violet was predicted to be active by both the docking and ML models, as well as by the consensus prediction.

**Compound name**	Gentian Violet	Basic Blue 7	Sulfan blue
**Molecular structure**	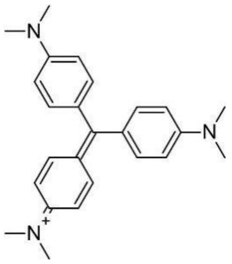	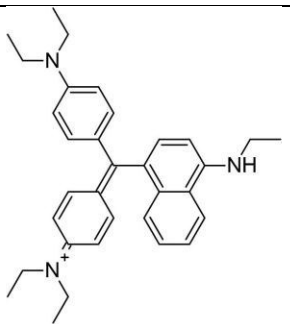	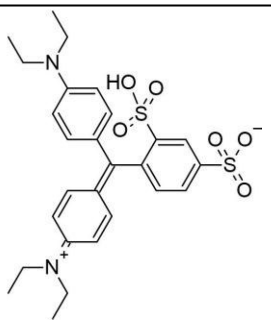
**Docking score**	-7.58	-9.27	-9.00
**Tanimoto similarity**		0.34	0.32
**Classification**	?	active	inactive

2,2'-Methylenebis(4-methyl-6-tert-butylphenol)

2,2'-Methylenebis(ethyl-6-tert-butylphenol) is an example for a prediction that is neither explainable by the ML nor the docking results. It received nine active votes from the ML models and was predicted as active by docking score. Its nearest neighbor (Tanimoto similarity: 0.74) was measured active. However, the second-closest neighbor, 4,4'-Methylenebis(2,6-di-t-butylphenol) (Tanimoto similarity: 0.59), is inactive, despite appearing more structurally similar due to the presence of two substituted phenyl rings (see Table S10). This substance also appears to be more hydrophobic, containing tert-butyl groups instead of ethyl groups.

Additionally, with the inactive compound receiving the most favorable docking score, and one of the active compounds scoring in the inactive range, the docking score fails to show a consistent trend. Given the conflicting results and high structural similarity among the compounds, the overall prediction remains inconclusive.

Table S10. Four nearest neighbors of predicted compound- 2,2'-Methylenebis(ethyl-6-tert- butylphenol) (highlighted in red), along with their molecular structures and corresponding docking scores. 2,2'-Methylenebis(ethyl-6-tert-butylphenol) was predicted to be active by both the docking and ML models, as well as by the consensus model.

**Compound name**	2,2'-Methylenebis(ethyl-6-tert-butylphenol)	2,6-Di-tert-butyl-4-ethylphenol	4,4'-Methylenebis(2,6-di-t-butylphenol)	2,4,6-Tris(tert-butyl)phenol	2,6-Di-tert-butyl-4-methoxyphenol
**Molecular structure**	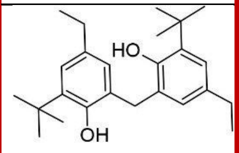	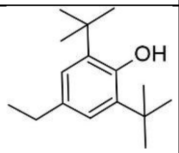	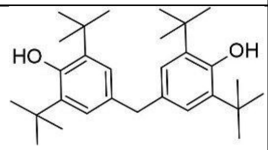	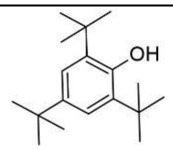	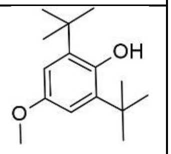
**Docking score**	-8.26	-7.18	-9.45	-6.31	-6.77
**Tanimoto similarity**		0.74	0.59	0.42	0.38
**Classification**	?	active	inactive	active	inactive

<u>Sodium hexyldecyl sulfate and Sodium myristyl sulfate</u>

Only two negatively predicted compounds (Sodium hexyldecyl sulfate and Sodium myristyl sulfate) were identified as being surrounded mainly by positively labeled neighbors. To better understand the model´s decision, two positively predicted analogs (sodium dodecyl sulfate and sodium tridecyl sulfate) were included for comparison. The model appears to apply a “cutoff” at 13 carbon atoms, beyond which alkylsulfates are labeled as inactive. This trend is supported

by a decrease in the number of positive ML votes with increasing carbon chain length. A likely explanation lies in the composition of the training set, where shorter alkyl sulfates (C8-C10) were labeled as active, except for sodium ethasulfate, which contains a branching unit. In contrast, sodium hexyldecyl sulfate was labeled as inactive. The docking score shows an improvement with increasing carbon chain length. However, this trend does not reflect biological reality.

Table S11. Sodium alkylsulfates from the training and the predicted sets**.** The sodium ions were removed during standardization, prior to docking and ML. For training compounds, activity corresponds to the experimentally measured activity. For predicted compounds, activity refers to the classification by the predicted consensus score. “DS” indicates the docking score, and “ML votes” refers to the number of active predictions out of the nine machine learning models.

Training Compounds

**Compound name**	**Molecular structure**	**Activity**	**DS**
Sodium octyl sulfate	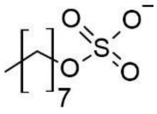	1	-1.98
Sodium nonyl sulfate	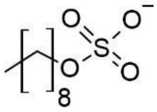	1	-1.93
Sodium ethasulfate	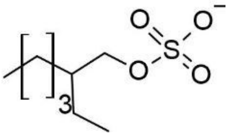	0	-4.64
Sodium decyl sulfate	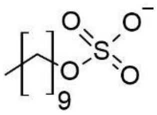	1	-2.11

Predicted Compounds

**Compound name**	**Molecular structure**	**Activity**	**ML votes**	**DS**
Sodium dodecyl sulfate	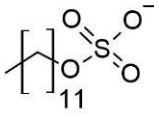	1	8	-2.73
Sodium tridecyl sulfate	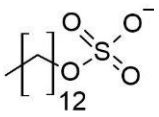	1	6	-3.23
Sodium myristyl sulfate	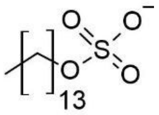	0	4	-3.10
Sodium hexyldecyl sulfate	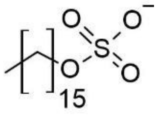	0	1	-5.44

Octadecyl sulfate sodium salt	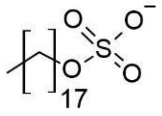	0	-4.82

Acetylcedrene

In the case of Acetylcedrene, the nearest neighbors (shown in Table S12) exhibit relatively low similarities, with Tanimoto coefficients between 0.204 and 0.195. Despite the negative activity labels of its nearest neighbors, Acetylcedrene received a positive prediction with five ML votes. Given the low similarity to the training compounds and their contrasting activity labels, the reliability of this prediction is limited. However, the docking score supports the ML prediction: Acetylcedrene showed a favorable score, while all neighbors except for Sodium abiate were classified as inactive by docking. Even Sodium abiate received a score that is very close to the activity threshold of -6.52, further suggesting that Acetylcedrene may indeed be more active than its neighbors.

Table S12. Five nearest neighbors of predicted compound- Acetylcedrene (highlighted in red), along with their molecular structures and corresponding docking scores. Acetylcedrene was predicted to be active by both the docking and ML models, as well as by the consensus model. All neighbors were classified as inactive in vitro. The sodium ion was removed during standardization.

**Compound name**	Acetylcedrene	Dehydroacetic acid	alpha-Ionone	alpha-Isomethylionone	Sodium abietate	Fenchol
**Molecular structure**	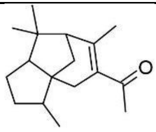	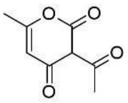	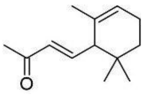	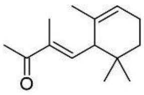	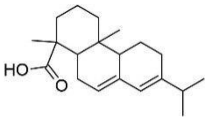	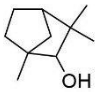
**Docking score**	-7.02	-5.40	-5.31	-5.52	-6.54	-5.83

**Tanimoto similarity**		0.20	0.20	0.20	0.20	0.20
**Classificatio n**	?	inactive	inactive	inactive	inactive	inactive
